# Embodied Cognition and the Structure of Personality: An Exploratory Study of Longitudinal Pathways From Early Psychomotor Function

**DOI:** 10.1111/jopy.13011

**Published:** 2025-02-01

**Authors:** Dimitris I. Tsomokos

**Affiliations:** ^1^ School of Psychology & Neuroscience University of Glasgow Glasgow UK; ^2^ Institute of Education, Department of Psychology and Human Development University College London London UK

**Keywords:** embodied cognition, infant motor control, personality, prosociality, self‐regulation, sensorimotor function, theory of mind

## Abstract

**Objective:**

To explore the developmental pathways linking infant psychomotor function with personality in late adolescence through cognitive, social, and self‐regulation skills. The broader research question, seen through the lens of embodied cognition, is whether cognition and personality in youth develop from basic sensorimotor and communicative systems in infancy.

**Method:**

The sample included 9202 participants from a representative UK birth cohort. A structural equation model examined the prospective associations between motor and communicative functions at age 9 months, cognition, self‐regulation, and prosociality at 5 years, and the five‐factor model of personality at 17 years. The associations between psychomotor function and the meta‐traits of stability and plasticity were also explored.

**Results:**

Even after controlling for confounders and correcting for multiple paths, there was robust evidence that psychomotor development significantly predicts personality structure, with indirect pathways mediated by self‐regulation skills and general or social cognitive skills in middle childhood. While infant communicative function was significantly associated with both meta‐traits, gross motor function was significantly associated with plasticity but not stability.

**Conclusions:**

Early psychomotor function may have long‐term effects on personality, mediated by cognitive, social, and self‐regulation skills. This finding can inform the development of socio‐educational interventions and tailored curricula in early childhood education.

## 1 Introduction

“I can't quite *grasp* this idea,” or “He never got *over* the experience,” or “She was able to see the issue from a different *perspective*.” Perhaps these are not just turns of phrase or mere metaphors (Lakoff and Johnson 1980), but point to something else—a deeper link between cognitive and motor functions. This is, of course, a key premise of embodied cognition, which posits that the body acts as a constraint on, or a distributor‐regulator of, cognitive processes (Lindblom 2015; Shapiro 2019). In recent years, a considerable amount of empirical evidence has emerged in support of embodied cognition (Boem et al. 2024; Castro‐Alonso et al. 2024; Ibanez et al. 2023; Muraki, Speed, and Pexman 2023; Solana 2023; Tuena et al. 2023), although the debate on the broader scope of this approach is still ongoing (Gallagher 2023; Hovhannisyan and Vervaeke 2022; Kersten 2023; Mahon and Caramazza 2008). Along with continuous refinements of the embodied cognition framework, researchers have been trying to understand the associations between embodied cognition and individual differences (Balcetis and Cole 2009; Glenberg 2010; Hovhannisyan and Vervaeke 2022; Meier et al. 2012; Niedenthal et al. 2005; Robinson et al. 2021; Robinson and Irvin 2023), a project that can be seen as part of a wider effort to map the common territory between cognition and personality (Boogert et al. 2018; Cantor and Kihlstrom 2017; Chen et al. 2022; Dougherty and Guillette 2018; Griffin, Guillette, and Healy 2015; Revelle, Wilt, and Rosenthal 2010), with recent findings by Stanek and Ones (2023), for instance, charting more than 60,000 associations among cognitive skills and personality constructs.

Embodied cognition posits that cognitive processes are inherently associated with sensory and motor systems. Grounded cognition, in particular, proposes that the repeated and varied interactions of a developing child with the environment (through these sensorimotor systems) *structure* cognition as these interactions are represented by neuronal correlates in the brain (Barsalou 2008; Shapiro 2019). In other words, cognition is not separate from the sensory and motor systems but rather is grounded on and emerges from them during development (Glenberg 2010; Iverson 2010; Smith and Gasser 2005). From this premise follows the hypothesis that early sensorimotor experiences—such as an infant's ability to grasp, crawl, and communicate through gestures—may be central to the development of higher‐order cognitive processes. For example, reaching for objects or coordinating movement involves not just motor control but also spatial awareness, attention, and problem solving, which are all components of cognition. It would be reasonable to assume then that, by early to middle childhood, such sensorimotor activities will have contributed to the relative maturation of brain systems responsible for these tasks, in turn impacting learning and cognition later in life. In fact, over the last two decades, certain findings have provided support for this hypothesis: Murray et al. (2006) found that within normal developmental ranges (in the general population), earlier maturation in gross motor function was predictive of superior executive function skills; Piek et al. (2008) showed that better infant motor coordination was associated with better working memory and processing speed, while inferior gross motor function was associated with anxiety and depression symptoms at school age (Piek et al. 2010), namely, mental health symptoms that are closely linked with self‐regulation in that age group (Robson, Allen, and Howard 2020; Sawyer et al. 2015; Wyman et al. 2010). Similarly, Grissmer et al. (2010) provided evidence that fine motor skills were associated with a range of cognitive abilities and were predictive of later academic achievement, while Gandotra et al. (2023) showed that fine motor function was a strong predictor of response inhibition, whereas gross motor function was a strong predictor of prosocial behavior in childhood. Zhou and Tolmie (2024) provided further evidence for a prospective association between infant motor function and cognitive skills (including executive function) and academic achievement in late childhood using a longitudinal dataset drawn from the general UK population.

A question of directionality arises at this point, namely, whether ‘higher’ cognitive skills always develop from the more ‘basic’ sensorimotor functions, or the reverse is (perhaps also) true—i.e., could it be that basic forms of cognitive ability are already present in infancy and support motor and communicative functions? While it is plausible that abilities such as early attention and simpler goal‐directed behaviors may influence the development of motor control, the bulk of evidence so far suggests that sensorimotor functions play a larger, initiating role in cognitive development. Infants rely heavily on motor exploration to learn about their environment, and these interactions are prospectively associated with cognitive processes like perception, attention, and executive control (Libertus, Joh, and Needham 2016; Smith and Gasser 2005; Wiesen, Watkins, and Needham 2016). As indicated previously, longitudinal studies further support this pathway, with motor skills in infancy predicting later cognitive abilities (Murray et al. 2006; Piek et al. 2008; Smith and Gasser 2005; Zhou and Tolmie 2024; Zhuo et al. 2022). Notably, in infancy, cognitive control systems and executive functions are still relatively immature, and it would therefore appear unlikely that early cognitive differences would account for individual variation in motor milestones. Instead, the evidence thus far aligns with embodied cognition theories, suggesting that sensorimotor experiences provide the initial structure for emerging cognitive abilities.

On the other hand, it is now known that personality traits are associated with cognitive abilities (Dougherty and Guillette 2018; Griffin, Guillette, and Healy 2015; Lounsbury et al. 2005; Rammstedt, Lechner, and Danner 2018; Stanek and Ones 2023). There are several approaches that can be used to account for the intricate relations between cognitive abilities and personality traits, ranging from research that identifies how individual differences in personality impact cognitive processes (Matthews 1997, 2012; Revelle, Wilt, and Rosenthal 2010), or the Cognitive‐Affective Personality System that views personality as a dynamic system in which cognitive‐affective units mediate personality structure between situations and cognitive or affective responses (Mischel and Ayduk 2002), to the Cybernetic Big Five Theory which proposes that personality traits reflect individual differences in the regulation of goal‐directed behavior, whereas cognitive processes are implicated in setting, monitoring, and achieving goals (DeYoung 2015). It should also be noted that, focusing on early sensorimotor function and later cognition and personality traits, some initial evidence has been provided in support of their interrelation. In particular, Flensborg‐Madsen et al. (2023) have shown that motor function in infancy (later age of attainment) predicts (higher) neuroticism and (lower) conscientiousness in midlife. In a large‐scale, preregistered analysis of data from the Twins Early Development Study, Bowler et al. (2024) provided robust evidence of both phenotypic and genetic links between superior early fine motor skills and later cognitive abilities (evidenced by educational outcomes and years of education), as well as more anxiety symptoms (correlated with trait neuroticism).

Despite this progress, several important gaps remain on the empirical front. First, there remains a lack of large‐scale, longitudinal studies on the association between psychomotor function in infancy and general or social cognitive abilities and executive function in childhood. Second, there is a scarcity of empirical evidence on the prospective associations between infant psychomotor skills and personality later in life. Third, the prospective association between social skills in childhood—such as social competence, mentalizing, and prosocial behaviors—and later personality structure has not been explored yet, and to the best of this author's knowledge, this link has been investigated only cross‐sectionally in adult populations (Thielmann, Hilbig, and Zettler 2022; Thielmann, Spadaro, and Balliet 2020). In addition to these gaps, there also appears to be a lack of research exploring the mediating role of general and social cognitive ability and self‐regulation in the prospective association between psychomotor function in infancy and later personality structure. This mediation hypothesis is motivated by the following logic: firstly, if cognition is embodied, then motor and communicative functions in infancy must be prospectively associated with childhood cognitive abilities and executive function (Balcetis and Cole 2009; Shapiro 2019; Smith and Gasser 2005; Zhou and Tolmie 2024); secondly, if personality is embodied, infant psychomotor functions must also be associated with later personality (Robinson et al. 2021). But, of course, cognition and personality are also associated (Dougherty and Guillette 2018; Stanek and Ones 2023), and—in the framework of embodied cognition—it is precisely the process of development and learning that is assumed to partially shape personality (Castro‐Alonso et al. 2024; Chen et al. 2022).

### 1.1 The Present Study

The present study is exploratory in nature and addresses these gaps in a unifying model, which spans around 17 years of child and adolescent development and controls for a range of confounders (associated both with developmental milestones and cognitive abilities in childhood) that include socioeconomic, neighborhood, family, and individual factors. To this end, the longitudinal dataset of the Millennium Cohort Study (MCS) was employed, as it contains the necessary variables and tracks a large, nationally representative birth cohort from the United Kingdom. A single structural equation model examines (1) the associations between gross motor, fine motor, and communicative function skills at age 9 months and cognitive abilities (verbal and non‐verbal skills), social cognition (theory of mind), prosocial behavior, as well as self‐regulation (emotion regulation and independence skills) at age 5 years; (2) the associations between these psychomotor functions in infancy and the five‐factor model of personality (McCrae and John 1992) at age 17 years; and (3) the role of social ability and cognitive/executive skills as mediators between infant psychomotor functions and adolescent personality. The choice of potential confounders at baseline (age 9 months) was based on variables that are known to impact both sensorimotor functions during infancy and later cognitive ability or personality traits, including a participant's biological sex (Moore 2024; Murray et al. 2007), family income and neighborhood disadvantage (Panceri et al. 2022; Rakesh et al. 2023; Wilkinson et al. 2023), maternal mental health in the perinatal period (Lubotzky‐Gete et al. 2021; Van den Bergh et al. 2020), and birth outcomes such as small for gestational age (Oudgenoeg‐Paz et al. 2017; Theodore et al. 2009; Zhuo et al. 2022). In an additional analysis on meta‐traits of personality structure (DeYoung, Peterson, and Higgins 2002; Digman 1997; Furnham and Cheng 2023; Karwowski and Lebuda 2016; Safron and DeYoung 2021), the five dimensions of Openness, Conscientiousness, Extraversion, Agreeableness, and Neuroticism were loaded onto *stability* (shared variance between conscientiousness, agreeableness, and negative neuroticism, which captures an individual's ability to avoid distractions in goal‐oriented behavior) and *plasticity* (shared variance between openness and extraversion, capturing the ability to explore the environment and generate new goals and strategies), so that the associations between infant psychomotor functions and these two higher‐order factors could be investigated in detail.

## 2 Materials and Methods

### 2.1 Participants and Analytic Sample

The MCS follows the life trajectories of more than 19,000 children born in the four UK countries during 2000–2002 (Joshi and Fitzsimons 2016). The survey's sampling frame was based on electoral wards and ensured over‐representation of families living in high child‐poverty areas and neighborhoods in England with at least 30% ethnic minority populations (Joshi and Fitzsimons 2016; Plewis et al. 2004). The collection of data was carried out via home visits and observations, structured interviews conducted with (and questionnaires completed by) caregivers and cohort members, who were also administered cognitive test batteries in their homes. Anonymized, cleaned, and suitably organized secondary data have been released for the first seven waves of the MCS childhood so far, namely, for the waves at age 9 months, and 3, 5, 7, 11, 14, and 17 years. Processes to obtain ethical approvals were followed prior to each wave by UK Multi‐Centre Ethics Committees led by the National Health Service. The first wave included 18,754 infants (aged approximately 9 months) who were singletons or first‐born twins, or triplets. In the present study, these infants were required to have valid records on the independent (exogenous) variables of psychomotor function, as well as for the outcome (endogenous) variables of the five‐factor model of personality. Under these two conditions, 9202 infants (52% female) remained in the final analytic sample.

### 2.2 Measures and Procedures

*Five‐Factor Model of Personality (17 years)*: The personality traits of openness (O), conscientiousness (C), extraversion (E), agreeableness (A), and neuroticism (N) were captured in late adolescence via cohort members' self‐completion of the NEO PI/FFI supplement for use with the NEO five‐factor inventory (Costa and McCrae 1992), which consisted of 15 items with possible responses to each statement on a Likert scale from 1 (“does not apply to me at all”) to 7 (“applies to me perfectly”). For example, the items “I see myself as someone who does a thorough job”, “I see myself as someone who tends to be lazy” (reverse coded), and “I see myself as someone who does things efficiently” load onto the factor of conscientiousness. Further information on which items were included in each case can be found in the supplemental online material (SOM 2024). Cronbach's alpha for each trait in the age 17 survey wave was $\alpha_{O}=0.67,\alpha_{C}=0.59,\alpha_{E}=0.66,\alpha_{A}=0.63,\alpha_{N}=0.79$. The five factors also load onto the meta‐traits of stability (combining conscientiousness, agreeableness, and negative neuroticism) and plasticity (openness and extraversion).

*Motor and Communicative Function (9 months)*: Gross and fine motor skills and communicative function (psychomotor functions) were assessed in the child's home at age nine months. Gross (4 items) and fine (4 items) motor coordination skills were measured with the Denver Developmental Screening Test (Frankenburg and Dodds 1967). Gross motor coordination includes the infant being able to sit up unsupported and stand up while holding onto something such as furniture. Fine motor coordination includes grabbing objects with the whole hand (palmar grasp) or picking up small objects using forefinger and thumb (pincer grasp). Communicative function (5 items) was measured with a UK‐adapted MacArthur Communicative Development Inventories (Fenson et al. 1993), which included communicative gestures such as the infant smiling back when being smiled at, reaching out and giving a toy, waving bye‐bye when someone leaves, or extending arms to express wanting to be picked up by a caregiver. Items had possible answers “not yet” (coded as 0), “sometimes” or “once or twice” (1), and “often” (2)—only one item from the gross motor skills (for walking a few steps unaided) had a no/yes response (recoded as 0/2). The numerical ranges in our sample were 0 to 8 for gross and fine motor skills and 0 to 10 for communicative function at age 9 months.

*Cognitive Ability (5 years)*: This was a latent variable in the structural equation model, which was composed of verbal and non‐verbal (spatial) ability. *Verbal ability*: Expressive language skills were measured with the British Ability Scales II (Hill 2005) Naming Vocabulary test in the age 5 wave. The test involved showing objects (in pictures used as visual cues) and asking the child to find a noun to label these objects. *Spatial ability (age 5)*: The Pattern Construction task from the British Ability Scales II was used; children were asked to recreate certain patterns as directed using a set of blocks that varied in shape, tone, and colors, which required rotation and rearrangement of the blocks to match the stimulus pattern. In both cases, age‐standardized T‐scores were used for these two variables, ranging from 20 to 80. These two tasks were administered by a trained interviewer in the child's home.

*Self‐Regulation (5 years)*: This also was a latent variable in the structural equation model, composed of emotion regulation and independence skills in the age 5 wave of the survey. *Emotion regulation* skills were evaluated with five items from the Adaptive Social Behavior Inventory (Hogan, Scott, and Bauer 1992) (Cronbach's α=0.65), where the primary caregiver rated on a Likert scale the extent of the child's display of mood swings, over‐excitement, frustration, inability to get over being upset, and impulsive behavior. *Independence* skills were evaluated with five items from the same inventory, indicating the extent to which the child likes to work things out by himself or herself, does not need much help with tasks, chooses activities on their own, and persists in the face of difficult tasks. The numerical range for both variables was recoded in 1 to 21, and higher values corresponded to better self‐regulation.

*Prosociality (5 years)*: The measurement of prosocial behaviors was made through the relevant subscale of the parent‐reported strengths and difficulties questionnaire (SDQ) (Goodman 1997), namely, a numerical variable from 0 to 10 (recoded as 1 to 11), given by the sum of 5 items (α=0.68) that admitted responses “not true”, “somewhat true”, and “certainly true”. The items were evaluating the extent to which the child was considerate of others' feelings; sharing with others; being helpful when someone is hurt, upset, or ill; being kind to younger children; and spontaneously volunteering to help others.

*Theory of Mind (5 years)*: At the very start of the cognitive test battery, which included the naming vocabulary and pattern construction tests, the interviewers gave children a vignette version of the Sally‐Anne task (Baron‐Cohen, Leslie, and Frith 1985) with eleven pointing‐and‐talking items in the narrative (pointing at figures on a suitable drawing) and three final questions that evaluated false belief understanding and checked recall and understanding. Details about this assessment in MCS have been provided elsewhere (Tsomokos and Flouri 2023), where it was argued that the particular assessment mode—as delivered in the MCS at age 5 and age 7 years—most likely measured superior social cognitive abilities and social competence (and not exclusively theory of mind, as such). In the present study, the variable for (superior social cognition and) theory of mind was dichotomous, and it corresponded to answering all three questions correctly on both occasions.

*Covariates*: These were all provided in the first wave (age 9 months) of the survey, concurrently with the exogenous (independent) variables. *Income* was the household income given in OECD‐equivalised quintiles. *Sex* was biological male or female at birth. A cohort member's exact *age* in months (ranging from 8 to 11 months in our sample) provided the correct age grouping at baseline (i.e., for the survey sweep that took place at around age 9 months). *Maternal mental health* captured the presence (1)/absence (0) of a diagnosis for depression or anxiety. *Small for gestational age* was a binary variable (yes/no, 1/0) corresponding to whether (or not) the neonate had significantly lower than average birth weight (or normal) for his or her gestational age and sex, using suitable cut‐offs (Talge et al. 2014). Since the calculation of this variable involves gestation time and birth weight, we also report on the clinically relevant variables of preterm birth (gestational age ≤ 36 weeks) and low birth weight (< 2.5 kg), which are used in sensitivity analyses and additional models but not included in the final model to avoid collinearity and collider bias (Hernández‐Díaz, Schisterman, and Hernán 2007). Finally, *area disadvantage*, which was also necessary for the survey design and accurate representation of the general youth population in the UK, was a categorical variable with an advantaged and a disadvantaged stratum for each country, where disadvantage corresponds to the case when an electoral ward was in the upper quartile (poorest 25%) of the child poverty index (in England there also was an ethnic minority stratum for wards with at least 30% of the population being “Black” or “Asian” as in the 1991 UK Census).

Other variables—also used in additional analyses but not the focal model of the main analysis presented below—included *ethnicity*, which was based on the UK Census simplified categorization (White, Mixed, Indian, Pakistani, and Bangladeshi, Black or Black British, Other Ethnic group including Chinese or Other); *maternal education* was the mother's highest educational level attained by the first survey wave, based on National Vocational Qualifications and its equivalents in the UK (numerical variable from 1 to 6); *maternal age* at the time of the child's birth (in years); *both parents in household* which was a binary variable (yes/no, 1/0) denoting whether both natural parents lived together in the household; *baby health problems* which was a parent‐reported score from 0 to 26 in our sample that tracked the number of health problems of the neonate and up to the age 9 month wave of the survey; and *siblings* (a numerical variable from 0 to 9) that provided the total number of other siblings in the household. More detailed information on all these variables (including their MCS labels, for ease of reference) is included in the Supplemental Online Material (SOM 2024).

### 2.3 Analytic Strategy

The study proceeded in three main steps: First, in a preliminary analysis, the sample bias, patterns of missingness, and correlations (for the numerical variables) were considered. At this stage, descriptive analyses were performed to investigate any differences between participants in the sample and those excluded from it and to ensure that missingness was low and that values were missing at random (and not missing completely at random). Pairwise correlations were calculated among the numerical variables as well. Second, survey design characteristics were used, and structural equation models were fitted before and after data imputation, both in a crude model and after controlling for a range of potential confounders. As this was an exploratory analysis of a secondary dataset, results were corrected using the Benjamini‐Hochberg (BH) adjusted p‐value procedure (Benjamini and Hochberg 1995). Missing data were imputed using multiple imputation by chained equations (Raghunathan et al. 2001), and m=25 imputed datasets were combined using Rubin's rules to produce coefficient estimates and standard errors (Rubin 1987). All calculations were carried out in R version 4.3.1 (R Core Team 2021) with the “mice” package for imputation (van Buuren and Groothuis‐Oudshoorn 2011) and “lavaan” for the structural equation models (Rosseel 2012). In the third step, (a) additional and (b) sensitivity analyses were performed to ensure robustness of the results.

#### 2.3.1 Main Analyses

**FIGURE 1:**
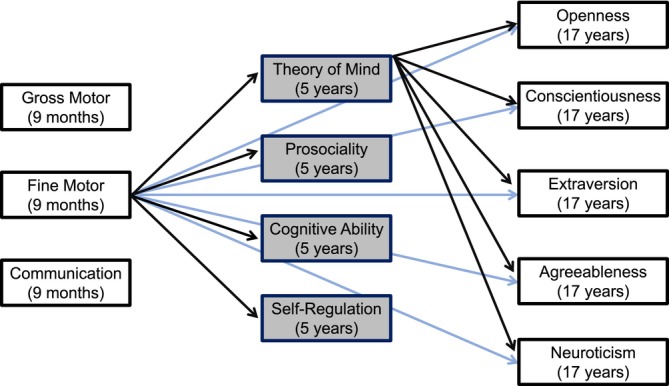
Simplified depiction of the structural equation model (only the paths for fine motor skills and theory of mind are shown here). Theory of mind, prosociality, cognitive and self‐regulation skills (shaded boxes) act as mediators between psychomotor functions at age 9 months and personality structure at 17 years.

Three survey‐weighted structural equation models (SEMs) were fitted, which differed on the level of adjustment with control variables, starting from the completely unadjusted model depicted in Figure 1. Therefore, Model 1 did not adjust for any control variables (unadjusted model); Model 2 controlled for sex, exact age at baseline (in months), income, and area disadvantage (survey stratum); whereas Model 3 additionally controlled for maternal mental health (diagnosis of depression or anxiety), and the participant being born small for gestational age. In the first analysis, the outcomes at age 17 years (exogenous variables in the SEMs) were the five personality factors. The models were fitted both with and without the hypothesized mediators at age 5 (theory of mind, prosociality, cognitive skills, and self‐regulation). In the second analysis, the primary outcomes (the five personality factors) were loaded onto the meta‐traits of stability (combining conscientiousness, agreeableness, and negative neuroticism) and plasticity (openness and extraversion), and the models were refitted with these meta‐traits as outcomes.

#### 2.3.2 Additional and Sensitivity Analyses

Additional models were fitted that controlled for other potential confounders, which had not been included in the focal models to keep them parsimonious and avoid introducing biases and collinearity effects. In these additional analyses, we added ethnicity, maternal education, maternal age (when the child was born), both natural parents living in the household, and number of other siblings, as these have also been known to affect cognitive development. Finally, the key aim of the sensitivity analysis was to investigate the impact of neonatal problems at birth as they have been associated with developmental delays in infancy (in the focal models, such problems are partially tracked by the variable ‘small for gestational age’). In the sensitivity analysis, therefore, cohort members who had been born prematurely or with low birthweight or had at least three health problems after birth were completely excluded from the model (and ‘small for gestational age’ was not required as a confounder).

## 3 Results

### 3.1 Sample Bias and Missing Data

In the present study, missingness due to attrition and non‐response was in line with typical MCS patterns (Connelly and Platt 2014; Plewis 2007); additionally, due to sample selection criteria, those excluded (N=9,552) from the final sample amounted to 51% of the singletons or first‐born twins/triplets in the first survey wave. 54% of those were male, had lower equivalized household income (Cohen's $d=-0.33,95\%\;\mathrm{CI}\left[-0.36,-0.30\right]$), and were over‐represented by families with one natural parent in the household (21% single‐parent families compared with 13% in the analytic sample, p<0.001). Full details for all variables are included in Table 1.

**TABLE 1:** Sample bias analysis: Unweighted variable distribution between the final analytic sample and the rest of the MCS at age 9 months (first wave of the survey).

Characteristic	Rest of MCS1 *N* = 9552	Analytic sample *N* = 9202	*p* ^a^
Sex, *n* (%)			**< 0.001**
Male	5173 (54)	4446 (48)	
Female	4379 (46)	4756 (52)	
Exact age (in months), *n* (%)			**< 0.001**
8	311 (3.3)	278 (3.0)	
9	7087 (74)	7161 (78)	
10	1874 (20)	1569 (17)	
11	280 (2.9)	194 (2.1)	
Area disadvantage (Stratum), *n* (%)			
England – Advantaged	2078 (22)	2604 (28)	
England – Disadvantaged	2399 (25)	2180 (24)	
England – Ethnic	1204 (13)	1197 (13)	
Wales – Advantaged	422 (4.4)	421 (4.6)	
Wales – Disadvantaged	1043 (11)	903 (9.8)	
Scotland – Advantaged	602 (6.3)	559 (6.1)	
Scotland – Disadvantaged	734 (7.7)	460 (5.0)	
Northern Ireland – Advantaged	361 (3.8)	370 (4.0)	
Northern Ireland – Disadvantaged	709 (7.4)	508 (5.5)	
Both parents in household, *n* (%)	7507 (79)	7963 (87)	**< 0.001**
(Missing)	23	7	
Siblings, Mean (SD)	0.97 (1.12)	0.90 (1.04)	**0.002**
Ethnicity, *n* (%)			**0.004**
White	7931 (83)	7549 (82)	
Mixed	299 (3.1)	262 (2.9)	
Indian	223 (2.3)	246 (2.7)	
Pakistani and Bangladeshi	580 (6.1)	683 (7.4)	
Black or Black British	355 (3.7)	323 (3.5)	
Other Ethnic group (inc Chinese)	137 (1.4)	127 (1.4)	
(Missing)	27	12	
Income, Mean (SD)	2.54 (1.36)	3.00 (1.42)	**< 0.001**
(Missing)	51	18	
Maternal education, Mean (SD)	3.12 (1.45)	3.60 (1.44)	**< 0.001**
(Missing)	314	265	
Maternal age (years), Mean (SD)	29 (6)	30 (6)	**< 0.001**
(Missing)	2	1	
Maternal mental health (depression/anxiety diagnosis), *n* (%)			**< 0.001**
0 (No)	7136 (75)	7078 (77)	
1 (Yes)	2412 (25)	2121 (23)	
(Missing)	4	3	
Small for gestational age, *n* (%)			**0.007**
0 (No)	8094 (86)	7956 (87)	
1 (Yes)	1306 (14)	1141 (13)	
(Missing)	152	105	
Communicative function, Mean (SD)	6.65 (1.74)	6.60 (1.69)	**0.007**
(Missing)	644	11	
Gross motor function, Mean (SD)	5.46 (1.43)	5.50 (1.33)	0.18
(Missing)	641	0	
Fine motor function, Mean (SD)	7.55 (0.91)	7.59 (0.83)	0.062
(Missing)	714	0	
Theory of mind (age 5), *n* (%)			0.070
0 (No)	5089 (85)	7282 (84)	
1 (Yes)	870 (15)	1356 (16)	
(Missing)	3593	564	
Prosociality (age 5), Mean (SD)	9.32 (1.72)	9.42 (1.65)	**0.004**
(Missing)	3631	747	
Verbal ability (age 5), Mean (SD)	52 (12)	54 (12)	**< 0.001**
(Missing)	3416	498	
Spatial ability (age 5), Mean (SD)	48 (12)	51 (10)	**< 0.001**
(Missing)	3427	516	
Independence (age 5), Mean (SD)	16.1 (3.9)	16.4 (3.7)	**< 0.001**
(Missing)	3613	740	
Emotion regulation (age 5), Mean (SD)	13.3 (4.8)	14.1 (4.7)	**< 0.001**
(Missing)	3614	739	
Openness, Mean (SD)	12.6 (7.5)	14.3 (3.8)	**0.003**
(Missing)	9026	0	
Conscientiousness, Mean (SD)	12.7 (7.4)	14.2 (3.3)	0.074
(Missing)	9035	0	
Extraversion, Mean (SD)	12.2 (6.9)	13.6 (3.9)	**0.013**
(Missing)	9043	0	
Agreeableness, Mean (SD)	15.4 (7.1)	16.6 (3.0)	0.12
(Missing)	9022	0	
Neuroticism, Mean (SD)	10.3 (7.0)	11.9 (4.8)	**< 0.001**
(Missing)	9030	0	

^a^ Pearson's Chi‐squared test; Wilcoxon rank sum test. Bold *p*‐value for *p* < 0.05.

In the final sample there were complete data on the dependent variables (five‐factor model) and motor skills (only 11 missing records for communicative function), as well as on the cohort member's sex, exact age at baseline, and number of siblings. There was minor missingness (up to 105 values, or 0.01% of the sample) for maternal age and maternal mental health, both parents in household, income, ethnicity, and small for gestational age. The largest amount of missingness (8.1%) occurred for prosociality at age 5 years, as can be seen in Table 1. A separate analysis, presented in full in the SOM in the preliminary analysis section (SOM 2024), confirmed that values were not missing completely at random (MCAR) using Little's MCAR test (Little 1988). Given the nature of this longitudinal survey and the patterns of missingness across survey waves (Plewis 2007), we may consider the present dataset to have information missing at random (MAR).

### 3.2 Correlations

Correlations for the key numerical covariates and mediators can be found in Table 2. The strongest positive correlation occurred between income and maternal education, $r\left(8923\right)=0.55,p<0.001$, which is why the focal models of the main analysis presented in the following subsection do not include both of these as control variables. For all remaining pairs of variables there were correlations with r<0.40, and full details are included in the preliminary analysis section of the SOM document (SOM 2024).

**TABLE 2:** Correlations between the key covariates (pairwise complete observations).

	(1)	(2)	(3)	(4)	(5)	(6)	(7)
Income (1)							
Maternal education (2)	**0.55**						
Verbal ability, 5 years (3)	**0.36**	**0.36**					
Spatial ability, 5 years (4)	**0.19**	**0.19**	**0.39**				
Emotion regulation, 5 years (5)	**0.24**	**0.21**	**0.18**	**0.15**			
Independence, 5 years (6)	**0.07**	**0.08**	**0.16**	**0.18**	**0.26**		
Prosociality, 5 years (7)	**0.07**	**0.06**	**0.11**	**0.09**	**0.29**	**0.37**	

*Note:* Pearson's correlation coefficients (bold for *p* < 0.05).

### 3.3 Survey‐Weighted, Imputed Models

Overall, the five factors at 17 years were indeed predicted by psychomotor functions at 9 months (fine motor skills, gross motor skills, and communicative skills), as well as by the mediators at 5 years (theory of mind, prosociality, cognitive ability, and self‐regulation). For instance, in the fully adjusted case (Model 3), after applying the BH correction to control the false discovery rate over the 122 longitudinal paths of interest, as detailed in the SOM (2024), trait openness in late adolescence was predicted by gross motor skills in infancy ($\begin{array}{c} b_{g\to O}=0.097,\mathrm{se}=0.036,z=2.685, \end{array}$ $\begin{array}{c} p=0.032,95\%\;\mathrm{CI}\;\left[0.026,0.167\right] \end{array}$, standardized $\beta_{g\to O}=0.034$), as well as cognitive ability ($\begin{array}{c} b_{\mathrm{cog}\to O}=0.051,\mathrm{se}=0.012,z=4.348, \end{array}$ $\begin{array}{c} p<0.001,95\%\;\mathrm{CI}\;\left[0.028,0.167\right] \end{array}$, standardized $\beta_{\mathrm{cog}\to O}=0.118$). Trait conscientiousness was significantly predicted by theory of mind (standardized $\beta_{t\to C}=0.035,p=0.011$) and self‐regulation skills (standardized $\beta_{t\to C}=0.191,p<0.001$), while the total effect of communicative function on this trait via all paths was (standardized) $\beta_{c\to C}=0.044,p=0.004$. The full results for all paths are presented in the SOM (2024), but the most interesting paths with significant associations (after a BH correction) are summarized in Table 3.

**FIGURE 2:**
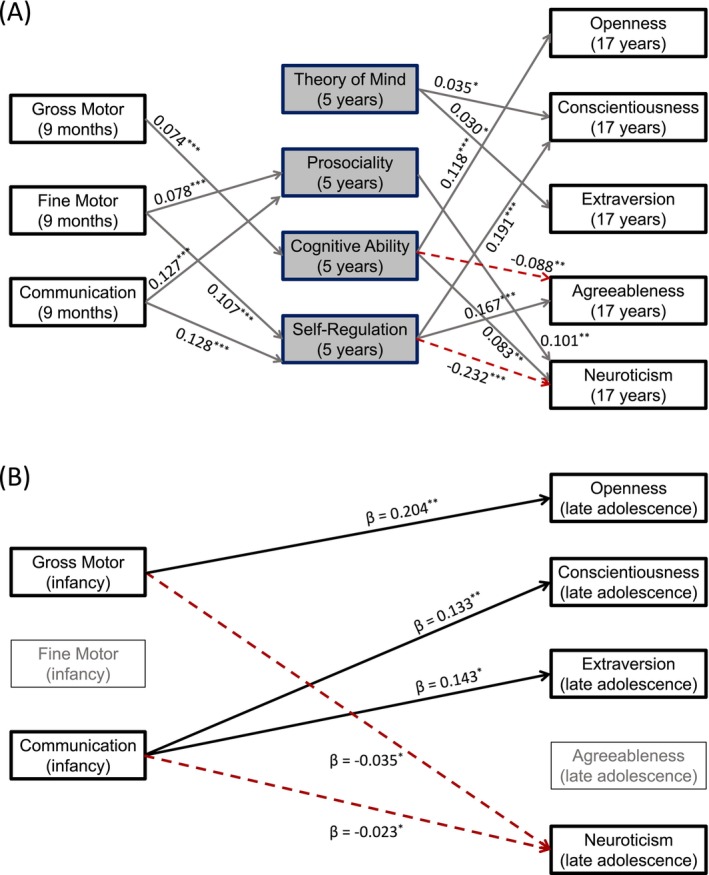
Results for the survey‐weighted, imputed, and fully adjusted model 3 (sample of N=9,202) after applying the Benjamini‐Hochberg procedure to control the false discovery rate over 122 possible paths. Plot A (top) shows significant paths (p<0.05, corrected *p*‐values) from infancy (age 9 months) to childhood (5 years) and from childhood to adolescence (17 years). Plot B (bottom) shows the total effects (direct and indirect) of infant psychomotor functions on the five factors of personality in late adolescence. Standardized estimates are shown in all cases (*p < 0.05, **p < 0.01, ***p < 0.001), and broken lines depict negative coefficients.

Furthermore, all four mediators at 5 years were predicted by psychomotor functions at 9 months. For instance, prosociality was predicted by fine motor skills ($\begin{array}{c} b_{f\to P}=0.151,\mathrm{se}=0.028,z=5.502, \end{array}$ $\begin{array}{c} p<0.001,95\%\;\mathrm{CI}\;\left[0.100,0.210\right] \end{array}$, $\beta_{f\to P}=0.078$) and communicative skills ($\begin{array}{c} b_{c\to P}=0.124,\;\mathrm{se}=0.013,\;z=9.833, \end{array}$ $\begin{array}{c} p<0.001,95\% \end{array}$ $\begin{array}{c} \mathrm{CI}\;\left[0.099,0.149\right] \end{array}$, standardized $\beta_{c\to P}=0.127$), but cognitive ability was predicted by gross motor skills ($\begin{array}{c} b_{g\to C}=0.488, \end{array}$ $\begin{array}{c} \mathrm{se}=0.108,z=4.497,p<0.001, \end{array}$ $\begin{array}{c} 95\%\;\mathrm{CI}\;\left[0.275,0.700\right] \end{array}$, $\beta_{g\to C}=0.074$) only. Again, it should be noted that these cases correspond to the fully adjusted model (3), after correcting with the BH process for 122 paths of interest. A simplified diagram of the significant paths is provided in Figure 2 and summarized in Table 4. Therefore, there is robust evidence for direct effects (Table 3), indirect mediation effects (Table 4), and total effects (Figure 2B) of infant psychomotor functions on adolescent personality structure.

**TABLE 3:** Survey‐weighted, imputed structural equation models (N=9,202) for no adjustment (Model 1), moderate adjustment with key covariates (Model 2), and full adjustment with all covariates (Model 3) [unstandardized estimates (std errors)].

	Model 1	Model 2	Model 3
*Regression slopes*			
Openness			
Fine motor	0.01 (0.06)	0.01 (0.06)	0.01 (0.06)
Gross motor	0.09 (0.04)*	0.10 (0.04)**	**0.10 (0.04)** **
Communicative	0.03 (0.03)	0.01 (0.03)	0.01 (0.03)
Theory of mind	−0.15 (0.13)	−0.17 (0.13)	−0.17 (0.13)
Prosociality	−0.07 (0.06)	−0.09 (0.06)	−0.09 (0.06)
Cognitive ability	0.05 (0.01)***	0.05 (0.01)***	**0.05 (0.01)** ***
Self‐regulation	0.13 (0.08)	0.15 (0.07)*	0.16 (0.07)*
Sex: Male		−0.08 (0.11)	−0.07 (0.11)
Age (in months)		−0.06 (0.10)	−0.07 (0.10)
Income		0.03 (0.04)	0.04 (0.04)
Mat. mental health			0.28 (0.11)*
Small for gest. age			0.06 (0.14)
Conscientiousness			
Fine motor	0.03 (0.05)	0.03 (0.05)	0.03 (0.05)
Gross motor	0.03 (0.03)	0.03 (0.03)	0.03 (0.03)
Communicative	0.03 (0.03)	0.04 (0.03)	0.04 (0.03)
Theory of mind	0.30 (0.10)**	0.32 (0.10)**	**0.32 (0.10)** **
Prosociality	−0.05 (0.06)	−0.06 (0.06)	−0.06 (0.06)
Cognitive ability	0.00 (0.01)	−0.00 (0.01)	−0.00 (0.01)
Self‐regulation	0.27 (0.06)***	0.27 (0.06)***	**0.26 (0.06)** ***
Sex: Male		0.13 (0.08)	0.13 (0.08)
Age (in months)		−0.06 (0.08)	−0.06 (0.08)
Income		0.02 (0.04)	0.02 (0.04)
Mat. mental health			−0.07 (0.10)
Small for gest. age			0.08 (0.11)
Extraversion			
Fine motor	0.03 (0.06)	0.01 (0.06)	0.01 (0.06)
Gross motor	0.03 (0.04)	0.05 (0.04)	0.04 (0.04)
Communicative	0.06 (0.03)*	0.07 (0.03)*	0.07 (0.03)*
Theory of mind	0.36 (0.13)**	0.33 (0.13)*	**0.33 (0.13)** *
Prosociality	0.03 (0.06)	0.06 (0.06)	0.07 (0.06)
Cognitive ability	−0.01 (0.01)	−0.01 (0.01)	−0.01 (0.01)
Self‐regulation	0.11 (0.08)	0.04 (0.07)	0.02 (0.07)
Sex: Male		−0.22 (0.11)*	−0.23 (0.11)*
Age (in months)		0.01 (0.09)	0.02 (0.09)
Income		0.18 (0.04)***	0.16 (0.04)***
Mat. mental health			−0.45 (0.12)***
Small for gest. age			−0.16 (0.15)
Agreeableness			
Fine motor	0.05 (0.05)	0.02 (0.05)	0.02 (0.05)
Gross motor	−0.05 (0.03)	−0.02 (0.03)	−0.02 (0.03)
Communicative	0.03 (0.03)	−0.00 (0.02)	−0.00 (0.02)
Theory of mind	0.18 (0.10)	0.11 (0.10)	0.11 (0.10)
Prosociality	−0.02 (0.05)	−0.01 (0.05)	−0.01 (0.05)
Cognitive ability	−0.04 (0.01)**	−0.03 (0.01)***	**−0.03 (0.01)** **
Self‐regulation	0.28 (0.06)***	0.21 (0.06)***	**0.22 (0.06)** ***
Sex: Male		−0.88 (0.08)***	−0.88 (0.08)***
Age (in months)		0.12 (0.07)	0.11 (0.07)
Income		0.09 (0.03)**	0.09 (0.03)**
Mat. mental health			−0.01 (0.09)
Small for gest. age			0.08 (0.11)
Neuroticism			
Fine motor	0.11 (0.07)	0.04 (0.07)	0.04 (0.07)
Gross motor	−0.22 (0.04)***	−0.10 (0.04)*	−0.10 (0.04)*
Communicative	0.10 (0.04)**	−0.05 (0.03)	−0.05 (0.03)
Theory of mind	0.16 (0.17)	−0.00 (0.16)	−0.00 (0.16)
Prosociality	0.31 (0.08)***	0.30 (0.07)***	**0.29 (0.08)** ***
Cognitive ability	0.08 (0.02)***	0.05 (0.01)***	**0.05 (0.01)** ***
Self‐regulation	−0.37 (0.10)***	−0.49 (0.09)***	**−0.47 (0.09)** ***
Sex: Male		−3.58 (0.11)***	−3.58 (0.11)***
Age (in months)		0.18 (0.12)	0.17 (0.12)
Income		0.08 (0.06)	0.10 (0.06)
Mat. mental health			0.48 (0.14)***
Small for gest. age			−0.09 (0.19)
*Factor loadings: Cognitive ability*			
Verbal ability	0.64	0.77	0.77
Spatial ability	0.58	0.48	0.49
*Factor loadings: Self‐regulation*			
Emotion regulation	0.47	0.49	0.50
Independence	0.55	0.52	0.52
*Fit indices*			
Scaled *χ* ^2^	169.12***	1191.75***	1241.86***
Robust CFI	0.98	0.91	0.91
Robust SRMR	0.01	0.02	0.02
Robust RMSEA	0.03	0.04	0.04

*Note:* Results in bold indicate that significance (*p* < 0.05) has been retained even after the Benjamini‐Hochberg procedure controlling the false discovery rate over 122 possible paths in Model 3. Area disadvantage (Stratum) covariates are omitted for brevity but can be found in Section 1 of the Supplemental Online Material (SOM 2024).

Abbreviations: CFI, comparative fit index; gest., gestational; Mat., Maternal; RMSEA, root mean square error of approximation; SRMR, standardized root mean square residual.

* *p* < 0.05.

** *p* < 0.01.

*** *p* < 0.001.

**TABLE 4:** Coefficient estimates [unstandardized (std errors)] for the mediators and significant partial mediation paths.

	Model 1	Model 2	Model 3
*Regression slopes*			
Theory of mind			
Fine motor	−0.01 (0.01)	−0.01 (0.01)	−0.01 (0.01)
Gross motor	−0.00 (0.00)	0.00 (0.00)	−0.00 (0.00)
Communicative	0.01 (0.00)*	0.01 (0.00)*	0.01 (0.00)*
Sex: Male		−0.04 (0.01)***	−0.04 (0.01)***
Age (in months)		−0.01 (0.01)	−0.00 (0.01)
Income		0.01 (0.00)***	0.01 (0.00)***
Mat. mental health			−0.00 (0.01)
Small for gest. age			−0.03 (0.01)*
Prosociality			
Fine motor	0.18 (0.03)***	0.16 (0.03)***	**0.16 (0.03)** ***
Gross motor	−0.01 (0.02)	0.01 (0.02)	0.01 (0.02)
Communicative	0.13 (0.01)***	0.12 (0.01)***	**0.12 (0.01)** ***
Sex: Male		−0.41 (0.04)***	−0.42 (0.04)***
Income		−0.11 (0.05)*	−0.11 (0.05)*
Mat. education		0.08 (0.02)***	0.08 (0.02)***
Small for gest. age			−0.05 (0.05)
Cognitive ability			
Fine motor	0.77 (0.17)***	0.40 (0.17)*	0.37 (0.17)*
Gross motor	0.47 (0.11)***	0.51 (0.11)***	**0.49 (0.11)** ***
Communicative	0.00 (0.09)	0.21 (0.08)*	0.20 (0.08)*
Sex: Male		−0.89 (0.26)***	−0.91 (0.26)***
Income		0.42 (0.31)	0.47 (0.31)
Mat. education		2.26 (0.15)***	2.20 (0.15)***
Small for gest. age			−0.27 (0.32)
Self regulation			
Fine motor	0.37 (0.05)***	0.31 (0.05)***	**0.30 (0.05)** ***
Gross motor	0.06 (0.03)	0.09 (0.03)**	0.08 (0.03)*
Communicative	0.18 (0.03)***	0.18 (0.03)***	**0.18 (0.03)** ***
Sex: Male		−0.84 (0.08)***	−0.85 (0.08)***
Income		−0.10 (0.10)	−0.08 (0.10)
Mat. education		0.42 (0.03)***	0.40 (0.04)***
Small for gest. age			−0.53 (0.12)***
Indirect effects (Model 3, *p* < 0.05 only)			
Gross motor → Cognitive ability → Openness			**0.03 (0.01)** **
Fine motor → Self‐regulation → Conscientiousness			**0.08 (0.02)** **
Gross motor → Self‐regulation → Conscientiousness			0.02 (0.01)*
Communicative → Self‐regulation → Conscientiousness			**0.05 (0.01)** ***
Gross motor → Cognitive ability → Agreeableness			**−0.02 (0.01)** **
Fine motor → Self‐regulation → Agreeableness			**0.07 (0.02)** **
Gross motor → Self‐regulation → Agreeableness			0.02 (0.01)*
Communicative → Self‐regulation → Agreeableness			**0.04 (0.01)** ***
Fine motor → Prosociality → Neuroticism			**0.05 (0.01)** **
Communicative → Prosociality → Neuroticism			**0.04 (0.01)** ***
Fine motor → Self‐regulation → Neuroticism			**−0.14 (0.04)** ***
Gross motor → Self‐regulation → Neuroticism			−0.04 (0.02)*
Communicative → Self‐regulation → Neuroticism			**−0.08 (0.02)** ***

*Note:* Results for the covariate of area disadvantage are omitted for brevity but can be found in the SOM (2024). Indirect effects are only shown for Model 3 when *p* < 0.05 (bold indicates that *p* < 0.05 after the Benjamini‐Hochberg procedure has been applied in Model 3; the correction only applies to fine, gross, and communicative function variables and the respective total and indirect paths). Fit indices are the same as in Table 3.

* *p* < 0.05.

** *p* < 0.01.

*** *p* < 0.001.

### 3.4 Stability and Plasticity

The two higher‐order factors of stability and plasticity at age 17 years were predicted by psychomotor functions at age 9 months. In particular, even in the fully adjusted case (core Model 3 without the mediating variables) and after applying BH corrections, stability in late adolescence was predicted by communicative function ($\begin{array}{c} b_{c\to \mathrm{St}}=0.080,\mathrm{se}=0.023,z=3.475,p=0.003,95\%\;\mathrm{CI}\;\left[0.035,0.126\right] \end{array}$, standardized $\beta_{c\to \mathrm{St}}=0.063$). On the other hand, plasticity in late adolescence was predicted by infant gross motor function ($\begin{array}{c} b_{g\to \mathrm{Pl}}=0.075,\mathrm{se}=0.026,z=2.873,p=0.009,95\%\;\mathrm{CI}\;\left[0.023,0.127\right] \end{array}$, standardized $\beta_{g\to \mathrm{Pl}}=0.075$) as well as communicative function ($\begin{array}{c} b_{c\to \mathrm{Pl}}=0.058,\mathrm{se}=0.020,z=2.882,p=0.009,95\%\;\mathrm{CI}\;\left[0.019,0.098\right] \end{array}$, standardized $\beta_{c\to \mathrm{Pl}}=0.074$). Table 5 includes the results for Models 1–3 in this case. Note that, in this case, two of the three model fit indices were within the generally acceptable range, but the third index (CFI) was not (Shi, Lee, and Maydeu‐Olivares 2019; Ximénez et al. 2022), and the latent variables of stability and plasticity had standardized factor loadings from 0.32 to 0.66. This is not necessarily problematic (Kline 2023), as these two constructs have been confirmed in previous studies (DeYoung 2015). For details on the factor loadings and higher numerical accuracy of p‐values before and after BH corrections, consult Section 2 of the Supplemental Online Material (SOM 2024).

**TABLE 5:** Results for the meta‐traits of Stability and Plasticity: Survey‐weighted, imputed structural equation models (N=9,202) for the core Models 1, 2, and 3 without mediators [unstandardized estimates (std errors)].

	Model 1	Model 2	Model 3
*Regression slopes*			
Stability			
Fine motor	0.11 (0.04)*	0.10 (0.05)*	0.09 (0.05)*
Gross motor	0.04 (0.03)	0.04 (0.03)	0.04 (0.03)
Communicative	0.07 (0.02)**	0.08 (0.02)***	**0.08 (0.02)** ***
Sex: Male		0.05 (0.08)	0.06 (0.07)
Age (in months)		−0.06 (0.07)	−0.05 (0.07)
Income		0.13 (0.03)***	0.11 (0.03)***
Mat. mental health			−0.28 (0.09)**
Small for gest. age			−0.03 (0.10)
Plasticity			
Fine motor	0.07 (0.04)	0.04 (0.04)	0.04 (0.04)
Gross motor	0.07 (0.03)*	0.08 (0.03)**	**0.07 (0.03)** **
Communicative	0.06 (0.02)**	0.06 (0.02)**	**0.06 (0.02)** **
Sex: Male		−0.22 (0.07)**	−0.22 (0.07)**
Age (in months)		−0.02 (0.06)	−0.02 (0.06)
Income		0.17 (0.02)***	0.16 (0.03)***
Mat. mental health			−0.15 (0.08)
Small for gest. age			−0.12 (0.09)
*Fit indices*			
Scaled *χ* ^2^	707.34 (13)***	3340.53 (79)***	3312.51 (91)***
Robust CFI	0.82	0.55	0.54
Robust SRMR	0.04	0.04	0.04
Robust RMSEA	0.09	0.07	0.07

*Note:* Results in bold indicate that significance (*p* < 0.05) has been retained even after the Benjamini‐Hochberg procedure controlling the false discovery rate in Model 3 (the correction only applies to fine, gross, and communicative function variables). Results for the covariate of area disadvantage are omitted for brevity but are available in the SOM (2024).

Abbreviations: CFI, comparative fit index; gest., gestational; Mat., Maternal; RMSEA, root mean square error of approximation; SRMR, standardized root mean square residual.

* *p* < 0.05.

** *p* < 0.01.

*** *p* < 0.001.

### 3.5 Additional and Sensitivity Analyses

The findings presented were robust to controlling for additional confounders. In particular, in the fully adjusted case (Model 3) with the five personality factors as outcomes, additionally controlling for maternal education and maternal age at baseline, as well as family structure at baseline (both natural parents in the household and number of siblings of the cohort member), did not alter the main results over the 122 paths of interest. The same held true for the fully adjusted model for stability and plasticity. Full details for these results are provided in Appendices A (five‐factor model outcomes) and B (stability and plasticity outcomes) in the Supplemental Online Material (SOM 2024).

Finally, in the sensitivity analysis, the main models (1–3 for both types of outcomes) were refitted in a complete‐records analysis after excluding 2634 cohort members from the initial analytic sample, namely, those who were (a) born preterm (566 participants), or (b) had low birthweight (646 participants), or (c) had more than two health problems postnatally (1882 participants). Therefore, a final analytic sample of N=6,568 participants was retained for this sensitivity analysis. The full output can be found in the Sensitivity Analysis sections S1 and S2 of the SOM (2024). As can be seen there, the results on most of the longitudinal paths of interest in the fully adjusted case (Model 3 after BH corrections) are robust, and it is evident that our findings on the long‐term impact of infant psychomotor functions on both childhood cognitive abilities and adolescent personality remain intact. The only notable difference occurs in the model for the meta‐traits, for which the association between gross motor function in infancy and stability in late adolescence becomes non‐significant (while communicative function still predicts both meta‐traits).

## 4 Discussion

The findings of this study have provided evidence that infant psychomotor functions are prospectively associated with general and social cognitive abilities in childhood, as well as personality later in life. In a unified structural equation model fitted to large‐scale longitudinal survey data from a sample of 9202 youth, it was shown that motor and communicative skills at 9 months were associated with verbal and non‐verbal cognitive abilities, executive function, and social skills (theory of mind and prosocial behavior) at 5 years, in turn associated with the five personality factors of openness, conscientiousness, extraversion, agreeableness, and neuroticism at 17 years. A mediation analysis illuminated the indirect pathways through which early psychomotor functions contribute to personality development directly or via cognitive ability, self‐regulation, and social skills in childhood.

Trait openness in late adolescence was predicted by gross motor function and communicative skills in infancy, with cognitive ability (verbal and spatial skills) providing the main pathway supporting this trait. The association between cognitive skills and openness is in line with previous studies (DeYoung et al. 2014), as is the association between infant gross motor function and cognitive abilities (Veldman et al. 2019)—a link that has recently been investigated from the perspective of neural correlates as well, where it was shown that there are shared learning mechanisms between motor and cognitive systems (Constantinidis et al. 2023). The partial mediation between gross motor function and trait openness through cognitive ability (but not social skills) is a new finding, to the best of this author's knowledge.

Trait conscientiousness was predicted by the total effect of communicative function, with mentalizing (theory of mind) and self‐regulation skills (independence and emotion regulation) also being prospectively associated with it. The link between self‐regulation and conscientiousness is in line with previous findings; for instance, *cf*. Eisenberg et al. (2014) and Inzlicht et al. (2020). The main indirect pathways into trait conscientiousness were from fine motor and communicative skills through self‐regulation. Therefore, in this study, further evidence has been provided for an association between social cognitive and self‐regulation skills in childhood (but not verbal/spatial ability, relating to general intelligence) and trait conscientiousness in adolescence (Baumeister 2020; McCrae and Löckenhoff 2010).

Trait extraversion was predicted by early communicative function, while social cognitive ability (theory of mind) also supported this trait directly. Trait neuroticism, on the other hand, was predicted by both gross motor and communicative functions in a direct (negative) association, and indirectly through prosociality (positively) and by self‐regulation (negatively).

Trait agreeableness was predicted positively by self‐regulation and negatively by cognitive ability. However, it was not predicted by prosociality in childhood. This might seem counterintuitive, given the prosocial aspects of agreeableness and that several studies have identified an association between the two constructs (Cavallini et al. 2021; Graziano and Eisenberg 1997; Thielmann, Spadaro, and Balliet 2020). However, it should be noted that previous studies had examined the association between prosociality and agreeableness either cross‐sectionally or in older age groups. In a study that followed 965 Norwegian children from age 1.5 to 16.5 years, Baardstu, Karevold, and von Soest (2017) showed that agreeableness in adolescence was instead predicted by difficultness at age 4 and poor self‐regulation at age 12. Further, by 7 years, prosociality is preceded by earlier self‐regulation skills, as shown in another longitudinal study from Australia (Williams and Berthelsen 2017). Therefore, it is possible that—in the unified model used here—the presence of both prosociality and self‐regulation skills leads to the former construct becoming a non‐significant predictor of agreeableness. However, this is a hypothesis that warrants further investigation.

The higher‐order meta‐traits of stability and plasticity were also predicted by early psychomotor function. In particular, stability in late adolescence was predicted by infant communicative function. Plasticity, on the other hand, was predicted by both gross motor and communicative function. These findings tentatively suggest that the two meta‐traits may have distinct developmental roots linked to differences in early psychomotor function, particularly gross motor control. While this aligns with perspectives from embodied cognition, further research is needed to examine this association, especially given the modest fit of the model for the meta‐traits in the present study. Nonetheless, certain interesting links can be drawn with more recent developments in the field of embodied cognition.

Cybernetic Big 5 Theory (CB5T) is an integrative framework that employs cybernetics—the study of goal‐directed, adaptive systems—to investigate the structure of personality as a manifestation of the need for behavior regulation and adaptive functioning in the pursuit of goals under continuous two‐way interactions with the environment (DeYoung 2015). At the same time, the Free Energy Principle and Active Inference (FEP‐AI) is another unifying framework based on cybernetics and the physics of dynamical systems, which posits that a biological organism's homeostasis can be achieved through a balance of active goal‐oriented exploration of the environment and continuous updates of internal probabilistic models, so that prediction errors between expected and actual outcomes are minimized (Clark 2013; Friston 2010; Pezzulo, Rigoli, and Friston 2018). In a recent synthesis of the two frameworks, Safron and DeYoung (2021) pointed out that the higher‐order factors of stability and plasticity have a central, overarching role in the cybernetic approach to personality. The dimension of *stability*—i.e., the shared variance between conscientiousness, agreeableness, and negative neuroticism—captures an individual's ability to avoid distractions in the process of pursuing a goal, interpreting information, or devising strategies. whereas *plasticity*—the shared variance between openness and extraversion—captures the ability to explore the environment, gather and interpret information in new ways, generate new goals and strategies (Safron and DeYoung 2021). The present study has provided first evidence of the fundamentally embodied nature of these personality meta‐traits, showing that gross motor skills support the creation and exploration of new goals and strategies, or new ways of modeling the environment (plasticity), whereas communicative skills support both plasticity and the ability to pursue goals or strategies methodically while avoiding distractions (stability).

Relevant to this finding, a sensitivity analysis showed that, when excluding participants born prematurely, with low birthweight, or those who had health comorbidities—i.e., key risk factors for psychomotor delays in infancy (de Kieviet et al. 2009; Golding et al. 2014; Matson, Matson, and Beighley 2011)—the association between gross motor function and plasticity became non‐significant after full adjustment. This association, therefore, is sensitive to birth outcomes and perinatal health problems (whereas more generally the sensitivity analysis did not alter the other main findings). In the context of the CB5T and FEP‐AI, the results of this analysis point to the deep relations between initial conditions set by biological variations or constraints (e.g., due to birth outcomes), early sensorimotor function, which mediates the interactions between the organism and its environment, and later personality structure in terms of creativity and exploration that involves new goals or strategies that the organism can follow.

However, the limitations of these findings should also be considered in detail. First, while the longitudinal design of the MCS provides a robust setting for the examination of developmental pathways from infancy to adolescence, the observational nature of this dataset precludes strict causal inferences. Second, the dataset's focus on the youth population in the United Kingdom may restrict the generalizability to other cultural contexts, where a different emphasis may be placed on certain aspects of cognition and personality traits (Church 2016; Costa Jr, Terracciano, and McCrae 2001; Kärtner, Schuhmacher, and Torréns 2020; Wang 2018). Third, the constructs used in the present study were necessarily limited by what was available in MCS—e.g., the five factors of personality were captured via 15 items (three items per factor), while a longer form of the questionnaire may have been more informative in each case. It should be noted, however, that Donnellan et al. (2006) have provided evidence of the psychometric acceptability of a short form (20‐item) questionnaire of the Big Five (with comparable Cronbach's alpha values to the ones reported here). Similarly, infant psychomotor functions were based on short forms of home visit assessments that, ideally, would have been performed by clinicians. Fourth, and perhaps most importantly, motor and communicative functions were only assessed once at 9 months, and there was no information available at later time points so as to track their development during early childhood. The link between psychomotor skills in infancy and personality in late adolescence may also depend on the relative gains in these skills over time, but it was not possible to address this possibility here.

Arguably, a crucial limitation—or perhaps fundamental objection—to the findings presented here concerns the theoretical lens through which they have been interpreted, given the variables and observations available in the study. In this respect, it must be noted that the associations between childhood cognition and adolescent personality do not by themselves establish a causal relation between embodied cognition and personality, if anything, because embodied cognition is more than the sum of the cognitive abilities measured in the available MCS data. Therefore, in future work, it would be necessary to manipulate cognitive functions experimentally in terms of their embodiment and explore how these predict later personality traits. This could be done in suitably designed longitudinal studies by, for instance, including an experimental setup to measure individual differences between various aspects of embodied cognition in childhood or early adolescence (Ale, Sturdee, and Rubegni 2022; Hirai, Muramatsu, and Nakamura 2020; Keehner and Fischer 2012; Muraki and Pexman 2024) and subsequently tracking personality development from late adolescence to early adulthood. We should also note, however, that the cognitive skills that were available to us for this study indeed were robustly predicted by early motor and communicative functions during infancy, which does lend support to the central claim of embodied cognition, namely, that higher‐order cognitive functions develop along with and emerge out of lower‐order sensorimotor functions.

## 5 Conclusions

The present study made use of a large, nationally representative birth cohort from the UK to extend our understanding of the complex pathways through which early sensorimotor and communicative functions influence childhood cognition and later personality structure. Future work should explore further the mechanisms underlying these developmental pathways, including the role of environmental, genetic, and epigenetic factors. Additional translational research could review targeted interventions in infancy and early childhood that support psychomotor skills as a means of fostering the development of cognitive abilities throughout childhood and adaptive personality traits later in life. In summary, the findings of this study suggest that children's verbal and spatial skills, theory of mind, prosocial behavior, and self‐regulation are dependent on and constrained by motor and communicative functions in infancy, and that—crucially—these social, cognitive, and self‐regulation skills mediate the association between early psychomotor development and later personality structure.

## Author Contributions

**Dimitris I. Tsomokos**: conceptualization; data curation; formal analysis; methodology; project administration; resources; software; validation; visualization; writing.

## Conflicts of Interest

The author declares no conflicts of interest.

## Open Science Framework

Supplemental Online Material (https://osf.io/9tsmk/).

## Supporting information


**Data S1.** Supporting Information.

## Data Availability

The data that support the findings of this research are publicly available under license from the UK Data Service. The secondary dataset used here had been fully anonymised and no additional ethics approvals were required. This was not a preregistered study.
