# Supplementary material for: Embodied Cognition and the Structure of Personality: An Exploratory Study of Longitudinal Pathways From Early Psychomotor Function
Source: J Pers. 2025 Feb 1;93(6):1332–50. doi: 10.1111/jopy.13011 (PMC12592596; doi:10.1111/jopy.13011)
Supplement: Supplementary file 1 — Data S1. Supporting Information. [file JOPY-93-1332-s001.html]

SOM: Supplemental Online Material (OSF)


# SOM: Supplemental Online Material (OSF)

### Motor and communicative functions & personality structure

#### based on the Millennium Cohort Study (waves MCS1-MCS7)

#### 11 September 2024

# Research Question & Hypothesis

## Context

In this SOM document we provide additional details for
reproducibility and expand on certain points of the main manuscript. We
also provide details on the Millennium
Cohort Study variables, assumption checks, data plots, various
results before / after imputation, and standardised / unstandardised
coefficients. This is an output (knit HTML) file from the R code used
for the data analysis; OS details and version of R have as follows:

```
##                _                           
## platform       aarch64-apple-darwin20      
## arch           aarch64                     
## os             darwin20                    
## system         aarch64, darwin20           
## status                                     
## major          4                           
## minor          4.1                         
## year           2024                        
## month          06                          
## day            14                          
## svn rev        86737                       
## language       R                           
## version.string R version 4.4.1 (2024-06-14)
## nickname       Race for Your Life
```

  
   


---

## Research questions

**Primary**: Is the structure of
personality partially embodied in early motor and communicative
function?

**Secondary**: Are social cognitive and
self-regulation skills embodied in early motor and communicative
function, and do they mediate the relationship with personality?

These questions have never been addressed together in a large,
general youth population study. Using data from the MCS, we can adjust
for a variety of potential confounders that may be associated with both
the exposure and outcome variables.

  
   


---

## Hypothesis

Hypothesis

- **Motor and communicative function**
in infancy (Gross/fine motor skills and communication) **predicts
the structure of personality** in late adolescence (Big 5 at age
17 years), and the relationship is **mediated by general/social
cognition and self-regulation** in childhood (age 5 years).

  
   


---

## Main variables

For more information on the below (from which most of the text has
been copied), see: https://cls.ucl.ac.uk/wp-content/uploads/2018/08/Guide-to-Psychological-Inventories-in-MCS3.pdf

**Communicative Function**:
Communicative Development Inventories in infancy (age 9 months)

5 items from an UK adaptation of the MacArthur Communicative
Development Inventories (CDI) were used to identify early communicative
gestures. The CDI is a checklist of words and gestures assessing the
child’s development of receptive and productive vocabulary through
parental report (Fenson, L., Dale, P.S., Resnick, J.S, Thal, D., Bates,
E., Hartung, J.P., Pethick, D. and Reilly J.S ( 1993) MacArthur
Communicative Inventories, San Diego CA: Singular Publishing Group. The
following five variables were used from MCS1:

- SMIL He smiles when you smile at him.
- GIVE He reaches out and gives you a toy or some other object that he
  is holding.
- WAVE He waves bye-bye on his own when someone leaves.
- ARMS He extends his arms to show he wants to be picked up.
- NODS He nods his head for ‘yes’.

**Gross and Fine Motor Function**:
Denver Developmental Screening Test in infancy (age 9 months)

A selection of 8 questions taken from the Denver Developmental
Screening Test (DDST) were used to assess fine (DDST(F)) and gross
(DDST(G)) motor coordination typical for a 9 months old child (see
Frankenburg, W.K., Dodds, J.B. Denver (1967). Developmental Screening
Test. J. Paediatrics, 71, 181-191). The following eight variables were
used from MCS1:

- ACHAND00 FINE MOTOR: CM puts hands together.
- ACGRAB00 FINE MOTOR: CM grabs objects with whole hand.
- ACPICK00 FINE MOTOR: CM can pick up a small object using forefinger
  and thumb only.
- ACPTOY00 FINE MOTOR: CM passes a toy back and forth from one hand to
  another.
- ACSITU00 GROSS MOTOR: CM can sit up without being supported.
- ACSTAN00 GROSS MOTOR: CM can stand up while holding onto something
  such as furniture.
- ACWALK00 GROSS MOTOR: CM can walk a few steps on his/her own.
- ACMOVE00 GROSS MOTOR: If you put child down on the floor, can s/he
  move about from one place to another?

**Big 5 Personality Structure** in late
adolescence (age 17 years)

From the Young Person Self-Completion Questionnaire (personality,
health, and well-being module): https://cls.ucl.ac.uk/wp-content/uploads/2020/01/MCS7-Young-Person-Self-Completion-Questionnaire.pdf
and the data note: https://cls.ucl.ac.uk/wp-content/uploads/2022/05/MCS7-user-guide-Age-17-ed2.pdf

Big Five personality traits, also known as the five factor mode.
Fifteen questions on common language descriptors of personality. NEO
PI/FFI manual supplement for use with the NEO Personality Inventory and
the NEO Five-Factor Inventory Paul T. Costa, Jr. & Robert R. McCrea.
Published 1989 by Psychological Assessment Resources in Odessa, Fla.
(P.O. Box 998, Odessa 33556) (available online from http://www.openlibrary.org) .

The following questions are about how you see yourself as a person.
Choose the number which best describes how you see yourself (from 1-Does
not apply to me at all, 2, 3, 4, 5, 6, to 7-Applies to me
perfectly).

BIGA I see myself as someone who is sometimes rude to others BIGB I
see myself as someone who does a thorough job BIGC I see myself as
someone who is talkative BIGD I see myself as someone who worries a lot
BIGE I see myself as someone who is original, comes up with new ideas
BIGF I see myself as someone who has a forgiving nature BIGG I see
myself as someone who tends to be lazy BIGH I see myself as someone who
is outgoing, sociable BIGI I see myself as someone who gets nervous
easily BIGJ I see myself as someone who values artistic, aesthetic
experiences BIGK I see myself as someone who is considerate and kind to
almost everyone BIGL I see myself as someone who does things efficiently
BIGM I see myself as someone who is reserved BIGN I see myself as
someone who is relaxed, handles stress well BIGO I see myself as someone
who has an active imagination

Subgroups of 3 items load onto the 5 factors of personality (OCEAN),
each factor having a minimum score of 3 and a maximum score of 21.

  
   


---

## Mediators

**Prosociality (age 5 years)**

- Parent-reported SDQ-Prosocial Scale
- 5 items with possible scores Note true (0), Somewhat true (1), or
  Certainly true (2).
- The total Prosocial score (sum of these items) ranges from 0 to 10
  (recoded as 1 to 11).
- Items: **CPSDPF00** - Considerate of other people’s
  feelings; **CPSDSR00** - Shares readily with other children
  (treats, toys, pencils etc.); **CPSDHU00** - Helpful if
  someone is hurt, upset or feeling ill; **CPSDKY00** - Kind
  to younger children; **CPSDVH00** - Often volunteers to
  help others.

[ REFERENCE: Goodman, R. (1997). The Strengths and Difficulties
Questionnaire: a research note. J. Child Psychol. Psychiatry 38,
581–586. doi: 10.1111/j.1469-7610.1997.tb01545.x ]

**Theory of Mind (age 5 years)**

Trained interviewers administered a vignette version of the Sally-Ann
Task (Baron-Cohen et al., 1985) to children aged 5 years (repeated at
age 7), and this was the first task in a cognitive test battery. It
consisted of 11 pointing-and-talking interactions and 3 final questions
that assessed false belief understanding and allowed for a ‘memory’ and
‘reality’ comprehension check. The test was repeated in the next survey
sweep (when the children were aged 7 years). As explained in recent
work, the particular ToM measure in the MCS (Tsomokos & Flouri,
2023b; Tsomokos & Flouri, 2023a) identified a group of children who
passed the Sally-Ann Test under specific conditions and demonstrated (1)
false belief understanding, and (2) above-average social competence in a
demanding social situation.

Baron-Cohen, S., Leslie, A. M., & Frith, U. (1985). Does the
autistic child have a “theory of mind” ? Cognition, 21(1), 37-46. https://doi.org/https://doi.org/10.1016/0010-0277(85)90022-8

Tsomokos, D. I., & Flouri, E. (2023b). The role of social
cognition in mental health trajectories from childhood to adolescence.
European Child & Adolescent Psychiatry. https://doi.org/10.1007/s00787-023-02187-8

Tsomokos, D. I., & Flouri, E. (2023a). Superior social cognitive
abilities in childhood are associated with better reward-seeking
strategies in adolescence: evidence for a Social-Motivational
Flexibility Model. advances.in/psychology, 01, 1-19. https://doi.org/https://doi.org/10.56296/aip00002

**Cognitive Ability (age 5): Verbal &
Spatial ability**

- Latent variable in our models, made up of Verbal and Spatial
  ability as below.
- MCS variable (CDBAST00) - BAS Naming Vocabulary (T-scores) –
  Numerical variable from 20 to 80
- Age 5 years (variable CCPCTSCORE): Age-based Pattern Construction
  T-score – Derived total score variable, scaled and age-standardised,
  from the British Ability Scale cognitive assessment at the child’s home
  at age 5. – Numerical variable from 20 to 80

**Self-regulation (age 5): Emotion regulation
and independence skills**

- Latent variable in our models, made up of Verbal and Spatial
  ability as below.
- CDCSBI00 Child Social Behaviour Questionnaire (Independence-Self
  Regulation)
- CDCSBE00 Child Social Behaviour Questionnaire
  (Emotional-Dysregulation)
- Variables derived in MCS (e.g. CDCSBE00 at age 5, recoded in the
  range from 1 to 21, with higher values corresponding to more emotion
  regulation (less dysregulation) based on the main adult respondent’s
  responses to the five items as below)

– CPSEMS00: Shows mood swings – CPSEOE00: Gets over excited –
CPSEEF00: Easily frustrated – CPSEUQ00: Gets over being upset quickly –
CPSEIA00: Acts impulsively

[ In each case, the possible scores are Not true (1), Somewhat true
(2), Certainly true (3), or Can’t say (4) ]

[ MCS Resource: https://cls.ucl.ac.uk/wp-content/uploads/2018/08/Guide-to-Psychological-Inventories-in-MCS3.pdf

Variables come from: Child Self Regulation - Child Social Behaviour
Questionnaire

Sammons, P., Sylva, K., Melhuish, E., Siraj-Blatchford, I., Taggart,
B, Elliott, K., & Marsh, A. (2004). The Effective Provision of
Pre-school Education (EPPE) Project: Technical Paper 11: The continuing
effect of pre-school education at age 7 years. London: Institute of
Education.

Hogan AC, Scott KG, Bauer CR. The adaptive social behaviour inventory
(ASBI): A new assessment of social competence in high-risk three year
olds. Journal of Psychoeducational Assessment. 1992;10:230–239. ]

  
   


---

## Control Variables

### Survey design

**Area disadvantage (stratum)**

- MCS variable (PTTYPE2) for country / advantaged-disadvantaged (and
  Ethnic in England).
- When implementing a survey design we use the UK-wide weights for MCS
  sweep 1 (AOVWT2)

**MCS survey sweeps**: We point out that two different
notations are used in this document to denote survey sweeps in MCS. As
is common in the literature, we use numbers as per MCS1, MCS2, etc.
corresponding to the 1st, 2nd, …, sweep (in turn corresponding to age 9
months, 3 years, and so on). However, we also use the letters A, B, C, …
This is because the variable names in MCS begin with such letters that
correspond to the sweep; for instance, the stratum variable here begins
with “A” as it corresponds to the first sweep.

  
   


---

### CM characteristics

**Sex**

- MCS variable for sex at birth: Male/Female (as determined by the
  study design and the Centre for Longitudinal Studies)

**Ethnicity**

- MCS variable, 6 values (as determined by the study design and the
  Centre for Longitudinal Studies, based on the UK’s Census data available
  in 2000)

**CM Age (in months)**

- MCS variable (AHCAGE00), numerical value of cohort member’s exact
  age in days (recalculated in months) corresponding to participant’s age
  on the day of the interview at the age 9 month sweep (when the
  psychomotor development assessment was performed).

**Preterm Birth**

- MCS variable (ADGEST00) Cohort Member Gestation Time in days
- Numerical variable derived by MCS team as explained (p. 91) in https://cls.ucl.ac.uk/wp-content/uploads/2017/07/MCS1-Derived-Variables-June-2006.pdf
- From this, we derive “preterm birth” measure as Gestation Time <=
  36 weeks
- Binary variable: 1 for preterm birth baby, and 0 for > 36 weeks
  gestational age.

**Low Birth Weight**

- MCS variable (ADBWGT00) Cohort Member birth weight in kilos
- Numerical variable derived by MCS team as explained (p. 87) in https://cls.ucl.ac.uk/wp-content/uploads/2017/07/MCS1-Derived-Variables-June-2006.pdf
- From this, we derive “low birth weight”, which is defined as <
  2.5 kg
- Binary variable: 1 for LBW baby, and 0 for >= 2.5kg of weight at
  birth.

**Small for Gestational Age**

- Derived from the previous two MCS variables (Gestation Time and
  Birth Weight) and adjusting for sex, based on
- The cutoffs are from: Talge NM, Mudd LM, Sikorskii A, Basso O.
  Pediatrics. 2014 May;133(5):844-53). See R package: https://rdrr.io/github/dreidpath/SGA-LGA/
- Binary variable: 1 for SGA baby, and 0 for normal (or big) for
  gestational age.
- (not used in final models, for reference only)

**Baby health problems**

- MCS variable ACADMO00 = Number of health problems
- cf. https://cls.ucl.ac.uk/wp-content/uploads/2017/07/MCS1\_CAPI\_Questionnaire\_Documentation\_March\_2006\_v1.1.pdf
- “We would like to know about any health problems for which ^Jack has
  been taken to the GP, Health Centre or Health visitor, or to Casualty,
  or you have called NHS direct. How many separate health problems, if
  any, has ^Jack had, not counting any accidents or injuries?
- (not used in final models, for reference only)

  
   


---

### Family characteristics

**Income (for household, equivalised)**

- MCS variable (AOECDUK0 for MCS1), derived, OECD Income Weighted
  Quintiles (UK Analysis)
- Numerical variable from 1 (lowest) to 5 (highest)

**Maternal Education**

- MCS variable (ADACAQ00) NVQ equivalent of highest Academic
  qualification by MCS1
- Numerical variable from 1 (no qualification) to 6 (NVQ Level 5)

**Maternal Mental Health**

- MCS derived variable (APDEAN00), from the parent questionnaire
- Whether mother had ever been diagnosed with depression /
  anxiety
- Binary variable: 0 (No) or 1 (Yes)

**Both Natural Parents in Household**

- MCS variables (ADMINH00: Natural mother in household; and ADFINH00
  for natural fathers)
- If both “Resident in household” then derived variable is “Yes”
  (numerical = 1)
- If one or both of the natural parents are not in household (at age 9
  months) then “No” (0)

**Siblings (number of siblings)**

- MCS variables (ADOTHS00: Number of siblings of cohort member in the
  household)
- Integer ranging from 0 to 9 in our dataset

  
   


---

# Preliminary Analysis

## Method

In this preliminary analysis, we investigate missingness in the
sample (number of missing values for each variable, patterns of
missingness, Little’s MCAR test, etc.), compare the analytic sample with
the rest of the sweep (MCS1 at age 9 months), calculate correlations
among the numerical variables, and related descriptive statistics.
Following this, the main results follow for (A) complete cases only, and
(B) for imputed data over the analytic sample, with and without
controlling for the false discovery rate. Finally, various sensitivity
analyses allows us to probe the robustness of these results and obtain
further insights from them.

  
   


---

## Analytic sample

#### We have 18,754 total “first” cohort members (singletons, or first-born twins / triplets) in MCS1, the survey wave at age 9 months

#### Based on the criteria below we get –> Analytic Sample (9,202 CMs) v. Rest of MCS3 (9,552 CMs)

**Analytic Sample criteria**:

#### 1. That there are valid records for infant motor function (age 9 months)

#### 2. And valid records for the five factor personality model (17 years)

  
   


---

```
## re-encoding from CP1252
```

## MCAR test

```
## |-------------------------------------|
## | misty 0.6.5 (2024-06-29)            |
## | Miscellaneous Functions T. Yanagida |
## |-------------------------------------|
```

```
## 
## Attaching package: 'misty'
```

```
## The following objects are masked from 'package:foreign':
## 
##     read.dta, write.dta
```

```
##  Little's MCAR Test
## 
##       n nIncomp nPattern     chi2   df  pval 
##   18754   11040      215 13275.65 4950 0.000
```

```
## Using dplyr::group_by() groups: missing
```

MAR test


| . | Missing | Not.Missing | P.Value |
| --- | --- | --- | --- |
|  | n = 11040 | n = 7714 |  |
| Sex |  |  | <.001 |
| Male | 5898 (53.4%) | 3721 (48.2%) |  |
| Female | 5142 (46.6%) | 3993 (51.8%) |  |
| NA | 0 (0%) | 0 (0%) |  |
| PTTYPE2 |  |  | <.001 |
| Not applicable | 0 (0%) | 0 (0%) |  |
| England - Advantaged | 2302 (20.9%) | 2380 (30.9%) |  |
| England - Disadvantaged | 2792 (25.3%) | 1787 (23.2%) |  |
| England - Ethnic | 1677 (15.2%) | 724 (9.4%) |  |
| Wales - Advantaged | 462 (4.2%) | 381 (4.9%) |  |
| Wales - Disadvantaged | 1193 (10.8%) | 753 (9.8%) |  |
| Scotland - Advantaged | 649 (5.9%) | 512 (6.6%) |  |
| Scotland - Disadvantaged | 791 (7.2%) | 403 (5.2%) |  |
| Northern Ireland - Advantaged | 389 (3.5%) | 342 (4.4%) |  |
| Northern Ireland - Disadvantaged | 785 (7.1%) | 432 (5.6%) |  |
| NA | 0 (0%) | 0 (0%) |  |
| CM\_Age |  |  | <.001 |
|  | 9.2 (0.5) | 9.2 (0.5) |  |
| Number\_of\_CMs |  |  | <.001 |
|  | 1.0 (0.2) | 1.0 (0.1) |  |
| Both\_Parents\_in\_Household |  |  | <.001 |
|  | 0.8 (0.4) | 0.9 (0.3) |  |
| Siblings |  |  | <.001 |
|  | 1.0 (1.1) | 0.9 (1.0) |  |
| Ethnicity |  |  | <.001 |
| White | 8780 (79.5%) | 6700 (86.9%) |  |
| Mixed | 368 (3.3%) | 193 (2.5%) |  |
| Indian | 278 (2.5%) | 191 (2.5%) |  |
| Pakistani and Bangladeshi | 902 (8.2%) | 361 (4.7%) |  |
| Black or Black British | 479 (4.3%) | 199 (2.6%) |  |
| Other Ethnic group (inc Chinese,Other) | 194 (1.8%) | 70 (0.9%) |  |
| NA | 39 (0.4%) | 0 (0%) |  |
| Income |  |  | <.001 |
|  | 2.5 (1.4) | 3.2 (1.4) |  |
| Maternal\_Education |  |  | <.001 |
|  | 3.1 (1.5) | 3.7 (1.4) |  |
| Maternal\_Age |  |  | <.001 |
|  | 28.6 (6.2) | 30.2 (5.6) |  |
| Maternal\_Mental\_Health |  |  | 0.002 |
|  | 0.2 (0.4) | 0.2 (0.4) |  |
| Maternal\_Alcohol |  |  | <.001 |
|  | 0.5 (1.0) | 0.6 (1.1) |  |
| Baby\_Health\_Problems |  |  | <.001 |
|  | 1.6 (1.9) | 1.6 (1.8) |  |
| Preterm\_Birth |  |  | 0.006 |
|  | 0.1 (0.3) | 0.1 (0.2) |  |
| Low\_Birthweight |  |  | <.001 |
|  | 0.1 (0.3) | 0.1 (0.2) |  |
| Small\_for\_Gestational\_Age |  |  | <.001 |
|  | 0.1 (0.4) | 0.1 (0.3) |  |
| Communication |  |  | <.001 |
|  | 6.7 (1.7) | 6.6 (1.7) |  |
| Motor\_gross |  |  | 0.508 |
|  | 5.5 (1.4) | 5.5 (1.3) |  |
| Motor\_fine |  |  | 0.018 |
|  | 7.5 (0.9) | 7.6 (0.8) |  |
| ToM\_age5 |  |  | 0.001 |
| 0 | 5904 (53.5%) | 6467 (83.8%) |  |
| 1 | 979 (8.9%) | 1247 (16.2%) |  |
| NA | 4157 (37.7%) | 0 (0%) |  |
| allQ |  |  | 0.005 |
|  | 0.1 (0.2) | 0.1 (0.3) |  |
| Prosociality\_age5 |  |  | <.001 |
|  | 9.3 (1.7) | 9.4 (1.6) |  |
| Verbal\_ability\_age5 |  |  | <.001 |
|  | 50.8 (12.7) | 55.6 (10.8) |  |
| Spatial\_ability\_age5 |  |  | <.001 |
|  | 47.7 (11.7) | 51.5 (9.6) |  |
| Independence\_age5 |  |  | <.001 |
|  | 16.1 (3.9) | 16.5 (3.6) |  |
| Emotion\_regl\_age5 |  |  | <.001 |
|  | 13.3 (4.8) | 14.2 (4.7) |  |
| OPEN |  |  | 0.098 |
|  | 13.9 (4.5) | 14.2 (3.9) |  |
| CONSC |  |  | 0.016 |
|  | 13.9 (3.9) | 14.2 (3.5) |  |
| EXTRAV |  |  | 0.181 |
|  | 13.4 (4.2) | 13.5 (4.1) |  |
| AGREE |  |  | 0.832 |
|  | 16.5 (3.9) | 16.6 (3.3) |  |
| NEUROT |  |  | <.001 |
|  | 11.5 (5.0) | 11.9 (4.9) |  |

  
   


---

## Missings

Analytic sample

| variable | n\_miss | pct\_miss |
| --- | --- | --- |
| allQ | 1050 | 11.4 |
| Prosociality\_age5 | 747 | 8.12 |
| Independence\_age5 | 740 | 8.04 |
| Emotion\_regl\_age5 | 739 | 8.03 |
| ToM\_age5 | 564 | 6.13 |
| Spatial\_ability\_age5 | 516 | 5.61 |
| Verbal\_ability\_age5 | 498 | 5.41 |
| Maternal\_Education | 265 | 2.88 |
| Small\_for\_Gestational\_Age | 105 | 1.14 |
| Preterm\_Birth | 97 | 1.05 |
| Income | 18 | 0.196 |
| Low\_Birthweight | 16 | 0.174 |
| Ethnicity | 12 | 0.130 |
| Communication | 11 | 0.120 |
| Maternal\_Alcohol | 8 | 0.0869 |
| Both\_Parents\_in\_Household | 7 | 0.0761 |
| Baby\_Health\_Problems | 4 | 0.0435 |
| Maternal\_Mental\_Health | 3 | 0.0326 |
| Maternal\_Age | 1 | 0.0109 |
| Sex | 0 | 0 |
| PTTYPE2 | 0 | 0 |
| CM\_Age | 0 | 0 |
| Number\_of\_CMs | 0 | 0 |
| Siblings | 0 | 0 |
| Motor\_gross | 0 | 0 |
| Motor\_fine | 0 | 0 |
| OPEN | 0 | 0 |
| CONSC | 0 | 0 |
| EXTRAV | 0 | 0 |
| AGREE | 0 | 0 |
| NEUROT | 0 | 0 |
| analytic\_sample | 0 | 0 |

Rest of sample

| variable | n\_miss | pct\_miss |
| --- | --- | --- |
| EXTRAV | 9043 | 94.7 |
| CONSC | 9035 | 94.6 |
| NEUROT | 9030 | 94.5 |
| OPEN | 9026 | 94.5 |
| AGREE | 9022 | 94.5 |
| allQ | 5099 | 53.4 |
| Prosociality\_age5 | 3631 | 38.0 |
| Emotion\_regl\_age5 | 3614 | 37.8 |
| Independence\_age5 | 3613 | 37.8 |
| ToM\_age5 | 3593 | 37.6 |
| Spatial\_ability\_age5 | 3427 | 35.9 |
| Verbal\_ability\_age5 | 3416 | 35.8 |
| Motor\_fine | 714 | 7.47 |
| Maternal\_Alcohol | 664 | 6.95 |
| Baby\_Health\_Problems | 644 | 6.74 |
| Communication | 644 | 6.74 |
| Motor\_gross | 641 | 6.71 |
| Maternal\_Education | 314 | 3.29 |
| Small\_for\_Gestational\_Age | 152 | 1.59 |
| Preterm\_Birth | 146 | 1.53 |
| Income | 51 | 0.534 |
| Low\_Birthweight | 39 | 0.408 |
| Ethnicity | 27 | 0.283 |
| Both\_Parents\_in\_Household | 23 | 0.241 |
| Maternal\_Mental\_Health | 4 | 0.0419 |
| Maternal\_Age | 2 | 0.0209 |
| Sex | 0 | 0 |
| PTTYPE2 | 0 | 0 |
| CM\_Age | 0 | 0 |
| Number\_of\_CMs | 0 | 0 |
| Siblings | 0 | 0 |
| analytic\_sample | 0 | 0 |

  
   


---

## Unweighted sample bias analysis

```
## Setting theme `JAMA`
## There was an error in 'add_p()/add_difference()' for variable 'PTTYPE2', p-value omitted:
## Error in stats::fisher.test(structure(c(6L, 5L, 5L, 3L, 3L, 3L, 8L, 2L, : FEXACT error 7(location). LDSTP=15120 is too small for this problem,
##   (pastp=1009.05, ipn_0:=ipoin[itp=116]=127, stp[ipn_0]=1074).
## Increase workspace or consider using 'simulate.p.value=TRUE'
```

| **Characteristic** | **Rest**, N = 9,552 | **Sample**, N = 9,202 | **p-value**1 |
| --- | --- | --- | --- |
| Sex, n (%) |  |  | <0.001 |
| Male | 5,173 (54) | 4,446 (48) |  |
| Female | 4,379 (46) | 4,756 (52) |  |
| PTTYPE2, n (%) |  |  |  |
| Not applicable | 0 (0) | 0 (0) |  |
| England - Advantaged | 2,078 (22) | 2,604 (28) |  |
| England - Disadvantaged | 2,399 (25) | 2,180 (24) |  |
| England - Ethnic | 1,204 (13) | 1,197 (13) |  |
| Wales - Advantaged | 422 (4.4) | 421 (4.6) |  |
| Wales - Disadvantaged | 1,043 (11) | 903 (9.8) |  |
| Scotland - Advantaged | 602 (6.3) | 559 (6.1) |  |
| Scotland - Disadvantaged | 734 (7.7) | 460 (5.0) |  |
| Northern Ireland - Advantaged | 361 (3.8) | 370 (4.0) |  |
| Northern Ireland - Disadvantaged | 709 (7.4) | 508 (5.5) |  |
| CM\_Age, n (%) |  |  | <0.001 |
| 8 | 311 (3.3) | 278 (3.0) |  |
| 9 | 7,087 (74) | 7,161 (78) |  |
| 10 | 1,874 (20) | 1,569 (17) |  |
| 11 | 280 (2.9) | 194 (2.1) |  |
| Both\_Parents\_in\_Household, n (%) | 7,507 (79) | 7,963 (87) | <0.001 |
| (Missing) | 23 | 7 |  |
| Siblings, Mean (SD) | 0.97 (1.12) | 0.90 (1.04) | 0.002 |
| Ethnicity, n (%) |  |  | 0.004 |
| White | 7,931 (83) | 7,549 (82) |  |
| Mixed | 299 (3.1) | 262 (2.9) |  |
| Indian | 223 (2.3) | 246 (2.7) |  |
| Pakistani and Bangladeshi | 580 (6.1) | 683 (7.4) |  |
| Black or Black British | 355 (3.7) | 323 (3.5) |  |
| Other Ethnic group (inc Chinese,Other) | 137 (1.4) | 127 (1.4) |  |
| (Missing) | 27 | 12 |  |
| Income, Mean (SD) | 2.54 (1.36) | 3.00 (1.42) | <0.001 |
| (Missing) | 51 | 18 |  |
| Maternal\_Education, Mean (SD) | 3.12 (1.45) | 3.60 (1.44) | <0.001 |
| (Missing) | 314 | 265 |  |
| Maternal\_Age, Mean (SD) | 29 (6) | 30 (6) | <0.001 |
| (Missing) | 2 | 1 |  |
| Maternal\_Mental\_Health, n (%) |  |  | <0.001 |
| 0 | 7,136 (75) | 7,078 (77) |  |
| 1 | 2,412 (25) | 2,121 (23) |  |
| (Missing) | 4 | 3 |  |
| Baby\_Health\_Problems, Mean (SD) | 1.61 (1.91) | 1.63 (1.85) | 0.058 |
| (Missing) | 644 | 4 |  |
| Preterm\_Birth, n (%) |  |  | 0.025 |
| 0 | 8,744 (93) | 8,539 (94) |  |
| 1 | 662 (7.0) | 566 (6.2) |  |
| (Missing) | 146 | 97 |  |
| Low\_Birthweight, n (%) |  |  | <0.001 |
| 0 | 8,718 (92) | 8,540 (93) |  |
| 1 | 795 (8.4) | 646 (7.0) |  |
| (Missing) | 39 | 16 |  |
| Small\_for\_Gestational\_Age, n (%) |  |  | 0.007 |
| 0 | 8,094 (86) | 7,956 (87) |  |
| 1 | 1,306 (14) | 1,141 (13) |  |
| (Missing) | 152 | 105 |  |
| Communication, Mean (SD) | 6.65 (1.74) | 6.60 (1.69) | 0.007 |
| (Missing) | 644 | 11 |  |
| Motor\_gross, Mean (SD) | 5.46 (1.43) | 5.50 (1.33) | 0.18 |
| (Missing) | 641 | 0 |  |
| Motor\_fine, Mean (SD) | 7.55 (0.91) | 7.59 (0.83) | 0.062 |
| (Missing) | 714 | 0 |  |
| ToM\_age5, n (%) |  |  | 0.070 |
| 0 | 5,089 (85) | 7,282 (84) |  |
| 1 | 870 (15) | 1,356 (16) |  |
| (Missing) | 3,593 | 564 |  |
| Prosociality\_age5, Mean (SD) | 9.32 (1.72) | 9.42 (1.65) | 0.004 |
| (Missing) | 3,631 | 747 |  |
| Verbal\_ability\_age5, Mean (SD) | 52 (12) | 54 (12) | <0.001 |
| (Missing) | 3,416 | 498 |  |
| Spatial\_ability\_age5, Mean (SD) | 48 (12) | 51 (10) | <0.001 |
| (Missing) | 3,427 | 516 |  |
| Independence\_age5, Mean (SD) | 16.1 (3.9) | 16.4 (3.7) | <0.001 |
| (Missing) | 3,613 | 740 |  |
| Emotion\_regl\_age5, Mean (SD) | 13.3 (4.8) | 14.1 (4.7) | <0.001 |
| (Missing) | 3,614 | 739 |  |
| OPEN, Mean (SD) | 12.6 (7.5) | 14.3 (3.8) | 0.003 |
| (Missing) | 9,026 | 0 |  |
| CONSC, Mean (SD) | 12.7 (7.4) | 14.2 (3.3) | 0.074 |
| (Missing) | 9,035 | 0 |  |
| EXTRAV, Mean (SD) | 12.2 (6.9) | 13.6 (3.9) | 0.013 |
| (Missing) | 9,043 | 0 |  |
| AGREE, Mean (SD) | 15.4 (7.1) | 16.6 (3.0) | 0.12 |
| (Missing) | 9,022 | 0 |  |
| NEUROT, Mean (SD) | 10.3 (7.0) | 11.9 (4.8) | <0.001 |
| (Missing) | 9,030 | 0 |  |
|  |  |  |  |
| --- | --- | --- | --- |
| 1 Pearson’s Chi-squared test; Wilcoxon rank sum test | | | |

Analytic sample


|  | vars | n | mean | sd | se |
| --- | --- | --- | --- | --- | --- |
| Income | 1 | 9184 | 2.9985845 | 1.4150211 | 0.0147655 |
| Maternal\_Education | 2 | 8937 | 3.6025512 | 1.4394004 | 0.0152260 |
| Maternal\_Age | 3 | 9201 | 29.8877296 | 5.7707190 | 0.0601606 |
| Maternal\_Mental\_Health | 4 | 9199 | 0.2305685 | 0.4212196 | 0.0043918 |
| Baby\_Health\_Problems | 5 | 9198 | 1.6320939 | 1.8501843 | 0.0192916 |
| Both\_Parents\_in\_Household | 6 | 9195 | 0.8660141 | 0.3406556 | 0.0035525 |
| Siblings | 7 | 9202 | 0.9046946 | 1.0420679 | 0.0108631 |
| Communication | 8 | 9191 | 6.6009139 | 1.6876996 | 0.0176041 |
| Motor\_gross | 9 | 9202 | 5.5031515 | 1.3306846 | 0.0138718 |
| Motor\_fine | 10 | 9202 | 7.5854162 | 0.8303138 | 0.0086557 |
| Prosociality\_age5 | 11 | 8455 | 9.4163217 | 1.6459996 | 0.0179008 |
| Verbal\_ability\_age5 | 12 | 8704 | 54.4441636 | 11.6059080 | 0.1243998 |
| Spatial\_ability\_age5 | 13 | 8686 | 50.8990329 | 10.1084189 | 0.1084609 |
| Independence\_age5 | 14 | 8462 | 16.4458757 | 3.6784013 | 0.0399873 |
| Emotion\_regl\_age5 | 15 | 8463 | 14.1107172 | 4.7057252 | 0.0511522 |
| OPEN | 16 | 9202 | 14.2515757 | 3.7650340 | 0.0392489 |
| CONSC | 17 | 9202 | 14.1834384 | 3.2624629 | 0.0340098 |
| EXTRAV | 18 | 9202 | 13.5582482 | 3.8957070 | 0.0406111 |
| AGREE | 19 | 9202 | 16.6352967 | 3.0475359 | 0.0317693 |
| NEUROT | 20 | 9202 | 11.9404477 | 4.7665575 | 0.0496894 |
| analytic\_sample | 21 | 9202 | 2.0000000 | 0.0000000 | 0.0000000 |

Rest of sample


|  | vars | n | mean | sd | se |
| --- | --- | --- | --- | --- | --- |
| Income | 1 | 9501 | 2.5405747 | 1.3621040 | 0.0139742 |
| Maternal\_Education | 2 | 9238 | 3.1236198 | 1.4518666 | 0.0151056 |
| Maternal\_Age | 3 | 9550 | 28.6813613 | 6.2196115 | 0.0636446 |
| Maternal\_Mental\_Health | 4 | 9548 | 0.2526183 | 0.4345366 | 0.0044470 |
| Baby\_Health\_Problems | 5 | 8908 | 1.6133812 | 1.9141941 | 0.0202813 |
| Both\_Parents\_in\_Household | 6 | 9529 | 0.7878056 | 0.4088832 | 0.0041887 |
| Siblings | 7 | 9552 | 0.9693258 | 1.1157496 | 0.0114161 |
| Communication | 8 | 8908 | 6.6541311 | 1.7387912 | 0.0184229 |
| Motor\_gross | 9 | 8911 | 5.4567389 | 1.4295688 | 0.0151440 |
| Motor\_fine | 10 | 8838 | 7.5468432 | 0.9102603 | 0.0096825 |
| Prosociality\_age5 | 11 | 5921 | 9.3197095 | 1.7156764 | 0.0222966 |
| Verbal\_ability\_age5 | 12 | 6136 | 51.6408083 | 12.3603089 | 0.1577926 |
| Spatial\_ability\_age5 | 13 | 6125 | 47.9826939 | 11.5466577 | 0.1475378 |
| Independence\_age5 | 14 | 5939 | 16.1262839 | 3.9314882 | 0.0510153 |
| Emotion\_regl\_age5 | 15 | 5938 | 13.2819131 | 4.7736729 | 0.0619488 |
| OPEN | 16 | 526 | 12.6083650 | 7.5205526 | 0.3279117 |
| CONSC | 17 | 517 | 12.6518375 | 7.3686602 | 0.3240733 |
| EXTRAV | 18 | 509 | 12.2062868 | 6.9295300 | 0.3071460 |
| AGREE | 19 | 530 | 15.3943396 | 7.1036856 | 0.3085644 |
| NEUROT | 20 | 522 | 10.2739464 | 7.0039509 | 0.3065546 |
| analytic\_sample | 21 | 9552 | 1.0000000 | 0.0000000 | 0.0000000 |

```
## 
##  Pearson's Chi-squared test with Yates' continuity correction
## 
## data:  df_all$analytic_sample and df_all$Sex
## X-squared = 63.762, df = 1, p-value = 1.404e-15
```

```
##         
##           Rest Sample   Sum
##   Male    5173   4446  9619
##   Female  4379   4756  9135
##   Sum     9552   9202 18754
```

```
## # A tibble: 4 × 4
## # Groups:   analytic_sample [2]
##   Sex    analytic_sample     n  freq
##   <fct>  <fct>           <int> <dbl>
## 1 Male   Rest             5173  54.2
## 2 Male   Sample           4446  48.3
## 3 Female Rest             4379  45.8
## 4 Female Sample           4756  51.7
```

```
## 
##  Pearson's Chi-squared test
## 
## data:  df_all$analytic_sample and df_all$PTTYPE2
## X-squared = 170.97, df = 8, p-value < 2.2e-16
```

```
##                                          
##                                            Rest Sample   Sum
##   Not applicable                              0      0     0
##   England - Advantaged                     2078   2604  4682
##   England - Disadvantaged                  2399   2180  4579
##   England - Ethnic                         1204   1197  2401
##   Wales - Advantaged                        422    421   843
##   Wales - Disadvantaged                    1043    903  1946
##   Scotland - Advantaged                     602    559  1161
##   Scotland - Disadvantaged                  734    460  1194
##   Northern Ireland - Advantaged             361    370   731
##   Northern Ireland - Disadvantaged          709    508  1217
##   Sum                                      9552   9202 18754
```

```
## # A tibble: 18 × 4
## # Groups:   analytic_sample [2]
##    PTTYPE2                                   analytic_sample     n  freq
##    <fct>                                     <fct>           <int> <dbl>
##  1 "England - Advantaged   "                 Rest             2078 21.8 
##  2 "England - Advantaged   "                 Sample           2604 28.3 
##  3 "England - Disadvantaged"                 Rest             2399 25.1 
##  4 "England - Disadvantaged"                 Sample           2180 23.7 
##  5 "England - Ethnic       "                 Rest             1204 12.6 
##  6 "England - Ethnic       "                 Sample           1197 13.0 
##  7 "Wales - Advantaged     "                 Rest              422  4.42
##  8 "Wales - Advantaged     "                 Sample            421  4.58
##  9 "Wales - Disadvantaged  "                 Rest             1043 10.9 
## 10 "Wales - Disadvantaged  "                 Sample            903  9.81
## 11 "Scotland - Advantaged  "                 Rest              602  6.30
## 12 "Scotland - Advantaged  "                 Sample            559  6.07
## 13 "Scotland - Disadvantaged       "         Rest              734  7.68
## 14 "Scotland - Disadvantaged       "         Sample            460  5.00
## 15 "Northern Ireland - Advantaged  "         Rest              361  3.78
## 16 "Northern Ireland - Advantaged  "         Sample            370  4.02
## 17 "Northern Ireland - Disadvantaged       " Rest              709  7.42
## 18 "Northern Ireland - Disadvantaged       " Sample            508  5.52
```

```
## 
##  Pearson's Chi-squared test
## 
## data:  df_all$analytic_sample and df_all$Ethnicity
## X-squared = 17.293, df = 5, p-value = 0.003977
```

```
##                                          
##                                            Rest Sample   Sum
##   White                                    7931   7549 15480
##   Mixed                                     299    262   561
##   Indian                                    223    246   469
##   Pakistani and Bangladeshi                 580    683  1263
##   Black or Black British                    355    323   678
##   Other Ethnic group (inc Chinese,Other)    137    127   264
##   Sum                                      9525   9190 18715
```

```
## # A tibble: 14 × 4
## # Groups:   analytic_sample [2]
##    Ethnicity                                 analytic_sample     n   freq
##    <fct>                                     <fct>           <int>  <dbl>
##  1 "White  "                                 Rest             7931 83.0  
##  2 "White  "                                 Sample           7549 82.0  
##  3 "Mixed  "                                 Rest              299  3.13 
##  4 "Mixed  "                                 Sample            262  2.85 
##  5 "Indian "                                 Rest              223  2.33 
##  6 "Indian "                                 Sample            246  2.67 
##  7 "Pakistani and Bangladeshi      "         Rest              580  6.07 
##  8 "Pakistani and Bangladeshi      "         Sample            683  7.42 
##  9 "Black or Black British "                 Rest              355  3.72 
## 10 "Black or Black British "                 Sample            323  3.51 
## 11 "Other Ethnic group (inc Chinese,Other) " Rest              137  1.43 
## 12 "Other Ethnic group (inc Chinese,Other) " Sample            127  1.38 
## 13  <NA>                                     Rest               27  0.283
## 14  <NA>                                     Sample             12  0.130
```

Mat Edu: Rest of MCS v. analytic sample

| estimate1 | estimate2 | statistic | p.value | parameter |
| --- | --- | --- | --- | --- |
| 3.12362 | 3.602551 | -22.33005 | 0 | 18162.09 |

```
## Cohen's d |         95% CI
## --------------------------
## -0.33     | [-0.36, -0.30]
## 
## - Estimated using un-pooled SD.
```

BAS Verbal Ability T Score: Rest of MCS v. analytic
sample

| estimate1 | estimate2 | statistic | p.value | parameter |
| --- | --- | --- | --- | --- |
| 51.64081 | 54.44416 | -13.95174 | 0 | 12678.61 |

```
## Cohen's d |         95% CI
## --------------------------
## -0.23     | [-0.27, -0.20]
## 
## - Estimated using un-pooled SD.
```

BAS Spatial Ability T Score: Rest of MCS v. analytic
sample

| estimate1 | estimate2 | statistic | p.value | parameter |
| --- | --- | --- | --- | --- |
| 47.98269 | 50.89903 | -15.92625 | 0 | 12050.17 |

```
## Cohen's d |         95% CI
## --------------------------
## -0.27     | [-0.30, -0.24]
## 
## - Estimated using un-pooled SD.
```

Income: Rest of MCS v. analytic sample

| estimate1 | estimate2 | statistic | p.value | parameter |
| --- | --- | --- | --- | --- |
| 2.540575 | 2.998585 | -22.52912 | 0 | 18586.64 |

```
## Cohen's d |         95% CI
## --------------------------
## -0.33     | [-0.36, -0.30]
## 
## - Estimated using un-pooled SD.
```

  
   


---

## Correlations

```
## Correlation computed with
## • Method: 'pearson'
## • Missing treated using: 'pairwise.complete.obs'
```

| term | Maternal\_Education | Income | Verbal\_ability\_age5 | Spatial\_ability\_age5 | Emotion\_regl\_age5 | Independence\_age5 | Prosociality\_age5 |
| --- | --- | --- | --- | --- | --- | --- | --- |
| Maternal\_Education |  |  |  |  |  |  |  |
| Income | .55 |  |  |  |  |  |  |
| Verbal\_ability\_age5 | .36 | .36 |  |  |  |  |  |
| Spatial\_ability\_age5 | .19 | .19 | .39 |  |  |  |  |
| Emotion\_regl\_age5 | .21 | .24 | .18 | .15 |  |  |  |
| Independence\_age5 | .08 | .07 | .16 | .18 | .26 |  |  |
| Prosociality\_age5 | .06 | .07 | .11 | .09 | .29 | .37 |  |

| Parameter1 | Parameter2 | r | p | df\_error |
| --- | --- | --- | --- | --- |
| Income | Maternal\_Education | 0.5507535 | 0.0e+00 | 8923 |
| Verbal\_ability\_age5 | Spatial\_ability\_age5 | 0.3900656 | 0.0e+00 | 8684 |
| Prosociality\_age5 | Independence\_age5 | 0.3694686 | 0.0e+00 | 8453 |
| Maternal\_Education | Verbal\_ability\_age5 | 0.3603218 | 0.0e+00 | 8465 |
| Income | Verbal\_ability\_age5 | 0.3562443 | 0.0e+00 | 8686 |
| Prosociality\_age5 | Emotion\_regl\_age5 | 0.2938392 | 0.0e+00 | 8453 |
| Independence\_age5 | Emotion\_regl\_age5 | 0.2559052 | 0.0e+00 | 8460 |
| Income | Emotion\_regl\_age5 | 0.2374975 | 0.0e+00 | 8448 |
| Maternal\_Education | Emotion\_regl\_age5 | 0.2113936 | 0.0e+00 | 8258 |
| Income | Spatial\_ability\_age5 | 0.1938838 | 0.0e+00 | 8668 |
| Maternal\_Education | Spatial\_ability\_age5 | 0.1891889 | 0.0e+00 | 8447 |
| Emotion\_regl\_age5 | Verbal\_ability\_age5 | 0.1802704 | 0.0e+00 | 8461 |
| Independence\_age5 | Spatial\_ability\_age5 | 0.1771816 | 0.0e+00 | 8442 |
| Independence\_age5 | Verbal\_ability\_age5 | 0.1591246 | 0.0e+00 | 8460 |
| Emotion\_regl\_age5 | Spatial\_ability\_age5 | 0.1471816 | 0.0e+00 | 8443 |
| Prosociality\_age5 | Verbal\_ability\_age5 | 0.1088261 | 0.0e+00 | 8453 |
| Prosociality\_age5 | Spatial\_ability\_age5 | 0.0868033 | 0.0e+00 | 8435 |
| Maternal\_Education | Independence\_age5 | 0.0840832 | 0.0e+00 | 8258 |
| Income | Independence\_age5 | 0.0714323 | 0.0e+00 | 8447 |
| Income | Prosociality\_age5 | 0.0708621 | 1.0e-07 | 8440 |
| Maternal\_Education | Prosociality\_age5 | 0.0646711 | 3.7e-06 | 8251 |

  
   


---

## GLM - unweighted analysis

```
## 
## Attaching package: 'performance'
```

```
## The following object is masked from 'package:xtable':
## 
##     display
```

```
## 
## Call:
## glm(formula = OPEN ~ Motor_fine + Motor_gross + Communication + 
##     Sex + PTTYPE2 + Ethnicity + CM_Age + Both_Parents_in_Household + 
##     Siblings + Income + Maternal_Education + Maternal_Age + Maternal_Mental_Health + 
##     Small_for_Gestational_Age + ToM_age5 + Prosociality_age5 + 
##     Verbal_ability_age5 + Spatial_ability_age5 + Emotion_regl_age5 + 
##     Independence_age5, data = df)
## 
## Coefficients:
##                                                   Estimate Std. Error t value
## (Intercept)                                       9.390134   0.909015  10.330
## Motor_fine                                        0.022692   0.053515   0.424
## Motor_gross                                       0.118354   0.033824   3.499
## Communication                                     0.011146   0.027638   0.403
## SexFemale                                         0.164115   0.084674   1.938
## PTTYPE2England - Disadvantaged                    0.184524   0.118157   1.562
## PTTYPE2England - Ethnic                           0.285259   0.203968   1.399
## PTTYPE2Wales - Advantaged                        -0.075073   0.201267  -0.373
## PTTYPE2Wales - Disadvantaged                      0.371505   0.155316   2.392
## PTTYPE2Scotland - Advantaged                      0.261426   0.178072   1.468
## PTTYPE2Scotland - Disadvantaged                   0.256486   0.197147   1.301
## PTTYPE2Northern Ireland - Advantaged              0.497526   0.211291   2.355
## PTTYPE2Northern Ireland - Disadvantaged           0.451583   0.194950   2.316
## EthnicityMixed                                    0.719667   0.265681   2.709
## EthnicityIndian                                   0.573924   0.295911   1.940
## EthnicityPakistani and Bangladeshi                1.386195   0.241194   5.747
## EthnicityBlack or Black British                   0.899136   0.280213   3.209
## EthnicityOther Ethnic group (inc Chinese,Other)   0.634184   0.441206   1.437
## CM_Age                                           -0.109299   0.085342  -1.281
## Both_Parents_in_Household                        -0.169945   0.144116  -1.179
## Siblings                                         -0.164148   0.049236  -3.334
## Income                                            0.015644   0.042948   0.364
## Maternal_Education                                0.160005   0.036416   4.394
## Maternal_Age                                      0.049752   0.009216   5.399
## Maternal_Mental_Health                            0.280211   0.099108   2.827
## Small_for_Gestational_Age                        -0.053785   0.130525  -0.412
## ToM_age51                                        -0.126575   0.113281  -1.117
## Prosociality_age5                                -0.001062   0.028227  -0.038
## Verbal_ability_age5                               0.026739   0.004529   5.904
## Spatial_ability_age5                              0.011396   0.004587   2.485
## Emotion_regl_age5                                 0.010981   0.009750   1.126
## Independence_age5                                 0.029695   0.012480   2.379
##                                                  Pr(>|t|)    
## (Intercept)                                       < 2e-16 ***
## Motor_fine                                       0.671559    
## Motor_gross                                      0.000469 ***
## Communication                                    0.686737    
## SexFemale                                        0.052634 .  
## PTTYPE2England - Disadvantaged                   0.118402    
## PTTYPE2England - Ethnic                          0.161989    
## PTTYPE2Wales - Advantaged                        0.709155    
## PTTYPE2Wales - Disadvantaged                     0.016783 *  
## PTTYPE2Scotland - Advantaged                     0.142119    
## PTTYPE2Scotland - Disadvantaged                  0.193299    
## PTTYPE2Northern Ireland - Advantaged             0.018562 *  
## PTTYPE2Northern Ireland - Disadvantaged          0.020561 *  
## EthnicityMixed                                   0.006768 ** 
## EthnicityIndian                                  0.052474 .  
## EthnicityPakistani and Bangladeshi               9.40e-09 ***
## EthnicityBlack or Black British                  0.001338 ** 
## EthnicityOther Ethnic group (inc Chinese,Other)  0.150647    
## CM_Age                                           0.200330    
## Both_Parents_in_Household                        0.238343    
## Siblings                                         0.000860 ***
## Income                                           0.715668    
## Maternal_Education                               1.13e-05 ***
## Maternal_Age                                     6.91e-08 ***
## Maternal_Mental_Health                           0.004705 ** 
## Small_for_Gestational_Age                        0.680303    
## ToM_age51                                        0.263876    
## Prosociality_age5                                0.969981    
## Verbal_ability_age5                              3.69e-09 ***
## Spatial_ability_age5                             0.012989 *  
## Emotion_regl_age5                                0.260053    
## Independence_age5                                0.017361 *  
## ---
## Signif. codes:  0 '***' 0.001 '**' 0.01 '*' 0.05 '.' 0.1 ' ' 1
## 
## (Dispersion parameter for gaussian family taken to be 13.58063)
## 
##     Null deviance: 113125  on 8077  degrees of freedom
## Residual deviance: 109270  on 8046  degrees of freedom
##   (1124 observations deleted due to missingness)
## AIC: 44031
## 
## Number of Fisher Scoring iterations: 2
```

  
   


---

## MIX - clustering at ward level

```
## WeMix v4.0.3
```

```
## Call:
## mix(formula = OPEN ~ Motor_fine + Motor_gross + Communication + 
##     Sex + PTTYPE2 + Ethnicity + CM_Age + Both_Parents_in_Household + 
##     Siblings + Income + Maternal_Education + Maternal_Age + Maternal_Mental_Health + 
##     Small_for_Gestational_Age + ToM_age5 + Prosociality_age5 + 
##     Verbal_ability_age5 + Spatial_ability_age5 + Emotion_regl_age5 + 
##     Independence_age5 + (1 | SPTN00), data = df_, weights = c("AOVWT2", 
##     "W2"))
## 
## Variance terms:
##  Level    Group        Name Variance Std. Error Std.Dev.
##      2   SPTN00 (Intercept)   0.9883    0.07188   0.9941
##      1 Residual              12.7111    0.22035   3.5653
## Groups:
##  Level  Group n size mean wgt  sum wgt
##      2 SPTN00    398        1      398
##      1    Obs   8078     2329 18817459
## 
## Fixed Effects:
##                                                   Estimate Std. Error t value
## (Intercept)                                       9.074386   1.132203   8.015
## Motor_fine                                        0.020430   0.064664   0.316
## Motor_gross                                       0.096614   0.037332   2.588
## Communication                                     0.034026   0.030242   1.125
## SexFemale                                         0.165809   0.106721   1.554
## PTTYPE2England - Disadvantaged                    0.124499   0.140130   0.888
## PTTYPE2England - Ethnic                           0.380100   0.210236   1.808
## PTTYPE2Wales - Advantaged                        -0.246553   0.241792  -1.020
## PTTYPE2Wales - Disadvantaged                      0.126265   0.195974   0.644
## PTTYPE2Scotland - Advantaged                      0.289347   0.195544   1.480
## PTTYPE2Scotland - Disadvantaged                   0.011190   0.226783   0.049
## PTTYPE2Northern Ireland - Advantaged              0.503775   0.222663   2.262
## PTTYPE2Northern Ireland - Disadvantaged           0.397327   0.215088   1.847
## EthnicityMixed                                    0.678436   0.302517   2.243
## EthnicityIndian                                   0.098749   0.333689   0.296
## EthnicityPakistani and Bangladeshi                1.044666   0.331225   3.154
## EthnicityBlack or Black British                   0.959047   0.352175   2.723
## EthnicityOther Ethnic group (inc Chinese,Other)   0.430131   0.499102   0.862
## CM_Age                                           -0.054114   0.103587  -0.522
## Both_Parents_in_Household                        -0.404054   0.186137  -2.171
## Siblings                                         -0.119597   0.063053  -1.897
## Income                                           -0.004171   0.047851  -0.087
## Maternal_Education                                0.171322   0.043661   3.924
## Maternal_Age                                      0.040175   0.010226   3.929
## Maternal_Mental_Health                            0.314050   0.113134   2.776
## Small_for_Gestational_Age                        -0.060527   0.147287  -0.411
## ToM_age51                                        -0.122099   0.135448  -0.901
## Prosociality_age5                                 0.024953   0.032014   0.779
## Verbal_ability_age5                               0.032266   0.005542   5.822
## Spatial_ability_age5                              0.009287   0.005806   1.600
## Emotion_regl_age5                                 0.017536   0.011555   1.518
## Independence_age5                                 0.014314   0.014846   0.964
## 
## lnl= -50623809.27 
## Intraclass Correlation= 0.07214
```

```
## Call:
## mix(formula = OPEN ~ Motor_fine + Motor_gross + Communication + 
##     Sex + PTTYPE2 + Ethnicity + CM_Age + Both_Parents_in_Household + 
##     Siblings + Income + Maternal_Education + Maternal_Age + Maternal_Mental_Health + 
##     Small_for_Gestational_Age + (1 | SPTN00), data = df_, weights = c("AOVWT2", 
##     "W2"))
## 
## Variance terms:
##  Level    Group        Name Variance Std. Error Std.Dev.
##      2   SPTN00 (Intercept)    1.006    0.07531    1.003
##      1 Residual               13.031    0.20348    3.610
## Groups:
##  Level  Group n size mean wgt  sum wgt
##      2 SPTN00    398        1      398
##      1    Obs   8797     2299 20228223
## 
## Fixed Effects:
##                                                   Estimate Std. Error t value
## (Intercept)                                      11.439217   1.024054  11.171
## Motor_fine                                        0.051821   0.062716   0.826
## Motor_gross                                       0.120176   0.036693   3.275
## Communication                                     0.065128   0.029464   2.210
## SexFemale                                         0.175083   0.104832   1.670
## PTTYPE2England - Disadvantaged                    0.057015   0.139201   0.410
## PTTYPE2England - Ethnic                           0.331437   0.198057   1.673
## PTTYPE2Wales - Advantaged                        -0.336479   0.263922  -1.275
## PTTYPE2Wales - Disadvantaged                     -0.001824   0.205986  -0.009
## PTTYPE2Scotland - Advantaged                      0.296610   0.187642   1.581
## PTTYPE2Scotland - Disadvantaged                  -0.100176   0.219615  -0.456
## PTTYPE2Northern Ireland - Advantaged              0.518412   0.216169   2.398
## PTTYPE2Northern Ireland - Disadvantaged           0.258128   0.212013   1.218
## EthnicityMixed                                    0.330163   0.286503   1.152
## EthnicityIndian                                  -0.023469   0.331828  -0.071
## EthnicityPakistani and Bangladeshi                0.802556   0.259523   3.092
## EthnicityBlack or Black British                   0.718845   0.308758   2.328
## EthnicityOther Ethnic group (inc Chinese,Other)   0.304951   0.454441   0.671
## CM_Age                                           -0.080071   0.099449  -0.805
## Both_Parents_in_Household                        -0.355744   0.168982  -2.105
## Siblings                                         -0.172680   0.058913  -2.931
## Income                                            0.024439   0.048901   0.500
## Maternal_Education                                0.237472   0.041583   5.711
## Maternal_Age                                      0.040213   0.009948   4.042
## Maternal_Mental_Health                            0.317886   0.106135   2.995
## Small_for_Gestational_Age                        -0.092341   0.142348  -0.649
## 
## lnl= -54670498.80 
## Intraclass Correlation= 0.07169
```

  
   


---

## Survey-weighted model

```
## 
## Attaching package: 'kableExtra'
```

```
## The following object is masked from 'package:srvyr':
## 
##     group_rows
```

```
## The following object is masked from 'package:dplyr':
## 
##     group_rows
```

```
## 
## Attaching package: 'jtools'
```

```
## The following object is masked from 'package:misty':
## 
##     center
```

```
## The following object is masked from 'package:effectsize':
## 
##     standardize
```

```
## 
## Call:
## svyglm(formula = OPEN ~ Motor_fine + Motor_gross + Communication + 
##     Sex + PTTYPE2 + Ethnicity + CM_Age + Both_Parents_in_Household + 
##     Siblings + Income + Maternal_Education + Maternal_Age + Maternal_Mental_Health + 
##     Small_for_Gestational_Age + ToM_age5 + Prosociality_age5 + 
##     Verbal_ability_age5 + Spatial_ability_age5 + Emotion_regl_age5 + 
##     Independence_age5, design = design_df)
## 
## Survey design:
## Called via srvyr
## 
## Coefficients:
##                                                   Estimate Std. Error t value
## (Intercept)                                       9.023883   1.088721   8.289
## Motor_fine                                        0.011348   0.060254   0.188
## Motor_gross                                       0.109537   0.036467   3.004
## Communication                                     0.028125   0.029210   0.963
## SexFemale                                         0.186129   0.103720   1.795
## PTTYPE2England - Disadvantaged                    0.183602   0.122375   1.500
## PTTYPE2England - Ethnic                           0.334035   0.171661   1.946
## PTTYPE2Wales - Advantaged                        -0.077805   0.214506  -0.363
## PTTYPE2Wales - Disadvantaged                      0.287096   0.169963   1.689
## PTTYPE2Scotland - Advantaged                      0.204755   0.174250   1.175
## PTTYPE2Scotland - Disadvantaged                   0.165369   0.226377   0.731
## PTTYPE2Northern Ireland - Advantaged              0.585071   0.185340   3.157
## PTTYPE2Northern Ireland - Disadvantaged           0.487485   0.182746   2.668
## EthnicityMixed                                    0.763471   0.288607   2.645
## EthnicityIndian                                   0.363426   0.301094   1.207
## EthnicityPakistani and Bangladeshi                1.267330   0.235362   5.385
## EthnicityBlack or Black British                   0.977517   0.264829   3.691
## EthnicityOther Ethnic group (inc Chinese,Other)   0.718187   0.500847   1.434
## CM_Age                                           -0.058598   0.099556  -0.589
## Both_Parents_in_Household                        -0.404898   0.176513  -2.294
## Siblings                                         -0.146976   0.060532  -2.428
## Income                                            0.026882   0.044820   0.600
## Maternal_Education                                0.163462   0.042599   3.837
## Maternal_Age                                      0.044749   0.009641   4.642
## Maternal_Mental_Health                            0.267766   0.109661   2.442
## Small_for_Gestational_Age                        -0.075470   0.140663  -0.537
## ToM_age51                                        -0.133361   0.130040  -1.026
## Prosociality_age5                                 0.011969   0.030994   0.386
## Verbal_ability_age5                               0.029791   0.005461   5.455
## Spatial_ability_age5                              0.009288   0.005397   1.721
## Emotion_regl_age5                                 0.019008   0.011031   1.723
## Independence_age5                                 0.025323   0.014803   1.711
##                                                  Pr(>|t|)    
## (Intercept)                                      2.34e-15 ***
## Motor_fine                                       0.850727    
## Motor_gross                                      0.002855 ** 
## Communication                                    0.336279    
## SexFemale                                        0.073571 .  
## PTTYPE2England - Disadvantaged                   0.134412    
## PTTYPE2England - Ethnic                          0.052449 .  
## PTTYPE2Wales - Advantaged                        0.717030    
## PTTYPE2Wales - Disadvantaged                     0.092057 .  
## PTTYPE2Scotland - Advantaged                     0.240751    
## PTTYPE2Scotland - Disadvantaged                  0.465560    
## PTTYPE2Northern Ireland - Advantaged             0.001731 ** 
## PTTYPE2Northern Ireland - Disadvantaged          0.007988 ** 
## EthnicityMixed                                   0.008520 ** 
## EthnicityIndian                                  0.228222    
## EthnicityPakistani and Bangladeshi               1.32e-07 ***
## EthnicityBlack or Black British                  0.000258 ***
## EthnicityOther Ethnic group (inc Chinese,Other)  0.152461    
## CM_Age                                           0.556509    
## Both_Parents_in_Household                        0.022376 *  
## Siblings                                         0.015672 *  
## Income                                           0.549036    
## Maternal_Education                               0.000147 ***
## Maternal_Age                                     4.86e-06 ***
## Maternal_Mental_Health                           0.015099 *  
## Small_for_Gestational_Age                        0.591923    
## ToM_age51                                        0.305801    
## Prosociality_age5                                0.699609    
## Verbal_ability_age5                              9.15e-08 ***
## Spatial_ability_age5                             0.086114 .  
## Emotion_regl_age5                                0.085718 .  
## Independence_age5                                0.088017 .  
## ---
## Signif. codes:  0 '***' 0.001 '**' 0.01 '*' 0.05 '.' 0.1 ' ' 1
## 
## (Dispersion parameter for gaussian family taken to be 13.64515)
## 
## Number of Fisher Scoring iterations: 2
```

|  |  |
| --- | --- |
| Observations | 8078 |
| Dependent variable | OPEN |
| Type | Survey-weighted linear regression |

|  |  |
| --- | --- |
| R² | 0.04 |
| Adj. R² | -0.05 |

|  | Est. | 2.5% | 97.5% | t val. | p |
| --- | --- | --- | --- | --- | --- |
| (Intercept) | -0.08 | -0.13 | -0.02 | -2.83 | 0.00 |
| Motor\_fine | 0.00 | -0.02 | 0.03 | 0.19 | 0.85 |
| Motor\_gross | 0.04 | 0.01 | 0.06 | 3.00 | 0.00 |
| Communication | 0.01 | -0.01 | 0.04 | 0.96 | 0.34 |
| SexFemale | 0.05 | -0.00 | 0.10 | 1.79 | 0.07 |
| PTTYPE2England - Disadvantaged | 0.05 | -0.02 | 0.11 | 1.50 | 0.13 |
| PTTYPE2England - Ethnic | 0.09 | -0.00 | 0.18 | 1.95 | 0.05 |
| PTTYPE2Wales - Advantaged | -0.02 | -0.13 | 0.09 | -0.36 | 0.72 |
| PTTYPE2Wales - Disadvantaged | 0.08 | -0.01 | 0.17 | 1.69 | 0.09 |
| PTTYPE2Scotland - Advantaged | 0.05 | -0.04 | 0.15 | 1.18 | 0.24 |
| PTTYPE2Scotland - Disadvantaged | 0.04 | -0.08 | 0.16 | 0.73 | 0.47 |
| PTTYPE2Northern Ireland - Advantaged | 0.16 | 0.06 | 0.25 | 3.16 | 0.00 |
| PTTYPE2Northern Ireland - Disadvantaged | 0.13 | 0.03 | 0.23 | 2.67 | 0.01 |
| EthnicityMixed | 0.21 | 0.05 | 0.36 | 2.65 | 0.01 |
| EthnicityIndian | 0.10 | -0.06 | 0.26 | 1.21 | 0.23 |
| EthnicityPakistani and Bangladeshi | 0.34 | 0.22 | 0.46 | 5.38 | 0.00 |
| EthnicityBlack or Black British | 0.26 | 0.12 | 0.40 | 3.69 | 0.00 |
| EthnicityOther Ethnic group (inc Chinese,Other) | 0.19 | -0.07 | 0.46 | 1.43 | 0.15 |
| CM\_Age | -0.01 | -0.03 | 0.02 | -0.59 | 0.56 |
| Both\_Parents\_in\_Household | -0.11 | -0.20 | -0.02 | -2.29 | 0.02 |
| Siblings | -0.04 | -0.07 | -0.01 | -2.43 | 0.02 |
| Income | 0.01 | -0.02 | 0.04 | 0.60 | 0.55 |
| Maternal\_Education | 0.06 | 0.03 | 0.09 | 3.84 | 0.00 |
| Maternal\_Age | 0.07 | 0.04 | 0.10 | 4.64 | 0.00 |
| Maternal\_Mental\_Health | 0.07 | 0.01 | 0.13 | 2.44 | 0.02 |
| Small\_for\_Gestational\_Age | -0.02 | -0.09 | 0.05 | -0.54 | 0.59 |
| ToM\_age51 | -0.04 | -0.10 | 0.03 | -1.03 | 0.31 |
| Prosociality\_age5 | 0.01 | -0.02 | 0.03 | 0.39 | 0.70 |
| Verbal\_ability\_age5 | 0.08 | 0.05 | 0.11 | 5.46 | 0.00 |
| Spatial\_ability\_age5 | 0.02 | -0.00 | 0.05 | 1.72 | 0.09 |
| Emotion\_regl\_age5 | 0.02 | -0.00 | 0.05 | 1.72 | 0.09 |
| Independence\_age5 | 0.02 | -0.00 | 0.05 | 1.71 | 0.09 |
|  |
| --- |
| Standard errors: Robust; Continuous variables are mean-centered and scaled by 1 s.d. |

```
## 
## Call:
## svyglm(formula = NEUROT ~ Motor_fine + Motor_gross + Communication + 
##     Sex + PTTYPE2 + Ethnicity + CM_Age + Both_Parents_in_Household + 
##     Siblings + Income + Maternal_Education + Maternal_Age + Maternal_Mental_Health + 
##     Small_for_Gestational_Age + ToM_age5 + Prosociality_age5 + 
##     Verbal_ability_age5 + Spatial_ability_age5 + Emotion_regl_age5 + 
##     Independence_age5, design = design_df)
## 
## Survey design:
## Called via srvyr
## 
## Coefficients:
##                                                   Estimate Std. Error t value
## (Intercept)                                       9.886443   1.278562   7.732
## Motor_fine                                        0.001780   0.068323   0.026
## Motor_gross                                      -0.117070   0.046452  -2.520
## Communication                                    -0.057715   0.035911  -1.607
## SexFemale                                         3.507976   0.109941  31.908
## PTTYPE2England - Disadvantaged                    0.013227   0.157671   0.084
## PTTYPE2England - Ethnic                           0.175450   0.233872   0.750
## PTTYPE2Wales - Advantaged                         0.230764   0.222288   1.038
## PTTYPE2Wales - Disadvantaged                      0.230201   0.181741   1.267
## PTTYPE2Scotland - Advantaged                      0.654784   0.197828   3.310
## PTTYPE2Scotland - Disadvantaged                   0.394141   0.179427   2.197
## PTTYPE2Northern Ireland - Advantaged             -0.396550   0.228424  -1.736
## PTTYPE2Northern Ireland - Disadvantaged          -0.115051   0.271044  -0.424
## EthnicityMixed                                   -0.538536   0.369051  -1.459
## EthnicityIndian                                  -0.899463   0.424343  -2.120
## EthnicityPakistani and Bangladeshi               -2.069992   0.247311  -8.370
## EthnicityBlack or Black British                  -2.054240   0.284836  -7.212
## EthnicityOther Ethnic group (inc Chinese,Other)  -1.368801   0.526455  -2.600
## CM_Age                                            0.234353   0.116743   2.007
## Both_Parents_in_Household                         0.039610   0.181644   0.218
## Siblings                                         -0.046607   0.061652  -0.756
## Income                                           -0.010050   0.057692  -0.174
## Maternal_Education                                0.033934   0.048812   0.695
## Maternal_Age                                      0.017863   0.012464   1.433
## Maternal_Mental_Health                            0.519192   0.130846   3.968
## Small_for_Gestational_Age                         0.067528   0.177278   0.381
## ToM_age51                                        -0.050867   0.165678  -0.307
## Prosociality_age5                                 0.019809   0.039145   0.506
## Verbal_ability_age5                              -0.001251   0.005363  -0.233
## Spatial_ability_age5                              0.004469   0.006347   0.704
## Emotion_regl_age5                                -0.063278   0.013129  -4.820
## Independence_age5                                -0.056278   0.016920  -3.326
##                                                  Pr(>|t|)    
## (Intercept)                                      1.08e-13 ***
## Motor_fine                                       0.979227    
## Motor_gross                                      0.012162 *  
## Communication                                    0.108895    
## SexFemale                                         < 2e-16 ***
## PTTYPE2England - Disadvantaged                   0.933189    
## PTTYPE2England - Ethnic                          0.453628    
## PTTYPE2Wales - Advantaged                        0.299910    
## PTTYPE2Wales - Disadvantaged                     0.206107    
## PTTYPE2Scotland - Advantaged                     0.001028 ** 
## PTTYPE2Scotland - Disadvantaged                  0.028685 *  
## PTTYPE2Northern Ireland - Advantaged             0.083419 .  
## PTTYPE2Northern Ireland - Disadvantaged          0.671475    
## EthnicityMixed                                   0.145374    
## EthnicityIndian                                  0.034723 *  
## EthnicityPakistani and Bangladeshi               1.31e-15 ***
## EthnicityBlack or Black British                  3.31e-12 ***
## EthnicityOther Ethnic group (inc Chinese,Other)  0.009707 ** 
## CM_Age                                           0.045456 *  
## Both_Parents_in_Household                        0.827503    
## Siblings                                         0.450162    
## Income                                           0.861807    
## Maternal_Education                               0.487385    
## Maternal_Age                                     0.152683    
## Maternal_Mental_Health                           8.76e-05 ***
## Small_for_Gestational_Age                        0.703491    
## ToM_age51                                        0.759006    
## Prosociality_age5                                0.613140    
## Verbal_ability_age5                              0.815667    
## Spatial_ability_age5                             0.481784    
## Emotion_regl_age5                                2.13e-06 ***
## Independence_age5                                0.000972 ***
## ---
## Signif. codes:  0 '***' 0.001 '**' 0.01 '*' 0.05 '.' 0.1 ' ' 1
## 
## (Dispersion parameter for gaussian family taken to be 19.69772)
## 
## Number of Fisher Scoring iterations: 2
```

|  |  |
| --- | --- |
| Observations | 8078 |
| Dependent variable | NEUROT |
| Type | Survey-weighted linear regression |

|  |  |
| --- | --- |
| R² | 0.15 |
| Adj. R² | 0.08 |

|  | Est. | 2.5% | 97.5% | t val. | p |
| --- | --- | --- | --- | --- | --- |
| (Intercept) | -0.36 | -0.40 | -0.31 | -15.86 | 0.00 |
| Motor\_fine | 0.00 | -0.02 | 0.02 | 0.03 | 0.98 |
| Motor\_gross | -0.03 | -0.06 | -0.01 | -2.52 | 0.01 |
| Communication | -0.02 | -0.05 | 0.00 | -1.61 | 0.11 |
| SexFemale | 0.74 | 0.69 | 0.78 | 31.91 | 0.00 |
| PTTYPE2England - Disadvantaged | 0.00 | -0.06 | 0.07 | 0.08 | 0.93 |
| PTTYPE2England - Ethnic | 0.04 | -0.06 | 0.13 | 0.75 | 0.45 |
| PTTYPE2Wales - Advantaged | 0.05 | -0.04 | 0.14 | 1.04 | 0.30 |
| PTTYPE2Wales - Disadvantaged | 0.05 | -0.03 | 0.12 | 1.27 | 0.21 |
| PTTYPE2Scotland - Advantaged | 0.14 | 0.06 | 0.22 | 3.31 | 0.00 |
| PTTYPE2Scotland - Disadvantaged | 0.08 | 0.01 | 0.16 | 2.20 | 0.03 |
| PTTYPE2Northern Ireland - Advantaged | -0.08 | -0.18 | 0.01 | -1.74 | 0.08 |
| PTTYPE2Northern Ireland - Disadvantaged | -0.02 | -0.14 | 0.09 | -0.42 | 0.67 |
| EthnicityMixed | -0.11 | -0.27 | 0.04 | -1.46 | 0.15 |
| EthnicityIndian | -0.19 | -0.36 | -0.01 | -2.12 | 0.03 |
| EthnicityPakistani and Bangladeshi | -0.43 | -0.54 | -0.33 | -8.37 | 0.00 |
| EthnicityBlack or Black British | -0.43 | -0.55 | -0.31 | -7.21 | 0.00 |
| EthnicityOther Ethnic group (inc Chinese,Other) | -0.29 | -0.50 | -0.07 | -2.60 | 0.01 |
| CM\_Age | 0.02 | 0.00 | 0.05 | 2.01 | 0.05 |
| Both\_Parents\_in\_Household | 0.01 | -0.07 | 0.08 | 0.22 | 0.83 |
| Siblings | -0.01 | -0.03 | 0.02 | -0.76 | 0.45 |
| Income | -0.00 | -0.04 | 0.03 | -0.17 | 0.86 |
| Maternal\_Education | 0.01 | -0.02 | 0.04 | 0.70 | 0.49 |
| Maternal\_Age | 0.02 | -0.01 | 0.05 | 1.43 | 0.15 |
| Maternal\_Mental\_Health | 0.11 | 0.05 | 0.16 | 3.97 | 0.00 |
| Small\_for\_Gestational\_Age | 0.01 | -0.06 | 0.09 | 0.38 | 0.70 |
| ToM\_age51 | -0.01 | -0.08 | 0.06 | -0.31 | 0.76 |
| Prosociality\_age5 | 0.01 | -0.02 | 0.03 | 0.51 | 0.61 |
| Verbal\_ability\_age5 | -0.00 | -0.03 | 0.02 | -0.23 | 0.82 |
| Spatial\_ability\_age5 | 0.01 | -0.02 | 0.03 | 0.70 | 0.48 |
| Emotion\_regl\_age5 | -0.06 | -0.09 | -0.04 | -4.82 | 0.00 |
| Independence\_age5 | -0.04 | -0.07 | -0.02 | -3.33 | 0.00 |
|  |
| --- |
| Standard errors: Robust; Continuous variables are mean-centered and scaled by 1 s.d. |

```
## 
## Call:
## svyglm(formula = OPEN ~ Motor_fine + Motor_gross + Communication + 
##     Sex + PTTYPE2 + Ethnicity + CM_Age + Both_Parents_in_Household + 
##     Siblings + Income + Maternal_Education + Maternal_Age + Maternal_Mental_Health + 
##     Small_for_Gestational_Age, design = design_df)
## 
## Survey design:
## Called via srvyr
## 
## Coefficients:
##                                                   Estimate Std. Error t value
## (Intercept)                                      11.177856   0.986236  11.334
## Motor_fine                                        0.037329   0.058789   0.635
## Motor_gross                                       0.126750   0.036045   3.516
## Communication                                     0.057452   0.028135   2.042
## SexFemale                                         0.201462   0.100900   1.997
## PTTYPE2England - Disadvantaged                    0.153131   0.121135   1.264
## PTTYPE2England - Ethnic                           0.279521   0.160347   1.743
## PTTYPE2Wales - Advantaged                        -0.137831   0.222832  -0.619
## PTTYPE2Wales - Disadvantaged                      0.218047   0.170846   1.276
## PTTYPE2Scotland - Advantaged                      0.223656   0.162338   1.378
## PTTYPE2Scotland - Disadvantaged                   0.050852   0.212433   0.239
## PTTYPE2Northern Ireland - Advantaged              0.618556   0.185019   3.343
## PTTYPE2Northern Ireland - Disadvantaged           0.370857   0.178598   2.076
## EthnicityMixed                                    0.446622   0.277758   1.608
## EthnicityIndian                                   0.198296   0.281668   0.704
## EthnicityPakistani and Bangladeshi                1.010804   0.189941   5.322
## EthnicityBlack or Black British                   0.747680   0.244862   3.053
## EthnicityOther Ethnic group (inc Chinese,Other)   0.578910   0.451305   1.283
## CM_Age                                           -0.059522   0.095232  -0.625
## Both_Parents_in_Household                        -0.375807   0.162513  -2.312
## Siblings                                         -0.199513   0.056750  -3.516
## Income                                            0.049035   0.045476   1.078
## Maternal_Education                                0.234609   0.040148   5.844
## Maternal_Age                                      0.045205   0.009396   4.811
## Maternal_Mental_Health                            0.282047   0.102258   2.758
## Small_for_Gestational_Age                        -0.131528   0.138885  -0.947
##                                                  Pr(>|t|)    
## (Intercept)                                       < 2e-16 ***
## Motor_fine                                       0.525848    
## Motor_gross                                      0.000493 ***
## Communication                                    0.041870 *  
## SexFemale                                        0.046608 *  
## PTTYPE2England - Disadvantaged                   0.206992    
## PTTYPE2England - Ethnic                          0.082139 .  
## PTTYPE2Wales - Advantaged                        0.536605    
## PTTYPE2Wales - Disadvantaged                     0.202671    
## PTTYPE2Scotland - Advantaged                     0.169137    
## PTTYPE2Scotland - Disadvantaged                  0.810946    
## PTTYPE2Northern Ireland - Advantaged             0.000914 ***
## PTTYPE2Northern Ireland - Disadvantaged          0.038550 *  
## EthnicityMixed                                   0.108713    
## EthnicityIndian                                  0.481879    
## EthnicityPakistani and Bangladeshi               1.80e-07 ***
## EthnicityBlack or Black British                  0.002428 ** 
## EthnicityOther Ethnic group (inc Chinese,Other)  0.200397    
## CM_Age                                           0.532350    
## Both_Parents_in_Household                        0.021309 *  
## Siblings                                         0.000494 ***
## Income                                           0.281637    
## Maternal_Education                               1.13e-08 ***
## Maternal_Age                                     2.20e-06 ***
## Maternal_Mental_Health                           0.006105 ** 
## Small_for_Gestational_Age                        0.344253    
## ---
## Signif. codes:  0 '***' 0.001 '**' 0.01 '*' 0.05 '.' 0.1 ' ' 1
## 
## (Dispersion parameter for gaussian family taken to be 13.7773)
## 
## Number of Fisher Scoring iterations: 2
```

|  |  |
| --- | --- |
| Observations | 8797 |
| Dependent variable | OPEN |
| Type | Survey-weighted linear regression |

|  |  |
| --- | --- |
| R² | 0.02 |
| Adj. R² | -0.04 |

|  | Est. | 2.5% | 97.5% | t val. | p |
| --- | --- | --- | --- | --- | --- |
| (Intercept) | -0.08 | -0.13 | -0.03 | -2.98 | 0.00 |
| Motor\_fine | 0.01 | -0.02 | 0.03 | 0.63 | 0.53 |
| Motor\_gross | 0.04 | 0.02 | 0.07 | 3.52 | 0.00 |
| Communication | 0.03 | 0.00 | 0.05 | 2.04 | 0.04 |
| SexFemale | 0.05 | 0.00 | 0.11 | 2.00 | 0.05 |
| PTTYPE2England - Disadvantaged | 0.04 | -0.02 | 0.10 | 1.26 | 0.21 |
| PTTYPE2England - Ethnic | 0.07 | -0.01 | 0.16 | 1.74 | 0.08 |
| PTTYPE2Wales - Advantaged | -0.04 | -0.15 | 0.08 | -0.62 | 0.54 |
| PTTYPE2Wales - Disadvantaged | 0.06 | -0.03 | 0.15 | 1.28 | 0.20 |
| PTTYPE2Scotland - Advantaged | 0.06 | -0.03 | 0.14 | 1.38 | 0.17 |
| PTTYPE2Scotland - Disadvantaged | 0.01 | -0.10 | 0.13 | 0.24 | 0.81 |
| PTTYPE2Northern Ireland - Advantaged | 0.17 | 0.07 | 0.26 | 3.34 | 0.00 |
| PTTYPE2Northern Ireland - Disadvantaged | 0.10 | 0.01 | 0.19 | 2.08 | 0.04 |
| EthnicityMixed | 0.12 | -0.03 | 0.27 | 1.61 | 0.11 |
| EthnicityIndian | 0.05 | -0.09 | 0.20 | 0.70 | 0.48 |
| EthnicityPakistani and Bangladeshi | 0.27 | 0.17 | 0.37 | 5.32 | 0.00 |
| EthnicityBlack or Black British | 0.20 | 0.07 | 0.33 | 3.05 | 0.00 |
| EthnicityOther Ethnic group (inc Chinese,Other) | 0.15 | -0.08 | 0.39 | 1.28 | 0.20 |
| CM\_Age | -0.01 | -0.03 | 0.02 | -0.63 | 0.53 |
| Both\_Parents\_in\_Household | -0.10 | -0.19 | -0.02 | -2.31 | 0.02 |
| Siblings | -0.05 | -0.08 | -0.02 | -3.52 | 0.00 |
| Income | 0.02 | -0.02 | 0.05 | 1.08 | 0.28 |
| Maternal\_Education | 0.09 | 0.06 | 0.12 | 5.84 | 0.00 |
| Maternal\_Age | 0.07 | 0.04 | 0.10 | 4.81 | 0.00 |
| Maternal\_Mental\_Health | 0.08 | 0.02 | 0.13 | 2.76 | 0.01 |
| Small\_for\_Gestational\_Age | -0.04 | -0.11 | 0.04 | -0.95 | 0.34 |
|  |
| --- |
| Standard errors: Robust; Continuous variables are mean-centered and scaled by 1 s.d. |

```
## 
## Call:
## svyglm(formula = CONSC ~ Motor_fine + Motor_gross + Communication + 
##     Sex + PTTYPE2 + Ethnicity + CM_Age + Both_Parents_in_Household + 
##     Siblings + Income + Maternal_Education + Maternal_Age + Maternal_Mental_Health + 
##     Small_for_Gestational_Age, design = design_df)
## 
## Survey design:
## Called via srvyr
## 
## Coefficients:
##                                                    Estimate Std. Error t value
## (Intercept)                                      12.9222211  0.8102963  15.948
## Motor_fine                                        0.0844797  0.0500729   1.687
## Motor_gross                                       0.0521819  0.0324310   1.609
## Communication                                     0.0907343  0.0271510   3.342
## SexFemale                                         0.1163897  0.0837363   1.390
## PTTYPE2England - Disadvantaged                   -0.1651513  0.1221491  -1.352
## PTTYPE2England - Ethnic                          -0.3401157  0.1576225  -2.158
## PTTYPE2Wales - Advantaged                         0.0546282  0.2254774   0.242
## PTTYPE2Wales - Disadvantaged                     -0.3011639  0.1390992  -2.165
## PTTYPE2Scotland - Advantaged                     -0.1970739  0.1533528  -1.285
## PTTYPE2Scotland - Disadvantaged                  -0.1343220  0.1632041  -0.823
## PTTYPE2Northern Ireland - Advantaged              0.4579485  0.2050790   2.233
## PTTYPE2Northern Ireland - Disadvantaged           0.0006767  0.2021569   0.003
## EthnicityMixed                                   -0.6283682  0.2164398  -2.903
## EthnicityIndian                                   0.2097487  0.2707153   0.775
## EthnicityPakistani and Bangladeshi                0.6094665  0.1627302   3.745
## EthnicityBlack or Black British                   0.3094820  0.2897191   1.068
## EthnicityOther Ethnic group (inc Chinese,Other)   0.2063419  0.2161588   0.955
## CM_Age                                           -0.0705876  0.0745544  -0.947
## Both_Parents_in_Household                         0.2369865  0.1406341   1.685
## Siblings                                         -0.1575211  0.0468091  -3.365
## Income                                            0.0476593  0.0417490   1.142
## Maternal_Education                                0.0763540  0.0349200   2.187
## Maternal_Age                                     -0.0018794  0.0079375  -0.237
## Maternal_Mental_Health                           -0.1469972  0.0947409  -1.552
## Small_for_Gestational_Age                        -0.0921157  0.1188267  -0.775
##                                                  Pr(>|t|)    
## (Intercept)                                       < 2e-16 ***
## Motor_fine                                       0.092434 .  
## Motor_gross                                      0.108481    
## Communication                                    0.000919 ***
## SexFemale                                        0.165392    
## PTTYPE2England - Disadvantaged                   0.177200    
## PTTYPE2England - Ethnic                          0.031598 *  
## PTTYPE2Wales - Advantaged                        0.808701    
## PTTYPE2Wales - Disadvantaged                     0.031029 *  
## PTTYPE2Scotland - Advantaged                     0.199574    
## PTTYPE2Scotland - Disadvantaged                  0.411029    
## PTTYPE2Northern Ireland - Advantaged             0.026154 *  
## PTTYPE2Northern Ireland - Disadvantaged          0.997331    
## EthnicityMixed                                   0.003919 ** 
## EthnicityIndian                                  0.438964    
## EthnicityPakistani and Bangladeshi               0.000209 ***
## EthnicityBlack or Black British                  0.286132    
## EthnicityOther Ethnic group (inc Chinese,Other)  0.340421    
## CM_Age                                           0.344373    
## Both_Parents_in_Household                        0.092821 .  
## Siblings                                         0.000846 ***
## Income                                           0.254384    
## Maternal_Education                               0.029412 *  
## Maternal_Age                                     0.812964    
## Maternal_Mental_Health                           0.121634    
## Small_for_Gestational_Age                        0.438719    
## ---
## Signif. codes:  0 '***' 0.001 '**' 0.01 '*' 0.05 '.' 0.1 ' ' 1
## 
## (Dispersion parameter for gaussian family taken to be 10.56035)
## 
## Number of Fisher Scoring iterations: 2
```

|  |  |
| --- | --- |
| Observations | 8797 |
| Dependent variable | CONSC |
| Type | Survey-weighted linear regression |

|  |  |
| --- | --- |
| R² | 0.02 |
| Adj. R² | -0.05 |

|  | Est. | 2.5% | 97.5% | t val. | p |
| --- | --- | --- | --- | --- | --- |
| (Intercept) | -0.00 | -0.06 | 0.05 | -0.10 | 0.92 |
| Motor\_fine | 0.02 | -0.00 | 0.05 | 1.69 | 0.09 |
| Motor\_gross | 0.02 | -0.00 | 0.05 | 1.61 | 0.11 |
| Communication | 0.05 | 0.02 | 0.07 | 3.34 | 0.00 |
| SexFemale | 0.04 | -0.01 | 0.09 | 1.39 | 0.17 |
| PTTYPE2England - Disadvantaged | -0.05 | -0.12 | 0.02 | -1.35 | 0.18 |
| PTTYPE2England - Ethnic | -0.10 | -0.20 | -0.01 | -2.16 | 0.03 |
| PTTYPE2Wales - Advantaged | 0.02 | -0.12 | 0.15 | 0.24 | 0.81 |
| PTTYPE2Wales - Disadvantaged | -0.09 | -0.18 | -0.01 | -2.17 | 0.03 |
| PTTYPE2Scotland - Advantaged | -0.06 | -0.15 | 0.03 | -1.29 | 0.20 |
| PTTYPE2Scotland - Disadvantaged | -0.04 | -0.14 | 0.06 | -0.82 | 0.41 |
| PTTYPE2Northern Ireland - Advantaged | 0.14 | 0.02 | 0.26 | 2.23 | 0.03 |
| PTTYPE2Northern Ireland - Disadvantaged | 0.00 | -0.12 | 0.12 | 0.00 | 1.00 |
| EthnicityMixed | -0.19 | -0.32 | -0.06 | -2.90 | 0.00 |
| EthnicityIndian | 0.06 | -0.10 | 0.23 | 0.77 | 0.44 |
| EthnicityPakistani and Bangladeshi | 0.19 | 0.09 | 0.28 | 3.75 | 0.00 |
| EthnicityBlack or Black British | 0.09 | -0.08 | 0.27 | 1.07 | 0.29 |
| EthnicityOther Ethnic group (inc Chinese,Other) | 0.06 | -0.07 | 0.19 | 0.95 | 0.34 |
| CM\_Age | -0.01 | -0.03 | 0.01 | -0.95 | 0.34 |
| Both\_Parents\_in\_Household | 0.07 | -0.01 | 0.16 | 1.69 | 0.09 |
| Siblings | -0.05 | -0.08 | -0.02 | -3.37 | 0.00 |
| Income | 0.02 | -0.01 | 0.06 | 1.14 | 0.25 |
| Maternal\_Education | 0.03 | 0.00 | 0.06 | 2.19 | 0.03 |
| Maternal\_Age | -0.00 | -0.03 | 0.02 | -0.24 | 0.81 |
| Maternal\_Mental\_Health | -0.05 | -0.10 | 0.01 | -1.55 | 0.12 |
| Small\_for\_Gestational\_Age | -0.03 | -0.10 | 0.04 | -0.78 | 0.44 |
|  |
| --- |
| Standard errors: Robust; Continuous variables are mean-centered and scaled by 1 s.d. |

```
## 
## Call:
## svyglm(formula = EXTRAV ~ Motor_fine + Motor_gross + Communication + 
##     Sex + PTTYPE2 + Ethnicity + CM_Age + Both_Parents_in_Household + 
##     Siblings + Income + Maternal_Education + Maternal_Age + Maternal_Mental_Health + 
##     Small_for_Gestational_Age, design = design_df)
## 
## Survey design:
## Called via srvyr
## 
## Coefficients:
##                                                   Estimate Std. Error t value
## (Intercept)                                      12.651628   0.913587  13.848
## Motor_fine                                        0.005820   0.059028   0.099
## Motor_gross                                       0.059143   0.038372   1.541
## Communication                                     0.071745   0.030891   2.323
## SexFemale                                         0.290641   0.100457   2.893
## PTTYPE2England - Disadvantaged                   -0.191769   0.128673  -1.490
## PTTYPE2England - Ethnic                          -0.302922   0.200986  -1.507
## PTTYPE2Wales - Advantaged                        -0.132813   0.160212  -0.829
## PTTYPE2Wales - Disadvantaged                     -0.151057   0.141361  -1.069
## PTTYPE2Scotland - Advantaged                      0.152522   0.196369   0.777
## PTTYPE2Scotland - Disadvantaged                   0.049758   0.211139   0.236
## PTTYPE2Northern Ireland - Advantaged              0.575784   0.259480   2.219
## PTTYPE2Northern Ireland - Disadvantaged           0.358271   0.216189   1.657
## EthnicityMixed                                   -0.287527   0.319875  -0.899
## EthnicityIndian                                   0.627424   0.349903   1.793
## EthnicityPakistani and Bangladeshi                0.719213   0.232119   3.098
## EthnicityBlack or Black British                  -0.318439   0.280420  -1.136
## EthnicityOther Ethnic group (inc Chinese,Other)  -0.304241   0.341337  -0.891
## CM_Age                                            0.008184   0.091844   0.089
## Both_Parents_in_Household                        -0.075240   0.161625  -0.466
## Siblings                                         -0.026204   0.051587  -0.508
## Income                                            0.192550   0.050986   3.777
## Maternal_Education                               -0.014086   0.041911  -0.336
## Maternal_Age                                     -0.015467   0.010393  -1.488
## Maternal_Mental_Health                           -0.492170   0.121170  -4.062
## Small_for_Gestational_Age                        -0.205355   0.144005  -1.426
##                                                  Pr(>|t|)    
## (Intercept)                                       < 2e-16 ***
## Motor_fine                                       0.921516    
## Motor_gross                                      0.124114    
## Communication                                    0.020756 *  
## SexFemale                                        0.004043 ** 
## PTTYPE2England - Disadvantaged                   0.136996    
## PTTYPE2England - Ethnic                          0.132632    
## PTTYPE2Wales - Advantaged                        0.407658    
## PTTYPE2Wales - Disadvantaged                     0.285964    
## PTTYPE2Scotland - Advantaged                     0.437834    
## PTTYPE2Scotland - Disadvantaged                  0.813827    
## PTTYPE2Northern Ireland - Advantaged             0.027103 *  
## PTTYPE2Northern Ireland - Disadvantaged          0.098338 .  
## EthnicityMixed                                   0.369315    
## EthnicityIndian                                  0.073781 .  
## EthnicityPakistani and Bangladeshi               0.002096 ** 
## EthnicityBlack or Black British                  0.256880    
## EthnicityOther Ethnic group (inc Chinese,Other)  0.373346    
## CM_Age                                           0.929047    
## Both_Parents_in_Household                        0.641836    
## Siblings                                         0.611793    
## Income                                           0.000186 ***
## Maternal_Education                               0.736990    
## Maternal_Age                                     0.137579    
## Maternal_Mental_Health                           5.97e-05 ***
## Small_for_Gestational_Age                        0.154719    
## ---
## Signif. codes:  0 '***' 0.001 '**' 0.01 '*' 0.05 '.' 0.1 ' ' 1
## 
## (Dispersion parameter for gaussian family taken to be 15.43992)
## 
## Number of Fisher Scoring iterations: 2
```

|  |  |
| --- | --- |
| Observations | 8797 |
| Dependent variable | EXTRAV |
| Type | Survey-weighted linear regression |

|  |  |
| --- | --- |
| R² | 0.01 |
| Adj. R² | -0.05 |

|  | Est. | 2.5% | 97.5% | t val. | p |
| --- | --- | --- | --- | --- | --- |
| (Intercept) | -0.03 | -0.08 | 0.01 | -1.41 | 0.16 |
| Motor\_fine | 0.00 | -0.02 | 0.03 | 0.10 | 0.92 |
| Motor\_gross | 0.02 | -0.01 | 0.04 | 1.54 | 0.12 |
| Communication | 0.03 | 0.00 | 0.06 | 2.32 | 0.02 |
| SexFemale | 0.07 | 0.02 | 0.12 | 2.89 | 0.00 |
| PTTYPE2England - Disadvantaged | -0.05 | -0.11 | 0.02 | -1.49 | 0.14 |
| PTTYPE2England - Ethnic | -0.08 | -0.18 | 0.02 | -1.51 | 0.13 |
| PTTYPE2Wales - Advantaged | -0.03 | -0.11 | 0.05 | -0.83 | 0.41 |
| PTTYPE2Wales - Disadvantaged | -0.04 | -0.11 | 0.03 | -1.07 | 0.29 |
| PTTYPE2Scotland - Advantaged | 0.04 | -0.06 | 0.14 | 0.78 | 0.44 |
| PTTYPE2Scotland - Disadvantaged | 0.01 | -0.09 | 0.12 | 0.24 | 0.81 |
| PTTYPE2Northern Ireland - Advantaged | 0.15 | 0.02 | 0.28 | 2.22 | 0.03 |
| PTTYPE2Northern Ireland - Disadvantaged | 0.09 | -0.02 | 0.20 | 1.66 | 0.10 |
| EthnicityMixed | -0.07 | -0.23 | 0.09 | -0.90 | 0.37 |
| EthnicityIndian | 0.16 | -0.02 | 0.33 | 1.79 | 0.07 |
| EthnicityPakistani and Bangladeshi | 0.18 | 0.07 | 0.30 | 3.10 | 0.00 |
| EthnicityBlack or Black British | -0.08 | -0.22 | 0.06 | -1.14 | 0.26 |
| EthnicityOther Ethnic group (inc Chinese,Other) | -0.08 | -0.25 | 0.09 | -0.89 | 0.37 |
| CM\_Age | 0.00 | -0.02 | 0.02 | 0.09 | 0.93 |
| Both\_Parents\_in\_Household | -0.02 | -0.10 | 0.06 | -0.47 | 0.64 |
| Siblings | -0.01 | -0.03 | 0.02 | -0.51 | 0.61 |
| Income | 0.07 | 0.03 | 0.10 | 3.78 | 0.00 |
| Maternal\_Education | -0.00 | -0.03 | 0.02 | -0.34 | 0.74 |
| Maternal\_Age | -0.02 | -0.05 | 0.01 | -1.49 | 0.14 |
| Maternal\_Mental\_Health | -0.12 | -0.19 | -0.06 | -4.06 | 0.00 |
| Small\_for\_Gestational\_Age | -0.05 | -0.12 | 0.02 | -1.43 | 0.15 |
|  |
| --- |
| Standard errors: Robust; Continuous variables are mean-centered and scaled by 1 s.d. |

```
## 
## Call:
## svyglm(formula = AGREE ~ Motor_fine + Motor_gross + Communication + 
##     Sex + PTTYPE2 + Ethnicity + CM_Age + Both_Parents_in_Household + 
##     Siblings + Income + Maternal_Education + Maternal_Age + Maternal_Mental_Health + 
##     Small_for_Gestational_Age, design = design_df)
## 
## Survey design:
## Called via srvyr
## 
## Coefficients:
##                                                    Estimate Std. Error t value
## (Intercept)                                      13.8696407  0.7999040  17.339
## Motor_fine                                        0.0560222  0.0456019   1.229
## Motor_gross                                      -0.0005722  0.0306624  -0.019
## Communication                                     0.0354414  0.0258456   1.371
## SexFemale                                         1.0655751  0.0836254  12.742
## PTTYPE2England - Disadvantaged                   -0.0399049  0.0964888  -0.414
## PTTYPE2England - Ethnic                           0.1076135  0.1510622   0.712
## PTTYPE2Wales - Advantaged                         0.3078077  0.1392845   2.210
## PTTYPE2Wales - Disadvantaged                      0.2172971  0.1609098   1.350
## PTTYPE2Scotland - Advantaged                      0.3780023  0.1090416   3.467
## PTTYPE2Scotland - Disadvantaged                   0.4574095  0.1328966   3.442
## PTTYPE2Northern Ireland - Advantaged              0.3349957  0.1290637   2.596
## PTTYPE2Northern Ireland - Disadvantaged           0.3539019  0.1697080   2.085
## EthnicityMixed                                   -0.3632503  0.2117812  -1.715
## EthnicityIndian                                   0.2695116  0.2339038   1.152
## EthnicityPakistani and Bangladeshi                0.6057377  0.1984907   3.052
## EthnicityBlack or Black British                  -0.3623531  0.2421008  -1.497
## EthnicityOther Ethnic group (inc Chinese,Other)   0.2281653  0.2164669   1.054
## CM_Age                                            0.0666827  0.0756526   0.881
## Both_Parents_in_Household                         0.1275489  0.1282838   0.994
## Siblings                                         -0.0884836  0.0435082  -2.034
## Income                                            0.0392227  0.0372716   1.052
## Maternal_Education                                0.0446583  0.0340847   1.310
## Maternal_Age                                      0.0169375  0.0077835   2.176
## Maternal_Mental_Health                           -0.0929991  0.0945374  -0.984
## Small_for_Gestational_Age                         0.0286812  0.1018480   0.282
##                                                  Pr(>|t|)    
## (Intercept)                                       < 2e-16 ***
## Motor_fine                                       0.220051    
## Motor_gross                                      0.985121    
## Communication                                    0.171134    
## SexFemale                                         < 2e-16 ***
## PTTYPE2England - Disadvantaged                   0.679432    
## PTTYPE2England - Ethnic                          0.476687    
## PTTYPE2Wales - Advantaged                        0.027732 *  
## PTTYPE2Wales - Disadvantaged                     0.177718    
## PTTYPE2Scotland - Advantaged                     0.000590 ***
## PTTYPE2Scotland - Disadvantaged                  0.000645 ***
## PTTYPE2Northern Ireland - Advantaged             0.009825 ** 
## PTTYPE2Northern Ireland - Disadvantaged          0.037733 *  
## EthnicityMixed                                   0.087157 .  
## EthnicityIndian                                  0.249982    
## EthnicityPakistani and Bangladeshi               0.002442 ** 
## EthnicityBlack or Black British                  0.135337    
## EthnicityOther Ethnic group (inc Chinese,Other)  0.292563    
## CM_Age                                           0.378665    
## Both_Parents_in_Household                        0.320751    
## Siblings                                         0.042704 *  
## Income                                           0.293338    
## Maternal_Education                               0.190950    
## Maternal_Age                                     0.030191 *  
## Maternal_Mental_Health                           0.325902    
## Small_for_Gestational_Age                        0.778404    
## ---
## Signif. codes:  0 '***' 0.001 '**' 0.01 '*' 0.05 '.' 0.1 ' ' 1
## 
## (Dispersion parameter for gaussian family taken to be 8.951561)
## 
## Number of Fisher Scoring iterations: 2
```

|  |  |
| --- | --- |
| Observations | 8797 |
| Dependent variable | AGREE |
| Type | Survey-weighted linear regression |

|  |  |
| --- | --- |
| R² | 0.04 |
| Adj. R² | -0.02 |

|  | Est. | 2.5% | 97.5% | t val. | p |
| --- | --- | --- | --- | --- | --- |
| (Intercept) | -0.21 | -0.25 | -0.16 | -8.25 | 0.00 |
| Motor\_fine | 0.02 | -0.01 | 0.04 | 1.23 | 0.22 |
| Motor\_gross | -0.00 | -0.03 | 0.03 | -0.02 | 0.99 |
| Communication | 0.02 | -0.01 | 0.05 | 1.37 | 0.17 |
| SexFemale | 0.35 | 0.30 | 0.40 | 12.74 | 0.00 |
| PTTYPE2England - Disadvantaged | -0.01 | -0.08 | 0.05 | -0.41 | 0.68 |
| PTTYPE2England - Ethnic | 0.04 | -0.06 | 0.13 | 0.71 | 0.48 |
| PTTYPE2Wales - Advantaged | 0.10 | 0.01 | 0.19 | 2.21 | 0.03 |
| PTTYPE2Wales - Disadvantaged | 0.07 | -0.03 | 0.18 | 1.35 | 0.18 |
| PTTYPE2Scotland - Advantaged | 0.12 | 0.05 | 0.19 | 3.47 | 0.00 |
| PTTYPE2Scotland - Disadvantaged | 0.15 | 0.06 | 0.24 | 3.44 | 0.00 |
| PTTYPE2Northern Ireland - Advantaged | 0.11 | 0.03 | 0.19 | 2.60 | 0.01 |
| PTTYPE2Northern Ireland - Disadvantaged | 0.12 | 0.01 | 0.23 | 2.09 | 0.04 |
| EthnicityMixed | -0.12 | -0.26 | 0.02 | -1.72 | 0.09 |
| EthnicityIndian | 0.09 | -0.06 | 0.24 | 1.15 | 0.25 |
| EthnicityPakistani and Bangladeshi | 0.20 | 0.07 | 0.33 | 3.05 | 0.00 |
| EthnicityBlack or Black British | -0.12 | -0.28 | 0.04 | -1.50 | 0.14 |
| EthnicityOther Ethnic group (inc Chinese,Other) | 0.07 | -0.06 | 0.21 | 1.05 | 0.29 |
| CM\_Age | 0.01 | -0.01 | 0.03 | 0.88 | 0.38 |
| Both\_Parents\_in\_Household | 0.04 | -0.04 | 0.12 | 0.99 | 0.32 |
| Siblings | -0.03 | -0.06 | -0.00 | -2.03 | 0.04 |
| Income | 0.02 | -0.02 | 0.05 | 1.05 | 0.29 |
| Maternal\_Education | 0.02 | -0.01 | 0.05 | 1.31 | 0.19 |
| Maternal\_Age | 0.03 | 0.00 | 0.06 | 2.18 | 0.03 |
| Maternal\_Mental\_Health | -0.03 | -0.09 | 0.03 | -0.98 | 0.33 |
| Small\_for\_Gestational\_Age | 0.01 | -0.06 | 0.08 | 0.28 | 0.78 |
|  |
| --- |
| Standard errors: Robust; Continuous variables are mean-centered and scaled by 1 s.d. |

```
## 
## Call:
## svyglm(formula = NEUROT ~ Motor_fine + Motor_gross + Communication + 
##     Sex + PTTYPE2 + Ethnicity + CM_Age + Both_Parents_in_Household + 
##     Siblings + Income + Maternal_Education + Maternal_Age + Maternal_Mental_Health + 
##     Small_for_Gestational_Age, design = design_df)
## 
## Survey design:
## Called via srvyr
## 
## Coefficients:
##                                                   Estimate Std. Error t value
## (Intercept)                                       9.851844   1.163127   8.470
## Motor_fine                                       -0.054832   0.068400  -0.802
## Motor_gross                                      -0.101578   0.042619  -2.383
## Communication                                    -0.088367   0.034242  -2.581
## SexFemale                                         3.322726   0.104222  31.881
## PTTYPE2England - Disadvantaged                   -0.013876   0.152295  -0.091
## PTTYPE2England - Ethnic                          -0.006780   0.225061  -0.030
## PTTYPE2Wales - Advantaged                         0.244549   0.230979   1.059
## PTTYPE2Wales - Disadvantaged                      0.304001   0.171679   1.771
## PTTYPE2Scotland - Advantaged                      0.553913   0.190545   2.907
## PTTYPE2Scotland - Disadvantaged                   0.357211   0.182179   1.961
## PTTYPE2Northern Ireland - Advantaged             -0.477673   0.234045  -2.041
## PTTYPE2Northern Ireland - Disadvantaged           0.016962   0.261018   0.065
## EthnicityMixed                                   -0.473176   0.344433  -1.374
## EthnicityIndian                                  -0.836152   0.407174  -2.054
## EthnicityPakistani and Bangladeshi               -1.644321   0.207803  -7.913
## EthnicityBlack or Black British                  -1.879121   0.280790  -6.692
## EthnicityOther Ethnic group (inc Chinese,Other)  -0.745515   0.438645  -1.700
## CM_Age                                            0.194795   0.114857   1.696
## Both_Parents_in_Household                        -0.071290   0.175405  -0.406
## Siblings                                         -0.065284   0.058760  -1.111
## Income                                           -0.029254   0.053819  -0.544
## Maternal_Education                                0.018647   0.047059   0.396
## Maternal_Age                                      0.009733   0.011990   0.812
## Maternal_Mental_Health                            0.646191   0.123971   5.212
## Small_for_Gestational_Age                         0.103986   0.171017   0.608
##                                                  Pr(>|t|)    
## (Intercept)                                      6.13e-16 ***
## Motor_fine                                        0.42329    
## Motor_gross                                       0.01767 *  
## Communication                                     0.01025 *  
## SexFemale                                         < 2e-16 ***
## PTTYPE2England - Disadvantaged                    0.92745    
## PTTYPE2England - Ethnic                           0.97599    
## PTTYPE2Wales - Advantaged                         0.29042    
## PTTYPE2Wales - Disadvantaged                      0.07744 .  
## PTTYPE2Scotland - Advantaged                      0.00387 ** 
## PTTYPE2Scotland - Disadvantaged                   0.05067 .  
## PTTYPE2Northern Ireland - Advantaged              0.04198 *  
## PTTYPE2Northern Ireland - Disadvantaged           0.94822    
## EthnicityMixed                                    0.17035    
## EthnicityIndian                                   0.04073 *  
## EthnicityPakistani and Bangladeshi               3.06e-14 ***
## EthnicityBlack or Black British                  8.32e-11 ***
## EthnicityOther Ethnic group (inc Chinese,Other)   0.09006 .  
## CM_Age                                            0.09075 .  
## Both_Parents_in_Household                         0.68466    
## Siblings                                          0.26728    
## Income                                            0.58708    
## Maternal_Education                                0.69216    
## Maternal_Age                                      0.41746    
## Maternal_Mental_Health                           3.13e-07 ***
## Small_for_Gestational_Age                         0.54354    
## ---
## Signif. codes:  0 '***' 0.001 '**' 0.01 '*' 0.05 '.' 0.1 ' ' 1
## 
## (Dispersion parameter for gaussian family taken to be 19.58275)
## 
## Number of Fisher Scoring iterations: 2
```

|  |  |
| --- | --- |
| Observations | 8797 |
| Dependent variable | NEUROT |
| Type | Survey-weighted linear regression |

|  |  |
| --- | --- |
| R² | 0.14 |
| Adj. R² | 0.08 |

|  | Est. | 2.5% | 97.5% | t val. | p |
| --- | --- | --- | --- | --- | --- |
| (Intercept) | -0.33 | -0.38 | -0.29 | -15.44 | 0.00 |
| Motor\_fine | -0.01 | -0.03 | 0.01 | -0.80 | 0.42 |
| Motor\_gross | -0.03 | -0.05 | -0.00 | -2.38 | 0.02 |
| Communication | -0.03 | -0.06 | -0.01 | -2.58 | 0.01 |
| SexFemale | 0.70 | 0.66 | 0.74 | 31.88 | 0.00 |
| PTTYPE2England - Disadvantaged | -0.00 | -0.07 | 0.06 | -0.09 | 0.93 |
| PTTYPE2England - Ethnic | -0.00 | -0.09 | 0.09 | -0.03 | 0.98 |
| PTTYPE2Wales - Advantaged | 0.05 | -0.04 | 0.15 | 1.06 | 0.29 |
| PTTYPE2Wales - Disadvantaged | 0.06 | -0.01 | 0.14 | 1.77 | 0.08 |
| PTTYPE2Scotland - Advantaged | 0.12 | 0.04 | 0.20 | 2.91 | 0.00 |
| PTTYPE2Scotland - Disadvantaged | 0.08 | -0.00 | 0.15 | 1.96 | 0.05 |
| PTTYPE2Northern Ireland - Advantaged | -0.10 | -0.20 | -0.00 | -2.04 | 0.04 |
| PTTYPE2Northern Ireland - Disadvantaged | 0.00 | -0.10 | 0.11 | 0.06 | 0.95 |
| EthnicityMixed | -0.10 | -0.24 | 0.04 | -1.37 | 0.17 |
| EthnicityIndian | -0.18 | -0.34 | -0.01 | -2.05 | 0.04 |
| EthnicityPakistani and Bangladeshi | -0.35 | -0.43 | -0.26 | -7.91 | 0.00 |
| EthnicityBlack or Black British | -0.40 | -0.51 | -0.28 | -6.69 | 0.00 |
| EthnicityOther Ethnic group (inc Chinese,Other) | -0.16 | -0.34 | 0.02 | -1.70 | 0.09 |
| CM\_Age | 0.02 | -0.00 | 0.04 | 1.70 | 0.09 |
| Both\_Parents\_in\_Household | -0.02 | -0.09 | 0.06 | -0.41 | 0.68 |
| Siblings | -0.01 | -0.04 | 0.01 | -1.11 | 0.27 |
| Income | -0.01 | -0.04 | 0.02 | -0.54 | 0.59 |
| Maternal\_Education | 0.01 | -0.02 | 0.03 | 0.40 | 0.69 |
| Maternal\_Age | 0.01 | -0.02 | 0.04 | 0.81 | 0.42 |
| Maternal\_Mental\_Health | 0.14 | 0.08 | 0.19 | 5.21 | 0.00 |
| Small\_for\_Gestational\_Age | 0.02 | -0.05 | 0.09 | 0.61 | 0.54 |
|  |
| --- |
| Standard errors: Robust; Continuous variables are mean-centered and scaled by 1 s.d. |

  
   


---

# Structural Regression, no mediators (unimputed)

## Approach

In this preliminary analysis section, we use an SEM regression model
with outcomes (exogenous variables) being the Big 5 dimensions and
exposures (endogenous variables) being the 3 primary variables of
interest (developmental milestones at age 9 months). In the first model
there are no control variables at all. In the second, the control
variables are Sex, exact participant age (in months), Stratum
(neighbourhood deprivation), and Family income (equivalised OECD
quantiles). In the third, fully-adjusted model, we also included
ethnicity, maternal education and age, maternal mental health, as well
as number of sibling and if both natural parents lived in the household.
We follow this approach to better understand the influence of entering
progressively more control variables. Complete cases only are considered
in this case. Note that in the final models of the following sections,
we combine the variable on preterm birth and low birthweight and replace
with the variable “small for gestational age”. Most crucially, in the
present section we do not account for any mediators in these
longitudinal relationships.

  
   


---

## Unadjusted

```
## lavaan 0.6-18 ended normally after 140 iterations
## 
##   Estimator                                         ML
##   Optimization method                           NLMINB
##   Number of model parameters                        35
## 
##   Number of observations                          9191
## 
## Model Test User Model:
##                                               Standard      Scaled
##   Test Statistic                                 0.000       0.000
##   Degrees of freedom                                 0           0
## 
## Model Test Baseline Model:
## 
##   Test statistic                              3434.121    2490.231
##   Degrees of freedom                                25          25
##   P-value                                        0.000       0.000
##   Scaling correction factor                                  1.379
## 
## User Model versus Baseline Model:
## 
##   Comparative Fit Index (CFI)                    1.000       1.000
##   Tucker-Lewis Index (TLI)                       1.000       1.000
##                                                                   
##   Robust Comparative Fit Index (CFI)                            NA
##   Robust Tucker-Lewis Index (TLI)                               NA
## 
## Loglikelihood and Information Criteria:
## 
##   Loglikelihood user model (H0)            -123674.211 -123674.211
##   Loglikelihood unrestricted model (H1)    -123674.211 -123674.211
##                                                                   
##   Akaike (AIC)                              247418.422  247418.422
##   Bayesian (BIC)                            247667.831  247667.831
##   Sample-size adjusted Bayesian (SABIC)     247556.607  247556.607
## 
## Root Mean Square Error of Approximation:
## 
##   RMSEA                                          0.000          NA
##   90 Percent confidence interval - lower         0.000          NA
##   90 Percent confidence interval - upper         0.000          NA
##   P-value H_0: RMSEA <= 0.050                       NA          NA
##   P-value H_0: RMSEA >= 0.080                       NA          NA
##                                                                   
##   Robust RMSEA                                               0.000
##   90 Percent confidence interval - lower                     0.000
##   90 Percent confidence interval - upper                     0.000
##   P-value H_0: Robust RMSEA <= 0.050                            NA
##   P-value H_0: Robust RMSEA >= 0.080                            NA
## 
## Standardized Root Mean Square Residual:
## 
##   SRMR                                           0.000       0.000
## 
## Parameter Estimates:
## 
##   Standard errors                           Robust.sem
##   Information                                 Expected
##   Information saturated (h1) model          Structured
## 
## Regressions:
##                    Estimate  Std.Err  z-value  P(>|z|)   Std.lv  Std.all
##   OPEN ~                                                                
##     Motor_fine        0.092    0.058    1.598    0.110    0.092    0.020
##     Motor_gross       0.120    0.036    3.354    0.001    0.120    0.042
##     Communication     0.038    0.028    1.375    0.169    0.038    0.017
##   CONSC ~                                                               
##     Motor_fine        0.116    0.050    2.317    0.021    0.116    0.029
##     Motor_gross       0.043    0.032    1.328    0.184    0.043    0.017
##     Communication     0.073    0.025    2.925    0.003    0.073    0.038
##   EXTRAV ~                                                              
##     Motor_fine        0.066    0.057    1.162    0.245    0.066    0.014
##     Motor_gross       0.029    0.037    0.788    0.430    0.029    0.010
##     Communication     0.085    0.029    2.895    0.004    0.085    0.037
##   AGREE ~                                                               
##     Motor_fine        0.120    0.046    2.623    0.009    0.120    0.033
##     Motor_gross      -0.049    0.029   -1.699    0.089   -0.049   -0.021
##     Communication     0.082    0.025    3.258    0.001    0.082    0.045
##   NEUROT ~                                                              
##     Motor_fine        0.087    0.066    1.322    0.186    0.087    0.015
##     Motor_gross      -0.203    0.042   -4.848    0.000   -0.203   -0.057
##     Communication     0.073    0.036    2.049    0.040    0.073    0.026
## 
## Covariances:
##                    Estimate  Std.Err  z-value  P(>|z|)   Std.lv  Std.all
##  .OPEN ~~                                                               
##    .CONSC             2.153    0.164   13.151    0.000    2.153    0.176
##    .EXTRAV            2.059    0.188   10.936    0.000    2.059    0.140
##    .AGREE             2.521    0.152   16.612    0.000    2.521    0.221
##    .NEUROT           -0.026    0.208   -0.123    0.902   -0.026   -0.001
##  .CONSC ~~                                                              
##    .EXTRAV            2.265    0.156   14.565    0.000    2.265    0.177
##    .AGREE             3.158    0.141   22.328    0.000    3.158    0.318
##    .NEUROT           -3.593    0.192  -18.734    0.000   -3.593   -0.233
##  .EXTRAV ~~                                                             
##    .AGREE             1.669    0.162   10.276    0.000    1.669    0.139
##    .NEUROT           -5.382    0.225  -23.930    0.000   -5.382   -0.289
##  .AGREE ~~                                                              
##    .NEUROT           -0.112    0.157   -0.713    0.476   -0.112   -0.008
## 
## Intercepts:
##                    Estimate  Std.Err  z-value  P(>|z|)   Std.lv  Std.all
##    .OPEN             12.596    0.459   27.436    0.000   12.596    3.358
##    .CONSC            12.610    0.377   33.416    0.000   12.610    3.861
##    .EXTRAV           12.326    0.464   26.565    0.000   12.326    3.132
##    .AGREE            15.407    0.364   42.291    0.000   15.407    5.051
##    .NEUROT           11.955    0.510   23.455    0.000   11.955    2.519
## 
## Variances:
##                    Estimate  Std.Err  z-value  P(>|z|)   Std.lv  Std.all
##    .OPEN             14.024    0.207   67.728    0.000   14.024    0.997
##    .CONSC            10.626    0.186   57.158    0.000   10.626    0.996
##    .EXTRAV           15.453    0.234   66.051    0.000   15.453    0.998
##    .AGREE             9.272    0.199   46.581    0.000    9.272    0.997
##    .NEUROT           22.454    0.262   85.691    0.000   22.454    0.997
## 
## R-Square:
##                    Estimate
##     OPEN              0.003
##     CONSC             0.004
##     EXTRAV            0.002
##     AGREE             0.003
##     NEUROT            0.003
```

  
   


---

## Adjusted Model

```
## lavaan 0.6-18 ended normally after 149 iterations
## 
##   Estimator                                         ML
##   Optimization method                           NLMINB
##   Number of model parameters                        95
## 
##   Number of observations                          9173
## 
## Model Test User Model:
##                                               Standard      Scaled
##   Test Statistic                                 0.000       0.000
##   Degrees of freedom                                 0           0
## 
## Model Test Baseline Model:
## 
##   Test statistic                              5356.981    4575.353
##   Degrees of freedom                                85          85
##   P-value                                        0.000       0.000
##   Scaling correction factor                                  1.171
## 
## User Model versus Baseline Model:
## 
##   Comparative Fit Index (CFI)                    1.000       1.000
##   Tucker-Lewis Index (TLI)                       1.000       1.000
##                                                                   
##   Robust Comparative Fit Index (CFI)                            NA
##   Robust Tucker-Lewis Index (TLI)                               NA
## 
## Loglikelihood and Information Criteria:
## 
##   Loglikelihood user model (H0)            -122462.304 -122462.304
##   Loglikelihood unrestricted model (H1)    -122462.304 -122462.304
##                                                                   
##   Akaike (AIC)                              245114.609  245114.609
##   Bayesian (BIC)                            245791.390  245791.390
##   Sample-size adjusted Bayesian (SABIC)     245489.496  245489.496
## 
## Root Mean Square Error of Approximation:
## 
##   RMSEA                                          0.000          NA
##   90 Percent confidence interval - lower         0.000          NA
##   90 Percent confidence interval - upper         0.000          NA
##   P-value H_0: RMSEA <= 0.050                       NA          NA
##   P-value H_0: RMSEA >= 0.080                       NA          NA
##                                                                   
##   Robust RMSEA                                               0.000
##   90 Percent confidence interval - lower                     0.000
##   90 Percent confidence interval - upper                     0.000
##   P-value H_0: Robust RMSEA <= 0.050                            NA
##   P-value H_0: Robust RMSEA >= 0.080                            NA
## 
## Standardized Root Mean Square Residual:
## 
##   SRMR                                           0.000       0.000
## 
## Parameter Estimates:
## 
##   Standard errors                           Robust.sem
##   Information                                 Expected
##   Information saturated (h1) model          Structured
## 
## Regressions:
##                    Estimate  Std.Err  z-value  P(>|z|)   Std.lv  Std.all
##   OPEN ~                                                                
##     Motor_fine        0.069    0.057    1.209    0.227    0.069    0.015
##     Motor_gross       0.132    0.037    3.550    0.000    0.132    0.046
##     Communication     0.038    0.028    1.329    0.184    0.038    0.017
##     Sex_Male         -0.214    0.103   -2.084    0.037   -0.214   -0.028
##     CM_Age           -0.043    0.094   -0.462    0.644   -0.043   -0.006
##     Number_of_CMs    -0.080    0.440   -0.183    0.855   -0.080   -0.003
##     Income            0.200    0.038    5.273    0.000    0.200    0.075
##     STRATUM_EN_Dsd    0.160    0.137    1.166    0.244    0.160    0.019
##     STRATUM_EN_Eth    0.685    0.157    4.359    0.000    0.685    0.045
##     STRATUM_NI_Adv    0.615    0.231    2.661    0.008    0.615    0.025
##     STRATUM_NI_Dsd    0.208    0.185    1.123    0.261    0.208    0.007
##     STRATUM_SC_Adv    0.312    0.165    1.886    0.059    0.312    0.019
##     STRATUM_SC_Dsd    0.027    0.229    0.120    0.905    0.027    0.001
##     STRATUM_WA_Adv   -0.106    0.222   -0.478    0.633   -0.106   -0.005
##     STRATUM_WA_Dsd    0.114    0.179    0.640    0.522    0.114    0.004
##   CONSC ~                                                               
##     Motor_fine        0.098    0.050    1.943    0.052    0.098    0.025
##     Motor_gross       0.055    0.032    1.694    0.090    0.055    0.022
##     Communication     0.087    0.026    3.327    0.001    0.087    0.045
##     Sex_Male         -0.084    0.082   -1.024    0.306   -0.084   -0.013
##     CM_Age           -0.074    0.074   -0.996    0.319   -0.074   -0.011
##     Number_of_CMs     0.651    0.405    1.607    0.108    0.651    0.029
##     Income            0.128    0.037    3.468    0.001    0.128    0.055
##     STRATUM_EN_Dsd   -0.122    0.121   -1.009    0.313   -0.122   -0.017
##     STRATUM_EN_Eth   -0.081    0.127   -0.640    0.522   -0.081   -0.006
##     STRATUM_NI_Adv    0.449    0.182    2.462    0.014    0.449    0.021
##     STRATUM_NI_Dsd   -0.110    0.204   -0.539    0.590   -0.110   -0.004
##     STRATUM_SC_Adv   -0.179    0.153   -1.174    0.240   -0.179   -0.013
##     STRATUM_SC_Dsd   -0.087    0.166   -0.520    0.603   -0.087   -0.005
##     STRATUM_WA_Adv    0.070    0.227    0.309    0.758    0.070    0.004
##     STRATUM_WA_Dsd   -0.290    0.142   -2.048    0.041   -0.290   -0.010
##   EXTRAV ~                                                              
##     Motor_fine        0.025    0.057    0.449    0.653    0.025    0.005
##     Motor_gross       0.039    0.037    1.067    0.286    0.039    0.013
##     Communication     0.082    0.030    2.741    0.006    0.082    0.035
##     Sex_Male         -0.283    0.103   -2.744    0.006   -0.283   -0.036
##     CM_Age           -0.001    0.092   -0.016    0.988   -0.001   -0.000
##     Number_of_CMs    -0.607    0.421   -1.441    0.150   -0.607   -0.023
##     Income            0.174    0.033    5.280    0.000    0.174    0.062
##     STRATUM_EN_Dsd   -0.142    0.127   -1.118    0.264   -0.142   -0.016
##     STRATUM_EN_Eth    0.042    0.145    0.288    0.773    0.042    0.003
##     STRATUM_NI_Adv    0.578    0.266    2.172    0.030    0.578    0.023
##     STRATUM_NI_Dsd    0.359    0.214    1.674    0.094    0.359    0.011
##     STRATUM_SC_Adv    0.169    0.211    0.799    0.424    0.169    0.010
##     STRATUM_SC_Dsd    0.065    0.218    0.297    0.767    0.065    0.003
##     STRATUM_WA_Adv   -0.128    0.163   -0.784    0.433   -0.128   -0.006
##     STRATUM_WA_Dsd   -0.100    0.148   -0.674    0.500   -0.100   -0.003
##   AGREE ~                                                               
##     Motor_fine        0.069    0.046    1.502    0.133    0.069    0.019
##     Motor_gross      -0.019    0.029   -0.662    0.508   -0.019   -0.008
##     Communication     0.030    0.026    1.147    0.252    0.030    0.016
##     Sex_Male         -1.031    0.083  -12.362    0.000   -1.031   -0.169
##     CM_Age            0.085    0.076    1.122    0.262    0.085    0.014
##     Number_of_CMs    -0.078    0.351   -0.223    0.823   -0.078   -0.004
##     Income            0.112    0.031    3.652    0.000    0.112    0.051
##     STRATUM_EN_Dsd   -0.019    0.098   -0.190    0.849   -0.019   -0.003
##     STRATUM_EN_Eth    0.275    0.142    1.940    0.052    0.275    0.022
##     STRATUM_NI_Adv    0.360    0.138    2.610    0.009    0.360    0.018
##     STRATUM_NI_Dsd    0.330    0.166    1.983    0.047    0.330    0.013
##     STRATUM_SC_Adv    0.408    0.123    3.303    0.001    0.408    0.031
##     STRATUM_SC_Dsd    0.463    0.141    3.294    0.001    0.463    0.028
##     STRATUM_WA_Adv    0.326    0.152    2.147    0.032    0.326    0.019
##     STRATUM_WA_Dsd    0.193    0.158    1.223    0.221    0.193    0.007
##   NEUROT ~                                                              
##     Motor_fine       -0.047    0.066   -0.716    0.474   -0.047   -0.008
##     Motor_gross      -0.115    0.043   -2.706    0.007   -0.115   -0.032
##     Communication    -0.088    0.037   -2.407    0.016   -0.088   -0.031
##     Sex_Male         -3.338    0.104  -32.141    0.000   -3.338   -0.352
##     CM_Age            0.210    0.124    1.694    0.090    0.210    0.022
##     Number_of_CMs     0.184    0.452    0.408    0.683    0.184    0.006
##     Income           -0.003    0.046   -0.062    0.950   -0.003   -0.001
##     STRATUM_EN_Dsd   -0.174    0.174   -0.999    0.318   -0.174   -0.017
##     STRATUM_EN_Eth   -1.225    0.297   -4.125    0.000   -1.225   -0.064
##     STRATUM_NI_Adv   -0.425    0.242   -1.760    0.078   -0.425   -0.014
##     STRATUM_NI_Dsd    0.114    0.261    0.436    0.663    0.114    0.003
##     STRATUM_SC_Adv    0.576    0.193    2.988    0.003    0.576    0.028
##     STRATUM_SC_Dsd    0.453    0.195    2.326    0.020    0.453    0.018
##     STRATUM_WA_Adv    0.297    0.233    1.273    0.203    0.297    0.011
##     STRATUM_WA_Dsd    0.262    0.192    1.363    0.173    0.262    0.006
## 
## Covariances:
##                    Estimate  Std.Err  z-value  P(>|z|)   Std.lv  Std.all
##  .OPEN ~~                                                               
##    .CONSC             2.099    0.164   12.786    0.000    2.099    0.173
##    .EXTRAV            1.977    0.184   10.716    0.000    1.977    0.135
##    .AGREE             2.427    0.152   15.962    0.000    2.427    0.217
##    .NEUROT           -0.159    0.194   -0.817    0.414   -0.159   -0.010
##  .CONSC ~~                                                              
##    .EXTRAV            2.204    0.155   14.254    0.000    2.204    0.173
##    .AGREE             3.119    0.142   22.040    0.000    3.119    0.320
##    .NEUROT           -3.672    0.181  -20.281    0.000   -3.672   -0.255
##  .EXTRAV ~~                                                             
##    .AGREE             1.561    0.152   10.243    0.000    1.561    0.133
##    .NEUROT           -5.626    0.204  -27.625    0.000   -5.626   -0.324
##  .AGREE ~~                                                              
##    .NEUROT           -0.957    0.149   -6.430    0.000   -0.957   -0.072
## 
## Intercepts:
##                    Estimate  Std.Err  z-value  P(>|z|)   Std.lv  Std.all
##    .OPEN             12.540    1.041   12.050    0.000   12.540    3.342
##    .CONSC            12.286    0.905   13.572    0.000   12.286    3.764
##    .EXTRAV           12.841    1.000   12.844    0.000   12.841    3.264
##    .AGREE            15.332    0.847   18.093    0.000   15.332    5.026
##    .NEUROT           13.131    1.348    9.740    0.000   13.131    2.767
## 
## Variances:
##                    Estimate  Std.Err  z-value  P(>|z|)   Std.lv  Std.all
##    .OPEN             13.931    0.205   67.862    0.000   13.931    0.990
##    .CONSC            10.555    0.188   56.004    0.000   10.555    0.991
##    .EXTRAV           15.334    0.233   65.682    0.000   15.334    0.991
##    .AGREE             8.973    0.193   46.451    0.000    8.973    0.964
##    .NEUROT           19.606    0.265   74.085    0.000   19.606    0.871
## 
## R-Square:
##                    Estimate
##     OPEN              0.010
##     CONSC             0.009
##     EXTRAV            0.009
##     AGREE             0.036
##     NEUROT            0.129
```

  
   


---

## Fully Adjusted

```
## lavaan 0.6-18 ended normally after 146 iterations
## 
##   Estimator                                         ML
##   Optimization method                           NLMINB
##   Number of model parameters                       145
## 
##   Number of observations                          8803
## 
## Model Test User Model:
##                                               Standard      Scaled
##   Test Statistic                                 0.000       0.000
##   Degrees of freedom                                 0           0
## 
## Model Test Baseline Model:
## 
##   Test statistic                              5439.981    4546.951
##   Degrees of freedom                               135         135
##   P-value                                        0.000       0.000
##   Scaling correction factor                                  1.196
## 
## User Model versus Baseline Model:
## 
##   Comparative Fit Index (CFI)                    1.000       1.000
##   Tucker-Lewis Index (TLI)                       1.000       1.000
##                                                                   
##   Robust Comparative Fit Index (CFI)                            NA
##   Robust Tucker-Lewis Index (TLI)                               NA
## 
## Loglikelihood and Information Criteria:
## 
##   Loglikelihood user model (H0)            -117382.045 -117382.045
##   Loglikelihood unrestricted model (H1)    -117382.045 -117382.045
##                                                                   
##   Akaike (AIC)                              235054.091  235054.091
##   Bayesian (BIC)                            236081.103  236081.103
##   Sample-size adjusted Bayesian (SABIC)     235620.319  235620.319
## 
## Root Mean Square Error of Approximation:
## 
##   RMSEA                                          0.000          NA
##   90 Percent confidence interval - lower         0.000          NA
##   90 Percent confidence interval - upper         0.000          NA
##   P-value H_0: RMSEA <= 0.050                       NA          NA
##   P-value H_0: RMSEA >= 0.080                       NA          NA
##                                                                   
##   Robust RMSEA                                               0.000
##   90 Percent confidence interval - lower                     0.000
##   90 Percent confidence interval - upper                     0.000
##   P-value H_0: Robust RMSEA <= 0.050                            NA
##   P-value H_0: Robust RMSEA >= 0.080                            NA
## 
## Standardized Root Mean Square Residual:
## 
##   SRMR                                           0.000       0.000
## 
## Parameter Estimates:
## 
##   Standard errors                           Robust.sem
##   Information                                 Expected
##   Information saturated (h1) model          Structured
## 
## Regressions:
##                    Estimate  Std.Err  z-value  P(>|z|)   Std.lv  Std.all
##   OPEN ~                                                                
##     Motor_fine        0.033    0.059    0.556    0.578    0.033    0.007
##     Motor_gross       0.126    0.037    3.384    0.001    0.126    0.044
##     Communication     0.058    0.029    2.009    0.044    0.058    0.026
##     Sex_Male         -0.198    0.103   -1.929    0.054   -0.198   -0.026
##     CM_Age           -0.045    0.097   -0.461    0.645   -0.045   -0.006
##     Number_of_CMs    -0.224    0.441   -0.508    0.612   -0.224   -0.009
##     Income            0.064    0.045    1.435    0.151    0.064    0.024
##     STRATUM_EN_Dsd    0.163    0.139    1.173    0.241    0.163    0.020
##     STRATUM_EN_Eth    0.281    0.187    1.504    0.133    0.281    0.018
##     STRATUM_NI_Adv    0.599    0.225    2.661    0.008    0.599    0.025
##     STRATUM_NI_Dsd    0.362    0.191    1.894    0.058    0.362    0.011
##     STRATUM_SC_Adv    0.236    0.167    1.417    0.157    0.236    0.015
##     STRATUM_SC_Dsd    0.104    0.229    0.455    0.649    0.104    0.005
##     STRATUM_WA_Adv   -0.142    0.225   -0.634    0.526   -0.142   -0.007
##     STRATUM_WA_Dsd    0.216    0.177    1.221    0.222    0.216    0.007
##     Ethnicity_Blck    0.807    0.256    3.157    0.002    0.807    0.035
##     Ethnicity_Indn    0.113    0.281    0.403    0.687    0.113    0.004
##     Ethnicity_Mixd    0.507    0.295    1.721    0.085    0.507    0.023
##     Ethnicity_Othr    0.540    0.443    1.219    0.223    0.540    0.014
##     Ethnicity_PaBn    0.829    0.220    3.772    0.000    0.829    0.046
##     Maternal_Edctn    0.258    0.040    6.375    0.000    0.258    0.096
##     Maternal_Age      0.026    0.009    3.010    0.003    0.026    0.039
##     Mtrnl_Mntl_Hlt    0.264    0.104    2.537    0.011    0.264    0.030
##     Preterm_Birth    -0.160    0.239   -0.670    0.503   -0.160   -0.011
##     Low_Birthweght    0.173    0.272    0.634    0.526    0.173    0.012
##   CONSC ~                                                               
##     Motor_fine        0.080    0.052    1.530    0.126    0.080    0.020
##     Motor_gross       0.050    0.033    1.516    0.130    0.050    0.020
##     Communication     0.093    0.027    3.446    0.001    0.093    0.048
##     Sex_Male         -0.112    0.084   -1.326    0.185   -0.112   -0.017
##     CM_Age           -0.062    0.077   -0.814    0.416   -0.062   -0.009
##     Number_of_CMs     0.736    0.388    1.896    0.058    0.736    0.033
##     Income            0.107    0.041    2.586    0.010    0.107    0.046
##     STRATUM_EN_Dsd   -0.159    0.126   -1.263    0.207   -0.159   -0.022
##     STRATUM_EN_Eth   -0.337    0.163   -2.066    0.039   -0.337   -0.025
##     STRATUM_NI_Adv    0.457    0.186    2.452    0.014    0.457    0.022
##     STRATUM_NI_Dsd   -0.053    0.202   -0.262    0.793   -0.053   -0.002
##     STRATUM_SC_Adv   -0.212    0.154   -1.374    0.169   -0.212   -0.015
##     STRATUM_SC_Dsd   -0.118    0.165   -0.712    0.477   -0.118   -0.007
##     STRATUM_WA_Adv    0.060    0.232    0.260    0.795    0.060    0.003
##     STRATUM_WA_Dsd   -0.301    0.144   -2.089    0.037   -0.301   -0.011
##     Ethnicity_Blck    0.221    0.301    0.732    0.464    0.221    0.011
##     Ethnicity_Indn    0.214    0.274    0.780    0.435    0.214    0.009
##     Ethnicity_Mixd   -0.654    0.235   -2.782    0.005   -0.654   -0.034
##     Ethnicity_Othr    0.230    0.218    1.055    0.291    0.230    0.007
##     Ethnicity_PaBn    0.579    0.180    3.218    0.001    0.579    0.037
##     Maternal_Edctn    0.093    0.035    2.663    0.008    0.093    0.039
##     Maternal_Age     -0.014    0.007   -1.911    0.056   -0.014   -0.024
##     Mtrnl_Mntl_Hlt   -0.161    0.094   -1.705    0.088   -0.161   -0.021
##     Preterm_Birth    -0.139    0.217   -0.641    0.522   -0.139   -0.010
##     Low_Birthweght   -0.057    0.229   -0.247    0.805   -0.057   -0.004
##   EXTRAV ~                                                              
##     Motor_fine        0.001    0.058    0.019    0.985    0.001    0.000
##     Motor_gross       0.052    0.038    1.373    0.170    0.052    0.017
##     Communication     0.067    0.031    2.172    0.030    0.067    0.029
##     Sex_Male         -0.299    0.103   -2.915    0.004   -0.299   -0.038
##     CM_Age            0.010    0.094    0.112    0.911    0.010    0.001
##     Number_of_CMs    -0.550    0.435   -1.264    0.206   -0.550   -0.021
##     Income            0.193    0.046    4.165    0.000    0.193    0.068
##     STRATUM_EN_Dsd   -0.190    0.132   -1.437    0.151   -0.190   -0.022
##     STRATUM_EN_Eth   -0.299    0.202   -1.486    0.137   -0.299   -0.018
##     STRATUM_NI_Adv    0.581    0.271    2.141    0.032    0.581    0.023
##     STRATUM_NI_Dsd    0.374    0.217    1.723    0.085    0.374    0.011
##     STRATUM_SC_Adv    0.162    0.211    0.770    0.441    0.162    0.010
##     STRATUM_SC_Dsd    0.055    0.212    0.261    0.794    0.055    0.003
##     STRATUM_WA_Adv   -0.129    0.164   -0.782    0.434   -0.129   -0.006
##     STRATUM_WA_Dsd   -0.158    0.147   -1.077    0.281   -0.158   -0.005
##     Ethnicity_Blck   -0.296    0.299   -0.989    0.323   -0.296   -0.012
##     Ethnicity_Indn    0.598    0.376    1.588    0.112    0.598    0.021
##     Ethnicity_Mixd   -0.285    0.338   -0.843    0.399   -0.285   -0.012
##     Ethnicity_Othr   -0.328    0.332   -0.988    0.323   -0.328   -0.008
##     Ethnicity_PaBn    0.681    0.226    3.016    0.003    0.681    0.036
##     Maternal_Edctn   -0.009    0.042   -0.219    0.826   -0.009   -0.003
##     Maternal_Age     -0.018    0.010   -1.794    0.073   -0.018   -0.026
##     Mtrnl_Mntl_Hlt   -0.492    0.122   -4.019    0.000   -0.492   -0.053
##     Preterm_Birth     0.128    0.233    0.548    0.584    0.128    0.008
##     Low_Birthweght   -0.327    0.225   -1.450    0.147   -0.327   -0.021
##   AGREE ~                                                               
##     Motor_fine        0.056    0.047    1.199    0.231    0.056    0.015
##     Motor_gross       0.002    0.031    0.065    0.948    0.002    0.001
##     Communication     0.035    0.026    1.326    0.185    0.035    0.019
##     Sex_Male         -1.064    0.084  -12.623    0.000   -1.064   -0.175
##     CM_Age            0.069    0.077    0.890    0.373    0.069    0.011
##     Number_of_CMs    -0.105    0.349   -0.301    0.763   -0.105   -0.005
##     Income            0.070    0.036    1.956    0.050    0.070    0.032
##     STRATUM_EN_Dsd   -0.035    0.100   -0.351    0.726   -0.035   -0.005
##     STRATUM_EN_Eth    0.104    0.168    0.619    0.536    0.104    0.008
##     STRATUM_NI_Adv    0.335    0.137    2.450    0.014    0.335    0.017
##     STRATUM_NI_Dsd    0.345    0.172    2.009    0.045    0.345    0.013
##     STRATUM_SC_Adv    0.382    0.125    3.062    0.002    0.382    0.030
##     STRATUM_SC_Dsd    0.467    0.147    3.182    0.001    0.467    0.029
##     STRATUM_WA_Adv    0.311    0.154    2.016    0.044    0.311    0.019
##     STRATUM_WA_Dsd    0.219    0.159    1.376    0.169    0.219    0.008
##     Ethnicity_Blck   -0.398    0.265   -1.502    0.133   -0.398   -0.021
##     Ethnicity_Indn    0.284    0.236    1.205    0.228    0.284    0.013
##     Ethnicity_Mixd   -0.364    0.230   -1.586    0.113   -0.364   -0.020
##     Ethnicity_Othr    0.244    0.246    0.991    0.322    0.244    0.008
##     Ethnicity_PaBn    0.595    0.237    2.513    0.012    0.595    0.041
##     Maternal_Edctn    0.057    0.034    1.678    0.093    0.057    0.026
##     Maternal_Age      0.010    0.007    1.565    0.118    0.010    0.019
##     Mtrnl_Mntl_Hlt   -0.104    0.097   -1.069    0.285   -0.104   -0.014
##     Preterm_Birth     0.069    0.195    0.356    0.722    0.069    0.006
##     Low_Birthweght    0.076    0.187    0.405    0.685    0.076    0.006
##   NEUROT ~                                                              
##     Motor_fine       -0.048    0.068   -0.696    0.487   -0.048   -0.008
##     Motor_gross      -0.094    0.044   -2.155    0.031   -0.094   -0.026
##     Communication    -0.082    0.037   -2.180    0.029   -0.082   -0.029
##     Sex_Male         -3.322    0.103  -32.101    0.000   -3.322   -0.349
##     CM_Age            0.192    0.126    1.527    0.127    0.192    0.020
##     Number_of_CMs     0.081    0.472    0.172    0.864    0.081    0.003
##     Income           -0.019    0.057   -0.326    0.744   -0.019   -0.005
##     STRATUM_EN_Dsd   -0.012    0.172   -0.068    0.946   -0.012   -0.001
##     STRATUM_EN_Eth   -0.010    0.364   -0.028    0.978   -0.010   -0.001
##     STRATUM_NI_Adv   -0.489    0.247   -1.977    0.048   -0.489   -0.016
##     STRATUM_NI_Dsd    0.006    0.271    0.022    0.983    0.006    0.000
##     STRATUM_SC_Adv    0.545    0.195    2.795    0.005    0.545    0.027
##     STRATUM_SC_Dsd    0.370    0.201    1.841    0.066    0.370    0.015
##     STRATUM_WA_Adv    0.241    0.239    1.011    0.312    0.241    0.009
##     STRATUM_WA_Dsd    0.294    0.179    1.643    0.100    0.294    0.007
##     Ethnicity_Blck   -1.874    0.486   -3.854    0.000   -1.874   -0.064
##     Ethnicity_Indn   -0.838    0.463   -1.811    0.070   -0.838   -0.024
##     Ethnicity_Mixd   -0.455    0.355   -1.283    0.200   -0.455   -0.016
##     Ethnicity_Othr   -0.735    0.524   -1.403    0.161   -0.735   -0.015
##     Ethnicity_PaBn   -1.679    0.377   -4.459    0.000   -1.679   -0.074
##     Maternal_Edctn    0.026    0.049    0.523    0.601    0.026    0.007
##     Maternal_Age      0.004    0.013    0.326    0.745    0.004    0.005
##     Mtrnl_Mntl_Hlt    0.635    0.131    4.850    0.000    0.635    0.057
##     Preterm_Birth     0.213    0.324    0.658    0.511    0.213    0.011
##     Low_Birthweght    0.181    0.322    0.561    0.575    0.181    0.010
## 
## Covariances:
##                    Estimate  Std.Err  z-value  P(>|z|)   Std.lv  Std.all
##  .OPEN ~~                                                               
##    .CONSC             2.012    0.165   12.185    0.000    2.012    0.168
##    .EXTRAV            1.949    0.186   10.486    0.000    1.949    0.134
##    .AGREE             2.357    0.151   15.650    0.000    2.357    0.213
##    .NEUROT           -0.085    0.200   -0.424    0.672   -0.085   -0.005
##  .CONSC ~~                                                              
##    .EXTRAV            2.170    0.148   14.706    0.000    2.170    0.171
##    .AGREE             3.049    0.142   21.531    0.000    3.049    0.315
##    .NEUROT           -3.659    0.185  -19.802    0.000   -3.659   -0.256
##  .EXTRAV ~~                                                             
##    .AGREE             1.546    0.152   10.202    0.000    1.546    0.132
##    .NEUROT           -5.606    0.203  -27.670    0.000   -5.606   -0.325
##  .AGREE ~~                                                              
##    .NEUROT           -0.936    0.153   -6.136    0.000   -0.936   -0.071
## 
## Intercepts:
##                    Estimate  Std.Err  z-value  P(>|z|)   Std.lv  Std.all
##    .OPEN             11.444    1.082   10.574    0.000   11.444    3.055
##    .CONSC            12.436    0.928   13.402    0.000   12.436    3.810
##    .EXTRAV           13.548    1.010   13.415    0.000   13.548    3.435
##    .AGREE            15.098    0.886   17.046    0.000   15.098    4.955
##    .NEUROT           12.957    1.415    9.156    0.000   12.957    2.727
## 
## Variances:
##                    Estimate  Std.Err  z-value  P(>|z|)   Std.lv  Std.all
##    .OPEN             13.723    0.206   66.750    0.000   13.723    0.978
##    .CONSC            10.495    0.194   54.221    0.000   10.495    0.985
##    .EXTRAV           15.323    0.229   66.830    0.000   15.323    0.985
##    .AGREE             8.905    0.197   45.179    0.000    8.905    0.959
##    .NEUROT           19.446    0.269   72.351    0.000   19.446    0.862
## 
## R-Square:
##                    Estimate
##     OPEN              0.022
##     CONSC             0.015
##     EXTRAV            0.015
##     AGREE             0.041
##     NEUROT            0.138
```

```
##    lhs op        rhs est.std    se     z pvalue ci.lower ci.upper
## 1 OPEN  ~ Motor_fine   0.007 0.013 0.556  0.578   -0.018    0.032
```

```
##    lhs op         rhs est.std    se     z pvalue ci.lower ci.upper
## 2 OPEN  ~ Motor_gross   0.044 0.013 3.395  0.001    0.019     0.07
```

```
##    lhs op           rhs est.std    se     z pvalue ci.lower ci.upper
## 3 OPEN  ~ Communication   0.026 0.013 2.008  0.045    0.001    0.051
```

```
##      lhs op        rhs est.std    se     z pvalue ci.lower ci.upper
## 26 CONSC  ~ Motor_fine    0.02 0.013 1.533  0.125   -0.006    0.046
```

```
##      lhs op         rhs est.std    se     z pvalue ci.lower ci.upper
## 27 CONSC  ~ Motor_gross    0.02 0.013 1.518  0.129   -0.006    0.046
```

```
##      lhs op           rhs est.std    se     z pvalue ci.lower ci.upper
## 28 CONSC  ~ Communication   0.048 0.014 3.444  0.001    0.021    0.075
```

```
##       lhs op        rhs est.std    se     z pvalue ci.lower ci.upper
## 51 EXTRAV  ~ Motor_fine       0 0.012 0.019  0.985   -0.024    0.024
```

```
##       lhs op         rhs est.std    se     z pvalue ci.lower ci.upper
## 52 EXTRAV  ~ Motor_gross   0.017 0.013 1.373   0.17   -0.007    0.042
```

```
##       lhs op           rhs est.std    se     z pvalue ci.lower ci.upper
## 53 EXTRAV  ~ Communication   0.029 0.013 2.173   0.03    0.003    0.055
```

```
##      lhs op        rhs est.std    se     z pvalue ci.lower ci.upper
## 76 AGREE  ~ Motor_fine   0.015 0.013 1.199   0.23    -0.01     0.04
```

```
##      lhs op         rhs est.std    se     z pvalue ci.lower ci.upper
## 77 AGREE  ~ Motor_gross   0.001 0.013 0.065  0.948   -0.025    0.027
```

```
##      lhs op           rhs est.std    se     z pvalue ci.lower ci.upper
## 78 AGREE  ~ Communication   0.019 0.015 1.325  0.185   -0.009    0.048
```

```
##        lhs op        rhs est.std    se      z pvalue ci.lower ci.upper
## 101 NEUROT  ~ Motor_fine  -0.008 0.012 -0.696  0.486   -0.031    0.015
```

```
##        lhs op         rhs est.std    se      z pvalue ci.lower ci.upper
## 102 NEUROT  ~ Motor_gross  -0.026 0.012 -2.154  0.031    -0.05   -0.002
```

```
##        lhs op           rhs est.std    se     z pvalue ci.lower ci.upper
## 103 NEUROT  ~ Communication  -0.029 0.013 -2.18  0.029   -0.055   -0.003
```

  
   


---

## Results Table

|  |  |  |  |
| --- | --- | --- | --- |
|  | Model 1 | Model 2 | Model 3 |
|  | Estimate (Std. Err.) | Estimate (Std. Err.) | Estimate (Std. Err.) |
|  | Regression Slopes | | |
| OPEN |
| Motor.fine | 0.09(0.06) | 0.07(0.06) | 0.03(0.06) |
| Motor.gross | 0.12(0.04)\*\*\* | 0.13(0.04)\*\*\* | 0.13(0.04)\*\*\* |
| Communication | 0.04(0.03) | 0.04(0.03) | 0.06(0.03)\* |
| Sex: Male |  | -0.21(0.10)\* | -0.20(0.10) |
| Cohort member’s age (in months) |  | -0.04(0.09) | -0.04(0.10) |
| Number of cohort members |  | -0.08(0.44) | -0.22(0.44) |
| Income |  | 0.20(0.04)\*\*\* | 0.06(0.04) |
| England - Disadvantaged |  | 0.16(0.14) | 0.16(0.14) |
| England - Ethnic |  | 0.69(0.16)\*\*\* | 0.28(0.19) |
| Northern Ireland - Advantaged |  | 0.61(0.23)\*\* | 0.60(0.22)\*\* |
| Northern Ireland - Disadvantaged |  | 0.21(0.19) | 0.36(0.19) |
| Scotland - Advantaged |  | 0.31(0.17) | 0.24(0.17) |
| Scotland - Disadvantaged |  | 0.03(0.23) | 0.10(0.23) |
| Wales - Advantaged |  | -0.11(0.22) | -0.14(0.22) |
| Wales - Disadvantaged |  | 0.11(0.18) | 0.22(0.18) |
| Black or Black British |  |  | 0.81(0.26)\*\* |
| Indian |  |  | 0.11(0.28) |
| Mixed |  |  | 0.51(0.29) |
| Other ethnic group |  |  | 0.54(0.44) |
| Pakistani & Bangladeshi |  |  | 0.83(0.22)\*\*\* |
| Maternal education |  |  | 0.26(0.04)\*\*\* |
| Maternal.Age |  |  | 0.03(0.01)\*\* |
| Maternal mental health |  |  | 0.26(0.10)\* |
| Preterm.Birth |  |  | -0.16(0.24) |
| Low.Birthweight |  |  | 0.17(0.27) |
| CONSC |
| Motor.fine | 0.12(0.05)\* | 0.10(0.05) | 0.08(0.05) |
| Motor.gross | 0.04(0.03) | 0.05(0.03) | 0.05(0.03) |
| Communication | 0.07(0.02)\*\* | 0.09(0.03)\*\*\* | 0.09(0.03)\*\*\* |
| Sex: Male |  | -0.08(0.08) | -0.11(0.08) |
| Cohort member’s age (in months) |  | -0.07(0.07) | -0.06(0.08) |
| Number of cohort members |  | 0.65(0.40) | 0.74(0.39) |
| Income |  | 0.13(0.04)\*\*\* | 0.11(0.04)\*\* |
| England - Disadvantaged |  | -0.12(0.12) | -0.16(0.13) |
| England - Ethnic |  | -0.08(0.13) | -0.34(0.16)\* |
| Northern Ireland - Advantaged |  | 0.45(0.18)\* | 0.46(0.19)\* |
| Northern Ireland - Disadvantaged |  | -0.11(0.20) | -0.05(0.20) |
| Scotland - Advantaged |  | -0.18(0.15) | -0.21(0.15) |
| Scotland - Disadvantaged |  | -0.09(0.17) | -0.12(0.17) |
| Wales - Advantaged |  | 0.07(0.23) | 0.06(0.23) |
| Wales - Disadvantaged |  | -0.29(0.14)\* | -0.30(0.14)\* |
| Black or Black British |  |  | 0.22(0.30) |
| Indian |  |  | 0.21(0.27) |
| Mixed |  |  | -0.65(0.24)\*\* |
| Other ethnic group |  |  | 0.23(0.22) |
| Pakistani & Bangladeshi |  |  | 0.58(0.18)\*\* |
| Maternal education |  |  | 0.09(0.03)\*\* |
| Maternal.Age |  |  | -0.01(0.01) |
| Maternal mental health |  |  | -0.16(0.09) |
| Preterm.Birth |  |  | -0.14(0.22) |
| Low.Birthweight |  |  | -0.06(0.23) |
| EXTRAV |
| Motor.fine | 0.07(0.06) | 0.03(0.06) | 0.00(0.06) |
| Motor.gross | 0.03(0.04) | 0.04(0.04) | 0.05(0.04) |
| Communication | 0.09(0.03)\*\* | 0.08(0.03)\*\* | 0.07(0.03)\* |
| Sex: Male |  | -0.28(0.10)\*\* | -0.30(0.10)\*\* |
| Cohort member’s age (in months) |  | -0.00(0.09) | 0.01(0.09) |
| Number of cohort members |  | -0.61(0.42) | -0.55(0.43) |
| Income |  | 0.17(0.03)\*\*\* | 0.19(0.05)\*\*\* |
| England - Disadvantaged |  | -0.14(0.13) | -0.19(0.13) |
| England - Ethnic |  | 0.04(0.15) | -0.30(0.20) |
| Northern Ireland - Advantaged |  | 0.58(0.27)\* | 0.58(0.27)\* |
| Northern Ireland - Disadvantaged |  | 0.36(0.21) | 0.37(0.22) |
| Scotland - Advantaged |  | 0.17(0.21) | 0.16(0.21) |
| Scotland - Disadvantaged |  | 0.06(0.22) | 0.06(0.21) |
| Wales - Advantaged |  | -0.13(0.16) | -0.13(0.16) |
| Wales - Disadvantaged |  | -0.10(0.15) | -0.16(0.15) |
| Black or Black British |  |  | -0.30(0.30) |
| Indian |  |  | 0.60(0.38) |
| Mixed |  |  | -0.29(0.34) |
| Other ethnic group |  |  | -0.33(0.33) |
| Pakistani & Bangladeshi |  |  | 0.68(0.23)\*\* |
| Maternal education |  |  | -0.01(0.04) |
| Maternal.Age |  |  | -0.02(0.01) |
| Maternal mental health |  |  | -0.49(0.12)\*\*\* |
| Preterm.Birth |  |  | 0.13(0.23) |
| Low.Birthweight |  |  | -0.33(0.23) |
| AGREE |
| Motor.fine | 0.12(0.05)\*\* | 0.07(0.05) | 0.06(0.05) |
| Motor.gross | -0.05(0.03) | -0.02(0.03) | 0.00(0.03) |
| Communication | 0.08(0.03)\*\* | 0.03(0.03) | 0.03(0.03) |
| Sex: Male |  | -1.03(0.08)\*\*\* | -1.06(0.08)\*\*\* |
| Cohort member’s age (in months) |  | 0.08(0.08) | 0.07(0.08) |
| Number of cohort members |  | -0.08(0.35) | -0.11(0.35) |
| Income |  | 0.11(0.03)\*\*\* | 0.07(0.04) |
| England - Disadvantaged |  | -0.02(0.10) | -0.04(0.10) |
| England - Ethnic |  | 0.28(0.14) | 0.10(0.17) |
| Northern Ireland - Advantaged |  | 0.36(0.14)\*\* | 0.33(0.14)\* |
| Northern Ireland - Disadvantaged |  | 0.33(0.17)\* | 0.35(0.17)\* |
| Scotland - Advantaged |  | 0.41(0.12)\*\*\* | 0.38(0.12)\*\* |
| Scotland - Disadvantaged |  | 0.46(0.14)\*\*\* | 0.47(0.15)\*\* |
| Wales - Advantaged |  | 0.33(0.15)\* | 0.31(0.15)\* |
| Wales - Disadvantaged |  | 0.19(0.16) | 0.22(0.16) |
| Black or Black British |  |  | -0.40(0.26) |
| Indian |  |  | 0.28(0.24) |
| Mixed |  |  | -0.36(0.23) |
| Other ethnic group |  |  | 0.24(0.25) |
| Pakistani & Bangladeshi |  |  | 0.59(0.24)\* |
| Maternal education |  |  | 0.06(0.03) |
| Maternal.Age |  |  | 0.01(0.01) |
| Maternal mental health |  |  | -0.10(0.10) |
| Preterm.Birth |  |  | 0.07(0.19) |
| Low.Birthweight |  |  | 0.08(0.19) |
| NEUROT |
| Motor.fine | 0.09(0.07) | -0.05(0.07) | -0.05(0.07) |
| Motor.gross | -0.20(0.04)\*\*\* | -0.12(0.04)\*\* | -0.09(0.04)\* |
| Communication | 0.07(0.04)\* | -0.09(0.04)\* | -0.08(0.04)\* |
| Sex: Male |  | -3.34(0.10)\*\*\* | -3.32(0.10)\*\*\* |
| Cohort member’s age (in months) |  | 0.21(0.12) | 0.19(0.13) |
| Number of cohort members |  | 0.18(0.45) | 0.08(0.47) |
| Income |  | -0.00(0.05) | -0.02(0.06) |
| England - Disadvantaged |  | -0.17(0.17) | -0.01(0.17) |
| England - Ethnic |  | -1.22(0.30)\*\*\* | -0.01(0.36) |
| Northern Ireland - Advantaged |  | -0.43(0.24) | -0.49(0.25)\* |
| Northern Ireland - Disadvantaged |  | 0.11(0.26) | 0.01(0.27) |
| Scotland - Advantaged |  | 0.58(0.19)\*\* | 0.54(0.19)\*\* |
| Scotland - Disadvantaged |  | 0.45(0.19)\* | 0.37(0.20) |
| Wales - Advantaged |  | 0.30(0.23) | 0.24(0.24) |
| Wales - Disadvantaged |  | 0.26(0.19) | 0.29(0.18) |
| Black or Black British |  |  | -1.87(0.49)\*\*\* |
| Indian |  |  | -0.84(0.46) |
| Mixed |  |  | -0.46(0.36) |
| Other ethnic group |  |  | -0.74(0.52) |
| Pakistani & Bangladeshi |  |  | -1.68(0.38)\*\*\* |
| Maternal education |  |  | 0.03(0.05) |
| Maternal.Age |  |  | 0.00(0.01) |
| Maternal mental health |  |  | 0.64(0.13)\*\*\* |
| Preterm.Birth |  |  | 0.21(0.32) |
| Low.Birthweight |  |  | 0.18(0.32) |
|  | Intercepts | | |
| OPEN | 12.60(0.46)\*\*\* | 12.54(1.04)\*\*\* | 11.44(1.08)\*\*\* |
| CONSC | 12.61(0.38)\*\*\* | 12.29(0.91)\*\*\* | 12.44(0.93)\*\*\* |
| EXTRAV | 12.33(0.46)\*\*\* | 12.84(1.00)\*\*\* | 13.55(1.01)\*\*\* |
| AGREE | 15.41(0.36)\*\*\* | 15.33(0.85)\*\*\* | 15.10(0.89)\*\*\* |
| NEUROT | 11.96(0.51)\*\*\* | 13.13(1.35)\*\*\* | 12.96(1.42)\*\*\* |
| Motor.fine | 7.58+ | 7.58+ | 7.59+ |
| Motor.gross | 5.50+ | 5.50+ | 5.50+ |
| Communication | 6.51+ | 6.51+ | 6.51+ |
| Sex: Male |  | 0.49+ | 0.48+ |
| Cohort member’s age (in months) |  | 9.18+ | 9.18+ |
| Number of cohort members |  | 1.02+ | 1.02+ |
| Income |  | 3.16+ | 3.19+ |
| England - Disadvantaged |  | 0.29+ | 0.28+ |
| England - Ethnic |  | 0.07+ | 0.06+ |
| Northern Ireland - Advantaged |  | 0.02+ | 0.02+ |
| Northern Ireland - Disadvantaged |  | 0.01+ | 0.01+ |
| Scotland - Advantaged |  | 0.06+ | 0.06+ |
| Scotland - Disadvantaged |  | 0.04+ | 0.04+ |
| Wales - Advantaged |  | 0.03+ | 0.03+ |
| Wales - Disadvantaged |  | 0.01+ | 0.01+ |
| Black or Black British |  |  | 0.03+ |
| Indian |  |  | 0.02+ |
| Mixed |  |  | 0.03+ |
| Other ethnic group |  |  | 0.01+ |
| Pakistani & Bangladeshi |  |  | 0.05+ |
| Maternal education |  |  | 3.70+ |
| Maternal.Age |  |  | 30.22+ |
| Maternal mental health |  |  | 0.23+ |
| Preterm.Birth |  |  | 0.06+ |
| Low.Birthweight |  |  | 0.07+ |
|  | Fit Indices | | |
| χ2 | 0.00 | 0.00 | 0.00 |
| CFI | 1.00 | 1.00 | 1.00 |
| TLI | 1.00 | 1.00 | 1.00 |
| RMSEA | 0.00 | 0.00 | 0.00 |
| Scaled χ2 | 0.00(0) | 0.00(0) | 0.00(0) |
| +Fixed parameter | | | |
| \*p<0.05, \*\*p<0.01, \*\*\*p<0.001 | | | |

|  |  |  |  |
| --- | --- | --- | --- |
|  | Model 1 | Model 2 | Model 3 |
|  | Estimate (Std. Err.) | Estimate (Std. Err.) | Estimate (Std. Err.) |
|  | Regression Slopes | | |
| OPEN |
| Motor.fine | 0.09(0.06) | 0.07(0.06) | 0.03(0.06) |
| Motor.gross | 0.12(0.04)\*\*\* | 0.13(0.04)\*\*\* | 0.13(0.04)\*\*\* |
| Communication | 0.04(0.03) | 0.04(0.03) | 0.06(0.03)\* |
| Sex: Male |  | -0.21(0.10)\* | -0.20(0.10) |
| Cohort member’s age (in months) |  | -0.04(0.09) | -0.04(0.10) |
| Number of cohort members |  | -0.08(0.44) | -0.22(0.44) |
| Income |  | 0.20(0.04)\*\*\* | 0.06(0.04) |
| England - Disadvantaged |  | 0.16(0.14) | 0.16(0.14) |
| England - Ethnic |  | 0.69(0.16)\*\*\* | 0.28(0.19) |
| Northern Ireland - Advantaged |  | 0.61(0.23)\*\* | 0.60(0.22)\*\* |
| Northern Ireland - Disadvantaged |  | 0.21(0.19) | 0.36(0.19) |
| Scotland - Advantaged |  | 0.31(0.17) | 0.24(0.17) |
| Scotland - Disadvantaged |  | 0.03(0.23) | 0.10(0.23) |
| Wales - Advantaged |  | -0.11(0.22) | -0.14(0.22) |
| Wales - Disadvantaged |  | 0.11(0.18) | 0.22(0.18) |
| Black or Black British |  |  | 0.81(0.26)\*\* |
| Indian |  |  | 0.11(0.28) |
| Mixed |  |  | 0.51(0.29) |
| Other ethnic group |  |  | 0.54(0.44) |
| Pakistani & Bangladeshi |  |  | 0.83(0.22)\*\*\* |
| Maternal education |  |  | 0.26(0.04)\*\*\* |
| Maternal.Age |  |  | 0.03(0.01)\*\* |
| Maternal mental health |  |  | 0.26(0.10)\* |
| Preterm.Birth |  |  | -0.16(0.24) |
| Low.Birthweight |  |  | 0.17(0.27) |
| CONSC |
| Motor.fine | 0.12(0.05)\* | 0.10(0.05) | 0.08(0.05) |
| Motor.gross | 0.04(0.03) | 0.05(0.03) | 0.05(0.03) |
| Communication | 0.07(0.02)\*\* | 0.09(0.03)\*\*\* | 0.09(0.03)\*\*\* |
| Sex: Male |  | -0.08(0.08) | -0.11(0.08) |
| Cohort member’s age (in months) |  | -0.07(0.07) | -0.06(0.08) |
| Number of cohort members |  | 0.65(0.40) | 0.74(0.39) |
| Income |  | 0.13(0.04)\*\*\* | 0.11(0.04)\*\* |
| England - Disadvantaged |  | -0.12(0.12) | -0.16(0.13) |
| England - Ethnic |  | -0.08(0.13) | -0.34(0.16)\* |
| Northern Ireland - Advantaged |  | 0.45(0.18)\* | 0.46(0.19)\* |
| Northern Ireland - Disadvantaged |  | -0.11(0.20) | -0.05(0.20) |
| Scotland - Advantaged |  | -0.18(0.15) | -0.21(0.15) |
| Scotland - Disadvantaged |  | -0.09(0.17) | -0.12(0.17) |
| Wales - Advantaged |  | 0.07(0.23) | 0.06(0.23) |
| Wales - Disadvantaged |  | -0.29(0.14)\* | -0.30(0.14)\* |
| Black or Black British |  |  | 0.22(0.30) |
| Indian |  |  | 0.21(0.27) |
| Mixed |  |  | -0.65(0.24)\*\* |
| Other ethnic group |  |  | 0.23(0.22) |
| Pakistani & Bangladeshi |  |  | 0.58(0.18)\*\* |
| Maternal education |  |  | 0.09(0.03)\*\* |
| Maternal.Age |  |  | -0.01(0.01) |
| Maternal mental health |  |  | -0.16(0.09) |
| Preterm.Birth |  |  | -0.14(0.22) |
| Low.Birthweight |  |  | -0.06(0.23) |
| EXTRAV |
| Motor.fine | 0.07(0.06) | 0.03(0.06) | 0.00(0.06) |
| Motor.gross | 0.03(0.04) | 0.04(0.04) | 0.05(0.04) |
| Communication | 0.09(0.03)\*\* | 0.08(0.03)\*\* | 0.07(0.03)\* |
| Sex: Male |  | -0.28(0.10)\*\* | -0.30(0.10)\*\* |
| Cohort member’s age (in months) |  | -0.00(0.09) | 0.01(0.09) |
| Number of cohort members |  | -0.61(0.42) | -0.55(0.43) |
| Income |  | 0.17(0.03)\*\*\* | 0.19(0.05)\*\*\* |
| England - Disadvantaged |  | -0.14(0.13) | -0.19(0.13) |
| England - Ethnic |  | 0.04(0.15) | -0.30(0.20) |
| Northern Ireland - Advantaged |  | 0.58(0.27)\* | 0.58(0.27)\* |
| Northern Ireland - Disadvantaged |  | 0.36(0.21) | 0.37(0.22) |
| Scotland - Advantaged |  | 0.17(0.21) | 0.16(0.21) |
| Scotland - Disadvantaged |  | 0.06(0.22) | 0.06(0.21) |
| Wales - Advantaged |  | -0.13(0.16) | -0.13(0.16) |
| Wales - Disadvantaged |  | -0.10(0.15) | -0.16(0.15) |
| Black or Black British |  |  | -0.30(0.30) |
| Indian |  |  | 0.60(0.38) |
| Mixed |  |  | -0.29(0.34) |
| Other ethnic group |  |  | -0.33(0.33) |
| Pakistani & Bangladeshi |  |  | 0.68(0.23)\*\* |
| Maternal education |  |  | -0.01(0.04) |
| Maternal.Age |  |  | -0.02(0.01) |
| Maternal mental health |  |  | -0.49(0.12)\*\*\* |
| Preterm.Birth |  |  | 0.13(0.23) |
| Low.Birthweight |  |  | -0.33(0.23) |
| AGREE |
| Motor.fine | 0.12(0.05)\*\* | 0.07(0.05) | 0.06(0.05) |
| Motor.gross | -0.05(0.03) | -0.02(0.03) | 0.00(0.03) |
| Communication | 0.08(0.03)\*\* | 0.03(0.03) | 0.03(0.03) |
| Sex: Male |  | -1.03(0.08)\*\*\* | -1.06(0.08)\*\*\* |
| Cohort member’s age (in months) |  | 0.08(0.08) | 0.07(0.08) |
| Number of cohort members |  | -0.08(0.35) | -0.11(0.35) |
| Income |  | 0.11(0.03)\*\*\* | 0.07(0.04) |
| England - Disadvantaged |  | -0.02(0.10) | -0.04(0.10) |
| England - Ethnic |  | 0.28(0.14) | 0.10(0.17) |
| Northern Ireland - Advantaged |  | 0.36(0.14)\*\* | 0.33(0.14)\* |
| Northern Ireland - Disadvantaged |  | 0.33(0.17)\* | 0.35(0.17)\* |
| Scotland - Advantaged |  | 0.41(0.12)\*\*\* | 0.38(0.12)\*\* |
| Scotland - Disadvantaged |  | 0.46(0.14)\*\*\* | 0.47(0.15)\*\* |
| Wales - Advantaged |  | 0.33(0.15)\* | 0.31(0.15)\* |
| Wales - Disadvantaged |  | 0.19(0.16) | 0.22(0.16) |
| Black or Black British |  |  | -0.40(0.26) |
| Indian |  |  | 0.28(0.24) |
| Mixed |  |  | -0.36(0.23) |
| Other ethnic group |  |  | 0.24(0.25) |
| Pakistani & Bangladeshi |  |  | 0.59(0.24)\* |
| Maternal education |  |  | 0.06(0.03) |
| Maternal.Age |  |  | 0.01(0.01) |
| Maternal mental health |  |  | -0.10(0.10) |
| Preterm.Birth |  |  | 0.07(0.19) |
| Low.Birthweight |  |  | 0.08(0.19) |
| NEUROT |
| Motor.fine | 0.09(0.07) | -0.05(0.07) | -0.05(0.07) |
| Motor.gross | -0.20(0.04)\*\*\* | -0.12(0.04)\*\* | -0.09(0.04)\* |
| Communication | 0.07(0.04)\* | -0.09(0.04)\* | -0.08(0.04)\* |
| Sex: Male |  | -3.34(0.10)\*\*\* | -3.32(0.10)\*\*\* |
| Cohort member’s age (in months) |  | 0.21(0.12) | 0.19(0.13) |
| Number of cohort members |  | 0.18(0.45) | 0.08(0.47) |
| Income |  | -0.00(0.05) | -0.02(0.06) |
| England - Disadvantaged |  | -0.17(0.17) | -0.01(0.17) |
| England - Ethnic |  | -1.22(0.30)\*\*\* | -0.01(0.36) |
| Northern Ireland - Advantaged |  | -0.43(0.24) | -0.49(0.25)\* |
| Northern Ireland - Disadvantaged |  | 0.11(0.26) | 0.01(0.27) |
| Scotland - Advantaged |  | 0.58(0.19)\*\* | 0.54(0.19)\*\* |
| Scotland - Disadvantaged |  | 0.45(0.19)\* | 0.37(0.20) |
| Wales - Advantaged |  | 0.30(0.23) | 0.24(0.24) |
| Wales - Disadvantaged |  | 0.26(0.19) | 0.29(0.18) |
| Black or Black British |  |  | -1.87(0.49)\*\*\* |
| Indian |  |  | -0.84(0.46) |
| Mixed |  |  | -0.46(0.36) |
| Other ethnic group |  |  | -0.74(0.52) |
| Pakistani & Bangladeshi |  |  | -1.68(0.38)\*\*\* |
| Maternal education |  |  | 0.03(0.05) |
| Maternal.Age |  |  | 0.00(0.01) |
| Maternal mental health |  |  | 0.64(0.13)\*\*\* |
| Preterm.Birth |  |  | 0.21(0.32) |
| Low.Birthweight |  |  | 0.18(0.32) |
|  | Intercepts | | |
| OPEN | 12.60(0.46)\*\*\* | 12.54(1.04)\*\*\* | 11.44(1.08)\*\*\* |
| CONSC | 12.61(0.38)\*\*\* | 12.29(0.91)\*\*\* | 12.44(0.93)\*\*\* |
| EXTRAV | 12.33(0.46)\*\*\* | 12.84(1.00)\*\*\* | 13.55(1.01)\*\*\* |
| AGREE | 15.41(0.36)\*\*\* | 15.33(0.85)\*\*\* | 15.10(0.89)\*\*\* |
| NEUROT | 11.96(0.51)\*\*\* | 13.13(1.35)\*\*\* | 12.96(1.42)\*\*\* |
| Motor.fine | 7.58+ | 7.58+ | 7.59+ |
| Motor.gross | 5.50+ | 5.50+ | 5.50+ |
| Communication | 6.51+ | 6.51+ | 6.51+ |
| Sex: Male |  | 0.49+ | 0.48+ |
| Cohort member’s age (in months) |  | 9.18+ | 9.18+ |
| Number of cohort members |  | 1.02+ | 1.02+ |
| Income |  | 3.16+ | 3.19+ |
| England - Disadvantaged |  | 0.29+ | 0.28+ |
| England - Ethnic |  | 0.07+ | 0.06+ |
| Northern Ireland - Advantaged |  | 0.02+ | 0.02+ |
| Northern Ireland - Disadvantaged |  | 0.01+ | 0.01+ |
| Scotland - Advantaged |  | 0.06+ | 0.06+ |
| Scotland - Disadvantaged |  | 0.04+ | 0.04+ |
| Wales - Advantaged |  | 0.03+ | 0.03+ |
| Wales - Disadvantaged |  | 0.01+ | 0.01+ |
| Black or Black British |  |  | 0.03+ |
| Indian |  |  | 0.02+ |
| Mixed |  |  | 0.03+ |
| Other ethnic group |  |  | 0.01+ |
| Pakistani & Bangladeshi |  |  | 0.05+ |
| Maternal education |  |  | 3.70+ |
| Maternal.Age |  |  | 30.22+ |
| Maternal mental health |  |  | 0.23+ |
| Preterm.Birth |  |  | 0.06+ |
| Low.Birthweight |  |  | 0.07+ |
|  | Fit Indices | | |
| χ2 | 0.00 | 0.00 | 0.00 |
| CFI | 1.00 | 1.00 | 1.00 |
| TLI | 1.00 | 1.00 | 1.00 |
| RMSEA | 0.00 | 0.00 | 0.00 |
| Scaled χ2 | 0.00(0) | 0.00(0) | 0.00(0) |
| +Fixed parameter | | | |
| \*p<0.05, \*\*p<0.01, \*\*\*p<0.001 | | | |

  

|  |  |  |  |
| --- | --- | --- | --- |
|  | Model 1 | Model 2 | Model 3 |
|  | Estimate (Std. Err.) | Estimate (Std. Err.) | Estimate (Std. Err.) |
|  | Regression Slopes | | |
| OPEN |
| Motor.fine | 0.09(0.06) | 0.07(0.06) | 0.03(0.06) |
| Motor.gross | 0.12(0.04)\*\*\* | 0.13(0.04)\*\*\* | 0.13(0.04)\*\*\* |
| Communication | 0.04(0.03) | 0.04(0.03) | 0.06(0.03)\* |
| Sex: Male |  | -0.21(0.10)\* | -0.20(0.10) |
| Cohort member’s age (in months) |  | -0.04(0.09) | -0.04(0.10) |
| Number of cohort members |  | -0.08(0.44) | -0.22(0.44) |
| Income |  | 0.20(0.04)\*\*\* | 0.06(0.04) |
| England - Disadvantaged |  | 0.16(0.14) | 0.16(0.14) |
| England - Ethnic |  | 0.69(0.16)\*\*\* | 0.28(0.19) |
| Northern Ireland - Advantaged |  | 0.61(0.23)\*\* | 0.60(0.22)\*\* |
| Northern Ireland - Disadvantaged |  | 0.21(0.19) | 0.36(0.19) |
| Scotland - Advantaged |  | 0.31(0.17) | 0.24(0.17) |
| Scotland - Disadvantaged |  | 0.03(0.23) | 0.10(0.23) |
| Wales - Advantaged |  | -0.11(0.22) | -0.14(0.22) |
| Wales - Disadvantaged |  | 0.11(0.18) | 0.22(0.18) |
| Black or Black British |  |  | 0.81(0.26)\*\* |
| Indian |  |  | 0.11(0.28) |
| Mixed |  |  | 0.51(0.29) |
| Other ethnic group |  |  | 0.54(0.44) |
| Pakistani & Bangladeshi |  |  | 0.83(0.22)\*\*\* |
| Maternal education |  |  | 0.26(0.04)\*\*\* |
| Maternal.Age |  |  | 0.03(0.01)\*\* |
| Maternal mental health |  |  | 0.26(0.10)\* |
| Preterm.Birth |  |  | -0.16(0.24) |
| Low.Birthweight |  |  | 0.17(0.27) |
| CONSC |
| Motor.fine | 0.12(0.05)\* | 0.10(0.05) | 0.08(0.05) |
| Motor.gross | 0.04(0.03) | 0.05(0.03) | 0.05(0.03) |
| Communication | 0.07(0.02)\*\* | 0.09(0.03)\*\*\* | 0.09(0.03)\*\*\* |
| Sex: Male |  | -0.08(0.08) | -0.11(0.08) |
| Cohort member’s age (in months) |  | -0.07(0.07) | -0.06(0.08) |
| Number of cohort members |  | 0.65(0.40) | 0.74(0.39) |
| Income |  | 0.13(0.04)\*\*\* | 0.11(0.04)\*\* |
| England - Disadvantaged |  | -0.12(0.12) | -0.16(0.13) |
| England - Ethnic |  | -0.08(0.13) | -0.34(0.16)\* |
| Northern Ireland - Advantaged |  | 0.45(0.18)\* | 0.46(0.19)\* |
| Northern Ireland - Disadvantaged |  | -0.11(0.20) | -0.05(0.20) |
| Scotland - Advantaged |  | -0.18(0.15) | -0.21(0.15) |
| Scotland - Disadvantaged |  | -0.09(0.17) | -0.12(0.17) |
| Wales - Advantaged |  | 0.07(0.23) | 0.06(0.23) |
| Wales - Disadvantaged |  | -0.29(0.14)\* | -0.30(0.14)\* |
| Black or Black British |  |  | 0.22(0.30) |
| Indian |  |  | 0.21(0.27) |
| Mixed |  |  | -0.65(0.24)\*\* |
| Other ethnic group |  |  | 0.23(0.22) |
| Pakistani & Bangladeshi |  |  | 0.58(0.18)\*\* |
| Maternal education |  |  | 0.09(0.03)\*\* |
| Maternal.Age |  |  | -0.01(0.01) |
| Maternal mental health |  |  | -0.16(0.09) |
| Preterm.Birth |  |  | -0.14(0.22) |
| Low.Birthweight |  |  | -0.06(0.23) |
| EXTRAV |
| Motor.fine | 0.07(0.06) | 0.03(0.06) | 0.00(0.06) |
| Motor.gross | 0.03(0.04) | 0.04(0.04) | 0.05(0.04) |
| Communication | 0.09(0.03)\*\* | 0.08(0.03)\*\* | 0.07(0.03)\* |
| Sex: Male |  | -0.28(0.10)\*\* | -0.30(0.10)\*\* |
| Cohort member’s age (in months) |  | -0.00(0.09) | 0.01(0.09) |
| Number of cohort members |  | -0.61(0.42) | -0.55(0.43) |
| Income |  | 0.17(0.03)\*\*\* | 0.19(0.05)\*\*\* |
| England - Disadvantaged |  | -0.14(0.13) | -0.19(0.13) |
| England - Ethnic |  | 0.04(0.15) | -0.30(0.20) |
| Northern Ireland - Advantaged |  | 0.58(0.27)\* | 0.58(0.27)\* |
| Northern Ireland - Disadvantaged |  | 0.36(0.21) | 0.37(0.22) |
| Scotland - Advantaged |  | 0.17(0.21) | 0.16(0.21) |
| Scotland - Disadvantaged |  | 0.06(0.22) | 0.06(0.21) |
| Wales - Advantaged |  | -0.13(0.16) | -0.13(0.16) |
| Wales - Disadvantaged |  | -0.10(0.15) | -0.16(0.15) |
| Black or Black British |  |  | -0.30(0.30) |
| Indian |  |  | 0.60(0.38) |
| Mixed |  |  | -0.29(0.34) |
| Other ethnic group |  |  | -0.33(0.33) |
| Pakistani & Bangladeshi |  |  | 0.68(0.23)\*\* |
| Maternal education |  |  | -0.01(0.04) |
| Maternal.Age |  |  | -0.02(0.01) |
| Maternal mental health |  |  | -0.49(0.12)\*\*\* |
| Preterm.Birth |  |  | 0.13(0.23) |
| Low.Birthweight |  |  | -0.33(0.23) |
| AGREE |
| Motor.fine | 0.12(0.05)\*\* | 0.07(0.05) | 0.06(0.05) |
| Motor.gross | -0.05(0.03) | -0.02(0.03) | 0.00(0.03) |
| Communication | 0.08(0.03)\*\* | 0.03(0.03) | 0.03(0.03) |
| Sex: Male |  | -1.03(0.08)\*\*\* | -1.06(0.08)\*\*\* |
| Cohort member’s age (in months) |  | 0.08(0.08) | 0.07(0.08) |
| Number of cohort members |  | -0.08(0.35) | -0.11(0.35) |
| Income |  | 0.11(0.03)\*\*\* | 0.07(0.04) |
| England - Disadvantaged |  | -0.02(0.10) | -0.04(0.10) |
| England - Ethnic |  | 0.28(0.14) | 0.10(0.17) |
| Northern Ireland - Advantaged |  | 0.36(0.14)\*\* | 0.33(0.14)\* |
| Northern Ireland - Disadvantaged |  | 0.33(0.17)\* | 0.35(0.17)\* |
| Scotland - Advantaged |  | 0.41(0.12)\*\*\* | 0.38(0.12)\*\* |
| Scotland - Disadvantaged |  | 0.46(0.14)\*\*\* | 0.47(0.15)\*\* |
| Wales - Advantaged |  | 0.33(0.15)\* | 0.31(0.15)\* |
| Wales - Disadvantaged |  | 0.19(0.16) | 0.22(0.16) |
| Black or Black British |  |  | -0.40(0.26) |
| Indian |  |  | 0.28(0.24) |
| Mixed |  |  | -0.36(0.23) |
| Other ethnic group |  |  | 0.24(0.25) |
| Pakistani & Bangladeshi |  |  | 0.59(0.24)\* |
| Maternal education |  |  | 0.06(0.03) |
| Maternal.Age |  |  | 0.01(0.01) |
| Maternal mental health |  |  | -0.10(0.10) |
| Preterm.Birth |  |  | 0.07(0.19) |
| Low.Birthweight |  |  | 0.08(0.19) |
| NEUROT |
| Motor.fine | 0.09(0.07) | -0.05(0.07) | -0.05(0.07) |
| Motor.gross | -0.20(0.04)\*\*\* | -0.12(0.04)\*\* | -0.09(0.04)\* |
| Communication | 0.07(0.04)\* | -0.09(0.04)\* | -0.08(0.04)\* |
| Sex: Male |  | -3.34(0.10)\*\*\* | -3.32(0.10)\*\*\* |
| Cohort member’s age (in months) |  | 0.21(0.12) | 0.19(0.13) |
| Number of cohort members |  | 0.18(0.45) | 0.08(0.47) |
| Income |  | -0.00(0.05) | -0.02(0.06) |
| England - Disadvantaged |  | -0.17(0.17) | -0.01(0.17) |
| England - Ethnic |  | -1.22(0.30)\*\*\* | -0.01(0.36) |
| Northern Ireland - Advantaged |  | -0.43(0.24) | -0.49(0.25)\* |
| Northern Ireland - Disadvantaged |  | 0.11(0.26) | 0.01(0.27) |
| Scotland - Advantaged |  | 0.58(0.19)\*\* | 0.54(0.19)\*\* |
| Scotland - Disadvantaged |  | 0.45(0.19)\* | 0.37(0.20) |
| Wales - Advantaged |  | 0.30(0.23) | 0.24(0.24) |
| Wales - Disadvantaged |  | 0.26(0.19) | 0.29(0.18) |
| Black or Black British |  |  | -1.87(0.49)\*\*\* |
| Indian |  |  | -0.84(0.46) |
| Mixed |  |  | -0.46(0.36) |
| Other ethnic group |  |  | -0.74(0.52) |
| Pakistani & Bangladeshi |  |  | -1.68(0.38)\*\*\* |
| Maternal education |  |  | 0.03(0.05) |
| Maternal.Age |  |  | 0.00(0.01) |
| Maternal mental health |  |  | 0.64(0.13)\*\*\* |
| Preterm.Birth |  |  | 0.21(0.32) |
| Low.Birthweight |  |  | 0.18(0.32) |
|  | Intercepts | | |
| OPEN | 12.60(0.46)\*\*\* | 12.54(1.04)\*\*\* | 11.44(1.08)\*\*\* |
| CONSC | 12.61(0.38)\*\*\* | 12.29(0.91)\*\*\* | 12.44(0.93)\*\*\* |
| EXTRAV | 12.33(0.46)\*\*\* | 12.84(1.00)\*\*\* | 13.55(1.01)\*\*\* |
| AGREE | 15.41(0.36)\*\*\* | 15.33(0.85)\*\*\* | 15.10(0.89)\*\*\* |
| NEUROT | 11.96(0.51)\*\*\* | 13.13(1.35)\*\*\* | 12.96(1.42)\*\*\* |
| Motor.fine | 7.58+ | 7.58+ | 7.59+ |
| Motor.gross | 5.50+ | 5.50+ | 5.50+ |
| Communication | 6.51+ | 6.51+ | 6.51+ |
| Sex: Male |  | 0.49+ | 0.48+ |
| Cohort member’s age (in months) |  | 9.18+ | 9.18+ |
| Number of cohort members |  | 1.02+ | 1.02+ |
| Income |  | 3.16+ | 3.19+ |
| England - Disadvantaged |  | 0.29+ | 0.28+ |
| England - Ethnic |  | 0.07+ | 0.06+ |
| Northern Ireland - Advantaged |  | 0.02+ | 0.02+ |
| Northern Ireland - Disadvantaged |  | 0.01+ | 0.01+ |
| Scotland - Advantaged |  | 0.06+ | 0.06+ |
| Scotland - Disadvantaged |  | 0.04+ | 0.04+ |
| Wales - Advantaged |  | 0.03+ | 0.03+ |
| Wales - Disadvantaged |  | 0.01+ | 0.01+ |
| Black or Black British |  |  | 0.03+ |
| Indian |  |  | 0.02+ |
| Mixed |  |  | 0.03+ |
| Other ethnic group |  |  | 0.01+ |
| Pakistani & Bangladeshi |  |  | 0.05+ |
| Maternal education |  |  | 3.70+ |
| Maternal.Age |  |  | 30.22+ |
| Maternal mental health |  |  | 0.23+ |
| Preterm.Birth |  |  | 0.06+ |
| Low.Birthweight |  |  | 0.07+ |
|  | Fit Indices | | |
| χ2 | 0.00 | 0.00 | 0.00 |
| CFI | 1.00 | 1.00 | 1.00 |
| TLI | 1.00 | 1.00 | 1.00 |
| RMSEA | 0.00 | 0.00 | 0.00 |
| Scaled χ2 | 0.00(0) | 0.00(0) | 0.00(0) |
| +Fixed parameter | | | |
| \*p<0.05, \*\*p<0.01, \*\*\*p<0.001 | | | |

# Structural Regression w/ Mediators (unimputed)

## Approach

In this preliminary analysis section, we use an SEM regression model
with outcomes (exogenous variables) being the Big 5 dimensions and
exposures (endogenous variables) being the 3 primary variables of
interest (developmental milestones at age 9 months). In the first model
there are no control variables at all. In the second, the control
variables are Sex, exact participant age (in months), Stratum
(neighbourhood deprivation), and Family income (equivalised OECD
quantiles). In the third, fully-adjusted model, we also included
ethnicity, maternal education and age, maternal mental health, as well
as number of sibling and if both natural parents lived in the household.
We follow this approach to better understand the influence of entering
progressively more control variables. Complete cases only are considered
in this case. Note that in the final models of the following sections,
we combine the variable on preterm birth and low birthweight and replace
with the variable “small for gestational age”. Most crucially, in the
present section we account for mediators in these longitudinal
relationships.

  
   


---

## Model 1 (unadjusted)

```
## lavaan 0.6-18 ended normally after 406 iterations
## 
##   Estimator                                         ML
##   Optimization method                           NLMINB
##   Number of model parameters                       102
## 
##   Number of observations                          8375
## 
## Model Test User Model:
##                                               Standard      Scaled
##   Test Statistic                               571.763     449.660
##   Degrees of freedom                                31          31
##   P-value (Chi-square)                           0.000       0.000
##   Scaling correction factor                                  1.272
##     Satorra-Bentler correction                                    
## 
## Model Test Baseline Model:
## 
##   Test statistic                              9719.871    6838.552
##   Degrees of freedom                               105         105
##   P-value                                        0.000       0.000
##   Scaling correction factor                                  1.421
## 
## User Model versus Baseline Model:
## 
##   Comparative Fit Index (CFI)                    0.944       0.938
##   Tucker-Lewis Index (TLI)                       0.810       0.789
##                                                                   
##   Robust Comparative Fit Index (CFI)                         0.944
##   Robust Tucker-Lewis Index (TLI)                            0.812
## 
## Loglikelihood and Information Criteria:
## 
##   Loglikelihood user model (H0)            -279740.460 -279740.460
##   Loglikelihood unrestricted model (H1)    -279454.578 -279454.578
##                                                                   
##   Akaike (AIC)                              559684.920  559684.920
##   Bayesian (BIC)                            560402.286  560402.286
##   Sample-size adjusted Bayesian (SABIC)     560078.149  560078.149
## 
## Root Mean Square Error of Approximation:
## 
##   RMSEA                                          0.046       0.040
##   90 Percent confidence interval - lower         0.042       0.037
##   90 Percent confidence interval - upper         0.049       0.043
##   P-value H_0: RMSEA <= 0.050                    0.985       1.000
##   P-value H_0: RMSEA >= 0.080                    0.000       0.000
##                                                                   
##   Robust RMSEA                                               0.045
##   90 Percent confidence interval - lower                     0.042
##   90 Percent confidence interval - upper                     0.049
##   P-value H_0: Robust RMSEA <= 0.050                         0.981
##   P-value H_0: Robust RMSEA >= 0.080                         0.000
## 
## Standardized Root Mean Square Residual:
## 
##   SRMR                                           0.025       0.025
## 
## Parameter Estimates:
## 
##   Standard errors                           Robust.sem
##   Information                                 Expected
##   Information saturated (h1) model          Structured
## 
## Latent Variables:
##                             Estimate  Std.Err  z-value  P(>|z|) ci.lower
##   Cognitive_ability_age5 =~                                             
##     Verbal_blty_g5             1.000                               1.000
##     Spatil_blty_g5             0.819    0.057   14.462    0.000    0.708
##   Self_regulation_age5 =~                                               
##     Independenc_g5             1.000                               1.000
##     Emotion_rgl_g5             1.161    0.047   24.447    0.000    1.068
##  ci.upper   Std.lv  Std.all
##                            
##     1.000    6.370    0.597
##     0.930    5.218    0.543
##                            
##     1.000    1.944    0.533
##     1.254    2.257    0.479
## 
## Regressions:
##                            Estimate  Std.Err  z-value  P(>|z|) ci.lower
##   ToM_age5 ~                                                           
##     Motr_fn   (fT)           -0.007    0.006   -1.202    0.229   -0.019
##     Mtr_grs   (gT)           -0.002    0.003   -0.480    0.631   -0.008
##     Cmmnctn   (cT)            0.007    0.003    2.404    0.016    0.001
##     CM_Age                   -0.004    0.009   -0.391    0.696   -0.022
##   Prosociality_age5 ~                                                  
##     Motr_fn   (fP)            0.190    0.030    6.315    0.000    0.131
##     Mtr_grs   (gP)           -0.015    0.017   -0.909    0.363   -0.047
##     Cmmnctn   (cP)            0.139    0.014   10.192    0.000    0.112
##     CM_Age                   -0.102    0.052   -1.971    0.049   -0.203
##   Cognitive_ability_age5 ~                                             
##     Motr_fn   (fC)            0.725    0.164    4.409    0.000    0.402
##     Mtr_grs   (gC)            0.340    0.100    3.394    0.001    0.143
##     Cmmnctn   (cC)            0.035    0.083    0.427    0.669   -0.127
##     CM_Age                    0.643    0.263    2.439    0.015    0.126
##   Self_regulation_age5 ~                                               
##     Motr_fn   (fS)            0.334    0.048    6.999    0.000    0.240
##     Mtr_grs   (gS)            0.036    0.030    1.218    0.223   -0.022
##     Cmmnctn   (cS)            0.161    0.025    6.420    0.000    0.112
##     CM_Age                   -0.039    0.079   -0.489    0.625   -0.195
##   OPEN ~                                                               
##     Motr_fn  (F_O)            0.001    0.061    0.019    0.985   -0.119
##     Mtr_grs  (G_O)            0.077    0.037    2.065    0.039    0.004
##     Cmmnctn  (C_O)            0.014    0.031    0.461    0.645   -0.046
##     ToM_ag5  (T_O)           -0.208    0.134   -1.554    0.120   -0.471
##     Prscl_5  (P_O)           -0.056    0.068   -0.813    0.416   -0.190
##     Cgnt__5 (Cg_O)            0.073    0.020    3.705    0.000    0.034
##     Slf_r_5  (S_O)            0.140    0.098    1.435    0.151   -0.051
##   CONSC ~                                                              
##     Motr_fn  (F_C)            0.003    0.053    0.061    0.952   -0.102
##     Mtr_grs  (G_C)            0.025    0.034    0.738    0.460   -0.042
##     Cmmnctn  (C_C)            0.041    0.029    1.426    0.154   -0.015
##     ToM_ag5  (T_C)            0.312    0.111    2.820    0.005    0.095
##     Prscl_5  (P_C)           -0.054    0.063   -0.859    0.390   -0.177
##     Cgnt__5 (Cg_C)            0.005    0.015    0.331    0.740   -0.025
##     Slf_r_5  (S_C)            0.302    0.078    3.900    0.000    0.150
##   EXTRAV ~                                                             
##     Motr_fn  (F_E)            0.033    0.065    0.516    0.606   -0.093
##     Mtr_grs  (G_E)            0.040    0.040    0.983    0.326   -0.039
##     Cmmnctn  (C_E)            0.063    0.032    1.942    0.052   -0.001
##     ToM_ag5  (T_E)            0.384    0.140    2.741    0.006    0.109
##     Prscl_5  (P_E)            0.022    0.071    0.312    0.755   -0.117
##     Cgnt__5 (Cg_E)           -0.018    0.019   -0.945    0.345   -0.055
##     Slf_r_5  (S_E)            0.131    0.101    1.288    0.198   -0.068
##   AGREE ~                                                              
##     Motr_fn  (F_A)            0.042    0.053    0.801    0.423   -0.061
##     Mtr_grs  (G_A)           -0.044    0.033   -1.326    0.185   -0.109
##     Cmmnctn  (C_A)            0.030    0.027    1.087    0.277   -0.024
##     ToM_ag5  (T_A)            0.205    0.109    1.891    0.059   -0.008
##     Prscl_5  (P_A)           -0.031    0.061   -0.499    0.618   -0.150
##     Cgnt__5 (Cg_A)           -0.046    0.017   -2.787    0.005   -0.079
##     Slf_r_5  (S_A)            0.335    0.080    4.168    0.000    0.177
##   NEUROT ~                                                             
##     Motr_fn  (F_N)            0.138    0.069    1.995    0.046    0.002
##     Mtr_grs  (G_N)           -0.245    0.047   -5.216    0.000   -0.338
##     Cmmnctn  (C_N)            0.114    0.038    3.011    0.003    0.040
##     ToM_ag5  (T_N)            0.146    0.181    0.804    0.421   -0.210
##     Prscl_5  (P_N)            0.362    0.090    4.004    0.000    0.185
##     Cgnt__5 (Cg_N)            0.091    0.024    3.827    0.000    0.045
##     Slf_r_5  (S_N)           -0.472    0.122   -3.856    0.000   -0.711
##  ci.upper   Std.lv  Std.all
##                            
##     0.005   -0.007   -0.016
##     0.005   -0.002   -0.006
##     0.012    0.007    0.031
##     0.015   -0.004   -0.005
##                            
##     0.249    0.190    0.095
##     0.017   -0.015   -0.012
##     0.165    0.139    0.143
##    -0.001   -0.102   -0.030
##                            
##     1.047    0.114    0.093
##     0.536    0.053    0.070
##     0.198    0.006    0.009
##     1.159    0.101    0.049
##                            
##     0.427    0.172    0.140
##     0.094    0.019    0.024
##     0.211    0.083    0.140
##     0.117   -0.020   -0.010
##                            
##     0.121    0.001    0.000
##     0.151    0.077    0.027
##     0.075    0.014    0.006
##     0.054   -0.208   -0.020
##     0.079   -0.056   -0.024
##     0.112    0.466    0.125
##     0.331    0.272    0.073
##                            
##     0.108    0.003    0.001
##     0.092    0.025    0.010
##     0.097    0.041    0.021
##     0.529    0.312    0.035
##     0.069   -0.054   -0.027
##     0.035    0.032    0.010
##     0.454    0.588    0.180
##                            
##     0.160    0.033    0.007
##     0.119    0.040    0.013
##     0.126    0.063    0.027
##     0.659    0.384    0.035
##     0.161    0.022    0.009
##     0.019   -0.114   -0.029
##     0.329    0.254    0.064
##                            
##     0.146    0.042    0.011
##     0.021   -0.044   -0.019
##     0.083    0.030    0.016
##     0.418    0.205    0.025
##     0.089   -0.031   -0.016
##    -0.014   -0.295   -0.097
##     0.492    0.651    0.214
##                            
##     0.273    0.138    0.024
##    -0.153   -0.245   -0.067
##     0.189    0.114    0.040
##     0.501    0.146    0.011
##     0.539    0.362    0.124
##     0.138    0.581    0.122
##    -0.232   -0.917   -0.192
## 
## Covariances:
##                             Estimate  Std.Err  z-value  P(>|z|) ci.lower
##  .ToM_age5 ~~                                                           
##    .Prosocialty_g5             0.024    0.007    3.543    0.000    0.011
##  .Cognitive_ability_age5 ~~                                             
##    .ToM_age5                   0.375    0.040    9.306    0.000    0.296
##  .Self_regulation_age5 ~~                                               
##    .ToM_age5                   0.063    0.014    4.434    0.000    0.035
##  .Cognitive_ability_age5 ~~                                             
##    .Prosocialty_g5             1.273    0.210    6.049    0.000    0.860
##  .Self_regulation_age5 ~~                                               
##    .Prosocialty_g5             1.893    0.079   23.855    0.000    1.737
##  .Cognitive_ability_age5 ~~                                             
##    .Self_regltn_g5             6.118    0.458   13.345    0.000    5.219
##   Motor_fine ~~                                                         
##     Motor_gross                0.201    0.019   10.636    0.000    0.164
##     Communication              0.361    0.023   15.692    0.000    0.316
##   Motor_gross ~~                                                        
##     Communication              0.704    0.032   21.813    0.000    0.641
##  .OPEN ~~                                                               
##    .CONSC                      1.823    0.173   10.540    0.000    1.484
##    .EXTRAV                     1.949    0.197    9.921    0.000    1.564
##    .AGREE                      2.355    0.163   14.410    0.000    2.035
##    .NEUROT                    -0.012    0.229   -0.054    0.957   -0.461
##  .CONSC ~~                                                              
##    .EXTRAV                     2.116    0.168   12.557    0.000    1.785
##    .AGREE                      2.882    0.155   18.623    0.000    2.579
##    .NEUROT                    -3.415    0.200  -17.080    0.000   -3.807
##  .EXTRAV ~~                                                             
##    .AGREE                      1.502    0.170    8.813    0.000    1.168
##    .NEUROT                    -5.488    0.244  -22.521    0.000   -5.965
##  .AGREE ~~                                                              
##    .NEUROT                     0.134    0.181    0.741    0.459   -0.221
##  ci.upper   Std.lv  Std.all
##                            
##     0.037    0.024    0.041
##                            
##     0.453    0.059    0.163
##                            
##     0.090    0.033    0.091
##                            
##     1.685    0.202    0.126
##                            
##     2.048    1.001    0.625
##                            
##     7.016    0.513    0.513
##                            
##     0.238    0.201    0.189
##     0.406    0.361    0.264
##                            
##     0.768    0.704    0.321
##                            
##     2.162    1.823    0.155
##     2.335    1.949    0.135
##     2.676    2.355    0.214
##     0.436   -0.012   -0.001
##                            
##     2.446    2.116    0.167
##     3.185    2.882    0.300
##    -3.023   -3.415   -0.226
##                            
##     1.836    1.502    0.127
##    -5.010   -5.488   -0.296
##                            
##     0.490    0.134    0.010
## 
## Intercepts:
##                    Estimate  Std.Err  z-value  P(>|z|) ci.lower ci.upper
##    .Verbal_blty_g5   42.426    2.777   15.276    0.000   36.983   47.870
##    .Spatil_blty_g5   40.496    2.355   17.192    0.000   35.879   45.113
##    .Independenc_g5   13.033    0.871   14.968    0.000   11.327   14.740
##    .Emotion_rgl_g5   10.217    1.019   10.023    0.000    8.219   12.215
##    .ToM_age5          1.211    0.106   11.377    0.000    1.002    1.420
##    .Prosocialty_g5    8.098    0.571   14.195    0.000    6.980    9.217
##    .OPEN             12.991    0.832   15.607    0.000   11.359   14.622
##    .CONSC            12.841    0.709   18.114    0.000   11.452   14.231
##    .EXTRAV           11.803    0.857   13.776    0.000   10.123   13.482
##    .AGREE            15.844    0.746   21.228    0.000   14.381   17.307
##    .NEUROT            8.381    0.956    8.764    0.000    6.506   10.255
##     Motor_fine        7.588    0.013  569.208    0.000    7.562    7.614
##     Motor_gross       5.504    0.017  322.781    0.000    5.470    5.537
##     Communication     6.497    0.026  253.464    0.000    6.447    6.547
##    Std.lv  Std.all
##    42.426    3.975
##    40.496    4.210
##    13.033    3.576
##    10.217    2.167
##     1.211    3.329
##     8.098    4.966
##    12.991    3.485
##    12.841    3.935
##    11.803    2.985
##    15.844    5.202
##     8.381    1.759
##     7.588    9.310
##     5.504    4.213
##     6.497    3.867
## 
## Variances:
##                    Estimate  Std.Err  z-value  P(>|z|) ci.lower ci.upper
##    .ToM_age5          0.132    0.004   37.162    0.000    0.125    0.139
##    .Prosocialty_g5    2.563    0.058   44.235    0.000    2.450    2.677
##    .Cogntv_blty_g5   39.793    3.302   12.050    0.000   33.320   46.265
##    .Self_regltn_g5    3.577    0.221   16.160    0.000    3.143    4.011
##     Motor_fine        0.664    0.026   25.554    0.000    0.613    0.715
##     Motor_gross       1.707    0.040   42.867    0.000    1.629    1.785
##     Communication     2.824    0.050   56.087    0.000    2.725    2.922
##    .Verbal_blty_g5   73.345    3.359   21.838    0.000   66.762   79.927
##    .Spatil_blty_g5   65.286    2.826   23.103    0.000   59.747   70.824
##    .Independenc_g5    9.501    0.255   37.216    0.000    9.000   10.001
##    .Emotion_rgl_g5   17.144    0.376   45.578    0.000   16.406   17.881
##    .OPEN             13.489    0.220   61.361    0.000   13.058   13.920
##    .CONSC            10.295    0.200   51.537    0.000    9.903   10.686
##    .EXTRAV           15.518    0.256   60.583    0.000   15.016   16.020
##    .AGREE             8.978    0.213   42.088    0.000    8.560    9.396
##    .NEUROT           22.217    0.294   75.513    0.000   21.641   22.794
##    Std.lv  Std.all
##     0.132    0.999
##     2.563    0.964
##     0.981    0.981
##     0.947    0.947
##     0.664    1.000
##     1.707    1.000
##     2.824    1.000
##    73.345    0.644
##    65.286    0.706
##     9.501    0.715
##    17.144    0.771
##    13.489    0.971
##    10.295    0.966
##    15.518    0.993
##     8.978    0.968
##    22.217    0.979
## 
## Defined Parameters:
##                    Estimate  Std.Err  z-value  P(>|z|) ci.lower ci.upper
##     fT_T_O            0.001    0.001    1.014    0.311   -0.001    0.004
##     gT_T_O            0.000    0.001    0.453    0.650   -0.001    0.002
##     cT_T_O           -0.001    0.001   -1.273    0.203   -0.004    0.001
##     fP_P_O           -0.011    0.013   -0.811    0.417   -0.036    0.015
##     gP_P_O            0.001    0.001    0.596    0.551   -0.002    0.004
##     cP_P_O           -0.008    0.010   -0.813    0.416   -0.026    0.011
##     fC_C_O            0.053    0.018    2.873    0.004    0.017    0.089
##     gC_C_O            0.025    0.009    2.627    0.009    0.006    0.043
##     cC_C_O            0.003    0.006    0.422    0.673   -0.009    0.015
##     fS_S_O            0.047    0.033    1.401    0.161   -0.019    0.112
##     gS_S_O            0.005    0.006    0.913    0.361   -0.006    0.016
##     cS_S_O            0.023    0.016    1.437    0.151   -0.008    0.053
##     total_fO          0.092    0.061    1.511    0.131   -0.027    0.211
##     total_gO          0.108    0.036    2.973    0.003    0.037    0.180
##     total_cO          0.030    0.029    1.037    0.300   -0.027    0.088
##     fT_T_C           -0.002    0.002   -1.081    0.280   -0.006    0.002
##     gT_T_C           -0.001    0.001   -0.478    0.633   -0.003    0.002
##     cT_T_C            0.002    0.001    1.900    0.057   -0.000    0.004
##     fP_P_C           -0.010    0.012   -0.833    0.405   -0.034    0.014
##     gP_P_C            0.001    0.001    0.602    0.547   -0.002    0.003
##     cP_P_C           -0.007    0.009   -0.849    0.396   -0.025    0.010
##     fC_C_C            0.004    0.011    0.331    0.740   -0.018    0.025
##     gC_C_C            0.002    0.005    0.336    0.737   -0.008    0.012
##     cC_C_C            0.000    0.001    0.256    0.798   -0.001    0.002
##     fS_S_C            0.101    0.030    3.310    0.001    0.041    0.161
##     gS_S_C            0.011    0.009    1.156    0.248   -0.008    0.029
##     cS_S_C            0.049    0.014    3.463    0.001    0.021    0.076
##     total_fC          0.095    0.052    1.840    0.066   -0.006    0.197
##     total_gC          0.038    0.033    1.148    0.251   -0.027    0.103
##     total_cC          0.084    0.027    3.159    0.002    0.032    0.137
##     fT_T_E           -0.003    0.002   -1.135    0.256   -0.007    0.002
##     gT_T_E           -0.001    0.001   -0.490    0.624   -0.003    0.002
##     cT_T_E            0.003    0.001    1.858    0.063   -0.000    0.005
##     fP_P_E            0.004    0.014    0.310    0.757   -0.022    0.031
##     gP_P_E           -0.000    0.001   -0.289    0.772   -0.003    0.002
##     cP_P_E            0.003    0.010    0.312    0.755   -0.016    0.022
##     fC_C_E           -0.013    0.014   -0.911    0.362   -0.041    0.015
##     gC_C_E           -0.006    0.007   -0.919    0.358   -0.019    0.007
##     cC_C_E           -0.001    0.002   -0.390    0.697   -0.004    0.003
##     fS_S_E            0.044    0.035    1.248    0.212   -0.025    0.112
##     gS_S_E            0.005    0.005    0.874    0.382   -0.006    0.015
##     cS_S_E            0.021    0.017    1.264    0.206   -0.012    0.054
##     total_fE          0.065    0.062    1.061    0.289   -0.056    0.186
##     total_gE          0.037    0.040    0.939    0.348   -0.041    0.115
##     total_cE          0.089    0.032    2.815    0.005    0.027    0.151
##     fT_T_A           -0.001    0.002   -0.977    0.329   -0.004    0.001
##     gT_T_A           -0.000    0.001   -0.466    0.641   -0.002    0.001
##     cT_T_A            0.001    0.001    1.585    0.113   -0.000    0.003
##     fP_P_A           -0.006    0.012   -0.495    0.621   -0.029    0.017
##     gP_P_A            0.000    0.001    0.424    0.672   -0.002    0.003
##     cP_P_A           -0.004    0.009   -0.496    0.620   -0.021    0.012
##     fC_C_A           -0.034    0.014   -2.319    0.020   -0.062   -0.005
##     gC_C_A           -0.016    0.008   -2.050    0.040   -0.031   -0.001
##     cC_C_A           -0.002    0.004   -0.425    0.671   -0.009    0.006
##     fS_S_A            0.112    0.032    3.477    0.001    0.049    0.175
##     gS_S_A            0.012    0.010    1.180    0.238   -0.008    0.032
##     cS_S_A            0.054    0.015    3.541    0.000    0.024    0.084
##     total_fA          0.113    0.050    2.246    0.025    0.014    0.212
##     total_gA         -0.048    0.031   -1.542    0.123   -0.108    0.013
##     total_cA          0.079    0.026    2.997    0.003    0.027    0.131
##     fT_T_N           -0.001    0.001   -0.708    0.479   -0.004    0.002
##     gT_T_N           -0.000    0.001   -0.440    0.660   -0.001    0.001
##     cT_T_N            0.001    0.001    0.779    0.436   -0.001    0.003
##     fP_P_N            0.069    0.020    3.503    0.000    0.030    0.107
##     gP_P_N           -0.005    0.006   -0.875    0.381   -0.018    0.007
##     cP_P_N            0.050    0.014    3.637    0.000    0.023    0.077
##     fC_C_N            0.066    0.022    3.016    0.003    0.023    0.109
##     gC_C_N            0.031    0.013    2.423    0.015    0.006    0.056
##     cC_C_N            0.003    0.008    0.428    0.668   -0.012    0.018
##     fS_S_N           -0.157    0.048   -3.288    0.001   -0.251   -0.064
##     gS_S_N           -0.017    0.015   -1.155    0.248   -0.046    0.012
##     cS_S_N           -0.076    0.024   -3.206    0.001   -0.123   -0.030
##     total_fN          0.114    0.068    1.671    0.095   -0.020    0.247
##     total_gN         -0.237    0.045   -5.291    0.000   -0.325   -0.149
##     total_cN          0.093    0.038    2.467    0.014    0.019    0.166
##    Std.lv  Std.all
##     0.001    0.000
##     0.000    0.000
##    -0.001   -0.001
##    -0.011   -0.002
##     0.001    0.000
##    -0.008   -0.003
##     0.053    0.012
##     0.025    0.009
##     0.003    0.001
##     0.047    0.010
##     0.005    0.002
##     0.023    0.010
##     0.092    0.020
##     0.108    0.038
##     0.030    0.014
##    -0.002   -0.001
##    -0.001   -0.000
##     0.002    0.001
##    -0.010   -0.003
##     0.001    0.000
##    -0.007   -0.004
##     0.004    0.001
##     0.002    0.001
##     0.000    0.000
##     0.101    0.025
##     0.011    0.004
##     0.049    0.025
##     0.095    0.024
##     0.038    0.015
##     0.084    0.043
##    -0.003   -0.001
##    -0.001   -0.000
##     0.003    0.001
##     0.004    0.001
##    -0.000   -0.000
##     0.003    0.001
##    -0.013   -0.003
##    -0.006   -0.002
##    -0.001   -0.000
##     0.044    0.009
##     0.005    0.002
##     0.021    0.009
##     0.065    0.013
##     0.037    0.012
##     0.089    0.038
##    -0.001   -0.000
##    -0.000   -0.000
##     0.001    0.001
##    -0.006   -0.002
##     0.000    0.000
##    -0.004   -0.002
##    -0.034   -0.009
##    -0.016   -0.007
##    -0.002   -0.001
##     0.112    0.030
##     0.012    0.005
##     0.054    0.030
##     0.113    0.030
##    -0.048   -0.020
##     0.079    0.044
##    -0.001   -0.000
##    -0.000   -0.000
##     0.001    0.000
##     0.069    0.012
##    -0.005   -0.001
##     0.050    0.018
##     0.066    0.011
##     0.031    0.008
##     0.003    0.001
##    -0.157   -0.027
##    -0.017   -0.005
##    -0.076   -0.027
##     0.114    0.019
##    -0.237   -0.065
##     0.093    0.033
```

## Model 2 (moderately adjusted)

```
## lavaan 0.6-18 ended normally after 784 iterations
## 
##   Estimator                                         ML
##   Optimization method                           NLMINB
##   Number of model parameters                       197
## 
##   Number of observations                          8362
## 
## Model Test User Model:
##                                               Standard      Scaled
##   Test Statistic                              1345.770    1116.422
##   Degrees of freedom                                76          76
##   P-value (Chi-square)                           0.000       0.000
##   Scaling correction factor                                  1.205
##     Satorra-Bentler correction                                    
## 
## Model Test Baseline Model:
## 
##   Test statistic                             13846.128   10669.351
##   Degrees of freedom                               245         245
##   P-value                                        0.000       0.000
##   Scaling correction factor                                  1.298
## 
## User Model versus Baseline Model:
## 
##   Comparative Fit Index (CFI)                    0.907       0.900
##   Tucker-Lewis Index (TLI)                       0.699       0.678
##                                                                   
##   Robust Comparative Fit Index (CFI)                         0.907
##   Robust Tucker-Lewis Index (TLI)                            0.701
## 
## Loglikelihood and Information Criteria:
## 
##   Loglikelihood user model (H0)            -277611.063 -277611.063
##   Loglikelihood unrestricted model (H1)    -276938.178 -276938.178
##                                                                   
##   Akaike (AIC)                              555616.126  555616.126
##   Bayesian (BIC)                            557001.322  557001.322
##   Sample-size adjusted Bayesian (SABIC)     556375.293  556375.293
## 
## Root Mean Square Error of Approximation:
## 
##   RMSEA                                          0.045       0.040
##   90 Percent confidence interval - lower         0.043       0.039
##   90 Percent confidence interval - upper         0.047       0.042
##   P-value H_0: RMSEA <= 0.050                    1.000       1.000
##   P-value H_0: RMSEA >= 0.080                    0.000       0.000
##                                                                   
##   Robust RMSEA                                               0.044
##   90 Percent confidence interval - lower                     0.042
##   90 Percent confidence interval - upper                     0.047
##   P-value H_0: Robust RMSEA <= 0.050                         1.000
##   P-value H_0: Robust RMSEA >= 0.080                         0.000
## 
## Standardized Root Mean Square Residual:
## 
##   SRMR                                           0.023       0.023
## 
## Parameter Estimates:
## 
##   Standard errors                           Robust.sem
##   Information                                 Expected
##   Information saturated (h1) model          Structured
## 
## Latent Variables:
##                             Estimate  Std.Err  z-value  P(>|z|) ci.lower
##   Cognitive_ability_age5 =~                                             
##     Verbal_blty_g5             1.000                               1.000
##     Spatil_blty_g5             0.570    0.035   16.355    0.000    0.502
##   Self_regulation_age5 =~                                               
##     Independenc_g5             1.000                               1.000
##     Emotion_rgl_g5             1.284    0.050   25.733    0.000    1.186
##  ci.upper   Std.lv  Std.all
##                            
##     1.000    7.665    0.718
##     0.638    4.368    0.454
##                            
##     1.000    1.846    0.507
##     1.382    2.371    0.503
## 
## Regressions:
##                            Estimate  Std.Err  z-value  P(>|z|) ci.lower
##   ToM_age5 ~                                                           
##     Motr_fn   (fT)           -0.011    0.006   -1.775    0.076   -0.022
##     Mtr_grs   (gT)            0.001    0.003    0.194    0.847   -0.006
##     Cmmnctn   (cT)            0.006    0.003    2.069    0.039    0.000
##     CM_Age                   -0.004    0.009   -0.458    0.647   -0.023
##   Prosociality_age5 ~                                                  
##     Motr_fn   (fP)            0.165    0.030    5.486    0.000    0.106
##     Mtr_grs   (gP)            0.000    0.016    0.012    0.991   -0.032
##     Cmmnctn   (cP)            0.128    0.013    9.483    0.000    0.101
##     CM_Age                   -0.114    0.052   -2.182    0.029   -0.217
##   Cognitive_ability_age5 ~                                             
##     Motr_fn   (fC)            0.440    0.164    2.677    0.007    0.118
##     Mtr_grs   (gC)            0.430    0.108    3.997    0.000    0.219
##     Cmmnctn   (cC)            0.238    0.079    3.004    0.003    0.083
##     CM_Age                    0.438    0.293    1.495    0.135   -0.136
##   Self_regulation_age5 ~                                               
##     Motr_fn   (fS)            0.260    0.044    5.904    0.000    0.174
##     Mtr_grs   (gS)            0.064    0.028    2.285    0.022    0.009
##     Cmmnctn   (cS)            0.146    0.023    6.288    0.000    0.100
##     CM_Age                   -0.081    0.080   -1.003    0.316   -0.238
##   OPEN ~                                                               
##     Motr_fn  (F_O)            0.002    0.062    0.035    0.972   -0.119
##     Mtr_grs  (G_O)            0.086    0.038    2.291    0.022    0.012
##     Cmmnctn  (C_O)           -0.001    0.030   -0.023    0.982   -0.060
##     ToM_ag5  (T_O)           -0.231    0.133   -1.742    0.082   -0.491
##     Prscl_5  (P_O)           -0.072    0.066   -1.095    0.273   -0.202
##     Cgnt__5 (Cg_O)            0.066    0.016    4.138    0.000    0.035
##     Slf_r_5  (S_O)            0.171    0.099    1.737    0.082   -0.022
##   CONSC ~                                                              
##     Motr_fn  (F_C)            0.004    0.054    0.070    0.944   -0.101
##     Mtr_grs  (G_C)            0.024    0.034    0.706    0.480   -0.043
##     Cmmnctn  (C_C)            0.049    0.028    1.769    0.077   -0.005
##     ToM_ag5  (T_C)            0.332    0.110    3.025    0.002    0.117
##     Prscl_5  (P_C)           -0.059    0.061   -0.961    0.337   -0.179
##     Cgnt__5 (Cg_C)            0.001    0.011    0.054    0.957   -0.021
##     Slf_r_5  (S_C)            0.333    0.078    4.259    0.000    0.180
##   EXTRAV ~                                                             
##     Motr_fn  (F_E)            0.013    0.063    0.205    0.838   -0.111
##     Mtr_grs  (G_E)            0.060    0.040    1.491    0.136   -0.019
##     Cmmnctn  (C_E)            0.077    0.032    2.428    0.015    0.015
##     ToM_ag5  (T_E)            0.356    0.141    2.531    0.011    0.080
##     Prscl_5  (P_E)            0.056    0.067    0.835    0.404   -0.076
##     Cgnt__5 (Cg_E)           -0.023    0.015   -1.526    0.127   -0.053
##     Slf_r_5  (S_E)            0.062    0.101    0.615    0.538   -0.136
##   AGREE ~                                                              
##     Motr_fn  (F_A)            0.012    0.052    0.224    0.823   -0.090
##     Mtr_grs  (G_A)           -0.015    0.033   -0.453    0.650   -0.079
##     Cmmnctn  (C_A)           -0.003    0.026   -0.113    0.910   -0.055
##     ToM_ag5  (T_A)            0.138    0.105    1.314    0.189   -0.068
##     Prscl_5  (P_A)           -0.022    0.056   -0.394    0.693   -0.133
##     Cgnt__5 (Cg_A)           -0.042    0.013   -3.381    0.001   -0.067
##     Slf_r_5  (S_A)            0.289    0.076    3.794    0.000    0.140
##   NEUROT ~                                                             
##     Motr_fn  (F_N)            0.076    0.067    1.133    0.257   -0.055
##     Mtr_grs  (G_N)           -0.122    0.048   -2.555    0.011   -0.216
##     Cmmnctn  (C_N)           -0.044    0.034   -1.294    0.196   -0.111
##     ToM_ag5  (T_N)           -0.040    0.175   -0.230    0.818   -0.384
##     Prscl_5  (P_N)            0.336    0.084    3.987    0.000    0.171
##     Cgnt__5 (Cg_N)            0.060    0.017    3.541    0.000    0.027
##     Slf_r_5  (S_N)           -0.650    0.117   -5.576    0.000   -0.879
##   OPEN ~                                                               
##     Sex_Mal                  -0.144    0.109   -1.324    0.185   -0.357
##     CM_Age                   -0.050    0.102   -0.490    0.624   -0.249
##     STRATUM                   0.316    0.133    2.372    0.018    0.055
##     STRATUM                   1.238    0.289    4.286    0.000    0.672
##     STRATUM                   0.421    0.231    1.825    0.068   -0.031
##     STRATUM                   0.310    0.185    1.674    0.094   -0.053
##     STRATUM                   0.177    0.176    1.006    0.315   -0.168
##     STRATUM                   0.168    0.238    0.705    0.481   -0.299
##     STRATUM                  -0.073    0.211   -0.346    0.730   -0.486
##     STRATUM                   0.300    0.186    1.608    0.108   -0.066
##     Income                    0.028    0.043    0.652    0.514   -0.056
##   CONSC ~                                                              
##     Sex_Mal                   0.121    0.086    1.415    0.157   -0.047
##     CM_Age                   -0.037    0.082   -0.457    0.648   -0.198
##     STRATUM                  -0.061    0.133   -0.461    0.645   -0.321
##     STRATUM                  -0.015    0.157   -0.095    0.924   -0.322
##     STRATUM                   0.137    0.204    0.672    0.502   -0.263
##     STRATUM                  -0.138    0.215   -0.642    0.521   -0.558
##     STRATUM                  -0.298    0.180   -1.659    0.097   -0.650
##     STRATUM                  -0.111    0.189   -0.588    0.557   -0.480
##     STRATUM                   0.064    0.222    0.286    0.775   -0.373
##     STRATUM                  -0.239    0.140   -1.703    0.089   -0.513
##     Income                    0.004    0.042    0.107    0.915   -0.077
##   EXTRAV ~                                                             
##     Sex_Mal                  -0.196    0.111   -1.766    0.077   -0.414
##     CM_Age                    0.012    0.097    0.127    0.899   -0.177
##     STRATUM                  -0.187    0.131   -1.428    0.153   -0.443
##     STRATUM                  -0.178    0.190   -0.938    0.348   -0.550
##     STRATUM                   0.534    0.284    1.884    0.060   -0.022
##     STRATUM                   0.331    0.213    1.551    0.121   -0.087
##     STRATUM                   0.146    0.220    0.666    0.505   -0.285
##     STRATUM                   0.031    0.233    0.135    0.893   -0.425
##     STRATUM                  -0.129    0.169   -0.765    0.445   -0.460
##     STRATUM                  -0.169    0.157   -1.072    0.284   -0.477
##     Income                    0.202    0.043    4.725    0.000    0.118
##   AGREE ~                                                              
##     Sex_Mal                  -0.861    0.085  -10.127    0.000   -1.028
##     CM_Age                    0.124    0.079    1.563    0.118   -0.031
##     STRATUM                  -0.013    0.107   -0.121    0.904   -0.224
##     STRATUM                  -0.116    0.150   -0.773    0.440   -0.410
##     STRATUM                   0.171    0.151    1.130    0.258   -0.125
##     STRATUM                   0.360    0.172    2.090    0.037    0.022
##     STRATUM                   0.346    0.135    2.570    0.010    0.082
##     STRATUM                   0.398    0.166    2.395    0.017    0.072
##     STRATUM                   0.239    0.149    1.598    0.110   -0.054
##     STRATUM                   0.076    0.161    0.473    0.636   -0.239
##     Income                    0.104    0.036    2.881    0.004    0.033
##   NEUROT ~                                                             
##     Sex_Mal                  -3.684    0.116  -31.709    0.000   -3.911
##     CM_Age                    0.201    0.124    1.624    0.104   -0.042
##     STRATUM                  -0.134    0.179   -0.749    0.454   -0.485
##     STRATUM                  -0.968    0.268   -3.612    0.000   -1.493
##     STRATUM                   0.068    0.275    0.248    0.804   -0.471
##     STRATUM                   0.003    0.293    0.011    0.991   -0.571
##     STRATUM                   0.820    0.231    3.543    0.000    0.366
##     STRATUM                   0.582    0.232    2.507    0.012    0.127
##     STRATUM                   0.447    0.210    2.124    0.034    0.035
##     STRATUM                   0.257    0.205    1.257    0.209   -0.144
##     Income                    0.066    0.061    1.081    0.280   -0.053
##   ToM_age5 ~                                                           
##     Sex_Mal                  -0.039    0.009   -4.335    0.000   -0.056
##     STRATUM                   0.001    0.014    0.069    0.945   -0.027
##     STRATUM                  -0.018    0.015   -1.203    0.229   -0.047
##     STRATUM                   0.066    0.028    2.398    0.016    0.012
##     STRATUM                   0.065    0.030    2.196    0.028    0.007
##     STRATUM                   0.081    0.028    2.925    0.003    0.027
##     STRATUM                   0.060    0.023    2.639    0.008    0.016
##     STRATUM                  -0.005    0.016   -0.320    0.749   -0.038
##     STRATUM                   0.023    0.018    1.315    0.189   -0.011
##     Income                    0.015    0.004    4.082    0.000    0.008
##   Prosociality_age5 ~                                                  
##     Sex_Mal                  -0.405    0.038  -10.713    0.000   -0.479
##     STRATUM                  -0.104    0.051   -2.035    0.042   -0.204
##     STRATUM                  -0.168    0.074   -2.276    0.023   -0.314
##     STRATUM                  -0.056    0.081   -0.697    0.486   -0.214
##     STRATUM                  -0.060    0.079   -0.758    0.449   -0.216
##     STRATUM                  -0.063    0.081   -0.782    0.434   -0.222
##     STRATUM                  -0.174    0.087   -2.013    0.044   -0.344
##     STRATUM                  -0.022    0.094   -0.235    0.814   -0.207
##     STRATUM                   0.053    0.073    0.725    0.468   -0.091
##     Income                    0.079    0.016    4.875    0.000    0.047
##   Cognitive_ability_age5 ~                                             
##     Sex_Mal                  -0.642    0.265   -2.418    0.016   -1.162
##     STRATUM                  -1.487    0.554   -2.686    0.007   -2.573
##     STRATUM                  -7.365    2.041   -3.608    0.000  -11.366
##     STRATUM                   0.807    0.863    0.935    0.350   -0.884
##     STRATUM                   0.130    0.964    0.135    0.893   -1.759
##     STRATUM                   1.205    0.723    1.665    0.096   -0.213
##     STRATUM                  -1.248    0.735   -1.698    0.090   -2.688
##     STRATUM                  -1.091    0.600   -1.816    0.069   -2.267
##     STRATUM                  -2.507    0.763   -3.288    0.001   -4.002
##     Income                    2.152    0.141   15.228    0.000    1.875
##   Self_regulation_age5 ~                                               
##     Sex_Mal                  -0.650    0.065   -9.987    0.000   -0.778
##     STRATUM                  -0.203    0.104   -1.952    0.051   -0.407
##     STRATUM                  -0.524    0.193   -2.722    0.006   -0.902
##     STRATUM                   0.821    0.184    4.471    0.000    0.461
##     STRATUM                   0.025    0.179    0.141    0.888   -0.325
##     STRATUM                   0.389    0.176    2.209    0.027    0.044
##     STRATUM                   0.000    0.197    0.000    1.000   -0.385
##     STRATUM                   0.191    0.155    1.229    0.219   -0.114
##     STRATUM                  -0.112    0.141   -0.793    0.428   -0.389
##     Income                    0.344    0.032   10.842    0.000    0.282
##  ci.upper   Std.lv  Std.all
##                            
##     0.001   -0.011   -0.024
##     0.007    0.001    0.002
##     0.011    0.006    0.026
##     0.014   -0.004   -0.006
##                            
##     0.223    0.165    0.082
##     0.033    0.000    0.000
##     0.154    0.128    0.132
##    -0.012   -0.114   -0.034
##                            
##     0.762    0.057    0.047
##     0.641    0.056    0.073
##     0.393    0.031    0.052
##     1.013    0.057    0.028
##                            
##     0.347    0.141    0.115
##     0.118    0.035    0.045
##     0.191    0.079    0.133
##     0.077   -0.044   -0.021
##                            
##     0.123    0.002    0.000
##     0.160    0.086    0.030
##     0.058   -0.001   -0.000
##     0.029   -0.231   -0.023
##     0.057   -0.072   -0.032
##     0.098    0.508    0.136
##     0.365    0.316    0.085
##                            
##     0.109    0.004    0.001
##     0.092    0.024    0.010
##     0.104    0.049    0.025
##     0.548    0.332    0.037
##     0.061   -0.059   -0.029
##     0.023    0.005    0.001
##     0.487    0.616    0.189
##                            
##     0.137    0.013    0.003
##     0.138    0.060    0.020
##     0.139    0.077    0.033
##     0.631    0.356    0.033
##     0.189    0.056    0.023
##     0.007   -0.177   -0.045
##     0.260    0.115    0.029
##                            
##     0.113    0.012    0.003
##     0.049   -0.015   -0.006
##     0.049   -0.003   -0.002
##     0.345    0.138    0.017
##     0.088   -0.022   -0.012
##    -0.018   -0.325   -0.107
##     0.438    0.533    0.175
##                            
##     0.207    0.076    0.013
##    -0.029   -0.122   -0.034
##     0.023   -0.044   -0.015
##     0.304   -0.040   -0.003
##     0.501    0.336    0.114
##     0.094    0.462    0.097
##    -0.422   -1.201   -0.252
##                            
##     0.069   -0.144   -0.019
##     0.149   -0.050   -0.006
##     0.576    0.316    0.038
##     1.804    1.238    0.074
##     0.874    0.421    0.018
##     0.673    0.310    0.010
##     0.521    0.177    0.011
##     0.635    0.168    0.009
##     0.341   -0.073   -0.004
##     0.665    0.300    0.009
##     0.111    0.028    0.010
##                            
##     0.290    0.121    0.019
##     0.123   -0.037   -0.006
##     0.199   -0.061   -0.008
##     0.292   -0.015   -0.001
##     0.537    0.137    0.007
##     0.283   -0.138   -0.005
##     0.054   -0.298   -0.022
##     0.259   -0.111   -0.006
##     0.500    0.064    0.004
##     0.036   -0.239   -0.008
##     0.086    0.004    0.002
##                            
##     0.022   -0.196   -0.025
##     0.201    0.012    0.002
##     0.069   -0.187   -0.021
##     0.194   -0.178   -0.010
##     1.090    0.534    0.021
##     0.749    0.331    0.010
##     0.577    0.146    0.009
##     0.488    0.031    0.002
##     0.202   -0.129   -0.006
##     0.140   -0.169   -0.005
##     0.285    0.202    0.070
##                            
##    -0.694   -0.861   -0.141
##     0.279    0.124    0.020
##     0.198   -0.013   -0.002
##     0.178   -0.116   -0.009
##     0.467    0.171    0.009
##     0.698    0.360    0.014
##     0.610    0.346    0.027
##     0.723    0.398    0.025
##     0.531    0.239    0.014
##     0.391    0.076    0.003
##     0.175    0.104    0.047
##                            
##    -3.456   -3.684   -0.386
##     0.443    0.201    0.020
##     0.217   -0.134   -0.013
##    -0.443   -0.968   -0.045
##     0.607    0.068    0.002
##     0.577    0.003    0.000
##     1.273    0.820    0.041
##     1.037    0.582    0.023
##     0.859    0.447    0.017
##     0.658    0.257    0.006
##     0.185    0.066    0.019
##                            
##    -0.021   -0.039   -0.053
##     0.028    0.001    0.001
##     0.011   -0.018   -0.011
##     0.121    0.066    0.029
##     0.123    0.065    0.021
##     0.135    0.081    0.052
##     0.105    0.060    0.031
##     0.027   -0.005   -0.003
##     0.057    0.023    0.007
##     0.022    0.015    0.057
##                            
##    -0.331   -0.405   -0.124
##    -0.004   -0.104   -0.029
##    -0.023   -0.168   -0.023
##     0.102   -0.056   -0.005
##     0.096   -0.060   -0.004
##     0.095   -0.063   -0.009
##    -0.005   -0.174   -0.020
##     0.162   -0.022   -0.002
##     0.197    0.053    0.004
##     0.110    0.079    0.067
##                            
##    -0.122   -0.084   -0.042
##    -0.402   -0.194   -0.087
##    -3.365   -0.961   -0.215
##     2.498    0.105    0.017
##     2.020    0.017    0.002
##     2.622    0.157    0.037
##     0.193   -0.163   -0.031
##     0.086   -0.142   -0.026
##    -1.013   -0.327   -0.037
##     2.428    0.281    0.387
##                            
##    -0.522   -0.352   -0.176
##     0.001   -0.110   -0.049
##    -0.147   -0.284   -0.064
##     1.180    0.445    0.070
##     0.376    0.014    0.002
##     0.735    0.211    0.050
##     0.385    0.000    0.000
##     0.496    0.104    0.019
##     0.165   -0.061   -0.007
##     0.406    0.186    0.257
## 
## Covariances:
##                             Estimate  Std.Err  z-value  P(>|z|) ci.lower
##  .ToM_age5 ~~                                                           
##    .Prosocialty_g5             0.018    0.007    2.695    0.007    0.005
##  .Cognitive_ability_age5 ~~                                             
##    .ToM_age5                   0.348    0.041    8.505    0.000    0.267
##  .Self_regulation_age5 ~~                                               
##    .ToM_age5                   0.040    0.013    3.146    0.002    0.015
##  .Cognitive_ability_age5 ~~                                             
##    .Prosocialty_g5             0.932    0.206    4.516    0.000    0.528
##  .Self_regulation_age5 ~~                                               
##    .Prosocialty_g5             1.670    0.072   23.287    0.000    1.530
##  .Cognitive_ability_age5 ~~                                             
##    .Self_regltn_g5             4.135    0.393   10.511    0.000    3.364
##   Motor_fine ~~                                                         
##     Motor_gross                0.202    0.019   10.635    0.000    0.164
##     Communication              0.361    0.023   15.633    0.000    0.315
##   Motor_gross ~~                                                        
##     Communication              0.703    0.032   21.740    0.000    0.640
##  .OPEN ~~                                                               
##    .CONSC                      1.825    0.174   10.476    0.000    1.484
##    .EXTRAV                     1.942    0.196    9.905    0.000    1.558
##    .AGREE                      2.325    0.164   14.156    0.000    2.003
##    .NEUROT                    -0.033    0.218   -0.153    0.878   -0.461
##  .CONSC ~~                                                              
##    .EXTRAV                     2.113    0.167   12.666    0.000    1.786
##    .AGREE                      2.900    0.155   18.756    0.000    2.597
##    .NEUROT                    -3.278    0.192  -17.044    0.000   -3.655
##  .EXTRAV ~~                                                             
##    .AGREE                      1.448    0.162    8.921    0.000    1.130
##    .NEUROT                    -5.704    0.220  -25.924    0.000   -6.135
##  .AGREE ~~                                                              
##    .NEUROT                    -0.638    0.170   -3.762    0.000   -0.970
##  ci.upper   Std.lv  Std.all
##                            
##     0.030    0.018    0.031
##                            
##     0.428    0.053    0.147
##                            
##     0.065    0.024    0.066
##                            
##     1.337    0.142    0.090
##                            
##     1.811    0.994    0.628
##                            
##     4.906    0.376    0.376
##                            
##     0.239    0.202    0.189
##     0.406    0.361    0.264
##                            
##     0.766    0.703    0.320
##                            
##     2.167    1.825    0.155
##     2.326    1.942    0.135
##     2.646    2.325    0.214
##     0.394   -0.033   -0.002
##                            
##     2.440    2.113    0.168
##     3.203    2.900    0.306
##    -2.901   -3.278   -0.235
##                            
##     1.766    1.448    0.125
##    -5.273   -5.704   -0.333
##                            
##    -0.305   -0.638   -0.049
## 
## Intercepts:
##                    Estimate  Std.Err  z-value  P(>|z|) ci.lower ci.upper
##    .Verbal_blty_g5   38.839    3.018   12.870    0.000   32.925   44.754
##    .Spatil_blty_g5   41.813    1.831   22.834    0.000   38.224   45.402
##    .Independenc_g5   13.158    0.869   15.142    0.000   11.455   14.861
##    .Emotion_rgl_g5    9.956    1.118    8.901    0.000    7.764   12.148
##    .ToM_age5          1.197    0.107   11.236    0.000    0.988    1.406
##    .Prosocialty_g5    8.387    0.572   14.670    0.000    7.266    9.507
##    .OPEN             13.234    1.249   10.595    0.000   10.786   15.682
##    .CONSC            13.113    1.053   12.451    0.000   11.049   15.177
##    .EXTRAV           11.231    1.239    9.067    0.000    8.803   13.658
##    .AGREE            15.318    1.010   15.160    0.000   13.338   17.298
##    .NEUROT           10.123    1.408    7.192    0.000    7.364   12.882
##     Motor_fine        7.588    0.013  570.616    0.000    7.562    7.614
##     Motor_gross       5.503    0.017  322.443    0.000    5.470    5.537
##     Communication     6.496    0.026  253.700    0.000    6.446    6.546
##    Std.lv  Std.all
##    38.839    3.636
##    41.813    4.342
##    13.158    3.612
##     9.956    2.112
##     1.197    3.290
##     8.387    5.155
##    13.234    3.549
##    13.113    4.019
##    11.231    2.841
##    15.318    5.032
##    10.123    2.122
##     7.588    9.318
##     5.503    4.212
##     6.496    3.867
## 
## Variances:
##                    Estimate  Std.Err  z-value  P(>|z|) ci.lower ci.upper
##    .ToM_age5          0.131    0.004   37.090    0.000    0.124    0.138
##    .Prosocialty_g5    2.507    0.056   44.705    0.000    2.397    2.617
##    .Cogntv_blty_g5   42.798    3.315   12.909    0.000   36.300   49.296
##    .Self_regltn_g5    2.822    0.184   15.362    0.000    2.462    3.182
##     Motor_fine        0.663    0.026   25.593    0.000    0.612    0.714
##     Motor_gross       1.707    0.040   42.750    0.000    1.629    1.785
##     Communication     2.823    0.050   56.127    0.000    2.724    2.921
##    .Verbal_blty_g5   55.351    3.065   18.058    0.000   49.343   61.359
##    .Spatil_blty_g5   73.667    2.455   30.002    0.000   68.855   78.480
##    .Independenc_g5    9.861    0.244   40.438    0.000    9.383   10.339
##    .Emotion_rgl_g5   16.607    0.383   43.340    0.000   15.856   17.358
##    .OPEN             13.427    0.220   60.990    0.000   12.995   13.858
##    .CONSC            10.272    0.201   51.221    0.000    9.879   10.665
##    .EXTRAV           15.424    0.249   62.027    0.000   14.936   15.911
##    .AGREE             8.765    0.209   41.999    0.000    8.356    9.174
##    .NEUROT           19.007    0.295   64.504    0.000   18.429   19.584
##    Std.lv  Std.all
##     0.131    0.988
##     2.507    0.947
##     0.728    0.728
##     0.828    0.828
##     0.663    1.000
##     1.707    1.000
##     2.823    1.000
##    55.351    0.485
##    73.667    0.794
##     9.861    0.743
##    16.607    0.747
##    13.427    0.966
##    10.272    0.965
##    15.424    0.987
##     8.765    0.946
##    19.007    0.835
## 
## Defined Parameters:
##                    Estimate  Std.Err  z-value  P(>|z|) ci.lower ci.upper
##     fT_T_O            0.002    0.002    1.319    0.187   -0.001    0.006
##     gT_T_O           -0.000    0.001   -0.193    0.847   -0.002    0.001
##     cT_T_O           -0.001    0.001   -1.290    0.197   -0.003    0.001
##     fP_P_O           -0.012    0.011   -1.087    0.277   -0.033    0.010
##     gP_P_O           -0.000    0.001   -0.012    0.991   -0.002    0.002
##     cP_P_O           -0.009    0.008   -1.090    0.276   -0.026    0.007
##     fC_C_O            0.029    0.013    2.265    0.024    0.004    0.054
##     gC_C_O            0.029    0.009    3.031    0.002    0.010    0.047
##     cC_C_O            0.016    0.007    2.382    0.017    0.003    0.029
##     fS_S_O            0.045    0.027    1.665    0.096   -0.008    0.097
##     gS_S_O            0.011    0.008    1.344    0.179   -0.005    0.027
##     cS_S_O            0.025    0.014    1.734    0.083   -0.003    0.053
##     total_fO          0.066    0.061    1.096    0.273   -0.052    0.185
##     total_gO          0.125    0.037    3.423    0.001    0.054    0.197
##     total_cO          0.030    0.030    1.000    0.317   -0.028    0.087
##     fT_T_C           -0.004    0.002   -1.487    0.137   -0.008    0.001
##     gT_T_C            0.000    0.001    0.192    0.847   -0.002    0.002
##     cT_T_C            0.002    0.001    1.765    0.078   -0.000    0.004
##     fP_P_C           -0.010    0.011   -0.921    0.357   -0.030    0.011
##     gP_P_C           -0.000    0.001   -0.012    0.991   -0.002    0.002
##     cP_P_C           -0.008    0.008   -0.947    0.344   -0.023    0.008
##     fC_C_C            0.000    0.005    0.054    0.957   -0.009    0.010
##     gC_C_C            0.000    0.005    0.054    0.957   -0.009    0.010
##     cC_C_C            0.000    0.003    0.054    0.957   -0.005    0.005
##     fS_S_C            0.087    0.026    3.343    0.001    0.036    0.138
##     gS_S_C            0.021    0.011    1.998    0.046    0.000    0.042
##     cS_S_C            0.049    0.013    3.693    0.000    0.023    0.074
##     total_fC          0.078    0.052    1.490    0.136   -0.024    0.180
##     total_gC          0.046    0.034    1.375    0.169   -0.020    0.112
##     total_cC          0.092    0.027    3.427    0.001    0.040    0.145
##     fT_T_E           -0.004    0.002   -1.514    0.130   -0.009    0.001
##     gT_T_E            0.000    0.001    0.190    0.849   -0.002    0.003
##     cT_T_E            0.002    0.001    1.649    0.099   -0.000    0.004
##     fP_P_E            0.009    0.011    0.819    0.413   -0.013    0.031
##     gP_P_E            0.000    0.001    0.012    0.991   -0.002    0.002
##     cP_P_E            0.007    0.009    0.834    0.405   -0.010    0.024
##     fC_C_E           -0.010    0.008   -1.261    0.207   -0.026    0.006
##     gC_C_E           -0.010    0.007   -1.452    0.147   -0.023    0.003
##     cC_C_E           -0.005    0.004   -1.385    0.166   -0.013    0.002
##     fS_S_E            0.016    0.027    0.606    0.544   -0.036    0.069
##     gS_S_E            0.004    0.007    0.590    0.555   -0.009    0.017
##     cS_S_E            0.009    0.015    0.612    0.541   -0.020    0.038
##     total_fE          0.025    0.061    0.403    0.687   -0.095    0.144
##     total_gE          0.054    0.040    1.366    0.172   -0.024    0.132
##     total_cE          0.090    0.032    2.830    0.005    0.028    0.152
##     fT_T_A           -0.001    0.001   -1.012    0.312   -0.004    0.001
##     gT_T_A            0.000    0.000    0.191    0.848   -0.001    0.001
##     cT_T_A            0.001    0.001    1.203    0.229   -0.000    0.002
##     fP_P_A           -0.004    0.009   -0.392    0.695   -0.022    0.015
##     gP_P_A           -0.000    0.000   -0.012    0.991   -0.001    0.001
##     cP_P_A           -0.003    0.007   -0.392    0.695   -0.017    0.011
##     fC_C_A           -0.019    0.009   -2.097    0.036   -0.036   -0.001
##     gC_C_A           -0.018    0.007   -2.543    0.011   -0.032   -0.004
##     cC_C_A           -0.010    0.005   -2.210    0.027   -0.019   -0.001
##     fS_S_A            0.075    0.024    3.128    0.002    0.028    0.122
##     gS_S_A            0.018    0.009    1.985    0.047    0.000    0.037
##     cS_S_A            0.042    0.013    3.251    0.001    0.017    0.068
##     total_fA          0.063    0.049    1.290    0.197   -0.033    0.159
##     total_gA         -0.015    0.031   -0.465    0.642   -0.076    0.047
##     total_cA          0.027    0.026    1.048    0.295   -0.024    0.078
##     fT_T_N            0.000    0.002    0.224    0.823   -0.003    0.004
##     gT_T_N           -0.000    0.000   -0.159    0.873   -0.000    0.000
##     cT_T_N           -0.000    0.001   -0.225    0.822   -0.002    0.002
##     fP_P_N            0.055    0.017    3.228    0.001    0.022    0.089
##     gP_P_N            0.000    0.006    0.012    0.991   -0.011    0.011
##     cP_P_N            0.043    0.012    3.599    0.000    0.020    0.066
##     fC_C_N            0.027    0.012    2.192    0.028    0.003    0.050
##     gC_C_N            0.026    0.010    2.521    0.012    0.006    0.046
##     cC_C_N            0.014    0.006    2.340    0.019    0.002    0.026
##     fS_S_N           -0.169    0.044   -3.835    0.000   -0.256   -0.083
##     gS_S_N           -0.041    0.020   -2.068    0.039   -0.081   -0.002
##     cS_S_N           -0.095    0.023   -4.167    0.000   -0.140   -0.050
##     total_fN         -0.011    0.067   -0.170    0.865   -0.142    0.119
##     total_gN         -0.138    0.043   -3.200    0.001   -0.222   -0.053
##     total_cN         -0.082    0.034   -2.397    0.017   -0.149   -0.015
##    Std.lv  Std.all
##     0.002    0.001
##    -0.000   -0.000
##    -0.001   -0.001
##    -0.012   -0.003
##    -0.000   -0.000
##    -0.009   -0.004
##     0.029    0.006
##     0.029    0.010
##     0.016    0.007
##     0.045    0.010
##     0.011    0.004
##     0.025    0.011
##     0.066    0.015
##     0.125    0.044
##     0.030    0.013
##    -0.004   -0.001
##     0.000    0.000
##     0.002    0.001
##    -0.010   -0.002
##    -0.000   -0.000
##    -0.008   -0.004
##     0.000    0.000
##     0.000    0.000
##     0.000    0.000
##     0.087    0.022
##     0.021    0.009
##     0.049    0.025
##     0.078    0.019
##     0.046    0.018
##     0.092    0.048
##    -0.004   -0.001
##     0.000    0.000
##     0.002    0.001
##     0.009    0.002
##     0.000    0.000
##     0.007    0.003
##    -0.010   -0.002
##    -0.010   -0.003
##    -0.005   -0.002
##     0.016    0.003
##     0.004    0.001
##     0.009    0.004
##     0.025    0.005
##     0.054    0.018
##     0.090    0.038
##    -0.001   -0.000
##     0.000    0.000
##     0.001    0.000
##    -0.004   -0.001
##    -0.000   -0.000
##    -0.003   -0.002
##    -0.019   -0.005
##    -0.018   -0.008
##    -0.010   -0.006
##     0.075    0.020
##     0.018    0.008
##     0.042    0.023
##     0.063    0.017
##    -0.015   -0.006
##     0.027    0.015
##     0.000    0.000
##    -0.000   -0.000
##    -0.000   -0.000
##     0.055    0.009
##     0.000    0.000
##     0.043    0.015
##     0.027    0.005
##     0.026    0.007
##     0.014    0.005
##    -0.169   -0.029
##    -0.041   -0.011
##    -0.095   -0.033
##    -0.011   -0.002
##    -0.138   -0.038
##    -0.082   -0.029
```

## Model 3 (fully adjusted)

```
## lavaan 0.6-18 ended normally after 994 iterations
## 
##   Estimator                                         ML
##   Optimization method                           NLMINB
##   Number of model parameters                       296
## 
##   Number of observations                          8078
## 
## Model Test User Model:
##                                               Standard      Scaled
##   Test Statistic                              1797.691    1433.103
##   Degrees of freedom                               131         131
##   P-value (Chi-square)                           0.000       0.000
##   Scaling correction factor                                  1.254
##     Satorra-Bentler correction                                    
## 
## Model Test Baseline Model:
## 
##   Test statistic                             15176.989   11179.146
##   Degrees of freedom                               399         399
##   P-value                                        0.000       0.000
##   Scaling correction factor                                  1.358
## 
## User Model versus Baseline Model:
## 
##   Comparative Fit Index (CFI)                    0.887       0.879
##   Tucker-Lewis Index (TLI)                       0.656       0.632
##                                                                   
##   Robust Comparative Fit Index (CFI)                         0.888
##   Robust Tucker-Lewis Index (TLI)                            0.660
## 
## Loglikelihood and Information Criteria:
## 
##   Loglikelihood user model (H0)            -267302.498 -267302.498
##   Loglikelihood unrestricted model (H1)    -266403.653 -266403.653
##                                                                   
##   Akaike (AIC)                              535196.997  535196.997
##   Bayesian (BIC)                            537268.079  537268.079
##   Sample-size adjusted Bayesian (SABIC)     536327.448  536327.448
## 
## Root Mean Square Error of Approximation:
## 
##   RMSEA                                          0.040       0.035
##   90 Percent confidence interval - lower         0.038       0.034
##   90 Percent confidence interval - upper         0.041       0.037
##   P-value H_0: RMSEA <= 0.050                    1.000       1.000
##   P-value H_0: RMSEA >= 0.080                    0.000       0.000
##                                                                   
##   Robust RMSEA                                               0.039
##   90 Percent confidence interval - lower                     0.037
##   90 Percent confidence interval - upper                     0.041
##   P-value H_0: Robust RMSEA <= 0.050                         1.000
##   P-value H_0: Robust RMSEA >= 0.080                         0.000
## 
## Standardized Root Mean Square Residual:
## 
##   SRMR                                           0.021       0.021
## 
## Parameter Estimates:
## 
##   Standard errors                           Robust.sem
##   Information                                 Expected
##   Information saturated (h1) model          Structured
## 
## Latent Variables:
##                             Estimate  Std.Err  z-value  P(>|z|) ci.lower
##   Cognitive_ability_age5 =~                                             
##     Verbal_blty_g5             1.000                               1.000
##     Spatil_blty_g5             0.505    0.029   17.273    0.000    0.448
##   Self_regulation_age5 =~                                               
##     Independenc_g5             1.000                               1.000
##     Emotion_rgl_g5             1.441    0.054   26.531    0.000    1.335
##  ci.upper   Std.lv  Std.all
##                            
##     1.000    8.021    0.758
##     0.562    4.052    0.422
##                            
##     1.000    1.778    0.489
##     1.547    2.562    0.544
## 
## Regressions:
##                            Estimate  Std.Err  z-value  P(>|z|) ci.lower
##   ToM_age5 ~                                                           
##     Motr_fn   (fT)           -0.013    0.006   -2.085    0.037   -0.025
##     Mtr_grs   (gT)            0.001    0.003    0.338    0.735   -0.006
##     Cmmnctn   (cT)            0.006    0.003    2.341    0.019    0.001
##     CM_Age                   -0.006    0.010   -0.612    0.540   -0.025
##   Prosociality_age5 ~                                                  
##     Motr_fn   (fP)            0.159    0.031    5.185    0.000    0.099
##     Mtr_grs   (gP)            0.001    0.017    0.079    0.937   -0.033
##     Cmmnctn   (cP)            0.128    0.014    9.382    0.000    0.101
##     CM_Age                   -0.115    0.052   -2.199    0.028   -0.218
##   Cognitive_ability_age5 ~                                             
##     Motr_fn   (fC)            0.354    0.154    2.304    0.021    0.053
##     Mtr_grs   (gC)            0.431    0.109    3.972    0.000    0.218
##     Cmmnctn   (cC)            0.319    0.077    4.148    0.000    0.168
##     CM_Age                    0.327    0.293    1.118    0.263   -0.246
##   Self_regulation_age5 ~                                               
##     Motr_fn   (fS)            0.207    0.041    5.011    0.000    0.126
##     Mtr_grs   (gS)            0.058    0.027    2.160    0.031    0.005
##     Cmmnctn   (cS)            0.157    0.022    7.035    0.000    0.113
##     CM_Age                   -0.053    0.077   -0.690    0.490   -0.205
##   OPEN ~                                                               
##     Motr_fn  (F_O)           -0.010    0.062   -0.163    0.871   -0.131
##     Mtr_grs  (G_O)            0.092    0.037    2.495    0.013    0.020
##     Cmmnctn  (C_O)            0.013    0.030    0.427    0.670   -0.046
##     ToM_ag5  (T_O)           -0.213    0.133   -1.599    0.110   -0.473
##     Prscl_5  (P_O)           -0.060    0.065   -0.921    0.357   -0.187
##     Cgnt__5 (Cg_O)            0.064    0.015    4.124    0.000    0.033
##     Slf_r_5  (S_O)            0.156    0.100    1.564    0.118   -0.039
##   CONSC ~                                                              
##     Motr_fn  (F_C)            0.005    0.053    0.096    0.923   -0.099
##     Mtr_grs  (G_C)            0.032    0.035    0.921    0.357   -0.037
##     Cmmnctn  (C_C)            0.045    0.028    1.593    0.111   -0.010
##     ToM_ag5  (T_C)            0.348    0.112    3.107    0.002    0.128
##     Prscl_5  (P_C)           -0.063    0.063   -0.993    0.321   -0.186
##     Cgnt__5 (Cg_C)            0.002    0.010    0.222    0.824   -0.018
##     Slf_r_5  (S_C)            0.369    0.084    4.411    0.000    0.205
##   EXTRAV ~                                                             
##     Motr_fn  (F_E)            0.001    0.065    0.019    0.985   -0.126
##     Mtr_grs  (G_E)            0.072    0.041    1.753    0.080   -0.008
##     Cmmnctn  (C_E)            0.069    0.033    2.118    0.034    0.005
##     ToM_ag5  (T_E)            0.330    0.143    2.314    0.021    0.050
##     Prscl_5  (P_E)            0.067    0.065    1.028    0.304   -0.060
##     Cgnt__5 (Cg_E)           -0.018    0.014   -1.327    0.185   -0.045
##     Slf_r_5  (S_E)            0.037    0.106    0.350    0.727   -0.170
##   AGREE ~                                                              
##     Motr_fn  (F_A)            0.005    0.052    0.090    0.928   -0.098
##     Mtr_grs  (G_A)            0.006    0.033    0.167    0.867   -0.059
##     Cmmnctn  (C_A)           -0.001    0.027   -0.028    0.978   -0.054
##     ToM_ag5  (T_A)            0.152    0.104    1.455    0.146   -0.053
##     Prscl_5  (P_A)           -0.006    0.056   -0.102    0.919   -0.115
##     Cgnt__5 (Cg_A)           -0.045    0.012   -3.580    0.000   -0.069
##     Slf_r_5  (S_A)            0.296    0.081    3.676    0.000    0.138
##   NEUROT ~                                                             
##     Motr_fn  (F_N)            0.045    0.069    0.657    0.511   -0.089
##     Mtr_grs  (G_N)           -0.101    0.049   -2.058    0.040   -0.198
##     Cmmnctn  (C_N)           -0.028    0.035   -0.799    0.424   -0.096
##     ToM_ag5  (T_N)           -0.052    0.174   -0.301    0.764   -0.394
##     Prscl_5  (P_N)            0.321    0.082    3.907    0.000    0.160
##     Cgnt__5 (Cg_N)            0.036    0.016    2.269    0.023    0.005
##     Slf_r_5  (S_N)           -0.645    0.120   -5.369    0.000   -0.880
##   OPEN ~                                                               
##     Sex_Mal                  -0.143    0.108   -1.324    0.185   -0.355
##     CM_Age                   -0.072    0.102   -0.706    0.480   -0.272
##     STRATUM                   0.208    0.135    1.541    0.123   -0.057
##     STRATUM                   0.379    0.272    1.397    0.162   -0.153
##     STRATUM                   0.517    0.224    2.308    0.021    0.078
##     STRATUM                   0.489    0.195    2.502    0.012    0.106
##     STRATUM                   0.159    0.176    0.902    0.367   -0.186
##     STRATUM                   0.207    0.235    0.881    0.378   -0.254
##     STRATUM                  -0.041    0.213   -0.192    0.847   -0.458
##     STRATUM                   0.361    0.181    1.998    0.046    0.007
##     Ethnc_B                   1.188    0.356    3.333    0.001    0.489
##     Ethnc_I                   0.524    0.315    1.664    0.096   -0.093
##     Ethnc_M                   0.833    0.311    2.680    0.007    0.224
##     Ethnc_O                   1.057    0.511    2.068    0.039    0.055
##     Ethn_PB                   1.568    0.372    4.214    0.000    0.839
##     Income                   -0.009    0.046   -0.189    0.850   -0.099
##     Mtrnl_E                   0.109    0.046    2.374    0.018    0.019
##     Mtrnl_A                   0.034    0.010    3.269    0.001    0.014
##     Mtr_M_H                   0.318    0.119    2.671    0.008    0.085
##     Bt_P__H                  -0.414    0.181   -2.286    0.022   -0.770
##     Siblngs                  -0.115    0.068   -1.704    0.088   -0.248
##     Sm__G_A                  -0.015    0.147   -0.100    0.921   -0.303
##   CONSC ~                                                              
##     Sex_Mal                   0.111    0.088    1.261    0.207   -0.062
##     CM_Age                   -0.047    0.084   -0.564    0.573   -0.211
##     STRATUM                  -0.131    0.137   -0.958    0.338   -0.400
##     STRATUM                  -0.411    0.204   -2.021    0.043   -0.810
##     STRATUM                   0.203    0.206    0.984    0.325   -0.201
##     STRATUM                  -0.056    0.214   -0.263    0.792   -0.476
##     STRATUM                  -0.304    0.180   -1.684    0.092   -0.657
##     STRATUM                  -0.170    0.188   -0.904    0.366   -0.539
##     STRATUM                   0.075    0.225    0.335    0.738   -0.365
##     STRATUM                  -0.266    0.142   -1.882    0.060   -0.544
##     Ethnc_B                   0.438    0.343    1.280    0.201   -0.233
##     Ethnc_I                   0.375    0.319    1.175    0.240   -0.250
##     Ethnc_M                  -0.612    0.261   -2.340    0.019   -1.124
##     Ethnc_O                   0.326    0.345    0.947    0.344   -0.349
##     Ethn_PB                   0.963    0.289    3.332    0.001    0.397
##     Income                    0.006    0.045    0.132    0.895   -0.083
##     Mtrnl_E                  -0.007    0.038   -0.186    0.853   -0.081
##     Mtrnl_A                  -0.020    0.009   -2.127    0.033   -0.038
##     Mtr_M_H                  -0.010    0.108   -0.096    0.924   -0.222
##     Bt_P__H                   0.053    0.147    0.365    0.715   -0.234
##     Siblngs                  -0.171    0.051   -3.319    0.001   -0.272
##     Sm__G_A                  -0.027    0.125   -0.214    0.830   -0.272
##   EXTRAV ~                                                             
##     Sex_Mal                  -0.206    0.113   -1.833    0.067   -0.427
##     CM_Age                    0.014    0.098    0.143    0.886   -0.178
##     STRATUM                  -0.235    0.133   -1.760    0.078   -0.496
##     STRATUM                  -0.516    0.231   -2.229    0.026   -0.969
##     STRATUM                   0.550    0.286    1.918    0.055   -0.012
##     STRATUM                   0.334    0.218    1.531    0.126   -0.094
##     STRATUM                   0.151    0.219    0.689    0.491   -0.279
##     STRATUM                   0.004    0.229    0.015    0.988   -0.445
##     STRATUM                  -0.143    0.169   -0.849    0.396   -0.474
##     STRATUM                  -0.195    0.155   -1.257    0.209   -0.500
##     Ethnc_B                  -0.507    0.417   -1.217    0.224   -1.324
##     Ethnc_I                   0.650    0.370    1.755    0.079   -0.076
##     Ethnc_M                  -0.107    0.370   -0.289    0.772   -0.833
##     Ethnc_O                  -0.310    0.456   -0.679    0.497   -1.204
##     Ethn_PB                   0.765    0.260    2.937    0.003    0.255
##     Income                    0.217    0.056    3.870    0.000    0.107
##     Mtrnl_E                  -0.018    0.045   -0.400    0.689   -0.106
##     Mtrnl_A                  -0.013    0.012   -1.099    0.272   -0.037
##     Mtr_M_H                  -0.501    0.135   -3.699    0.000   -0.766
##     Bt_P__H                  -0.114    0.177   -0.643    0.520   -0.462
##     Siblngs                  -0.024    0.060   -0.399    0.690   -0.142
##     Sm__G_A                  -0.212    0.157   -1.345    0.179   -0.520
##   AGREE ~                                                              
##     Sex_Mal                  -0.881    0.087  -10.167    0.000   -1.051
##     CM_Age                    0.109    0.080    1.357    0.175   -0.048
##     STRATUM                  -0.034    0.106   -0.318    0.750   -0.241
##     STRATUM                  -0.121    0.208   -0.583    0.560   -0.529
##     STRATUM                   0.180    0.150    1.197    0.231   -0.115
##     STRATUM                   0.349    0.182    1.916    0.055   -0.008
##     STRATUM                   0.317    0.136    2.328    0.020    0.050
##     STRATUM                   0.343    0.173    1.987    0.047    0.005
##     STRATUM                   0.213    0.150    1.422    0.155   -0.081
##     STRATUM                   0.078    0.169    0.459    0.646   -0.254
##     Ethnc_B                  -0.617    0.335   -1.844    0.065   -1.273
##     Ethnc_I                   0.272    0.276    0.986    0.324   -0.268
##     Ethnc_M                  -0.373    0.254   -1.469    0.142   -0.870
##     Ethnc_O                  -0.223    0.380   -0.585    0.558   -0.968
##     Ethn_PB                   0.387    0.309    1.250    0.211   -0.220
##     Income                    0.042    0.039    1.091    0.275   -0.034
##     Mtrnl_E                   0.054    0.038    1.429    0.153   -0.020
##     Mtrnl_A                   0.011    0.010    1.137    0.255   -0.008
##     Mtr_M_H                   0.007    0.104    0.068    0.946   -0.197
##     Bt_P__H                  -0.056    0.133   -0.425    0.671   -0.316
##     Siblngs                  -0.172    0.051   -3.357    0.001   -0.273
##     Sm__G_A                   0.043    0.117    0.372    0.710   -0.185
##   NEUROT ~                                                             
##     Sex_Mal                  -3.669    0.115  -31.859    0.000   -3.895
##     CM_Age                    0.230    0.125    1.837    0.066   -0.015
##     STRATUM                   0.020    0.181    0.108    0.914   -0.336
##     STRATUM                   0.190    0.380    0.501    0.616   -0.555
##     STRATUM                  -0.077    0.279   -0.274    0.784   -0.624
##     STRATUM                  -0.092    0.299   -0.306    0.759   -0.677
##     STRATUM                   0.791    0.227    3.483    0.000    0.346
##     STRATUM                   0.523    0.243    2.147    0.032    0.046
##     STRATUM                   0.353    0.212    1.664    0.096   -0.063
##     STRATUM                   0.300    0.196    1.529    0.126   -0.085
##     Ethnc_B                  -1.817    0.538   -3.378    0.001   -2.872
##     Ethnc_I                  -0.953    0.492   -1.938    0.053   -1.916
##     Ethnc_M                  -0.633    0.403   -1.568    0.117   -1.423
##     Ethnc_O                  -1.381    0.761   -1.814    0.070   -2.873
##     Ethn_PB                  -2.016    0.467   -4.315    0.000   -2.931
##     Income                    0.011    0.069    0.160    0.873   -0.124
##     Mtrnl_E                   0.064    0.057    1.117    0.264   -0.048
##     Mtrnl_A                   0.038    0.015    2.470    0.014    0.008
##     Mtr_M_H                   0.332    0.151    2.199    0.028    0.036
##     Bt_P__H                   0.135    0.203    0.666    0.505   -0.263
##     Siblngs                   0.055    0.076    0.726    0.468   -0.094
##     Sm__G_A                  -0.011    0.208   -0.054    0.957   -0.419
##   ToM_age5 ~                                                           
##     Sex_Mal                  -0.038    0.009   -4.147    0.000   -0.056
##     STRATUM                   0.008    0.014    0.563    0.573   -0.020
##     STRATUM                   0.010    0.018    0.548    0.584   -0.026
##     STRATUM                   0.058    0.028    2.108    0.035    0.004
##     STRATUM                   0.060    0.028    2.097    0.036    0.004
##     STRATUM                   0.074    0.028    2.660    0.008    0.019
##     STRATUM                   0.057    0.023    2.456    0.014    0.012
##     STRATUM                  -0.011    0.017   -0.632    0.527   -0.044
##     STRATUM                   0.029    0.018    1.591    0.112   -0.007
##     Ethnc_B                  -0.087    0.023   -3.848    0.000   -0.132
##     Ethnc_I                  -0.010    0.033   -0.303    0.762   -0.075
##     Ethnc_M                  -0.031    0.026   -1.174    0.240   -0.083
##     Ethnc_O                  -0.094    0.034   -2.813    0.005   -0.160
##     Ethn_PB                  -0.016    0.023   -0.702    0.483   -0.062
##     Income                    0.003    0.005    0.684    0.494   -0.006
##     Mtrnl_E                   0.015    0.004    3.839    0.000    0.007
##     Mtrnl_A                   0.002    0.001    2.370    0.018    0.000
##     Mtr_M_H                  -0.004    0.011   -0.338    0.735   -0.025
##     Bt_P__H                  -0.023    0.016   -1.450    0.147   -0.053
##     Siblngs                  -0.006    0.005   -1.207    0.227   -0.015
##     Sm__G_A                  -0.023    0.012   -1.900    0.057   -0.047
##   Prosociality_age5 ~                                                  
##     Sex_Mal                  -0.415    0.039  -10.628    0.000   -0.491
##     STRATUM                  -0.093    0.053   -1.767    0.077   -0.196
##     STRATUM                  -0.029    0.107   -0.268    0.789   -0.239
##     STRATUM                  -0.056    0.086   -0.647    0.518   -0.224
##     STRATUM                  -0.024    0.083   -0.287    0.774   -0.187
##     STRATUM                  -0.072    0.082   -0.881    0.378   -0.233
##     STRATUM                  -0.159    0.090   -1.767    0.077   -0.335
##     STRATUM                  -0.039    0.097   -0.402    0.688   -0.228
##     STRATUM                   0.054    0.079    0.683    0.495   -0.101
##     Ethnc_B                   0.097    0.174    0.556    0.578   -0.245
##     Ethnc_I                   0.073    0.124    0.591    0.555   -0.170
##     Ethnc_M                   0.145    0.124    1.173    0.241   -0.097
##     Ethnc_O                  -0.229    0.264   -0.867    0.386   -0.747
##     Ethn_PB                  -0.202    0.157   -1.286    0.198   -0.510
##     Income                    0.037    0.021    1.758    0.079   -0.004
##     Mtrnl_E                   0.025    0.019    1.366    0.172   -0.011
##     Mtrnl_A                   0.003    0.004    0.651    0.515   -0.006
##     Mtr_M_H                  -0.036    0.053   -0.679    0.497   -0.140
##     Bt_P__H                   0.071    0.074    0.963    0.335   -0.074
##     Siblngs                  -0.077    0.025   -3.096    0.002   -0.126
##     Sm__G_A                  -0.131    0.068   -1.934    0.053   -0.263
##   Cognitive_ability_age5 ~                                             
##     Sex_Mal                  -0.509    0.269   -1.891    0.059   -1.037
##     STRATUM                  -0.695    0.546   -1.274    0.203   -1.764
##     STRATUM                  -1.306    1.978   -0.660    0.509   -5.183
##     STRATUM                   0.239    0.877    0.273    0.785   -1.479
##     STRATUM                  -0.058    0.955   -0.061    0.951   -1.930
##     STRATUM                   0.592    0.706    0.839    0.401   -0.791
##     STRATUM                  -1.819    0.763   -2.384    0.017   -3.314
##     STRATUM                  -1.766    0.599   -2.948    0.003   -2.940
##     STRATUM                  -2.347    0.788   -2.977    0.003   -3.893
##     Ethnc_B                  -7.317    2.139   -3.420    0.001  -11.510
##     Ethnc_I                  -4.052    1.391   -2.914    0.004   -6.778
##     Ethnc_M                  -1.427    0.929   -1.536    0.125   -3.249
##     Ethnc_O                  -9.521    2.703   -3.523    0.000  -14.818
##     Ethn_PB                  -8.641    2.629   -3.287    0.001  -13.793
##     Income                    0.858    0.150    5.709    0.000    0.563
##     Mtrnl_E                   1.354    0.127   10.635    0.000    1.104
##     Mtrnl_A                   0.178    0.028    6.308    0.000    0.123
##     Mtr_M_H                  -0.291    0.309   -0.942    0.346   -0.897
##     Bt_P__H                  -0.355    0.440   -0.807    0.420   -1.218
##     Siblngs                  -1.490    0.169   -8.815    0.000   -1.822
##     Sm__G_A                  -1.342    0.406   -3.309    0.001   -2.137
##   Self_regulation_age5 ~                                               
##     Sex_Mal                  -0.598    0.063   -9.518    0.000   -0.722
##     STRATUM                  -0.093    0.100   -0.937    0.349   -0.289
##     STRATUM                  -0.084    0.231   -0.361    0.718   -0.537
##     STRATUM                   0.610    0.177    3.455    0.001    0.264
##     STRATUM                   0.031    0.167    0.188    0.851   -0.296
##     STRATUM                   0.286    0.165    1.738    0.082   -0.037
##     STRATUM                   0.049    0.192    0.253    0.801   -0.328
##     STRATUM                   0.099    0.143    0.691    0.489   -0.182
##     STRATUM                   0.002    0.141    0.013    0.990   -0.275
##     Ethnc_B                   0.015    0.275    0.056    0.956   -0.525
##     Ethnc_I                  -0.366    0.228   -1.602    0.109   -0.813
##     Ethnc_M                  -0.196    0.216   -0.907    0.365   -0.621
##     Ethnc_O                  -0.852    0.385   -2.213    0.027   -1.606
##     Ethn_PB                  -0.642    0.318   -2.021    0.043   -1.265
##     Income                    0.126    0.038    3.289    0.001    0.051
##     Mtrnl_E                   0.171    0.030    5.623    0.000    0.112
##     Mtrnl_A                   0.055    0.007    7.542    0.000    0.041
##     Mtr_M_H                  -0.424    0.097   -4.380    0.000   -0.613
##     Bt_P__H                   0.216    0.133    1.616    0.106   -0.046
##     Siblngs                   0.050    0.041    1.218    0.223   -0.031
##     Sm__G_A                  -0.320    0.124   -2.586    0.010   -0.563
##  ci.upper   Std.lv  Std.all
##                            
##    -0.001   -0.013   -0.028
##     0.008    0.001    0.004
##     0.012    0.006    0.030
##     0.013   -0.006   -0.008
##                            
##     0.219    0.159    0.079
##     0.035    0.001    0.001
##     0.154    0.128    0.133
##    -0.013   -0.115   -0.035
##                            
##     0.655    0.044    0.036
##     0.644    0.054    0.070
##     0.469    0.040    0.067
##     0.901    0.041    0.020
##                            
##     0.288    0.116    0.094
##     0.110    0.032    0.042
##     0.201    0.088    0.148
##     0.098   -0.030   -0.015
##                            
##     0.111   -0.010   -0.002
##     0.164    0.092    0.032
##     0.071    0.013    0.006
##     0.048   -0.213   -0.021
##     0.067   -0.060   -0.026
##     0.094    0.509    0.137
##     0.351    0.277    0.074
##                            
##     0.110    0.005    0.001
##     0.101    0.032    0.013
##     0.101    0.045    0.023
##     0.567    0.348    0.039
##     0.061   -0.063   -0.031
##     0.023    0.019    0.006
##     0.533    0.656    0.201
##                            
##     0.128    0.001    0.000
##     0.152    0.072    0.024
##     0.133    0.069    0.029
##     0.609    0.330    0.030
##     0.194    0.067    0.027
##     0.009   -0.146   -0.037
##     0.244    0.066    0.017
##                            
##     0.108    0.005    0.001
##     0.070    0.006    0.002
##     0.052   -0.001   -0.000
##     0.357    0.152    0.018
##     0.104   -0.006   -0.003
##    -0.020   -0.359   -0.118
##     0.454    0.526    0.173
##                            
##     0.179    0.045    0.008
##    -0.005   -0.101   -0.028
##     0.040   -0.028   -0.010
##     0.289   -0.052   -0.004
##     0.481    0.321    0.109
##     0.067    0.289    0.060
##    -0.410   -1.147   -0.240
##                            
##     0.069   -0.143   -0.019
##     0.128   -0.072   -0.009
##     0.474    0.208    0.025
##     0.912    0.379    0.022
##     0.957    0.517    0.022
##     0.872    0.489    0.015
##     0.504    0.159    0.010
##     0.668    0.207    0.011
##     0.376   -0.041   -0.002
##     0.716    0.361    0.011
##     1.887    1.188    0.048
##     1.142    0.524    0.019
##     1.443    0.833    0.037
##     2.060    1.057    0.024
##     2.297    1.568    0.078
##     0.082   -0.009   -0.003
##     0.199    0.109    0.040
##     0.055    0.034    0.051
##     0.551    0.318    0.036
##    -0.059   -0.414   -0.034
##     0.017   -0.115   -0.030
##     0.274   -0.015   -0.001
##                            
##     0.284    0.111    0.017
##     0.117   -0.047   -0.007
##     0.137   -0.131   -0.018
##    -0.012   -0.411   -0.027
##     0.608    0.203    0.010
##     0.363   -0.056   -0.002
##     0.050   -0.304   -0.022
##     0.199   -0.170   -0.010
##     0.516    0.075    0.004
##     0.011   -0.266   -0.009
##     1.110    0.438    0.020
##     1.000    0.375    0.016
##    -0.099   -0.612   -0.031
##     1.002    0.326    0.008
##     1.530    0.963    0.055
##     0.094    0.006    0.003
##     0.067   -0.007   -0.003
##    -0.002   -0.020   -0.034
##     0.202   -0.010   -0.001
##     0.341    0.053    0.005
##    -0.070   -0.171   -0.050
##     0.219   -0.027   -0.003
##                            
##     0.014   -0.206   -0.026
##     0.207    0.014    0.002
##     0.027   -0.235   -0.027
##    -0.062   -0.516   -0.028
##     1.111    0.550    0.022
##     0.762    0.334    0.010
##     0.581    0.151    0.009
##     0.452    0.004    0.000
##     0.187   -0.143   -0.007
##     0.109   -0.195   -0.006
##     0.310   -0.507   -0.019
##     1.376    0.650    0.023
##     0.619   -0.107   -0.004
##     0.585   -0.310   -0.007
##     1.275    0.765    0.036
##     0.326    0.217    0.075
##     0.070   -0.018   -0.006
##     0.010   -0.013   -0.019
##    -0.235   -0.501   -0.053
##     0.234   -0.114   -0.009
##     0.094   -0.024   -0.006
##     0.097   -0.212   -0.017
##                            
##    -0.711   -0.881   -0.145
##     0.265    0.109    0.017
##     0.174   -0.034   -0.005
##     0.286   -0.121   -0.009
##     0.475    0.180    0.009
##     0.707    0.349    0.013
##     0.584    0.317    0.025
##     0.682    0.343    0.022
##     0.507    0.213    0.013
##     0.409    0.078    0.003
##     0.039   -0.617   -0.031
##     0.812    0.272    0.012
##     0.125   -0.373   -0.020
##     0.523   -0.223   -0.006
##     0.993    0.387    0.024
##     0.118    0.042    0.019
##     0.129    0.054    0.024
##     0.030    0.011    0.020
##     0.211    0.007    0.001
##     0.204   -0.056   -0.006
##    -0.072   -0.172   -0.055
##     0.272    0.043    0.005
##                            
##    -3.444   -3.669   -0.384
##     0.476    0.230    0.023
##     0.375    0.020    0.002
##     0.935    0.190    0.009
##     0.471   -0.077   -0.003
##     0.494   -0.092   -0.002
##     1.236    0.791    0.039
##     1.000    0.523    0.021
##     0.768    0.353    0.014
##     0.684    0.300    0.007
##    -0.763   -1.817   -0.057
##     0.011   -0.953   -0.027
##     0.158   -0.633   -0.022
##     0.111   -1.381   -0.024
##    -1.100   -2.016   -0.078
##     0.146    0.011    0.003
##     0.177    0.064    0.018
##     0.068    0.038    0.044
##     0.627    0.332    0.029
##     0.533    0.135    0.009
##     0.205    0.055    0.011
##     0.396   -0.011   -0.001
##                            
##    -0.020   -0.038   -0.052
##     0.036    0.008    0.010
##     0.046    0.010    0.006
##     0.113    0.058    0.025
##     0.116    0.060    0.019
##     0.128    0.074    0.048
##     0.103    0.057    0.030
##     0.022   -0.011   -0.005
##     0.064    0.029    0.009
##    -0.043   -0.087   -0.036
##     0.055   -0.010   -0.004
##     0.021   -0.031   -0.014
##    -0.029   -0.094   -0.022
##     0.029   -0.016   -0.008
##     0.013    0.003    0.013
##     0.022    0.015    0.055
##     0.004    0.002    0.035
##     0.018   -0.004   -0.004
##     0.008   -0.023   -0.019
##     0.004   -0.006   -0.015
##     0.001   -0.023   -0.020
##                            
##    -0.338   -0.415   -0.128
##     0.010   -0.093   -0.026
##     0.182   -0.029   -0.004
##     0.113   -0.056   -0.005
##     0.140   -0.024   -0.002
##     0.088   -0.072   -0.011
##     0.017   -0.159   -0.019
##     0.150   -0.039   -0.004
##     0.208    0.054    0.004
##     0.438    0.097    0.009
##     0.316    0.073    0.006
##     0.388    0.145    0.015
##     0.289   -0.229   -0.012
##     0.106   -0.202   -0.023
##     0.079    0.037    0.032
##     0.062    0.025    0.021
##     0.012    0.003    0.010
##     0.068   -0.036   -0.009
##     0.217    0.071    0.014
##    -0.028   -0.077   -0.046
##     0.002   -0.131   -0.026
##                            
##     0.019   -0.063   -0.032
##     0.374   -0.087   -0.039
##     2.571   -0.163   -0.035
##     1.957    0.030    0.005
##     1.813   -0.007   -0.001
##     1.975    0.074    0.018
##    -0.324   -0.227   -0.043
##    -0.592   -0.220   -0.041
##    -0.802   -0.293   -0.033
##    -3.124   -0.912   -0.137
##    -1.327   -0.505   -0.070
##     0.394   -0.178   -0.029
##    -4.224   -1.187   -0.099
##    -3.489   -1.077   -0.200
##     1.152    0.107    0.147
##     1.603    0.169    0.229
##     0.233    0.022    0.124
##     0.315   -0.036   -0.015
##     0.507   -0.044   -0.014
##    -1.159   -0.186   -0.179
##    -0.547   -0.167   -0.054
##                            
##    -0.475   -0.337   -0.168
##     0.102   -0.053   -0.024
##     0.370   -0.047   -0.010
##     0.956    0.343    0.054
##     0.359    0.018    0.002
##     0.609    0.161    0.038
##     0.425    0.027    0.005
##     0.380    0.056    0.010
##     0.279    0.001    0.000
##     0.555    0.009    0.001
##     0.082   -0.206   -0.028
##     0.228   -0.110   -0.018
##    -0.097   -0.479   -0.040
##    -0.019   -0.361   -0.067
##     0.202    0.071    0.097
##     0.231    0.096    0.131
##     0.070    0.031    0.175
##    -0.234   -0.238   -0.101
##     0.477    0.121    0.038
##     0.132    0.028    0.027
##    -0.078   -0.180   -0.058
## 
## Covariances:
##                             Estimate  Std.Err  z-value  P(>|z|) ci.lower
##  .ToM_age5 ~~                                                           
##    .Prosocialty_g5             0.017    0.006    2.607    0.009    0.004
##  .Cognitive_ability_age5 ~~                                             
##    .ToM_age5                   0.290    0.041    7.092    0.000    0.210
##  .Self_regulation_age5 ~~                                               
##    .ToM_age5                   0.030    0.012    2.545    0.011    0.007
##  .Cognitive_ability_age5 ~~                                             
##    .Prosocialty_g5             0.713    0.201    3.548    0.000    0.319
##  .Self_regulation_age5 ~~                                               
##    .Prosocialty_g5             1.537    0.069   22.387    0.000    1.402
##  .Cognitive_ability_age5 ~~                                             
##    .Self_regltn_g5             3.152    0.387    8.148    0.000    2.394
##   Motor_fine ~~                                                         
##     Motor_gross                0.204    0.019   10.599    0.000    0.166
##     Communication              0.360    0.023   15.411    0.000    0.314
##   Motor_gross ~~                                                        
##     Communication              0.706    0.033   21.548    0.000    0.642
##  .OPEN ~~                                                               
##    .CONSC                      1.776    0.173   10.277    0.000    1.437
##    .EXTRAV                     1.906    0.193    9.866    0.000    1.527
##    .AGREE                      2.268    0.165   13.704    0.000    1.944
##    .NEUROT                     0.062    0.221    0.282    0.778   -0.371
##  .CONSC ~~                                                              
##    .EXTRAV                     2.074    0.157   13.241    0.000    1.767
##    .AGREE                      2.820    0.153   18.459    0.000    2.521
##    .NEUROT                    -3.273    0.194  -16.846    0.000   -3.654
##  .EXTRAV ~~                                                             
##    .AGREE                      1.438    0.162    8.867    0.000    1.120
##    .NEUROT                    -5.703    0.219  -26.069    0.000   -6.132
##  .AGREE ~~                                                              
##    .NEUROT                    -0.650    0.170   -3.816    0.000   -0.985
##  ci.upper   Std.lv  Std.all
##                            
##     0.029    0.017    0.030
##                            
##     0.370    0.046    0.126
##                            
##     0.054    0.020    0.054
##                            
##     1.106    0.112    0.071
##                            
##     1.672    0.990    0.630
##                            
##     3.910    0.320    0.320
##                            
##     0.242    0.204    0.193
##     0.406    0.360    0.265
##                            
##     0.770    0.706    0.322
##                            
##     2.114    1.776    0.153
##     2.284    1.906    0.133
##     2.592    2.268    0.212
##     0.496    0.062    0.004
##                            
##     2.381    2.074    0.166
##     3.120    2.820    0.300
##    -2.892   -3.273   -0.236
##                            
##     1.756    1.438    0.124
##    -5.274   -5.703   -0.334
##                            
##    -0.316   -0.650   -0.051
## 
## Intercepts:
##                    Estimate  Std.Err  z-value  P(>|z|) ci.lower ci.upper
##    .Verbal_blty_g5   35.825    3.182   11.258    0.000   29.588   42.062
##    .Spatil_blty_g5   41.381    1.719   24.071    0.000   38.012   44.751
##    .Independenc_g5   11.531    0.846   13.629    0.000    9.873   13.190
##    .Emotion_rgl_g5    7.130    1.235    5.773    0.000    4.709    9.550
##    .ToM_age5          1.165    0.111   10.454    0.000    0.946    1.383
##    .Prosocialty_g5    8.406    0.592   14.196    0.000    7.245    9.567
##    .OPEN             11.929    1.295    9.209    0.000    9.391   14.468
##    .CONSC            13.178    1.092   12.065    0.000   11.037   15.319
##    .EXTRAV           11.924    1.253    9.517    0.000    9.469   14.380
##    .AGREE            14.772    1.067   13.847    0.000   12.681   16.863
##    .NEUROT            9.961    1.437    6.930    0.000    7.144   12.779
##     Motor_fine        7.591    0.013  572.145    0.000    7.565    7.617
##     Motor_gross       5.503    0.017  328.411    0.000    5.470    5.536
##     Communication     6.494    0.026  245.961    0.000    6.442    6.545
##    Std.lv  Std.all
##    35.825    3.385
##    41.381    4.312
##    11.531    3.170
##     7.130    1.513
##     1.165    3.192
##     8.406    5.196
##    11.929    3.203
##    13.178    4.036
##    11.924    3.012
##    14.772    4.861
##     9.961    2.086
##     7.591    9.380
##     5.503    4.218
##     6.494    3.865
## 
## Variances:
##                    Estimate  Std.Err  z-value  P(>|z|) ci.lower ci.upper
##    .ToM_age5          0.131    0.004   37.176    0.000    0.124    0.138
##    .Prosocialty_g5    2.468    0.054   45.935    0.000    2.362    2.573
##    .Cogntv_blty_g5   40.289    3.316   12.148    0.000   33.789   46.789
##    .Self_regltn_g5    2.410    0.165   14.638    0.000    2.087    2.732
##     Motor_fine        0.655    0.026   25.365    0.000    0.604    0.705
##     Motor_gross       1.702    0.040   42.667    0.000    1.623    1.780
##     Communication     2.822    0.050   56.288    0.000    2.724    2.921
##    .Verbal_blty_g5   47.694    3.263   14.616    0.000   41.299   54.090
##    .Spatil_blty_g5   75.680    2.406   31.457    0.000   70.965   80.396
##    .Independenc_g5   10.075    0.233   43.150    0.000    9.617   10.532
##    .Emotion_rgl_g5   15.642    0.384   40.728    0.000   14.889   16.395
##    .OPEN             13.251    0.222   59.659    0.000   12.816   13.687
##    .CONSC            10.201    0.202   50.415    0.000    9.804   10.597
##    .EXTRAV           15.390    0.243   63.369    0.000   14.914   15.866
##    .AGREE             8.677    0.210   41.369    0.000    8.266    9.088
##    .NEUROT           18.904    0.290   65.127    0.000   18.336   19.473
##    Std.lv  Std.all
##     0.131    0.983
##     2.468    0.943
##     0.626    0.626
##     0.762    0.762
##     0.655    1.000
##     1.702    1.000
##     2.822    1.000
##    47.694    0.426
##    75.680    0.822
##    10.075    0.761
##    15.642    0.704
##    13.251    0.955
##    10.201    0.957
##    15.390    0.982
##     8.677    0.939
##    18.904    0.829
## 
## Defined Parameters:
##                    Estimate  Std.Err  z-value  P(>|z|) ci.lower ci.upper
##     fT_T_O            0.003    0.002    1.357    0.175   -0.001    0.007
##     gT_T_O           -0.000    0.001   -0.333    0.739   -0.002    0.001
##     cT_T_O           -0.001    0.001   -1.290    0.197   -0.003    0.001
##     fP_P_O           -0.009    0.010   -0.910    0.363   -0.030    0.011
##     gP_P_O           -0.000    0.001   -0.079    0.937   -0.002    0.002
##     cP_P_O           -0.008    0.008   -0.914    0.361   -0.024    0.009
##     fC_C_O            0.022    0.011    2.048    0.041    0.001    0.044
##     gC_C_O            0.027    0.009    3.049    0.002    0.010    0.045
##     cC_C_O            0.020    0.007    2.875    0.004    0.006    0.034
##     fS_S_O            0.032    0.022    1.481    0.139   -0.010    0.075
##     gS_S_O            0.009    0.007    1.221    0.222   -0.005    0.023
##     cS_S_O            0.024    0.016    1.561    0.119   -0.006    0.055
##     total_fO          0.038    0.061    0.618    0.536   -0.082    0.158
##     total_gO          0.128    0.036    3.519    0.000    0.057    0.199
##     total_cO          0.048    0.029    1.652    0.099   -0.009    0.106
##     fT_T_C           -0.004    0.003   -1.685    0.092   -0.010    0.001
##     gT_T_C            0.000    0.001    0.334    0.738   -0.002    0.003
##     cT_T_C            0.002    0.001    1.940    0.052   -0.000    0.004
##     fP_P_C           -0.010    0.011   -0.945    0.345   -0.031    0.011
##     gP_P_C           -0.000    0.001   -0.079    0.937   -0.002    0.002
##     cP_P_C           -0.008    0.008   -0.977    0.329   -0.024    0.008
##     fC_C_C            0.001    0.004    0.222    0.824   -0.006    0.008
##     gC_C_C            0.001    0.005    0.222    0.824   -0.008    0.010
##     cC_C_C            0.001    0.003    0.223    0.824   -0.006    0.007
##     fS_S_C            0.076    0.024    3.188    0.001    0.029    0.123
##     gS_S_C            0.021    0.011    1.923    0.054   -0.000    0.043
##     cS_S_C            0.058    0.015    3.867    0.000    0.029    0.087
##     total_fC          0.068    0.052    1.301    0.193   -0.034    0.170
##     total_gC          0.055    0.034    1.626    0.104   -0.011    0.121
##     total_cC          0.098    0.027    3.583    0.000    0.044    0.152
##     fT_T_E           -0.004    0.003   -1.603    0.109   -0.009    0.001
##     gT_T_E            0.000    0.001    0.326    0.745   -0.002    0.003
##     cT_T_E            0.002    0.001    1.703    0.089   -0.000    0.005
##     fP_P_E            0.011    0.010    1.008    0.313   -0.010    0.031
##     gP_P_E            0.000    0.001    0.079    0.937   -0.002    0.002
##     cP_P_E            0.009    0.008    1.025    0.305   -0.008    0.025
##     fC_C_E           -0.006    0.006   -1.099    0.272   -0.018    0.005
##     gC_C_E           -0.008    0.006   -1.290    0.197   -0.020    0.004
##     cC_C_E           -0.006    0.005   -1.262    0.207   -0.015    0.003
##     fS_S_E            0.008    0.022    0.346    0.729   -0.036    0.051
##     gS_S_E            0.002    0.006    0.345    0.730   -0.010    0.014
##     cS_S_E            0.006    0.017    0.349    0.727   -0.027    0.038
##     total_fE          0.009    0.063    0.140    0.889   -0.115    0.133
##     total_gE          0.067    0.041    1.633    0.102   -0.013    0.147
##     total_cE          0.080    0.032    2.475    0.013    0.017    0.143
##     fT_T_A           -0.002    0.002   -1.144    0.253   -0.005    0.001
##     gT_T_A            0.000    0.001    0.328    0.743   -0.001    0.001
##     cT_T_A            0.001    0.001    1.354    0.176   -0.000    0.002
##     fP_P_A           -0.001    0.009   -0.101    0.919   -0.018    0.017
##     gP_P_A           -0.000    0.000   -0.066    0.948   -0.000    0.000
##     cP_P_A           -0.001    0.007   -0.101    0.919   -0.015    0.013
##     fC_C_A           -0.016    0.008   -1.895    0.058   -0.032    0.001
##     gC_C_A           -0.019    0.007   -2.813    0.005   -0.033   -0.006
##     cC_C_A           -0.014    0.005   -2.591    0.010   -0.025   -0.003
##     fS_S_A            0.061    0.022    2.826    0.005    0.019    0.104
##     gS_S_A            0.017    0.009    1.908    0.056   -0.000    0.035
##     cS_S_A            0.046    0.015    3.194    0.001    0.018    0.075
##     total_fA          0.047    0.050    0.944    0.345   -0.051    0.145
##     total_gA          0.004    0.032    0.111    0.912   -0.059    0.066
##     total_cA          0.032    0.026    1.208    0.227   -0.020    0.083
##     fT_T_N            0.001    0.002    0.293    0.770   -0.004    0.005
##     gT_T_N           -0.000    0.000   -0.243    0.808   -0.001    0.000
##     cT_T_N           -0.000    0.001   -0.293    0.770   -0.003    0.002
##     fP_P_N            0.051    0.017    3.079    0.002    0.018    0.083
##     gP_P_N            0.000    0.006    0.079    0.937   -0.010    0.011
##     cP_P_N            0.041    0.012    3.530    0.000    0.018    0.064
##     fC_C_N            0.013    0.008    1.678    0.093   -0.002    0.028
##     gC_C_N            0.016    0.008    1.898    0.058   -0.001    0.032
##     cC_C_N            0.011    0.006    2.036    0.042    0.000    0.023
##     fS_S_N           -0.133    0.038   -3.476    0.001   -0.209   -0.058
##     gS_S_N           -0.037    0.019   -1.964    0.050   -0.074   -0.000
##     cS_S_N           -0.101    0.024   -4.293    0.000   -0.147   -0.055
##     total_fN         -0.024    0.069   -0.349    0.727   -0.159    0.111
##     total_gN         -0.123    0.045   -2.745    0.006   -0.211   -0.035
##     total_cN         -0.077    0.035   -2.189    0.029   -0.146   -0.008
##    Std.lv  Std.all
##     0.003    0.001
##    -0.000   -0.000
##    -0.001   -0.001
##    -0.009   -0.002
##    -0.000   -0.000
##    -0.008   -0.003
##     0.022    0.005
##     0.027    0.010
##     0.020    0.009
##     0.032    0.007
##     0.009    0.003
##     0.024    0.011
##     0.038    0.008
##     0.128    0.045
##     0.048    0.022
##    -0.004   -0.001
##     0.000    0.000
##     0.002    0.001
##    -0.010   -0.002
##    -0.000   -0.000
##    -0.008   -0.004
##     0.001    0.000
##     0.001    0.000
##     0.001    0.000
##     0.076    0.019
##     0.021    0.009
##     0.058    0.030
##     0.068    0.017
##     0.055    0.022
##     0.098    0.051
##    -0.004   -0.001
##     0.000    0.000
##     0.002    0.001
##     0.011    0.002
##     0.000    0.000
##     0.009    0.004
##    -0.006   -0.001
##    -0.008   -0.003
##    -0.006   -0.002
##     0.008    0.002
##     0.002    0.001
##     0.006    0.002
##     0.009    0.002
##     0.067    0.022
##     0.080    0.034
##    -0.002   -0.001
##     0.000    0.000
##     0.001    0.001
##    -0.001   -0.000
##    -0.000   -0.000
##    -0.001   -0.000
##    -0.016   -0.004
##    -0.019   -0.008
##    -0.014   -0.008
##     0.061    0.016
##     0.017    0.007
##     0.046    0.026
##     0.047    0.013
##     0.004    0.002
##     0.032    0.018
##     0.001    0.000
##    -0.000   -0.000
##    -0.000   -0.000
##     0.051    0.009
##     0.000    0.000
##     0.041    0.014
##     0.013    0.002
##     0.016    0.004
##     0.011    0.004
##    -0.133   -0.023
##    -0.037   -0.010
##    -0.101   -0.036
##    -0.024   -0.004
##    -0.123   -0.034
##    -0.077   -0.027
```

## Results table (unimputed)

|  |  |  |  |
| --- | --- | --- | --- |
|  | Model 1 | Model 2 | Model 3 |
|  | Estimate (Std. Err.) | Estimate (Std. Err.) | Estimate (Std. Err.) |
|  | Regression Slopes | | |
| Theory of mind (age 5) |
| Motor.fine | -0.01(0.01) | -0.01(0.01) | -0.01(0.01)\* |
| Motor.gross | -0.00(0.00) | 0.00(0.00) | 0.00(0.00) |
| Communication | 0.01(0.00)\* | 0.01(0.00)\* | 0.01(0.00)\* |
| Cohort member’s age (in months) | -0.00(0.01) | -0.00(0.01) | -0.01(0.01) |
| Sex: Male |  | -0.04(0.01)\*\*\* | -0.04(0.01)\*\*\* |
| England - Disadvantaged |  | 0.00(0.01) | 0.01(0.01) |
| England - Ethnic |  | -0.02(0.01) | 0.01(0.02) |
| Northern Ireland - Advantaged |  | 0.07(0.03)\* | 0.06(0.03)\* |
| Northern Ireland - Disadvantaged |  | 0.06(0.03)\* | 0.06(0.03)\* |
| Scotland - Advantaged |  | 0.08(0.03)\*\* | 0.07(0.03)\*\* |
| Scotland - Disadvantaged |  | 0.06(0.02)\*\* | 0.06(0.02)\* |
| Wales - Advantaged |  | -0.01(0.02) | -0.01(0.02) |
| Wales - Disadvantaged |  | 0.02(0.02) | 0.03(0.02) |
| Income |  | 0.01(0.00)\*\*\* | 0.00(0.00) |
| Black or Black British |  |  | -0.09(0.02)\*\*\* |
| Indian |  |  | -0.01(0.03) |
| Mixed |  |  | -0.03(0.03) |
| Other ethnic group |  |  | -0.09(0.03)\*\* |
| Pakistani & Bangladeshi |  |  | -0.02(0.02) |
| Maternal education |  |  | 0.01(0.00)\*\*\* |
| Maternal.Age |  |  | 0.00(0.00)\* |
| Maternal mental health |  |  | -0.00(0.01) |
| Both.Parents.in.Household |  |  | -0.02(0.02) |
| Siblings |  |  | -0.01(0.00) |
| Small.for.Gestational.Age |  |  | -0.02(0.01) |
| Prosociality (age 5) |
| Motor.fine | 0.19(0.03)\*\*\* | 0.16(0.03)\*\*\* | 0.16(0.03)\*\*\* |
| Motor.gross | -0.02(0.02) | 0.00(0.02) | 0.00(0.02) |
| Communication | 0.14(0.01)\*\*\* | 0.13(0.01)\*\*\* | 0.13(0.01)\*\*\* |
| Cohort member’s age (in months) | -0.10(0.05)\* | -0.11(0.05)\* | -0.12(0.05)\* |
| Sex: Male |  | -0.40(0.04)\*\*\* | -0.41(0.04)\*\*\* |
| England - Disadvantaged |  | -0.10(0.05)\* | -0.09(0.05) |
| England - Ethnic |  | -0.17(0.07)\* | -0.03(0.11) |
| Northern Ireland - Advantaged |  | -0.06(0.08) | -0.06(0.09) |
| Northern Ireland - Disadvantaged |  | -0.06(0.08) | -0.02(0.08) |
| Scotland - Advantaged |  | -0.06(0.08) | -0.07(0.08) |
| Scotland - Disadvantaged |  | -0.17(0.09)\* | -0.16(0.09) |
| Wales - Advantaged |  | -0.02(0.09) | -0.04(0.10) |
| Wales - Disadvantaged |  | 0.05(0.07) | 0.05(0.08) |
| Income |  | 0.08(0.02)\*\*\* | 0.04(0.02) |
| Black or Black British |  |  | 0.10(0.17) |
| Indian |  |  | 0.07(0.12) |
| Mixed |  |  | 0.15(0.12) |
| Other ethnic group |  |  | -0.23(0.26) |
| Pakistani & Bangladeshi |  |  | -0.20(0.16) |
| Maternal education |  |  | 0.03(0.02) |
| Maternal.Age |  |  | 0.00(0.00) |
| Maternal mental health |  |  | -0.04(0.05) |
| Both.Parents.in.Household |  |  | 0.07(0.07) |
| Siblings |  |  | -0.08(0.02)\*\* |
| Small.for.Gestational.Age |  |  | -0.13(0.07) |
| Cognitive\_ability\_age5 |
| Motor.fine | 0.72(0.16)\*\*\* | 0.44(0.16)\*\* | 0.35(0.15)\* |
| Motor.gross | 0.34(0.10)\*\*\* | 0.43(0.11)\*\*\* | 0.43(0.11)\*\*\* |
| Communication | 0.04(0.08) | 0.24(0.08)\*\* | 0.32(0.08)\*\*\* |
| Cohort member’s age (in months) | 0.64(0.26)\* | 0.44(0.29) | 0.33(0.29) |
| Sex: Male |  | -0.64(0.27)\* | -0.51(0.27) |
| England - Disadvantaged |  | -1.49(0.55)\*\* | -0.70(0.55) |
| England - Ethnic |  | -7.37(2.04)\*\*\* | -1.31(1.98) |
| Northern Ireland - Advantaged |  | 0.81(0.86) | 0.24(0.88) |
| Northern Ireland - Disadvantaged |  | 0.13(0.96) | -0.06(0.95) |
| Scotland - Advantaged |  | 1.20(0.72) | 0.59(0.71) |
| Scotland - Disadvantaged |  | -1.25(0.73) | -1.82(0.76)\* |
| Wales - Advantaged |  | -1.09(0.60) | -1.77(0.60)\*\* |
| Wales - Disadvantaged |  | -2.51(0.76)\*\* | -2.35(0.79)\*\* |
| Income |  | 2.15(0.14)\*\*\* | 0.86(0.15)\*\*\* |
| Black or Black British |  |  | -7.32(2.14)\*\*\* |
| Indian |  |  | -4.05(1.39)\*\* |
| Mixed |  |  | -1.43(0.93) |
| Other ethnic group |  |  | -9.52(2.70)\*\*\* |
| Pakistani & Bangladeshi |  |  | -8.64(2.63)\*\* |
| Maternal education |  |  | 1.35(0.13)\*\*\* |
| Maternal.Age |  |  | 0.18(0.03)\*\*\* |
| Maternal mental health |  |  | -0.29(0.31) |
| Both.Parents.in.Household |  |  | -0.36(0.44) |
| Siblings |  |  | -1.49(0.17)\*\*\* |
| Small.for.Gestational.Age |  |  | -1.34(0.41)\*\*\* |
| Self\_regulation\_age5 |
| Motor.fine | 0.33(0.05)\*\*\* | 0.26(0.04)\*\*\* | 0.21(0.04)\*\*\* |
| Motor.gross | 0.04(0.03) | 0.06(0.03)\* | 0.06(0.03)\* |
| Communication | 0.16(0.03)\*\*\* | 0.15(0.02)\*\*\* | 0.16(0.02)\*\*\* |
| Cohort member’s age (in months) | -0.04(0.08) | -0.08(0.08) | -0.05(0.08) |
| Sex: Male |  | -0.65(0.07)\*\*\* | -0.60(0.06)\*\*\* |
| England - Disadvantaged |  | -0.20(0.10) | -0.09(0.10) |
| England - Ethnic |  | -0.52(0.19)\*\* | -0.08(0.23) |
| Northern Ireland - Advantaged |  | 0.82(0.18)\*\*\* | 0.61(0.18)\*\*\* |
| Northern Ireland - Disadvantaged |  | 0.03(0.18) | 0.03(0.17) |
| Scotland - Advantaged |  | 0.39(0.18)\* | 0.29(0.16) |
| Scotland - Disadvantaged |  | 0.00(0.20) | 0.05(0.19) |
| Wales - Advantaged |  | 0.19(0.16) | 0.10(0.14) |
| Wales - Disadvantaged |  | -0.11(0.14) | 0.00(0.14) |
| Income |  | 0.34(0.03)\*\*\* | 0.13(0.04)\*\* |
| Black or Black British |  |  | 0.02(0.28) |
| Indian |  |  | -0.37(0.23) |
| Mixed |  |  | -0.20(0.22) |
| Other ethnic group |  |  | -0.85(0.38)\* |
| Pakistani & Bangladeshi |  |  | -0.64(0.32)\* |
| Maternal education |  |  | 0.17(0.03)\*\*\* |
| Maternal.Age |  |  | 0.06(0.01)\*\*\* |
| Maternal mental health |  |  | -0.42(0.10)\*\*\* |
| Both.Parents.in.Household |  |  | 0.22(0.13) |
| Siblings |  |  | 0.05(0.04) |
| Small.for.Gestational.Age |  |  | -0.32(0.12)\*\* |
| OPEN |
| Motor.fine | 0.00(0.06) | 0.00(0.06) | -0.01(0.06) |
| Motor.gross | 0.08(0.04)\* | 0.09(0.04)\* | 0.09(0.04)\* |
| Communication | 0.01(0.03) | -0.00(0.03) | 0.01(0.03) |
| Theory of mind (age 5) | -0.21(0.13) | -0.23(0.13) | -0.21(0.13) |
| Prosociality (age 5) | -0.06(0.07) | -0.07(0.07) | -0.06(0.06) |
| Cognitive.ability.age5 | 0.07(0.02)\*\*\* | 0.07(0.02)\*\*\* | 0.06(0.02)\*\*\* |
| Self.regulation.age5 | 0.14(0.10) | 0.17(0.10) | 0.16(0.10) |
| Sex: Male |  | -0.14(0.11) | -0.14(0.11) |
| Cohort member’s age (in months) |  | -0.05(0.10) | -0.07(0.10) |
| England - Disadvantaged |  | 0.32(0.13)\* | 0.21(0.14) |
| England - Ethnic |  | 1.24(0.29)\*\*\* | 0.38(0.27) |
| Northern Ireland - Advantaged |  | 0.42(0.23) | 0.52(0.22)\* |
| Northern Ireland - Disadvantaged |  | 0.31(0.19) | 0.49(0.20)\* |
| Scotland - Advantaged |  | 0.18(0.18) | 0.16(0.18) |
| Scotland - Disadvantaged |  | 0.17(0.24) | 0.21(0.24) |
| Wales - Advantaged |  | -0.07(0.21) | -0.04(0.21) |
| Wales - Disadvantaged |  | 0.30(0.19) | 0.36(0.18)\* |
| Income |  | 0.03(0.04) | -0.01(0.05) |
| Black or Black British |  |  | 1.19(0.36)\*\*\* |
| Indian |  |  | 0.52(0.32) |
| Mixed |  |  | 0.83(0.31)\*\* |
| Other ethnic group |  |  | 1.06(0.51)\* |
| Pakistani & Bangladeshi |  |  | 1.57(0.37)\*\*\* |
| Maternal education |  |  | 0.11(0.05)\* |
| Maternal.Age |  |  | 0.03(0.01)\*\* |
| Maternal mental health |  |  | 0.32(0.12)\*\* |
| Both.Parents.in.Household |  |  | -0.41(0.18)\* |
| Siblings |  |  | -0.12(0.07) |
| Small.for.Gestational.Age |  |  | -0.01(0.15) |
| CONSC |
| Motor.fine | 0.00(0.05) | 0.00(0.05) | 0.01(0.05) |
| Motor.gross | 0.03(0.03) | 0.02(0.03) | 0.03(0.04) |
| Communication | 0.04(0.03) | 0.05(0.03) | 0.05(0.03) |
| Theory of mind (age 5) | 0.31(0.11)\*\* | 0.33(0.11)\*\* | 0.35(0.11)\*\* |
| Prosociality (age 5) | -0.05(0.06) | -0.06(0.06) | -0.06(0.06) |
| Cognitive.ability.age5 | 0.01(0.02) | 0.00(0.01) | 0.00(0.01) |
| Self.regulation.age5 | 0.30(0.08)\*\*\* | 0.33(0.08)\*\*\* | 0.37(0.08)\*\*\* |
| Sex: Male |  | 0.12(0.09) | 0.11(0.09) |
| Cohort member’s age (in months) |  | -0.04(0.08) | -0.05(0.08) |
| England - Disadvantaged |  | -0.06(0.13) | -0.13(0.14) |
| England - Ethnic |  | -0.01(0.16) | -0.41(0.20)\* |
| Northern Ireland - Advantaged |  | 0.14(0.20) | 0.20(0.21) |
| Northern Ireland - Disadvantaged |  | -0.14(0.21) | -0.06(0.21) |
| Scotland - Advantaged |  | -0.30(0.18) | -0.30(0.18) |
| Scotland - Disadvantaged |  | -0.11(0.19) | -0.17(0.19) |
| Wales - Advantaged |  | 0.06(0.22) | 0.08(0.22) |
| Wales - Disadvantaged |  | -0.24(0.14) | -0.27(0.14) |
| Income |  | 0.00(0.04) | 0.01(0.05) |
| Black or Black British |  |  | 0.44(0.34) |
| Indian |  |  | 0.37(0.32) |
| Mixed |  |  | -0.61(0.26)\* |
| Other ethnic group |  |  | 0.33(0.34) |
| Pakistani & Bangladeshi |  |  | 0.96(0.29)\*\*\* |
| Maternal education |  |  | -0.01(0.04) |
| Maternal.Age |  |  | -0.02(0.01)\* |
| Maternal mental health |  |  | -0.01(0.11) |
| Both.Parents.in.Household |  |  | 0.05(0.15) |
| Siblings |  |  | -0.17(0.05)\*\*\* |
| Small.for.Gestational.Age |  |  | -0.03(0.13) |
| EXTRAV |
| Motor.fine | 0.03(0.06) | 0.01(0.06) | 0.00(0.06) |
| Motor.gross | 0.04(0.04) | 0.06(0.04) | 0.07(0.04) |
| Communication | 0.06(0.03) | 0.08(0.03)\* | 0.07(0.03)\* |
| Theory of mind (age 5) | 0.38(0.14)\*\* | 0.36(0.14)\* | 0.33(0.14)\* |
| Prosociality (age 5) | 0.02(0.07) | 0.06(0.07) | 0.07(0.06) |
| Cognitive.ability.age5 | -0.02(0.02) | -0.02(0.02) | -0.02(0.01) |
| Self.regulation.age5 | 0.13(0.10) | 0.06(0.10) | 0.04(0.11) |
| Sex: Male |  | -0.20(0.11) | -0.21(0.11) |
| Cohort member’s age (in months) |  | 0.01(0.10) | 0.01(0.10) |
| England - Disadvantaged |  | -0.19(0.13) | -0.23(0.13) |
| England - Ethnic |  | -0.18(0.19) | -0.52(0.23)\* |
| Northern Ireland - Advantaged |  | 0.53(0.28) | 0.55(0.29) |
| Northern Ireland - Disadvantaged |  | 0.33(0.21) | 0.33(0.22) |
| Scotland - Advantaged |  | 0.15(0.22) | 0.15(0.22) |
| Scotland - Disadvantaged |  | 0.03(0.23) | 0.00(0.23) |
| Wales - Advantaged |  | -0.13(0.17) | -0.14(0.17) |
| Wales - Disadvantaged |  | -0.17(0.16) | -0.20(0.16) |
| Income |  | 0.20(0.04)\*\*\* | 0.22(0.06)\*\*\* |
| Black or Black British |  |  | -0.51(0.42) |
| Indian |  |  | 0.65(0.37) |
| Mixed |  |  | -0.11(0.37) |
| Other ethnic group |  |  | -0.31(0.46) |
| Pakistani & Bangladeshi |  |  | 0.76(0.26)\*\* |
| Maternal education |  |  | -0.02(0.04) |
| Maternal.Age |  |  | -0.01(0.01) |
| Maternal mental health |  |  | -0.50(0.14)\*\*\* |
| Both.Parents.in.Household |  |  | -0.11(0.18) |
| Siblings |  |  | -0.02(0.06) |
| Small.for.Gestational.Age |  |  | -0.21(0.16) |
| AGREE |
| Motor.fine | 0.04(0.05) | 0.01(0.05) | 0.00(0.05) |
| Motor.gross | -0.04(0.03) | -0.01(0.03) | 0.01(0.03) |
| Communication | 0.03(0.03) | -0.00(0.03) | -0.00(0.03) |
| Theory of mind (age 5) | 0.21(0.11) | 0.14(0.11) | 0.15(0.10) |
| Prosociality (age 5) | -0.03(0.06) | -0.02(0.06) | -0.01(0.06) |
| Cognitive.ability.age5 | -0.05(0.02)\*\* | -0.04(0.01)\*\*\* | -0.04(0.01)\*\*\* |
| Self.regulation.age5 | 0.33(0.08)\*\*\* | 0.29(0.08)\*\*\* | 0.30(0.08)\*\*\* |
| Sex: Male |  | -0.86(0.09)\*\*\* | -0.88(0.09)\*\*\* |
| Cohort member’s age (in months) |  | 0.12(0.08) | 0.11(0.08) |
| England - Disadvantaged |  | -0.01(0.11) | -0.03(0.11) |
| England - Ethnic |  | -0.12(0.15) | -0.12(0.21) |
| Northern Ireland - Advantaged |  | 0.17(0.15) | 0.18(0.15) |
| Northern Ireland - Disadvantaged |  | 0.36(0.17)\* | 0.35(0.18) |
| Scotland - Advantaged |  | 0.35(0.13)\* | 0.32(0.14)\* |
| Scotland - Disadvantaged |  | 0.40(0.17)\* | 0.34(0.17)\* |
| Wales - Advantaged |  | 0.24(0.15) | 0.21(0.15) |
| Wales - Disadvantaged |  | 0.08(0.16) | 0.08(0.17) |
| Income |  | 0.10(0.04)\*\* | 0.04(0.04) |
| Black or Black British |  |  | -0.62(0.33) |
| Indian |  |  | 0.27(0.28) |
| Mixed |  |  | -0.37(0.25) |
| Other ethnic group |  |  | -0.22(0.38) |
| Pakistani & Bangladeshi |  |  | 0.39(0.31) |
| Maternal education |  |  | 0.05(0.04) |
| Maternal.Age |  |  | 0.01(0.01) |
| Maternal mental health |  |  | 0.01(0.10) |
| Both.Parents.in.Household |  |  | -0.06(0.13) |
| Siblings |  |  | -0.17(0.05)\*\*\* |
| Small.for.Gestational.Age |  |  | 0.04(0.12) |
| NEUROT |
| Motor.fine | 0.14(0.07)\* | 0.08(0.07) | 0.04(0.07) |
| Motor.gross | -0.25(0.05)\*\*\* | -0.12(0.05)\* | -0.10(0.05)\* |
| Communication | 0.11(0.04)\*\* | -0.04(0.03) | -0.03(0.03) |
| Theory of mind (age 5) | 0.15(0.18) | -0.04(0.18) | -0.05(0.17) |
| Prosociality (age 5) | 0.36(0.09)\*\*\* | 0.34(0.08)\*\*\* | 0.32(0.08)\*\*\* |
| Cognitive.ability.age5 | 0.09(0.02)\*\*\* | 0.06(0.02)\*\*\* | 0.04(0.02)\* |
| Self.regulation.age5 | -0.47(0.12)\*\*\* | -0.65(0.12)\*\*\* | -0.65(0.12)\*\*\* |
| Sex: Male |  | -3.68(0.12)\*\*\* | -3.67(0.12)\*\*\* |
| Cohort member’s age (in months) |  | 0.20(0.12) | 0.23(0.13) |
| England - Disadvantaged |  | -0.13(0.18) | 0.02(0.18) |
| England - Ethnic |  | -0.97(0.27)\*\*\* | 0.19(0.38) |
| Northern Ireland - Advantaged |  | 0.07(0.28) | -0.08(0.28) |
| Northern Ireland - Disadvantaged |  | 0.00(0.29) | -0.09(0.30) |
| Scotland - Advantaged |  | 0.82(0.23)\*\*\* | 0.79(0.23)\*\*\* |
| Scotland - Disadvantaged |  | 0.58(0.23)\* | 0.52(0.24)\* |
| Wales - Advantaged |  | 0.45(0.21)\* | 0.35(0.21) |
| Wales - Disadvantaged |  | 0.26(0.20) | 0.30(0.20) |
| Income |  | 0.07(0.06) | 0.01(0.07) |
| Black or Black British |  |  | -1.82(0.54)\*\*\* |
| Indian |  |  | -0.95(0.49) |
| Mixed |  |  | -0.63(0.40) |
| Other ethnic group |  |  | -1.38(0.76) |
| Pakistani & Bangladeshi |  |  | -2.02(0.47)\*\*\* |
| Maternal education |  |  | 0.06(0.06) |
| Maternal.Age |  |  | 0.04(0.02)\* |
| Maternal mental health |  |  | 0.33(0.15)\* |
| Both.Parents.in.Household |  |  | 0.14(0.20) |
| Siblings |  |  | 0.06(0.08) |
| Small.for.Gestational.Age |  |  | -0.01(0.21) |
|  | Intercepts | | |
| Verbal ability (age 5) | 42.43(2.78)\*\*\* | 38.84(3.02)\*\*\* | 35.82(3.18)\*\*\* |
| Spatial ability (age 5) | 40.50(2.36)\*\*\* | 41.81(1.83)\*\*\* | 41.38(1.72)\*\*\* |
| Independence skills (age 5) | 13.03(0.87)\*\*\* | 13.16(0.87)\*\*\* | 11.53(0.85)\*\*\* |
| Emotion regulation (age 5) | 10.22(1.02)\*\*\* | 9.96(1.12)\*\*\* | 7.13(1.23)\*\*\* |
| Theory of mind (age 5) | 1.21(0.11)\*\*\* | 1.20(0.11)\*\*\* | 1.16(0.11)\*\*\* |
| Prosociality (age 5) | 8.10(0.57)\*\*\* | 8.39(0.57)\*\*\* | 8.41(0.59)\*\*\* |
| OPEN | 12.99(0.83)\*\*\* | 13.23(1.25)\*\*\* | 11.93(1.30)\*\*\* |
| CONSC | 12.84(0.71)\*\*\* | 13.11(1.05)\*\*\* | 13.18(1.09)\*\*\* |
| EXTRAV | 11.80(0.86)\*\*\* | 11.23(1.24)\*\*\* | 11.92(1.25)\*\*\* |
| AGREE | 15.84(0.75)\*\*\* | 15.32(1.01)\*\*\* | 14.77(1.07)\*\*\* |
| NEUROT | 8.38(0.96)\*\*\* | 10.12(1.41)\*\*\* | 9.96(1.44)\*\*\* |
| Motor.fine | 7.59(0.01)\*\*\* | 7.59(0.01)\*\*\* | 7.59(0.01)\*\*\* |
| Motor.gross | 5.50(0.02)\*\*\* | 5.50(0.02)\*\*\* | 5.50(0.02)\*\*\* |
| Communication | 6.50(0.03)\*\*\* | 6.50(0.03)\*\*\* | 6.49(0.03)\*\*\* |
| Cohort member’s age (in months) | 9.17+ | 9.17+ | 9.17+ |
| Sex: Male |  | 0.48+ | 0.48+ |
| England - Disadvantaged |  | 0.28+ | 0.28+ |
| England - Ethnic |  | 0.05+ | 0.05+ |
| Northern Ireland - Advantaged |  | 0.03+ | 0.03+ |
| Northern Ireland - Disadvantaged |  | 0.01+ | 0.01+ |
| Scotland - Advantaged |  | 0.06+ | 0.06+ |
| Scotland - Disadvantaged |  | 0.04+ | 0.04+ |
| Wales - Advantaged |  | 0.03+ | 0.04+ |
| Wales - Disadvantaged |  | 0.01+ | 0.01+ |
| Income |  | 3.23+ | 3.26+ |
| Black or Black British |  |  | 0.02+ |
| Indian |  |  | 0.02+ |
| Mixed |  |  | 0.03+ |
| Other ethnic group |  |  | 0.01+ |
| Pakistani & Bangladeshi |  |  | 0.04+ |
| Maternal education |  |  | 3.76+ |
| Maternal.Age |  |  | 30.33+ |
| Maternal mental health |  |  | 0.23+ |
| Both.Parents.in.Household |  |  | 0.89+ |
| Siblings |  |  | 0.86+ |
| Small.for.Gestational.Age |  |  | 0.12+ |
|  | Fit Indices | | |
| χ2 | 571.76 | 1345.77 | 1797.69 |
| CFI | 0.94 | 0.91 | 0.89 |
| TLI | 0.81 | 0.70 | 0.66 |
| RMSEA | 0.05 | 0.04 | 0.04 |
| Scaled χ2 | 449.66(31)\*\*\* | 1116.42(76)\*\*\* | 1433.10(131)\*\*\* |
| +Fixed parameter | | | |
| \*p<0.05, \*\*p<0.01, \*\*\*p<0.001 | | | |

|  |  |  |  |
| --- | --- | --- | --- |
|  | Model 1 | Model 2 | Model 3 |
|  | Estimate (Std. Err.) | Estimate (Std. Err.) | Estimate (Std. Err.) |
|  | Regression Slopes | | |
| Theory of mind (age 5) |
| Motor.fine | -0.01(0.01) | -0.01(0.01) | -0.01(0.01)\* |
| Motor.gross | -0.00(0.00) | 0.00(0.00) | 0.00(0.00) |
| Communication | 0.01(0.00)\* | 0.01(0.00)\* | 0.01(0.00)\* |
| Cohort member’s age (in months) | -0.00(0.01) | -0.00(0.01) | -0.01(0.01) |
| Sex: Male |  | -0.04(0.01)\*\*\* | -0.04(0.01)\*\*\* |
| England - Disadvantaged |  | 0.00(0.01) | 0.01(0.01) |
| England - Ethnic |  | -0.02(0.01) | 0.01(0.02) |
| Northern Ireland - Advantaged |  | 0.07(0.03)\* | 0.06(0.03)\* |
| Northern Ireland - Disadvantaged |  | 0.06(0.03)\* | 0.06(0.03)\* |
| Scotland - Advantaged |  | 0.08(0.03)\*\* | 0.07(0.03)\*\* |
| Scotland - Disadvantaged |  | 0.06(0.02)\*\* | 0.06(0.02)\* |
| Wales - Advantaged |  | -0.01(0.02) | -0.01(0.02) |
| Wales - Disadvantaged |  | 0.02(0.02) | 0.03(0.02) |
| Income |  | 0.01(0.00)\*\*\* | 0.00(0.00) |
| Black or Black British |  |  | -0.09(0.02)\*\*\* |
| Indian |  |  | -0.01(0.03) |
| Mixed |  |  | -0.03(0.03) |
| Other ethnic group |  |  | -0.09(0.03)\*\* |
| Pakistani & Bangladeshi |  |  | -0.02(0.02) |
| Maternal education |  |  | 0.01(0.00)\*\*\* |
| Maternal.Age |  |  | 0.00(0.00)\* |
| Maternal mental health |  |  | -0.00(0.01) |
| Both.Parents.in.Household |  |  | -0.02(0.02) |
| Siblings |  |  | -0.01(0.00) |
| Small.for.Gestational.Age |  |  | -0.02(0.01) |
| Prosociality (age 5) |
| Motor.fine | 0.19(0.03)\*\*\* | 0.16(0.03)\*\*\* | 0.16(0.03)\*\*\* |
| Motor.gross | -0.02(0.02) | 0.00(0.02) | 0.00(0.02) |
| Communication | 0.14(0.01)\*\*\* | 0.13(0.01)\*\*\* | 0.13(0.01)\*\*\* |
| Cohort member’s age (in months) | -0.10(0.05)\* | -0.11(0.05)\* | -0.12(0.05)\* |
| Sex: Male |  | -0.40(0.04)\*\*\* | -0.41(0.04)\*\*\* |
| England - Disadvantaged |  | -0.10(0.05)\* | -0.09(0.05) |
| England - Ethnic |  | -0.17(0.07)\* | -0.03(0.11) |
| Northern Ireland - Advantaged |  | -0.06(0.08) | -0.06(0.09) |
| Northern Ireland - Disadvantaged |  | -0.06(0.08) | -0.02(0.08) |
| Scotland - Advantaged |  | -0.06(0.08) | -0.07(0.08) |
| Scotland - Disadvantaged |  | -0.17(0.09)\* | -0.16(0.09) |
| Wales - Advantaged |  | -0.02(0.09) | -0.04(0.10) |
| Wales - Disadvantaged |  | 0.05(0.07) | 0.05(0.08) |
| Income |  | 0.08(0.02)\*\*\* | 0.04(0.02) |
| Black or Black British |  |  | 0.10(0.17) |
| Indian |  |  | 0.07(0.12) |
| Mixed |  |  | 0.15(0.12) |
| Other ethnic group |  |  | -0.23(0.26) |
| Pakistani & Bangladeshi |  |  | -0.20(0.16) |
| Maternal education |  |  | 0.03(0.02) |
| Maternal.Age |  |  | 0.00(0.00) |
| Maternal mental health |  |  | -0.04(0.05) |
| Both.Parents.in.Household |  |  | 0.07(0.07) |
| Siblings |  |  | -0.08(0.02)\*\* |
| Small.for.Gestational.Age |  |  | -0.13(0.07) |
| Cognitive\_ability\_age5 |
| Motor.fine | 0.72(0.16)\*\*\* | 0.44(0.16)\*\* | 0.35(0.15)\* |
| Motor.gross | 0.34(0.10)\*\*\* | 0.43(0.11)\*\*\* | 0.43(0.11)\*\*\* |
| Communication | 0.04(0.08) | 0.24(0.08)\*\* | 0.32(0.08)\*\*\* |
| Cohort member’s age (in months) | 0.64(0.26)\* | 0.44(0.29) | 0.33(0.29) |
| Sex: Male |  | -0.64(0.27)\* | -0.51(0.27) |
| England - Disadvantaged |  | -1.49(0.55)\*\* | -0.70(0.55) |
| England - Ethnic |  | -7.37(2.04)\*\*\* | -1.31(1.98) |
| Northern Ireland - Advantaged |  | 0.81(0.86) | 0.24(0.88) |
| Northern Ireland - Disadvantaged |  | 0.13(0.96) | -0.06(0.95) |
| Scotland - Advantaged |  | 1.20(0.72) | 0.59(0.71) |
| Scotland - Disadvantaged |  | -1.25(0.73) | -1.82(0.76)\* |
| Wales - Advantaged |  | -1.09(0.60) | -1.77(0.60)\*\* |
| Wales - Disadvantaged |  | -2.51(0.76)\*\* | -2.35(0.79)\*\* |
| Income |  | 2.15(0.14)\*\*\* | 0.86(0.15)\*\*\* |
| Black or Black British |  |  | -7.32(2.14)\*\*\* |
| Indian |  |  | -4.05(1.39)\*\* |
| Mixed |  |  | -1.43(0.93) |
| Other ethnic group |  |  | -9.52(2.70)\*\*\* |
| Pakistani & Bangladeshi |  |  | -8.64(2.63)\*\* |
| Maternal education |  |  | 1.35(0.13)\*\*\* |
| Maternal.Age |  |  | 0.18(0.03)\*\*\* |
| Maternal mental health |  |  | -0.29(0.31) |
| Both.Parents.in.Household |  |  | -0.36(0.44) |
| Siblings |  |  | -1.49(0.17)\*\*\* |
| Small.for.Gestational.Age |  |  | -1.34(0.41)\*\*\* |
| Self\_regulation\_age5 |
| Motor.fine | 0.33(0.05)\*\*\* | 0.26(0.04)\*\*\* | 0.21(0.04)\*\*\* |
| Motor.gross | 0.04(0.03) | 0.06(0.03)\* | 0.06(0.03)\* |
| Communication | 0.16(0.03)\*\*\* | 0.15(0.02)\*\*\* | 0.16(0.02)\*\*\* |
| Cohort member’s age (in months) | -0.04(0.08) | -0.08(0.08) | -0.05(0.08) |
| Sex: Male |  | -0.65(0.07)\*\*\* | -0.60(0.06)\*\*\* |
| England - Disadvantaged |  | -0.20(0.10) | -0.09(0.10) |
| England - Ethnic |  | -0.52(0.19)\*\* | -0.08(0.23) |
| Northern Ireland - Advantaged |  | 0.82(0.18)\*\*\* | 0.61(0.18)\*\*\* |
| Northern Ireland - Disadvantaged |  | 0.03(0.18) | 0.03(0.17) |
| Scotland - Advantaged |  | 0.39(0.18)\* | 0.29(0.16) |
| Scotland - Disadvantaged |  | 0.00(0.20) | 0.05(0.19) |
| Wales - Advantaged |  | 0.19(0.16) | 0.10(0.14) |
| Wales - Disadvantaged |  | -0.11(0.14) | 0.00(0.14) |
| Income |  | 0.34(0.03)\*\*\* | 0.13(0.04)\*\* |
| Black or Black British |  |  | 0.02(0.28) |
| Indian |  |  | -0.37(0.23) |
| Mixed |  |  | -0.20(0.22) |
| Other ethnic group |  |  | -0.85(0.38)\* |
| Pakistani & Bangladeshi |  |  | -0.64(0.32)\* |
| Maternal education |  |  | 0.17(0.03)\*\*\* |
| Maternal.Age |  |  | 0.06(0.01)\*\*\* |
| Maternal mental health |  |  | -0.42(0.10)\*\*\* |
| Both.Parents.in.Household |  |  | 0.22(0.13) |
| Siblings |  |  | 0.05(0.04) |
| Small.for.Gestational.Age |  |  | -0.32(0.12)\*\* |
| OPEN |
| Motor.fine | 0.00(0.06) | 0.00(0.06) | -0.01(0.06) |
| Motor.gross | 0.08(0.04)\* | 0.09(0.04)\* | 0.09(0.04)\* |
| Communication | 0.01(0.03) | -0.00(0.03) | 0.01(0.03) |
| Theory of mind (age 5) | -0.21(0.13) | -0.23(0.13) | -0.21(0.13) |
| Prosociality (age 5) | -0.06(0.07) | -0.07(0.07) | -0.06(0.06) |
| Cognitive.ability.age5 | 0.07(0.02)\*\*\* | 0.07(0.02)\*\*\* | 0.06(0.02)\*\*\* |
| Self.regulation.age5 | 0.14(0.10) | 0.17(0.10) | 0.16(0.10) |
| Sex: Male |  | -0.14(0.11) | -0.14(0.11) |
| Cohort member’s age (in months) |  | -0.05(0.10) | -0.07(0.10) |
| England - Disadvantaged |  | 0.32(0.13)\* | 0.21(0.14) |
| England - Ethnic |  | 1.24(0.29)\*\*\* | 0.38(0.27) |
| Northern Ireland - Advantaged |  | 0.42(0.23) | 0.52(0.22)\* |
| Northern Ireland - Disadvantaged |  | 0.31(0.19) | 0.49(0.20)\* |
| Scotland - Advantaged |  | 0.18(0.18) | 0.16(0.18) |
| Scotland - Disadvantaged |  | 0.17(0.24) | 0.21(0.24) |
| Wales - Advantaged |  | -0.07(0.21) | -0.04(0.21) |
| Wales - Disadvantaged |  | 0.30(0.19) | 0.36(0.18)\* |
| Income |  | 0.03(0.04) | -0.01(0.05) |
| Black or Black British |  |  | 1.19(0.36)\*\*\* |
| Indian |  |  | 0.52(0.32) |
| Mixed |  |  | 0.83(0.31)\*\* |
| Other ethnic group |  |  | 1.06(0.51)\* |
| Pakistani & Bangladeshi |  |  | 1.57(0.37)\*\*\* |
| Maternal education |  |  | 0.11(0.05)\* |
| Maternal.Age |  |  | 0.03(0.01)\*\* |
| Maternal mental health |  |  | 0.32(0.12)\*\* |
| Both.Parents.in.Household |  |  | -0.41(0.18)\* |
| Siblings |  |  | -0.12(0.07) |
| Small.for.Gestational.Age |  |  | -0.01(0.15) |
| CONSC |
| Motor.fine | 0.00(0.05) | 0.00(0.05) | 0.01(0.05) |
| Motor.gross | 0.03(0.03) | 0.02(0.03) | 0.03(0.04) |
| Communication | 0.04(0.03) | 0.05(0.03) | 0.05(0.03) |
| Theory of mind (age 5) | 0.31(0.11)\*\* | 0.33(0.11)\*\* | 0.35(0.11)\*\* |
| Prosociality (age 5) | -0.05(0.06) | -0.06(0.06) | -0.06(0.06) |
| Cognitive.ability.age5 | 0.01(0.02) | 0.00(0.01) | 0.00(0.01) |
| Self.regulation.age5 | 0.30(0.08)\*\*\* | 0.33(0.08)\*\*\* | 0.37(0.08)\*\*\* |
| Sex: Male |  | 0.12(0.09) | 0.11(0.09) |
| Cohort member’s age (in months) |  | -0.04(0.08) | -0.05(0.08) |
| England - Disadvantaged |  | -0.06(0.13) | -0.13(0.14) |
| England - Ethnic |  | -0.01(0.16) | -0.41(0.20)\* |
| Northern Ireland - Advantaged |  | 0.14(0.20) | 0.20(0.21) |
| Northern Ireland - Disadvantaged |  | -0.14(0.21) | -0.06(0.21) |
| Scotland - Advantaged |  | -0.30(0.18) | -0.30(0.18) |
| Scotland - Disadvantaged |  | -0.11(0.19) | -0.17(0.19) |
| Wales - Advantaged |  | 0.06(0.22) | 0.08(0.22) |
| Wales - Disadvantaged |  | -0.24(0.14) | -0.27(0.14) |
| Income |  | 0.00(0.04) | 0.01(0.05) |
| Black or Black British |  |  | 0.44(0.34) |
| Indian |  |  | 0.37(0.32) |
| Mixed |  |  | -0.61(0.26)\* |
| Other ethnic group |  |  | 0.33(0.34) |
| Pakistani & Bangladeshi |  |  | 0.96(0.29)\*\*\* |
| Maternal education |  |  | -0.01(0.04) |
| Maternal.Age |  |  | -0.02(0.01)\* |
| Maternal mental health |  |  | -0.01(0.11) |
| Both.Parents.in.Household |  |  | 0.05(0.15) |
| Siblings |  |  | -0.17(0.05)\*\*\* |
| Small.for.Gestational.Age |  |  | -0.03(0.13) |
| EXTRAV |
| Motor.fine | 0.03(0.06) | 0.01(0.06) | 0.00(0.06) |
| Motor.gross | 0.04(0.04) | 0.06(0.04) | 0.07(0.04) |
| Communication | 0.06(0.03) | 0.08(0.03)\* | 0.07(0.03)\* |
| Theory of mind (age 5) | 0.38(0.14)\*\* | 0.36(0.14)\* | 0.33(0.14)\* |
| Prosociality (age 5) | 0.02(0.07) | 0.06(0.07) | 0.07(0.06) |
| Cognitive.ability.age5 | -0.02(0.02) | -0.02(0.02) | -0.02(0.01) |
| Self.regulation.age5 | 0.13(0.10) | 0.06(0.10) | 0.04(0.11) |
| Sex: Male |  | -0.20(0.11) | -0.21(0.11) |
| Cohort member’s age (in months) |  | 0.01(0.10) | 0.01(0.10) |
| England - Disadvantaged |  | -0.19(0.13) | -0.23(0.13) |
| England - Ethnic |  | -0.18(0.19) | -0.52(0.23)\* |
| Northern Ireland - Advantaged |  | 0.53(0.28) | 0.55(0.29) |
| Northern Ireland - Disadvantaged |  | 0.33(0.21) | 0.33(0.22) |
| Scotland - Advantaged |  | 0.15(0.22) | 0.15(0.22) |
| Scotland - Disadvantaged |  | 0.03(0.23) | 0.00(0.23) |
| Wales - Advantaged |  | -0.13(0.17) | -0.14(0.17) |
| Wales - Disadvantaged |  | -0.17(0.16) | -0.20(0.16) |
| Income |  | 0.20(0.04)\*\*\* | 0.22(0.06)\*\*\* |
| Black or Black British |  |  | -0.51(0.42) |
| Indian |  |  | 0.65(0.37) |
| Mixed |  |  | -0.11(0.37) |
| Other ethnic group |  |  | -0.31(0.46) |
| Pakistani & Bangladeshi |  |  | 0.76(0.26)\*\* |
| Maternal education |  |  | -0.02(0.04) |
| Maternal.Age |  |  | -0.01(0.01) |
| Maternal mental health |  |  | -0.50(0.14)\*\*\* |
| Both.Parents.in.Household |  |  | -0.11(0.18) |
| Siblings |  |  | -0.02(0.06) |
| Small.for.Gestational.Age |  |  | -0.21(0.16) |
| AGREE |
| Motor.fine | 0.04(0.05) | 0.01(0.05) | 0.00(0.05) |
| Motor.gross | -0.04(0.03) | -0.01(0.03) | 0.01(0.03) |
| Communication | 0.03(0.03) | -0.00(0.03) | -0.00(0.03) |
| Theory of mind (age 5) | 0.21(0.11) | 0.14(0.11) | 0.15(0.10) |
| Prosociality (age 5) | -0.03(0.06) | -0.02(0.06) | -0.01(0.06) |
| Cognitive.ability.age5 | -0.05(0.02)\*\* | -0.04(0.01)\*\*\* | -0.04(0.01)\*\*\* |
| Self.regulation.age5 | 0.33(0.08)\*\*\* | 0.29(0.08)\*\*\* | 0.30(0.08)\*\*\* |
| Sex: Male |  | -0.86(0.09)\*\*\* | -0.88(0.09)\*\*\* |
| Cohort member’s age (in months) |  | 0.12(0.08) | 0.11(0.08) |
| England - Disadvantaged |  | -0.01(0.11) | -0.03(0.11) |
| England - Ethnic |  | -0.12(0.15) | -0.12(0.21) |
| Northern Ireland - Advantaged |  | 0.17(0.15) | 0.18(0.15) |
| Northern Ireland - Disadvantaged |  | 0.36(0.17)\* | 0.35(0.18) |
| Scotland - Advantaged |  | 0.35(0.13)\* | 0.32(0.14)\* |
| Scotland - Disadvantaged |  | 0.40(0.17)\* | 0.34(0.17)\* |
| Wales - Advantaged |  | 0.24(0.15) | 0.21(0.15) |
| Wales - Disadvantaged |  | 0.08(0.16) | 0.08(0.17) |
| Income |  | 0.10(0.04)\*\* | 0.04(0.04) |
| Black or Black British |  |  | -0.62(0.33) |
| Indian |  |  | 0.27(0.28) |
| Mixed |  |  | -0.37(0.25) |
| Other ethnic group |  |  | -0.22(0.38) |
| Pakistani & Bangladeshi |  |  | 0.39(0.31) |
| Maternal education |  |  | 0.05(0.04) |
| Maternal.Age |  |  | 0.01(0.01) |
| Maternal mental health |  |  | 0.01(0.10) |
| Both.Parents.in.Household |  |  | -0.06(0.13) |
| Siblings |  |  | -0.17(0.05)\*\*\* |
| Small.for.Gestational.Age |  |  | 0.04(0.12) |
| NEUROT |
| Motor.fine | 0.14(0.07)\* | 0.08(0.07) | 0.04(0.07) |
| Motor.gross | -0.25(0.05)\*\*\* | -0.12(0.05)\* | -0.10(0.05)\* |
| Communication | 0.11(0.04)\*\* | -0.04(0.03) | -0.03(0.03) |
| Theory of mind (age 5) | 0.15(0.18) | -0.04(0.18) | -0.05(0.17) |
| Prosociality (age 5) | 0.36(0.09)\*\*\* | 0.34(0.08)\*\*\* | 0.32(0.08)\*\*\* |
| Cognitive.ability.age5 | 0.09(0.02)\*\*\* | 0.06(0.02)\*\*\* | 0.04(0.02)\* |
| Self.regulation.age5 | -0.47(0.12)\*\*\* | -0.65(0.12)\*\*\* | -0.65(0.12)\*\*\* |
| Sex: Male |  | -3.68(0.12)\*\*\* | -3.67(0.12)\*\*\* |
| Cohort member’s age (in months) |  | 0.20(0.12) | 0.23(0.13) |
| England - Disadvantaged |  | -0.13(0.18) | 0.02(0.18) |
| England - Ethnic |  | -0.97(0.27)\*\*\* | 0.19(0.38) |
| Northern Ireland - Advantaged |  | 0.07(0.28) | -0.08(0.28) |
| Northern Ireland - Disadvantaged |  | 0.00(0.29) | -0.09(0.30) |
| Scotland - Advantaged |  | 0.82(0.23)\*\*\* | 0.79(0.23)\*\*\* |
| Scotland - Disadvantaged |  | 0.58(0.23)\* | 0.52(0.24)\* |
| Wales - Advantaged |  | 0.45(0.21)\* | 0.35(0.21) |
| Wales - Disadvantaged |  | 0.26(0.20) | 0.30(0.20) |
| Income |  | 0.07(0.06) | 0.01(0.07) |
| Black or Black British |  |  | -1.82(0.54)\*\*\* |
| Indian |  |  | -0.95(0.49) |
| Mixed |  |  | -0.63(0.40) |
| Other ethnic group |  |  | -1.38(0.76) |
| Pakistani & Bangladeshi |  |  | -2.02(0.47)\*\*\* |
| Maternal education |  |  | 0.06(0.06) |
| Maternal.Age |  |  | 0.04(0.02)\* |
| Maternal mental health |  |  | 0.33(0.15)\* |
| Both.Parents.in.Household |  |  | 0.14(0.20) |
| Siblings |  |  | 0.06(0.08) |
| Small.for.Gestational.Age |  |  | -0.01(0.21) |
|  | Intercepts | | |
| Verbal ability (age 5) | 42.43(2.78)\*\*\* | 38.84(3.02)\*\*\* | 35.82(3.18)\*\*\* |
| Spatial ability (age 5) | 40.50(2.36)\*\*\* | 41.81(1.83)\*\*\* | 41.38(1.72)\*\*\* |
| Independence skills (age 5) | 13.03(0.87)\*\*\* | 13.16(0.87)\*\*\* | 11.53(0.85)\*\*\* |
| Emotion regulation (age 5) | 10.22(1.02)\*\*\* | 9.96(1.12)\*\*\* | 7.13(1.23)\*\*\* |
| Theory of mind (age 5) | 1.21(0.11)\*\*\* | 1.20(0.11)\*\*\* | 1.16(0.11)\*\*\* |
| Prosociality (age 5) | 8.10(0.57)\*\*\* | 8.39(0.57)\*\*\* | 8.41(0.59)\*\*\* |
| OPEN | 12.99(0.83)\*\*\* | 13.23(1.25)\*\*\* | 11.93(1.30)\*\*\* |
| CONSC | 12.84(0.71)\*\*\* | 13.11(1.05)\*\*\* | 13.18(1.09)\*\*\* |
| EXTRAV | 11.80(0.86)\*\*\* | 11.23(1.24)\*\*\* | 11.92(1.25)\*\*\* |
| AGREE | 15.84(0.75)\*\*\* | 15.32(1.01)\*\*\* | 14.77(1.07)\*\*\* |
| NEUROT | 8.38(0.96)\*\*\* | 10.12(1.41)\*\*\* | 9.96(1.44)\*\*\* |
| Motor.fine | 7.59(0.01)\*\*\* | 7.59(0.01)\*\*\* | 7.59(0.01)\*\*\* |
| Motor.gross | 5.50(0.02)\*\*\* | 5.50(0.02)\*\*\* | 5.50(0.02)\*\*\* |
| Communication | 6.50(0.03)\*\*\* | 6.50(0.03)\*\*\* | 6.49(0.03)\*\*\* |
| Cohort member’s age (in months) | 9.17+ | 9.17+ | 9.17+ |
| Sex: Male |  | 0.48+ | 0.48+ |
| England - Disadvantaged |  | 0.28+ | 0.28+ |
| England - Ethnic |  | 0.05+ | 0.05+ |
| Northern Ireland - Advantaged |  | 0.03+ | 0.03+ |
| Northern Ireland - Disadvantaged |  | 0.01+ | 0.01+ |
| Scotland - Advantaged |  | 0.06+ | 0.06+ |
| Scotland - Disadvantaged |  | 0.04+ | 0.04+ |
| Wales - Advantaged |  | 0.03+ | 0.04+ |
| Wales - Disadvantaged |  | 0.01+ | 0.01+ |
| Income |  | 3.23+ | 3.26+ |
| Black or Black British |  |  | 0.02+ |
| Indian |  |  | 0.02+ |
| Mixed |  |  | 0.03+ |
| Other ethnic group |  |  | 0.01+ |
| Pakistani & Bangladeshi |  |  | 0.04+ |
| Maternal education |  |  | 3.76+ |
| Maternal.Age |  |  | 30.33+ |
| Maternal mental health |  |  | 0.23+ |
| Both.Parents.in.Household |  |  | 0.89+ |
| Siblings |  |  | 0.86+ |
| Small.for.Gestational.Age |  |  | 0.12+ |
|  | Fit Indices | | |
| χ2 | 571.76 | 1345.77 | 1797.69 |
| CFI | 0.94 | 0.91 | 0.89 |
| TLI | 0.81 | 0.70 | 0.66 |
| RMSEA | 0.05 | 0.04 | 0.04 |
| Scaled χ2 | 449.66(31)\*\*\* | 1116.42(76)\*\*\* | 1433.10(131)\*\*\* |
| +Fixed parameter | | | |
| \*p<0.05, \*\*p<0.01, \*\*\*p<0.001 | | | |

  

|  |  |  |  |
| --- | --- | --- | --- |
|  | Model 1 | Model 2 | Model 3 |
|  | Estimate (Std. Err.) | Estimate (Std. Err.) | Estimate (Std. Err.) |
|  | Regression Slopes | | |
| Theory of mind (age 5) |
| Motor.fine | -0.01(0.01) | -0.01(0.01) | -0.01(0.01)\* |
| Motor.gross | -0.00(0.00) | 0.00(0.00) | 0.00(0.00) |
| Communication | 0.01(0.00)\* | 0.01(0.00)\* | 0.01(0.00)\* |
| Cohort member’s age (in months) | -0.00(0.01) | -0.00(0.01) | -0.01(0.01) |
| Sex: Male |  | -0.04(0.01)\*\*\* | -0.04(0.01)\*\*\* |
| England - Disadvantaged |  | 0.00(0.01) | 0.01(0.01) |
| England - Ethnic |  | -0.02(0.01) | 0.01(0.02) |
| Northern Ireland - Advantaged |  | 0.07(0.03)\* | 0.06(0.03)\* |
| Northern Ireland - Disadvantaged |  | 0.06(0.03)\* | 0.06(0.03)\* |
| Scotland - Advantaged |  | 0.08(0.03)\*\* | 0.07(0.03)\*\* |
| Scotland - Disadvantaged |  | 0.06(0.02)\*\* | 0.06(0.02)\* |
| Wales - Advantaged |  | -0.01(0.02) | -0.01(0.02) |
| Wales - Disadvantaged |  | 0.02(0.02) | 0.03(0.02) |
| Income |  | 0.01(0.00)\*\*\* | 0.00(0.00) |
| Black or Black British |  |  | -0.09(0.02)\*\*\* |
| Indian |  |  | -0.01(0.03) |
| Mixed |  |  | -0.03(0.03) |
| Other ethnic group |  |  | -0.09(0.03)\*\* |
| Pakistani & Bangladeshi |  |  | -0.02(0.02) |
| Maternal education |  |  | 0.01(0.00)\*\*\* |
| Maternal.Age |  |  | 0.00(0.00)\* |
| Maternal mental health |  |  | -0.00(0.01) |
| Both.Parents.in.Household |  |  | -0.02(0.02) |
| Siblings |  |  | -0.01(0.00) |
| Small.for.Gestational.Age |  |  | -0.02(0.01) |
| Prosociality (age 5) |
| Motor.fine | 0.19(0.03)\*\*\* | 0.16(0.03)\*\*\* | 0.16(0.03)\*\*\* |
| Motor.gross | -0.02(0.02) | 0.00(0.02) | 0.00(0.02) |
| Communication | 0.14(0.01)\*\*\* | 0.13(0.01)\*\*\* | 0.13(0.01)\*\*\* |
| Cohort member’s age (in months) | -0.10(0.05)\* | -0.11(0.05)\* | -0.12(0.05)\* |
| Sex: Male |  | -0.40(0.04)\*\*\* | -0.41(0.04)\*\*\* |
| England - Disadvantaged |  | -0.10(0.05)\* | -0.09(0.05) |
| England - Ethnic |  | -0.17(0.07)\* | -0.03(0.11) |
| Northern Ireland - Advantaged |  | -0.06(0.08) | -0.06(0.09) |
| Northern Ireland - Disadvantaged |  | -0.06(0.08) | -0.02(0.08) |
| Scotland - Advantaged |  | -0.06(0.08) | -0.07(0.08) |
| Scotland - Disadvantaged |  | -0.17(0.09)\* | -0.16(0.09) |
| Wales - Advantaged |  | -0.02(0.09) | -0.04(0.10) |
| Wales - Disadvantaged |  | 0.05(0.07) | 0.05(0.08) |
| Income |  | 0.08(0.02)\*\*\* | 0.04(0.02) |
| Black or Black British |  |  | 0.10(0.17) |
| Indian |  |  | 0.07(0.12) |
| Mixed |  |  | 0.15(0.12) |
| Other ethnic group |  |  | -0.23(0.26) |
| Pakistani & Bangladeshi |  |  | -0.20(0.16) |
| Maternal education |  |  | 0.03(0.02) |
| Maternal.Age |  |  | 0.00(0.00) |
| Maternal mental health |  |  | -0.04(0.05) |
| Both.Parents.in.Household |  |  | 0.07(0.07) |
| Siblings |  |  | -0.08(0.02)\*\* |
| Small.for.Gestational.Age |  |  | -0.13(0.07) |
| Cognitive\_ability\_age5 |
| Motor.fine | 0.72(0.16)\*\*\* | 0.44(0.16)\*\* | 0.35(0.15)\* |
| Motor.gross | 0.34(0.10)\*\*\* | 0.43(0.11)\*\*\* | 0.43(0.11)\*\*\* |
| Communication | 0.04(0.08) | 0.24(0.08)\*\* | 0.32(0.08)\*\*\* |
| Cohort member’s age (in months) | 0.64(0.26)\* | 0.44(0.29) | 0.33(0.29) |
| Sex: Male |  | -0.64(0.27)\* | -0.51(0.27) |
| England - Disadvantaged |  | -1.49(0.55)\*\* | -0.70(0.55) |
| England - Ethnic |  | -7.37(2.04)\*\*\* | -1.31(1.98) |
| Northern Ireland - Advantaged |  | 0.81(0.86) | 0.24(0.88) |
| Northern Ireland - Disadvantaged |  | 0.13(0.96) | -0.06(0.95) |
| Scotland - Advantaged |  | 1.20(0.72) | 0.59(0.71) |
| Scotland - Disadvantaged |  | -1.25(0.73) | -1.82(0.76)\* |
| Wales - Advantaged |  | -1.09(0.60) | -1.77(0.60)\*\* |
| Wales - Disadvantaged |  | -2.51(0.76)\*\* | -2.35(0.79)\*\* |
| Income |  | 2.15(0.14)\*\*\* | 0.86(0.15)\*\*\* |
| Black or Black British |  |  | -7.32(2.14)\*\*\* |
| Indian |  |  | -4.05(1.39)\*\* |
| Mixed |  |  | -1.43(0.93) |
| Other ethnic group |  |  | -9.52(2.70)\*\*\* |
| Pakistani & Bangladeshi |  |  | -8.64(2.63)\*\* |
| Maternal education |  |  | 1.35(0.13)\*\*\* |
| Maternal.Age |  |  | 0.18(0.03)\*\*\* |
| Maternal mental health |  |  | -0.29(0.31) |
| Both.Parents.in.Household |  |  | -0.36(0.44) |
| Siblings |  |  | -1.49(0.17)\*\*\* |
| Small.for.Gestational.Age |  |  | -1.34(0.41)\*\*\* |
| Self\_regulation\_age5 |
| Motor.fine | 0.33(0.05)\*\*\* | 0.26(0.04)\*\*\* | 0.21(0.04)\*\*\* |
| Motor.gross | 0.04(0.03) | 0.06(0.03)\* | 0.06(0.03)\* |
| Communication | 0.16(0.03)\*\*\* | 0.15(0.02)\*\*\* | 0.16(0.02)\*\*\* |
| Cohort member’s age (in months) | -0.04(0.08) | -0.08(0.08) | -0.05(0.08) |
| Sex: Male |  | -0.65(0.07)\*\*\* | -0.60(0.06)\*\*\* |
| England - Disadvantaged |  | -0.20(0.10) | -0.09(0.10) |
| England - Ethnic |  | -0.52(0.19)\*\* | -0.08(0.23) |
| Northern Ireland - Advantaged |  | 0.82(0.18)\*\*\* | 0.61(0.18)\*\*\* |
| Northern Ireland - Disadvantaged |  | 0.03(0.18) | 0.03(0.17) |
| Scotland - Advantaged |  | 0.39(0.18)\* | 0.29(0.16) |
| Scotland - Disadvantaged |  | 0.00(0.20) | 0.05(0.19) |
| Wales - Advantaged |  | 0.19(0.16) | 0.10(0.14) |
| Wales - Disadvantaged |  | -0.11(0.14) | 0.00(0.14) |
| Income |  | 0.34(0.03)\*\*\* | 0.13(0.04)\*\* |
| Black or Black British |  |  | 0.02(0.28) |
| Indian |  |  | -0.37(0.23) |
| Mixed |  |  | -0.20(0.22) |
| Other ethnic group |  |  | -0.85(0.38)\* |
| Pakistani & Bangladeshi |  |  | -0.64(0.32)\* |
| Maternal education |  |  | 0.17(0.03)\*\*\* |
| Maternal.Age |  |  | 0.06(0.01)\*\*\* |
| Maternal mental health |  |  | -0.42(0.10)\*\*\* |
| Both.Parents.in.Household |  |  | 0.22(0.13) |
| Siblings |  |  | 0.05(0.04) |
| Small.for.Gestational.Age |  |  | -0.32(0.12)\*\* |
| OPEN |
| Motor.fine | 0.00(0.06) | 0.00(0.06) | -0.01(0.06) |
| Motor.gross | 0.08(0.04)\* | 0.09(0.04)\* | 0.09(0.04)\* |
| Communication | 0.01(0.03) | -0.00(0.03) | 0.01(0.03) |
| Theory of mind (age 5) | -0.21(0.13) | -0.23(0.13) | -0.21(0.13) |
| Prosociality (age 5) | -0.06(0.07) | -0.07(0.07) | -0.06(0.06) |
| Cognitive.ability.age5 | 0.07(0.02)\*\*\* | 0.07(0.02)\*\*\* | 0.06(0.02)\*\*\* |
| Self.regulation.age5 | 0.14(0.10) | 0.17(0.10) | 0.16(0.10) |
| Sex: Male |  | -0.14(0.11) | -0.14(0.11) |
| Cohort member’s age (in months) |  | -0.05(0.10) | -0.07(0.10) |
| England - Disadvantaged |  | 0.32(0.13)\* | 0.21(0.14) |
| England - Ethnic |  | 1.24(0.29)\*\*\* | 0.38(0.27) |
| Northern Ireland - Advantaged |  | 0.42(0.23) | 0.52(0.22)\* |
| Northern Ireland - Disadvantaged |  | 0.31(0.19) | 0.49(0.20)\* |
| Scotland - Advantaged |  | 0.18(0.18) | 0.16(0.18) |
| Scotland - Disadvantaged |  | 0.17(0.24) | 0.21(0.24) |
| Wales - Advantaged |  | -0.07(0.21) | -0.04(0.21) |
| Wales - Disadvantaged |  | 0.30(0.19) | 0.36(0.18)\* |
| Income |  | 0.03(0.04) | -0.01(0.05) |
| Black or Black British |  |  | 1.19(0.36)\*\*\* |
| Indian |  |  | 0.52(0.32) |
| Mixed |  |  | 0.83(0.31)\*\* |
| Other ethnic group |  |  | 1.06(0.51)\* |
| Pakistani & Bangladeshi |  |  | 1.57(0.37)\*\*\* |
| Maternal education |  |  | 0.11(0.05)\* |
| Maternal.Age |  |  | 0.03(0.01)\*\* |
| Maternal mental health |  |  | 0.32(0.12)\*\* |
| Both.Parents.in.Household |  |  | -0.41(0.18)\* |
| Siblings |  |  | -0.12(0.07) |
| Small.for.Gestational.Age |  |  | -0.01(0.15) |
| CONSC |
| Motor.fine | 0.00(0.05) | 0.00(0.05) | 0.01(0.05) |
| Motor.gross | 0.03(0.03) | 0.02(0.03) | 0.03(0.04) |
| Communication | 0.04(0.03) | 0.05(0.03) | 0.05(0.03) |
| Theory of mind (age 5) | 0.31(0.11)\*\* | 0.33(0.11)\*\* | 0.35(0.11)\*\* |
| Prosociality (age 5) | -0.05(0.06) | -0.06(0.06) | -0.06(0.06) |
| Cognitive.ability.age5 | 0.01(0.02) | 0.00(0.01) | 0.00(0.01) |
| Self.regulation.age5 | 0.30(0.08)\*\*\* | 0.33(0.08)\*\*\* | 0.37(0.08)\*\*\* |
| Sex: Male |  | 0.12(0.09) | 0.11(0.09) |
| Cohort member’s age (in months) |  | -0.04(0.08) | -0.05(0.08) |
| England - Disadvantaged |  | -0.06(0.13) | -0.13(0.14) |
| England - Ethnic |  | -0.01(0.16) | -0.41(0.20)\* |
| Northern Ireland - Advantaged |  | 0.14(0.20) | 0.20(0.21) |
| Northern Ireland - Disadvantaged |  | -0.14(0.21) | -0.06(0.21) |
| Scotland - Advantaged |  | -0.30(0.18) | -0.30(0.18) |
| Scotland - Disadvantaged |  | -0.11(0.19) | -0.17(0.19) |
| Wales - Advantaged |  | 0.06(0.22) | 0.08(0.22) |
| Wales - Disadvantaged |  | -0.24(0.14) | -0.27(0.14) |
| Income |  | 0.00(0.04) | 0.01(0.05) |
| Black or Black British |  |  | 0.44(0.34) |
| Indian |  |  | 0.37(0.32) |
| Mixed |  |  | -0.61(0.26)\* |
| Other ethnic group |  |  | 0.33(0.34) |
| Pakistani & Bangladeshi |  |  | 0.96(0.29)\*\*\* |
| Maternal education |  |  | -0.01(0.04) |
| Maternal.Age |  |  | -0.02(0.01)\* |
| Maternal mental health |  |  | -0.01(0.11) |
| Both.Parents.in.Household |  |  | 0.05(0.15) |
| Siblings |  |  | -0.17(0.05)\*\*\* |
| Small.for.Gestational.Age |  |  | -0.03(0.13) |
| EXTRAV |
| Motor.fine | 0.03(0.06) | 0.01(0.06) | 0.00(0.06) |
| Motor.gross | 0.04(0.04) | 0.06(0.04) | 0.07(0.04) |
| Communication | 0.06(0.03) | 0.08(0.03)\* | 0.07(0.03)\* |
| Theory of mind (age 5) | 0.38(0.14)\*\* | 0.36(0.14)\* | 0.33(0.14)\* |
| Prosociality (age 5) | 0.02(0.07) | 0.06(0.07) | 0.07(0.06) |
| Cognitive.ability.age5 | -0.02(0.02) | -0.02(0.02) | -0.02(0.01) |
| Self.regulation.age5 | 0.13(0.10) | 0.06(0.10) | 0.04(0.11) |
| Sex: Male |  | -0.20(0.11) | -0.21(0.11) |
| Cohort member’s age (in months) |  | 0.01(0.10) | 0.01(0.10) |
| England - Disadvantaged |  | -0.19(0.13) | -0.23(0.13) |
| England - Ethnic |  | -0.18(0.19) | -0.52(0.23)\* |
| Northern Ireland - Advantaged |  | 0.53(0.28) | 0.55(0.29) |
| Northern Ireland - Disadvantaged |  | 0.33(0.21) | 0.33(0.22) |
| Scotland - Advantaged |  | 0.15(0.22) | 0.15(0.22) |
| Scotland - Disadvantaged |  | 0.03(0.23) | 0.00(0.23) |
| Wales - Advantaged |  | -0.13(0.17) | -0.14(0.17) |
| Wales - Disadvantaged |  | -0.17(0.16) | -0.20(0.16) |
| Income |  | 0.20(0.04)\*\*\* | 0.22(0.06)\*\*\* |
| Black or Black British |  |  | -0.51(0.42) |
| Indian |  |  | 0.65(0.37) |
| Mixed |  |  | -0.11(0.37) |
| Other ethnic group |  |  | -0.31(0.46) |
| Pakistani & Bangladeshi |  |  | 0.76(0.26)\*\* |
| Maternal education |  |  | -0.02(0.04) |
| Maternal.Age |  |  | -0.01(0.01) |
| Maternal mental health |  |  | -0.50(0.14)\*\*\* |
| Both.Parents.in.Household |  |  | -0.11(0.18) |
| Siblings |  |  | -0.02(0.06) |
| Small.for.Gestational.Age |  |  | -0.21(0.16) |
| AGREE |
| Motor.fine | 0.04(0.05) | 0.01(0.05) | 0.00(0.05) |
| Motor.gross | -0.04(0.03) | -0.01(0.03) | 0.01(0.03) |
| Communication | 0.03(0.03) | -0.00(0.03) | -0.00(0.03) |
| Theory of mind (age 5) | 0.21(0.11) | 0.14(0.11) | 0.15(0.10) |
| Prosociality (age 5) | -0.03(0.06) | -0.02(0.06) | -0.01(0.06) |
| Cognitive.ability.age5 | -0.05(0.02)\*\* | -0.04(0.01)\*\*\* | -0.04(0.01)\*\*\* |
| Self.regulation.age5 | 0.33(0.08)\*\*\* | 0.29(0.08)\*\*\* | 0.30(0.08)\*\*\* |
| Sex: Male |  | -0.86(0.09)\*\*\* | -0.88(0.09)\*\*\* |
| Cohort member’s age (in months) |  | 0.12(0.08) | 0.11(0.08) |
| England - Disadvantaged |  | -0.01(0.11) | -0.03(0.11) |
| England - Ethnic |  | -0.12(0.15) | -0.12(0.21) |
| Northern Ireland - Advantaged |  | 0.17(0.15) | 0.18(0.15) |
| Northern Ireland - Disadvantaged |  | 0.36(0.17)\* | 0.35(0.18) |
| Scotland - Advantaged |  | 0.35(0.13)\* | 0.32(0.14)\* |
| Scotland - Disadvantaged |  | 0.40(0.17)\* | 0.34(0.17)\* |
| Wales - Advantaged |  | 0.24(0.15) | 0.21(0.15) |
| Wales - Disadvantaged |  | 0.08(0.16) | 0.08(0.17) |
| Income |  | 0.10(0.04)\*\* | 0.04(0.04) |
| Black or Black British |  |  | -0.62(0.33) |
| Indian |  |  | 0.27(0.28) |
| Mixed |  |  | -0.37(0.25) |
| Other ethnic group |  |  | -0.22(0.38) |
| Pakistani & Bangladeshi |  |  | 0.39(0.31) |
| Maternal education |  |  | 0.05(0.04) |
| Maternal.Age |  |  | 0.01(0.01) |
| Maternal mental health |  |  | 0.01(0.10) |
| Both.Parents.in.Household |  |  | -0.06(0.13) |
| Siblings |  |  | -0.17(0.05)\*\*\* |
| Small.for.Gestational.Age |  |  | 0.04(0.12) |
| NEUROT |
| Motor.fine | 0.14(0.07)\* | 0.08(0.07) | 0.04(0.07) |
| Motor.gross | -0.25(0.05)\*\*\* | -0.12(0.05)\* | -0.10(0.05)\* |
| Communication | 0.11(0.04)\*\* | -0.04(0.03) | -0.03(0.03) |
| Theory of mind (age 5) | 0.15(0.18) | -0.04(0.18) | -0.05(0.17) |
| Prosociality (age 5) | 0.36(0.09)\*\*\* | 0.34(0.08)\*\*\* | 0.32(0.08)\*\*\* |
| Cognitive.ability.age5 | 0.09(0.02)\*\*\* | 0.06(0.02)\*\*\* | 0.04(0.02)\* |
| Self.regulation.age5 | -0.47(0.12)\*\*\* | -0.65(0.12)\*\*\* | -0.65(0.12)\*\*\* |
| Sex: Male |  | -3.68(0.12)\*\*\* | -3.67(0.12)\*\*\* |
| Cohort member’s age (in months) |  | 0.20(0.12) | 0.23(0.13) |
| England - Disadvantaged |  | -0.13(0.18) | 0.02(0.18) |
| England - Ethnic |  | -0.97(0.27)\*\*\* | 0.19(0.38) |
| Northern Ireland - Advantaged |  | 0.07(0.28) | -0.08(0.28) |
| Northern Ireland - Disadvantaged |  | 0.00(0.29) | -0.09(0.30) |
| Scotland - Advantaged |  | 0.82(0.23)\*\*\* | 0.79(0.23)\*\*\* |
| Scotland - Disadvantaged |  | 0.58(0.23)\* | 0.52(0.24)\* |
| Wales - Advantaged |  | 0.45(0.21)\* | 0.35(0.21) |
| Wales - Disadvantaged |  | 0.26(0.20) | 0.30(0.20) |
| Income |  | 0.07(0.06) | 0.01(0.07) |
| Black or Black British |  |  | -1.82(0.54)\*\*\* |
| Indian |  |  | -0.95(0.49) |
| Mixed |  |  | -0.63(0.40) |
| Other ethnic group |  |  | -1.38(0.76) |
| Pakistani & Bangladeshi |  |  | -2.02(0.47)\*\*\* |
| Maternal education |  |  | 0.06(0.06) |
| Maternal.Age |  |  | 0.04(0.02)\* |
| Maternal mental health |  |  | 0.33(0.15)\* |
| Both.Parents.in.Household |  |  | 0.14(0.20) |
| Siblings |  |  | 0.06(0.08) |
| Small.for.Gestational.Age |  |  | -0.01(0.21) |
|  | Intercepts | | |
| Verbal ability (age 5) | 42.43(2.78)\*\*\* | 38.84(3.02)\*\*\* | 35.82(3.18)\*\*\* |
| Spatial ability (age 5) | 40.50(2.36)\*\*\* | 41.81(1.83)\*\*\* | 41.38(1.72)\*\*\* |
| Independence skills (age 5) | 13.03(0.87)\*\*\* | 13.16(0.87)\*\*\* | 11.53(0.85)\*\*\* |
| Emotion regulation (age 5) | 10.22(1.02)\*\*\* | 9.96(1.12)\*\*\* | 7.13(1.23)\*\*\* |
| Theory of mind (age 5) | 1.21(0.11)\*\*\* | 1.20(0.11)\*\*\* | 1.16(0.11)\*\*\* |
| Prosociality (age 5) | 8.10(0.57)\*\*\* | 8.39(0.57)\*\*\* | 8.41(0.59)\*\*\* |
| OPEN | 12.99(0.83)\*\*\* | 13.23(1.25)\*\*\* | 11.93(1.30)\*\*\* |
| CONSC | 12.84(0.71)\*\*\* | 13.11(1.05)\*\*\* | 13.18(1.09)\*\*\* |
| EXTRAV | 11.80(0.86)\*\*\* | 11.23(1.24)\*\*\* | 11.92(1.25)\*\*\* |
| AGREE | 15.84(0.75)\*\*\* | 15.32(1.01)\*\*\* | 14.77(1.07)\*\*\* |
| NEUROT | 8.38(0.96)\*\*\* | 10.12(1.41)\*\*\* | 9.96(1.44)\*\*\* |
| Motor.fine | 7.59(0.01)\*\*\* | 7.59(0.01)\*\*\* | 7.59(0.01)\*\*\* |
| Motor.gross | 5.50(0.02)\*\*\* | 5.50(0.02)\*\*\* | 5.50(0.02)\*\*\* |
| Communication | 6.50(0.03)\*\*\* | 6.50(0.03)\*\*\* | 6.49(0.03)\*\*\* |
| Cohort member’s age (in months) | 9.17+ | 9.17+ | 9.17+ |
| Sex: Male |  | 0.48+ | 0.48+ |
| England - Disadvantaged |  | 0.28+ | 0.28+ |
| England - Ethnic |  | 0.05+ | 0.05+ |
| Northern Ireland - Advantaged |  | 0.03+ | 0.03+ |
| Northern Ireland - Disadvantaged |  | 0.01+ | 0.01+ |
| Scotland - Advantaged |  | 0.06+ | 0.06+ |
| Scotland - Disadvantaged |  | 0.04+ | 0.04+ |
| Wales - Advantaged |  | 0.03+ | 0.04+ |
| Wales - Disadvantaged |  | 0.01+ | 0.01+ |
| Income |  | 3.23+ | 3.26+ |
| Black or Black British |  |  | 0.02+ |
| Indian |  |  | 0.02+ |
| Mixed |  |  | 0.03+ |
| Other ethnic group |  |  | 0.01+ |
| Pakistani & Bangladeshi |  |  | 0.04+ |
| Maternal education |  |  | 3.76+ |
| Maternal.Age |  |  | 30.33+ |
| Maternal mental health |  |  | 0.23+ |
| Both.Parents.in.Household |  |  | 0.89+ |
| Siblings |  |  | 0.86+ |
| Small.for.Gestational.Age |  |  | 0.12+ |
|  | Fit Indices | | |
| χ2 | 571.76 | 1345.77 | 1797.69 |
| CFI | 0.94 | 0.91 | 0.89 |
| TLI | 0.81 | 0.70 | 0.66 |
| RMSEA | 0.05 | 0.04 | 0.04 |
| Scaled χ2 | 449.66(31)\*\*\* | 1116.42(76)\*\*\* | 1433.10(131)\*\*\* |
| +Fixed parameter | | | |
| \*p<0.05, \*\*p<0.01, \*\*\*p<0.001 | | | |

## Model 3 prime (sensitivity analysis, to compare)

```
## lavaan 0.6-18 ended normally after 1041 iterations
## 
##   Estimator                                         ML
##   Optimization method                           NLMINB
##   Number of model parameters                       314
## 
##   Number of observations                          8076
## 
## Model Test User Model:
##                                               Standard      Scaled
##   Test Statistic                              2269.309    1740.440
##   Degrees of freedom                               141         141
##   P-value (Chi-square)                           0.000       0.000
##   Scaling correction factor                                  1.304
##     Satorra-Bentler correction                                    
## 
## Model Test Baseline Model:
## 
##   Test statistic                             15663.791   11342.793
##   Degrees of freedom                               427         427
##   P-value                                        0.000       0.000
##   Scaling correction factor                                  1.381
## 
## User Model versus Baseline Model:
## 
##   Comparative Fit Index (CFI)                    0.860       0.853
##   Tucker-Lewis Index (TLI)                       0.577       0.556
##                                                                   
##   Robust Comparative Fit Index (CFI)                         0.862
##   Robust Tucker-Lewis Index (TLI)                            0.581
## 
## Loglikelihood and Information Criteria:
## 
##   Loglikelihood user model (H0)            -267187.265 -267187.265
##   Loglikelihood unrestricted model (H1)    -266052.610 -266052.610
##                                                                   
##   Akaike (AIC)                              535002.530  535002.530
##   Bayesian (BIC)                            537199.478  537199.478
##   Sample-size adjusted Bayesian (SABIC)     536201.647  536201.647
## 
## Root Mean Square Error of Approximation:
## 
##   RMSEA                                          0.043       0.037
##   90 Percent confidence interval - lower         0.042       0.036
##   90 Percent confidence interval - upper         0.045       0.039
##   P-value H_0: RMSEA <= 0.050                    1.000       1.000
##   P-value H_0: RMSEA >= 0.080                    0.000       0.000
##                                                                   
##   Robust RMSEA                                               0.043
##   90 Percent confidence interval - lower                     0.041
##   90 Percent confidence interval - upper                     0.045
##   P-value H_0: Robust RMSEA <= 0.050                         1.000
##   P-value H_0: Robust RMSEA >= 0.080                         0.000
## 
## Standardized Root Mean Square Residual:
## 
##   SRMR                                           0.024       0.024
## 
## Parameter Estimates:
## 
##   Standard errors                           Robust.sem
##   Information                                 Expected
##   Information saturated (h1) model          Structured
## 
## Latent Variables:
##                             Estimate  Std.Err  z-value  P(>|z|) ci.lower
##   Cognitive_ability_age5 =~                                             
##     Verbal_blty_g5             1.000                               1.000
##     Spatil_blty_g5             0.499    0.029   17.213    0.000    0.442
##   Self_regulation_age5 =~                                               
##     Independenc_g5             1.000                               1.000
##     Emotion_rgl_g5             1.446    0.054   26.554    0.000    1.339
##  ci.upper   Std.lv  Std.all
##                            
##     1.000    8.065    0.762
##     0.556    4.027    0.420
##                            
##     1.000    1.773    0.488
##     1.553    2.564    0.544
## 
## Regressions:
##                            Estimate  Std.Err  z-value  P(>|z|) ci.lower
##   ToM_age5 ~                                                           
##     Motr_fn   (fT)           -0.012    0.006   -1.957    0.050   -0.024
##     Mtr_grs   (gT)            0.002    0.003    0.701    0.483   -0.004
##     Cmmnctn   (cT)            0.007    0.003    2.444    0.015    0.001
##     CM_Age                   -0.007    0.010   -0.743    0.458   -0.026
##   Prosociality_age5 ~                                                  
##     Motr_fn   (fP)            0.167    0.031    5.382    0.000    0.106
##     Mtr_grs   (gP)            0.012    0.017    0.668    0.504   -0.022
##     Cmmnctn   (cP)            0.133    0.014    9.711    0.000    0.106
##     CM_Age                   -0.123    0.052   -2.371    0.018   -0.224
##   Cognitive_ability_age5 ~                                             
##     Motr_fn   (fC)            0.351    0.156    2.254    0.024    0.046
##     Mtr_grs   (gC)            0.426    0.109    3.924    0.000    0.213
##     Cmmnctn   (cC)            0.315    0.077    4.106    0.000    0.164
##     CM_Age                    0.325    0.293    1.110    0.267   -0.249
##   Self_regulation_age5 ~                                               
##     Motr_fn   (fS)            0.204    0.042    4.859    0.000    0.122
##     Mtr_grs   (gS)            0.057    0.027    2.115    0.034    0.004
##     Cmmnctn   (cS)            0.156    0.022    7.012    0.000    0.113
##     CM_Age                   -0.050    0.076   -0.664    0.507   -0.199
##   OPEN ~                                                               
##     Motr_fn  (F_O)           -0.005    0.062   -0.074    0.941   -0.126
##     Mtr_grs  (G_O)            0.095    0.037    2.577    0.010    0.023
##     Cmmnctn  (C_O)            0.013    0.030    0.448    0.654   -0.045
##     ToM_ag5  (T_O)           -0.212    0.132   -1.600    0.110   -0.472
##     Prscl_5  (P_O)           -0.061    0.065   -0.942    0.346   -0.189
##     Cgnt__5 (Cg_O)            0.062    0.015    4.139    0.000    0.033
##     Slf_r_5  (S_O)            0.162    0.100    1.624    0.104   -0.033
##   CONSC ~                                                              
##     Motr_fn  (F_C)            0.005    0.053    0.086    0.932   -0.100
##     Mtr_grs  (G_C)            0.031    0.035    0.891    0.373   -0.038
##     Cmmnctn  (C_C)            0.044    0.028    1.540    0.124   -0.012
##     ToM_ag5  (T_C)            0.346    0.111    3.113    0.002    0.128
##     Prscl_5  (P_C)           -0.061    0.063   -0.976    0.329   -0.185
##     Cgnt__5 (Cg_C)            0.002    0.010    0.241    0.810   -0.018
##     Slf_r_5  (S_C)            0.370    0.084    4.434    0.000    0.207
##   EXTRAV ~                                                             
##     Motr_fn  (F_E)           -0.005    0.065   -0.083    0.934   -0.133
##     Mtr_grs  (G_E)            0.067    0.041    1.624    0.104   -0.014
##     Cmmnctn  (C_E)            0.067    0.033    2.055    0.040    0.003
##     ToM_ag5  (T_E)            0.327    0.143    2.284    0.022    0.046
##     Prscl_5  (P_E)            0.068    0.065    1.052    0.293   -0.059
##     Cgnt__5 (Cg_E)           -0.017    0.013   -1.283    0.200   -0.044
##     Slf_r_5  (S_E)            0.036    0.105    0.340    0.734   -0.170
##   AGREE ~                                                              
##     Motr_fn  (F_A)            0.011    0.052    0.220    0.826   -0.090
##     Mtr_grs  (G_A)            0.009    0.033    0.276    0.782   -0.056
##     Cmmnctn  (C_A)            0.001    0.027    0.046    0.963   -0.052
##     ToM_ag5  (T_A)            0.148    0.105    1.412    0.158   -0.057
##     Prscl_5  (P_A)           -0.006    0.056   -0.111    0.911   -0.116
##     Cgnt__5 (Cg_A)           -0.044    0.012   -3.588    0.000   -0.068
##     Slf_r_5  (S_A)            0.298    0.081    3.694    0.000    0.140
##   NEUROT ~                                                             
##     Motr_fn  (F_N)            0.054    0.069    0.779    0.436   -0.081
##     Mtr_grs  (G_N)           -0.092    0.049   -1.880    0.060   -0.188
##     Cmmnctn  (C_N)           -0.024    0.035   -0.683    0.495   -0.092
##     ToM_ag5  (T_N)           -0.063    0.173   -0.363    0.717   -0.402
##     Prscl_5  (P_N)            0.313    0.082    3.800    0.000    0.151
##     Cgnt__5 (Cg_N)            0.035    0.015    2.294    0.022    0.005
##     Slf_r_5  (S_N)           -0.636    0.120   -5.302    0.000   -0.871
##   OPEN ~                                                               
##     Sex_Mal                  -0.142    0.108   -1.314    0.189   -0.354
##     CM_Age                   -0.079    0.102   -0.780    0.435   -0.279
##     STRATUM                   0.203    0.135    1.505    0.132   -0.061
##     STRATUM                   0.377    0.271    1.388    0.165   -0.155
##     STRATUM                   0.517    0.224    2.309    0.021    0.078
##     STRATUM                   0.492    0.195    2.521    0.012    0.110
##     STRATUM                   0.161    0.176    0.916    0.360   -0.184
##     STRATUM                   0.205    0.235    0.874    0.382   -0.255
##     STRATUM                  -0.043    0.213   -0.204    0.838   -0.461
##     STRATUM                   0.361    0.181    1.998    0.046    0.007
##     Ethnc_B                   1.176    0.354    3.320    0.001    0.482
##     Ethnc_I                   0.515    0.316    1.631    0.103   -0.104
##     Ethnc_M                   0.832    0.310    2.688    0.007    0.225
##     Ethnc_O                   1.049    0.509    2.061    0.039    0.051
##     Ethn_PB                   1.566    0.371    4.225    0.000    0.840
##     Income                   -0.011    0.046   -0.229    0.819   -0.101
##     Mtrnl_E                   0.110    0.046    2.394    0.017    0.020
##     Mtrnl_A                   0.035    0.010    3.357    0.001    0.015
##     Mtr_M_H                   0.312    0.119    2.634    0.008    0.080
##     Bt_P__H                  -0.418    0.181   -2.311    0.021   -0.773
##     Siblngs                  -0.119    0.067   -1.773    0.076   -0.251
##     Bby_H_P                   0.007    0.028    0.253    0.800   -0.048
##     Prtrm_B                  -0.084    0.236   -0.357    0.721   -0.548
##     Lw_Brth                   0.072    0.260    0.279    0.780   -0.437
##   CONSC ~                                                              
##     Sex_Mal                   0.111    0.088    1.250    0.211   -0.063
##     CM_Age                   -0.049    0.084   -0.587    0.557   -0.213
##     STRATUM                  -0.133    0.137   -0.968    0.333   -0.402
##     STRATUM                  -0.408    0.204   -2.000    0.045   -0.807
##     STRATUM                   0.208    0.206    1.008    0.314   -0.196
##     STRATUM                  -0.054    0.214   -0.252    0.801   -0.474
##     STRATUM                  -0.295    0.180   -1.633    0.102   -0.648
##     STRATUM                  -0.166    0.188   -0.883    0.377   -0.535
##     STRATUM                   0.076    0.225    0.336    0.737   -0.365
##     STRATUM                  -0.263    0.142   -1.852    0.064   -0.541
##     Ethnc_B                   0.439    0.343    1.281    0.200   -0.232
##     Ethnc_I                   0.369    0.320    1.154    0.248   -0.257
##     Ethnc_M                  -0.612    0.262   -2.339    0.019   -1.125
##     Ethnc_O                   0.326    0.348    0.936    0.349   -0.357
##     Ethn_PB                   0.965    0.289    3.340    0.001    0.399
##     Income                    0.004    0.045    0.081    0.935   -0.085
##     Mtrnl_E                  -0.008    0.038   -0.208    0.835   -0.081
##     Mtrnl_A                  -0.020    0.009   -2.086    0.037   -0.038
##     Mtr_M_H                  -0.015    0.109   -0.137    0.891   -0.228
##     Bt_P__H                   0.053    0.147    0.363    0.716   -0.234
##     Siblngs                  -0.172    0.051   -3.347    0.001   -0.272
##     Bby_H_P                   0.015    0.021    0.686    0.493   -0.027
##     Prtrm_B                  -0.173    0.229   -0.759    0.448   -0.622
##     Lw_Brth                   0.039    0.226    0.172    0.863   -0.404
##   EXTRAV ~                                                             
##     Sex_Mal                  -0.222    0.113   -1.963    0.050   -0.443
##     CM_Age                    0.017    0.099    0.169    0.866   -0.177
##     STRATUM                  -0.231    0.133   -1.739    0.082   -0.492
##     STRATUM                  -0.495    0.231   -2.145    0.032   -0.947
##     STRATUM                   0.570    0.286    1.991    0.046    0.009
##     STRATUM                   0.339    0.218    1.557    0.119   -0.088
##     STRATUM                   0.173    0.219    0.790    0.429   -0.256
##     STRATUM                   0.012    0.229    0.054    0.957   -0.436
##     STRATUM                  -0.130    0.168   -0.770    0.441   -0.459
##     STRATUM                  -0.192    0.155   -1.235    0.217   -0.496
##     Ethnc_B                  -0.498    0.414   -1.204    0.229   -1.308
##     Ethnc_I                   0.637    0.381    1.674    0.094   -0.109
##     Ethnc_M                  -0.120    0.371   -0.322    0.747   -0.847
##     Ethnc_O                  -0.299    0.457   -0.655    0.512   -1.195
##     Ethn_PB                   0.754    0.262    2.880    0.004    0.241
##     Income                    0.216    0.056    3.872    0.000    0.107
##     Mtrnl_E                  -0.021    0.045   -0.467    0.640   -0.108
##     Mtrnl_A                  -0.014    0.012   -1.109    0.267   -0.037
##     Mtr_M_H                  -0.505    0.135   -3.740    0.000   -0.770
##     Bt_P__H                  -0.109    0.177   -0.614    0.539   -0.456
##     Siblngs                  -0.018    0.061   -0.295    0.768   -0.137
##     Bby_H_P                   0.038    0.023    1.637    0.102   -0.007
##     Prtrm_B                   0.162    0.261    0.622    0.534   -0.350
##     Lw_Brth                  -0.462    0.234   -1.975    0.048   -0.921
##   AGREE ~                                                              
##     Sex_Mal                  -0.884    0.086  -10.220    0.000   -1.054
##     CM_Age                    0.099    0.080    1.241    0.215   -0.057
##     STRATUM                  -0.037    0.106   -0.350    0.726   -0.244
##     STRATUM                  -0.117    0.209   -0.560    0.576   -0.526
##     STRATUM                   0.183    0.150    1.217    0.223   -0.111
##     STRATUM                   0.354    0.182    1.945    0.052   -0.003
##     STRATUM                   0.324    0.137    2.370    0.018    0.056
##     STRATUM                   0.346    0.172    2.008    0.045    0.008
##     STRATUM                   0.215    0.149    1.440    0.150   -0.078
##     STRATUM                   0.080    0.169    0.472    0.637   -0.251
##     Ethnc_B                  -0.612    0.334   -1.831    0.067   -1.267
##     Ethnc_I                   0.283    0.279    1.015    0.310   -0.264
##     Ethnc_M                  -0.369    0.255   -1.449    0.147   -0.869
##     Ethnc_O                  -0.207    0.380   -0.545    0.586   -0.953
##     Ethn_PB                   0.402    0.311    1.291    0.197   -0.208
##     Income                    0.038    0.039    0.990    0.322   -0.038
##     Mtrnl_E                   0.052    0.038    1.374    0.169   -0.022
##     Mtrnl_A                   0.012    0.010    1.208    0.227   -0.007
##     Mtr_M_H                  -0.001    0.103   -0.014    0.989   -0.204
##     Bt_P__H                  -0.058    0.133   -0.433    0.665   -0.319
##     Siblngs                  -0.173    0.052   -3.343    0.001   -0.274
##     Bby_H_P                   0.016    0.020    0.829    0.407   -0.022
##     Prtrm_B                   0.028    0.208    0.137    0.891   -0.379
##     Lw_Brth                   0.038    0.199    0.191    0.849   -0.353
##   NEUROT ~                                                             
##     Sex_Mal                  -3.677    0.114  -32.217    0.000   -3.901
##     CM_Age                    0.218    0.126    1.733    0.083   -0.028
##     STRATUM                   0.016    0.181    0.090    0.929   -0.338
##     STRATUM                   0.202    0.379    0.531    0.595   -0.542
##     STRATUM                  -0.066    0.279   -0.237    0.813   -0.612
##     STRATUM                  -0.075    0.298   -0.251    0.802   -0.660
##     STRATUM                   0.815    0.227    3.595    0.000    0.371
##     STRATUM                   0.532    0.243    2.191    0.028    0.056
##     STRATUM                   0.358    0.212    1.684    0.092   -0.059
##     STRATUM                   0.301    0.196    1.538    0.124   -0.083
##     Ethnc_B                  -1.832    0.540   -3.394    0.001   -2.890
##     Ethnc_I                  -0.960    0.494   -1.942    0.052   -1.930
##     Ethnc_M                  -0.631    0.402   -1.572    0.116   -1.418
##     Ethnc_O                  -1.373    0.762   -1.803    0.071   -2.866
##     Ethn_PB                  -2.017    0.465   -4.341    0.000   -2.928
##     Income                    0.008    0.069    0.111    0.912   -0.127
##     Mtrnl_E                   0.063    0.057    1.097    0.273   -0.050
##     Mtrnl_A                   0.038    0.015    2.519    0.012    0.009
##     Mtr_M_H                   0.314    0.152    2.065    0.039    0.016
##     Bt_P__H                   0.134    0.202    0.665    0.506   -0.262
##     Siblngs                   0.062    0.076    0.822    0.411   -0.086
##     Bby_H_P                   0.057    0.034    1.649    0.099   -0.011
##     Prtrm_B                   0.103    0.333    0.309    0.758   -0.550
##     Lw_Brth                   0.094    0.334    0.282    0.778   -0.560
##   ToM_age5 ~                                                           
##     Sex_Mal                  -0.039    0.009   -4.234    0.000   -0.057
##     STRATUM                   0.008    0.014    0.546    0.585   -0.020
##     STRATUM                   0.010    0.019    0.539    0.590   -0.027
##     STRATUM                   0.062    0.028    2.224    0.026    0.007
##     STRATUM                   0.063    0.028    2.209    0.027    0.007
##     STRATUM                   0.078    0.028    2.821    0.005    0.024
##     STRATUM                   0.059    0.023    2.560    0.010    0.014
##     STRATUM                  -0.009    0.017   -0.548    0.584   -0.042
##     STRATUM                   0.029    0.018    1.631    0.103   -0.006
##     Ethnc_B                  -0.091    0.023   -3.972    0.000   -0.135
##     Ethnc_I                  -0.016    0.033   -0.492    0.623   -0.082
##     Ethnc_M                  -0.032    0.027   -1.209    0.227   -0.084
##     Ethnc_O                  -0.096    0.034   -2.874    0.004   -0.162
##     Ethn_PB                  -0.021    0.023   -0.909    0.363   -0.066
##     Income                    0.003    0.005    0.683    0.495   -0.006
##     Mtrnl_E                   0.015    0.004    3.911    0.000    0.007
##     Mtrnl_A                   0.002    0.001    2.454    0.014    0.000
##     Mtr_M_H                  -0.007    0.011   -0.609    0.542   -0.028
##     Bt_P__H                  -0.023    0.016   -1.445    0.148   -0.053
##     Siblngs                  -0.004    0.005   -0.833    0.405   -0.014
##     Bby_H_P                   0.008    0.002    3.424    0.001    0.004
##     Prtrm_B                  -0.005    0.025   -0.198    0.843   -0.054
##     Lw_Brth                   0.019    0.024    0.815    0.415   -0.027
##   Prosociality_age5 ~                                                  
##     Sex_Mal                  -0.417    0.039  -10.563    0.000   -0.494
##     STRATUM                  -0.094    0.052   -1.799    0.072   -0.197
##     STRATUM                  -0.033    0.107   -0.305    0.760   -0.242
##     STRATUM                  -0.048    0.086   -0.554    0.579   -0.216
##     STRATUM                  -0.011    0.083   -0.135    0.893   -0.175
##     STRATUM                  -0.069    0.082   -0.836    0.403   -0.230
##     STRATUM                  -0.153    0.090   -1.702    0.089   -0.330
##     STRATUM                  -0.031    0.097   -0.322    0.747   -0.222
##     STRATUM                   0.051    0.079    0.648    0.517   -0.103
##     Ethnc_B                   0.068    0.174    0.391    0.696   -0.274
##     Ethnc_I                   0.033    0.122    0.270    0.787   -0.206
##     Ethnc_M                   0.136    0.124    1.099    0.272   -0.107
##     Ethnc_O                  -0.252    0.265   -0.952    0.341   -0.771
##     Ethn_PB                  -0.220    0.156   -1.410    0.159   -0.525
##     Income                    0.042    0.021    1.953    0.051   -0.000
##     Mtrnl_E                   0.028    0.019    1.493    0.136   -0.009
##     Mtrnl_A                   0.003    0.004    0.607    0.544   -0.006
##     Mtr_M_H                  -0.044    0.053   -0.828    0.408   -0.148
##     Bt_P__H                   0.070    0.074    0.944    0.345   -0.075
##     Siblngs                  -0.065    0.025   -2.613    0.009   -0.115
##     Bby_H_P                   0.009    0.011    0.793    0.428   -0.013
##     Prtrm_B                   0.284    0.102    2.788    0.005    0.084
##     Lw_Brth                   0.053    0.102    0.522    0.602   -0.147
##   Cognitive_ability_age5 ~                                             
##     Sex_Mal                  -0.507    0.266   -1.908    0.056   -1.028
##     STRATUM                  -0.706    0.546   -1.292    0.196   -1.776
##     STRATUM                  -1.340    1.991   -0.673    0.501   -5.243
##     STRATUM                   0.260    0.879    0.296    0.767   -1.462
##     STRATUM                  -0.043    0.955   -0.045    0.964   -1.916
##     STRATUM                   0.581    0.707    0.822    0.411   -0.804
##     STRATUM                  -1.810    0.766   -2.365    0.018   -3.311
##     STRATUM                  -1.741    0.600   -2.901    0.004   -2.917
##     STRATUM                  -2.354    0.790   -2.982    0.003   -3.902
##     Ethnc_B                  -7.444    2.136   -3.485    0.000  -11.631
##     Ethnc_I                  -4.327    1.404   -3.081    0.002   -7.079
##     Ethnc_M                  -1.508    0.930   -1.621    0.105   -3.331
##     Ethnc_O                  -9.730    2.717   -3.581    0.000  -15.055
##     Ethn_PB                  -8.793    2.641   -3.330    0.001  -13.969
##     Income                    0.883    0.151    5.840    0.000    0.587
##     Mtrnl_E                   1.364    0.128   10.676    0.000    1.113
##     Mtrnl_A                   0.177    0.028    6.272    0.000    0.122
##     Mtr_M_H                  -0.278    0.310   -0.897    0.370   -0.887
##     Bt_P__H                  -0.365    0.440   -0.830    0.407   -1.229
##     Siblngs                  -1.484    0.170   -8.727    0.000   -1.817
##     Bby_H_P                  -0.078    0.092   -0.845    0.398   -0.259
##     Prtrm_B                   0.310    0.731    0.425    0.671   -1.122
##     Lw_Brth                  -0.930    0.661   -1.408    0.159   -2.225
##   Self_regulation_age5 ~                                               
##     Sex_Mal                  -0.596    0.063   -9.385    0.000   -0.720
##     STRATUM                  -0.095    0.099   -0.956    0.339   -0.289
##     STRATUM                  -0.087    0.232   -0.374    0.708   -0.541
##     STRATUM                   0.610    0.176    3.472    0.001    0.266
##     STRATUM                   0.032    0.167    0.193    0.847   -0.295
##     STRATUM                   0.272    0.164    1.656    0.098   -0.050
##     STRATUM                   0.046    0.192    0.238    0.812   -0.330
##     STRATUM                   0.106    0.143    0.737    0.461   -0.175
##     STRATUM                  -0.001    0.141   -0.007    0.994   -0.278
##     Ethnc_B                  -0.016    0.274   -0.060    0.953   -0.554
##     Ethnc_I                  -0.432    0.229   -1.886    0.059   -0.881
##     Ethnc_M                  -0.217    0.216   -1.005    0.315   -0.640
##     Ethnc_O                  -0.899    0.385   -2.332    0.020   -1.654
##     Ethn_PB                  -0.662    0.315   -2.104    0.035   -1.279
##     Income                    0.135    0.038    3.512    0.000    0.060
##     Mtrnl_E                   0.173    0.030    5.696    0.000    0.114
##     Mtrnl_A                   0.055    0.007    7.435    0.000    0.040
##     Mtr_M_H                  -0.415    0.096   -4.341    0.000   -0.603
##     Bt_P__H                   0.214    0.133    1.607    0.108   -0.047
##     Siblngs                   0.056    0.041    1.368    0.171   -0.024
##     Bby_H_P                  -0.033    0.017   -1.901    0.057   -0.067
##     Prtrm_B                   0.172    0.186    0.926    0.355   -0.192
##     Lw_Brth                  -0.233    0.192   -1.215    0.224   -0.608
##  ci.upper   Std.lv  Std.all
##                            
##     0.000   -0.012   -0.026
##     0.009    0.002    0.009
##     0.012    0.007    0.031
##     0.012   -0.007   -0.010
##                            
##     0.227    0.167    0.083
##     0.045    0.012    0.009
##     0.160    0.133    0.138
##    -0.021   -0.123   -0.037
##                            
##     0.656    0.044    0.035
##     0.639    0.053    0.069
##     0.465    0.039    0.065
##     0.899    0.040    0.020
##                            
##     0.287    0.115    0.093
##     0.109    0.032    0.042
##     0.200    0.088    0.148
##     0.098   -0.028   -0.014
##                            
##     0.117   -0.005   -0.001
##     0.167    0.095    0.033
##     0.072    0.013    0.006
##     0.048   -0.212   -0.021
##     0.066   -0.061   -0.027
##     0.092    0.504    0.135
##     0.357    0.287    0.077
##                            
##     0.109    0.005    0.001
##     0.101    0.031    0.013
##     0.100    0.044    0.023
##     0.564    0.346    0.039
##     0.062   -0.061   -0.030
##     0.023    0.020    0.006
##     0.534    0.657    0.201
##                            
##     0.122   -0.005   -0.001
##     0.147    0.067    0.022
##     0.131    0.067    0.028
##     0.607    0.327    0.030
##     0.195    0.068    0.028
##     0.009   -0.139   -0.035
##     0.242    0.063    0.016
##                            
##     0.113    0.011    0.003
##     0.074    0.009    0.004
##     0.054    0.001    0.001
##     0.353    0.148    0.018
##     0.103   -0.006   -0.003
##    -0.020   -0.354   -0.117
##     0.456    0.528    0.174
##                            
##     0.188    0.054    0.009
##     0.004   -0.092   -0.025
##     0.044   -0.024   -0.008
##     0.276   -0.063   -0.005
##     0.474    0.313    0.106
##     0.066    0.285    0.060
##    -0.401   -1.127   -0.236
##                            
##     0.070   -0.142   -0.019
##     0.120   -0.079   -0.010
##     0.467    0.203    0.024
##     0.909    0.377    0.022
##     0.956    0.517    0.022
##     0.875    0.492    0.015
##     0.506    0.161    0.010
##     0.665    0.205    0.010
##     0.374   -0.043   -0.002
##     0.715    0.361    0.011
##     1.870    1.176    0.047
##     1.135    0.515    0.019
##     1.439    0.832    0.037
##     2.048    1.049    0.024
##     2.293    1.566    0.078
##     0.080   -0.011   -0.004
##     0.199    0.110    0.040
##     0.055    0.035    0.052
##     0.545    0.312    0.035
##    -0.064   -0.418   -0.035
##     0.013   -0.119   -0.031
##     0.063    0.007    0.003
##     0.379   -0.084   -0.006
##     0.581    0.072    0.005
##                            
##     0.284    0.111    0.017
##     0.115   -0.049   -0.007
##     0.136   -0.133   -0.018
##    -0.008   -0.408   -0.027
##     0.612    0.208    0.010
##     0.366   -0.054   -0.002
##     0.059   -0.295   -0.022
##     0.203   -0.166   -0.010
##     0.516    0.076    0.004
##     0.015   -0.263   -0.009
##     1.110    0.439    0.020
##     0.995    0.369    0.016
##    -0.099   -0.612   -0.031
##     1.009    0.326    0.008
##     1.531    0.965    0.055
##     0.093    0.004    0.002
##     0.066   -0.008   -0.003
##    -0.001   -0.020   -0.033
##     0.198   -0.015   -0.002
##     0.341    0.053    0.005
##    -0.071   -0.172   -0.051
##     0.057    0.015    0.008
##     0.275   -0.173   -0.013
##     0.482    0.039    0.003
##                            
##    -0.000   -0.222   -0.028
##     0.210    0.017    0.002
##     0.029   -0.231   -0.026
##    -0.043   -0.495   -0.027
##     1.132    0.570    0.023
##     0.766    0.339    0.010
##     0.603    0.173    0.010
##     0.460    0.012    0.001
##     0.200   -0.130   -0.006
##     0.113   -0.192   -0.006
##     0.313   -0.498   -0.019
##     1.384    0.637    0.022
##     0.608   -0.120   -0.005
##     0.596   -0.299   -0.006
##     1.267    0.754    0.035
##     0.326    0.216    0.075
##     0.067   -0.021   -0.007
##     0.010   -0.014   -0.019
##    -0.240   -0.505   -0.054
##     0.238   -0.109   -0.009
##     0.101   -0.018   -0.004
##     0.084    0.038    0.017
##     0.674    0.162    0.010
##    -0.003   -0.462   -0.030
##                            
##    -0.714   -0.884   -0.145
##     0.256    0.099    0.016
##     0.170   -0.037   -0.005
##     0.292   -0.117   -0.008
##     0.477    0.183    0.010
##     0.711    0.354    0.014
##     0.592    0.324    0.025
##     0.684    0.346    0.022
##     0.507    0.215    0.013
##     0.410    0.080    0.003
##     0.043   -0.612   -0.030
##     0.830    0.283    0.013
##     0.130   -0.369   -0.020
##     0.538   -0.207   -0.006
##     1.012    0.402    0.025
##     0.114    0.038    0.017
##     0.127    0.052    0.023
##     0.031    0.012    0.022
##     0.201   -0.001   -0.000
##     0.203   -0.058   -0.006
##    -0.072   -0.173   -0.055
##     0.055    0.016    0.010
##     0.436    0.028    0.002
##     0.429    0.038    0.003
##                            
##    -3.453   -3.677   -0.385
##     0.464    0.218    0.022
##     0.370    0.016    0.002
##     0.945    0.202    0.009
##     0.480   -0.066   -0.002
##     0.510   -0.075   -0.002
##     1.260    0.815    0.041
##     1.008    0.532    0.021
##     0.774    0.358    0.014
##     0.685    0.301    0.007
##    -0.774   -1.832   -0.058
##     0.009   -0.960   -0.028
##     0.156   -0.631   -0.022
##     0.120   -1.373   -0.024
##    -1.107   -2.017   -0.078
##     0.143    0.008    0.002
##     0.176    0.063    0.018
##     0.068    0.038    0.045
##     0.612    0.314    0.028
##     0.530    0.134    0.009
##     0.210    0.062    0.013
##     0.124    0.057    0.021
##     0.756    0.103    0.005
##     0.748    0.094    0.005
##                            
##    -0.021   -0.039   -0.053
##     0.036    0.008    0.010
##     0.047    0.010    0.006
##     0.116    0.062    0.027
##     0.118    0.063    0.020
##     0.132    0.078    0.051
##     0.105    0.059    0.031
##     0.024   -0.009   -0.005
##     0.065    0.029    0.009
##    -0.046   -0.091   -0.037
##     0.049   -0.016   -0.006
##     0.020   -0.032   -0.014
##    -0.031   -0.096   -0.022
##     0.024   -0.021   -0.011
##     0.013    0.003    0.013
##     0.022    0.015    0.056
##     0.004    0.002    0.036
##     0.015   -0.007   -0.008
##     0.008   -0.023   -0.019
##     0.005   -0.004   -0.011
##     0.013    0.008    0.042
##     0.044   -0.005   -0.003
##     0.066    0.019    0.014
##                            
##    -0.340   -0.417   -0.129
##     0.008   -0.094   -0.026
##     0.177   -0.033   -0.004
##     0.121   -0.048   -0.005
##     0.152   -0.011   -0.001
##     0.092   -0.069   -0.010
##     0.023   -0.153   -0.018
##     0.159   -0.031   -0.004
##     0.205    0.051    0.004
##     0.410    0.068    0.006
##     0.272    0.033    0.003
##     0.379    0.136    0.014
##     0.267   -0.252   -0.013
##     0.086   -0.220   -0.025
##     0.084    0.042    0.035
##     0.064    0.028    0.023
##     0.012    0.003    0.009
##     0.060   -0.044   -0.011
##     0.215    0.070    0.013
##    -0.016   -0.065   -0.039
##     0.031    0.009    0.010
##     0.484    0.284    0.043
##     0.254    0.053    0.008
##                            
##     0.014   -0.063   -0.031
##     0.365   -0.087   -0.039
##     2.563   -0.166   -0.036
##     1.982    0.032    0.005
##     1.829   -0.005   -0.001
##     1.966    0.072    0.017
##    -0.310   -0.224   -0.043
##    -0.565   -0.216   -0.040
##    -0.807   -0.292   -0.033
##    -3.257   -0.923   -0.139
##    -1.575   -0.536   -0.074
##     0.315   -0.187   -0.031
##    -4.404   -1.206   -0.101
##    -3.617   -1.090   -0.202
##     1.180    0.110    0.150
##     1.614    0.169    0.229
##     0.233    0.022    0.123
##     0.330   -0.035   -0.015
##     0.498   -0.045   -0.014
##    -1.151   -0.184   -0.177
##     0.103   -0.010   -0.017
##     1.743    0.038    0.009
##     0.365   -0.115   -0.029
##                            
##    -0.471   -0.336   -0.168
##     0.099   -0.053   -0.024
##     0.368   -0.049   -0.011
##     0.955    0.344    0.055
##     0.360    0.018    0.002
##     0.594    0.153    0.037
##     0.421    0.026    0.005
##     0.386    0.060    0.011
##     0.276   -0.001   -0.000
##     0.521   -0.009   -0.001
##     0.017   -0.244   -0.034
##     0.206   -0.122   -0.020
##    -0.144   -0.507   -0.042
##    -0.045   -0.373   -0.069
##     0.210    0.076    0.104
##     0.233    0.098    0.133
##     0.069    0.031    0.172
##    -0.228   -0.234   -0.099
##     0.475    0.121    0.037
##     0.136    0.031    0.030
##     0.001   -0.019   -0.034
##     0.536    0.097    0.024
##     0.143   -0.131   -0.033
## 
## Covariances:
##                             Estimate  Std.Err  z-value  P(>|z|) ci.lower
##  .ToM_age5 ~~                                                           
##    .Prosocialty_g5             0.017    0.006    2.584    0.010    0.004
##  .Cognitive_ability_age5 ~~                                             
##    .ToM_age5                   0.295    0.041    7.220    0.000    0.215
##  .Self_regulation_age5 ~~                                               
##    .ToM_age5                   0.032    0.012    2.710    0.007    0.009
##  .Cognitive_ability_age5 ~~                                             
##    .Prosocialty_g5             0.740    0.200    3.689    0.000    0.347
##  .Self_regulation_age5 ~~                                               
##    .Prosocialty_g5             1.537    0.068   22.608    0.000    1.403
##  .Cognitive_ability_age5 ~~                                             
##    .Self_regltn_g5             3.165    0.383    8.260    0.000    2.414
##   Motor_fine ~~                                                         
##     Motor_gross                0.202    0.019   10.636    0.000    0.165
##     Communication              0.357    0.023   15.472    0.000    0.312
##   Motor_gross ~~                                                        
##     Communication              0.704    0.033   21.550    0.000    0.640
##  .OPEN ~~                                                               
##    .CONSC                      1.772    0.172   10.306    0.000    1.435
##    .EXTRAV                     1.911    0.194    9.874    0.000    1.532
##    .AGREE                      2.258    0.165   13.670    0.000    1.934
##    .NEUROT                     0.058    0.221    0.263    0.792   -0.375
##  .CONSC ~~                                                              
##    .EXTRAV                     2.075    0.156   13.262    0.000    1.769
##    .AGREE                      2.817    0.153   18.470    0.000    2.518
##    .NEUROT                    -3.278    0.193  -16.949    0.000   -3.657
##  .EXTRAV ~~                                                             
##    .AGREE                      1.442    0.162    8.928    0.000    1.126
##    .NEUROT                    -5.708    0.219  -26.074    0.000   -6.137
##  .AGREE ~~                                                              
##    .NEUROT                    -0.661    0.171   -3.875    0.000   -0.995
##  ci.upper   Std.lv  Std.all
##                            
##     0.029    0.017    0.029
##                            
##     0.375    0.046    0.128
##                            
##     0.056    0.021    0.058
##                            
##     1.133    0.115    0.074
##                            
##     1.670    0.991    0.632
##                            
##     3.916    0.319    0.319
##                            
##     0.239    0.202    0.192
##     0.403    0.357    0.263
##                            
##     0.768    0.704    0.321
##                            
##     2.109    1.772    0.152
##     2.290    1.911    0.134
##     2.582    2.258    0.211
##     0.491    0.058    0.004
##                            
##     2.382    2.075    0.166
##     3.116    2.817    0.300
##    -2.899   -3.278   -0.236
##                            
##     1.759    1.442    0.125
##    -5.278   -5.708   -0.335
##                            
##    -0.326   -0.661   -0.052
## 
## Intercepts:
##                    Estimate  Std.Err  z-value  P(>|z|) ci.lower ci.upper
##    .Verbal_blty_g5   35.860    3.181   11.275    0.000   29.626   42.093
##    .Spatil_blty_g5   41.523    1.713   24.236    0.000   38.165   44.881
##    .Independenc_g5   11.546    0.851   13.567    0.000    9.878   13.214
##    .Emotion_rgl_g5    7.127    1.242    5.737    0.000    4.692    9.562
##    .ToM_age5          1.139    0.111   10.263    0.000    0.922    1.357
##    .Prosocialty_g5    8.252    0.596   13.850    0.000    7.084    9.420
##    .OPEN             11.921    1.295    9.207    0.000    9.383   14.458
##    .CONSC            13.181    1.088   12.117    0.000   11.049   15.313
##    .EXTRAV           11.914    1.251    9.526    0.000    9.462   14.365
##    .AGREE            14.735    1.062   13.876    0.000   12.654   16.816
##    .NEUROT            9.865    1.434    6.881    0.000    7.055   12.675
##     Motor_fine        7.591    0.013  572.735    0.000    7.565    7.617
##     Motor_gross       5.503    0.017  327.695    0.000    5.470    5.536
##     Communication     6.494    0.026  245.640    0.000    6.442    6.546
##    Std.lv  Std.all
##    35.860    3.388
##    41.523    4.330
##    11.546    3.175
##     7.127    1.513
##     1.139    3.122
##     8.252    5.092
##    11.921    3.200
##    13.181    4.036
##    11.914    3.010
##    14.735    4.849
##     9.865    2.066
##     7.591    9.396
##     5.503    4.221
##     6.494    3.868
## 
## Variances:
##                    Estimate  Std.Err  z-value  P(>|z|) ci.lower ci.upper
##    .ToM_age5          0.131    0.004   37.090    0.000    0.124    0.138
##    .Prosocialty_g5    2.463    0.053   46.157    0.000    2.358    2.567
##    .Cogntv_blty_g5   41.047    3.370   12.179    0.000   34.441   47.653
##    .Self_regltn_g5    2.404    0.162   14.810    0.000    2.086    2.722
##     Motor_fine        0.653    0.026   25.439    0.000    0.602    0.703
##     Motor_gross       1.700    0.040   42.540    0.000    1.622    1.778
##     Communication     2.819    0.050   56.316    0.000    2.721    2.917
##    .Verbal_blty_g5   46.990    3.280   14.328    0.000   40.562   53.418
##    .Spatil_blty_g5   75.724    2.406   31.477    0.000   71.009   80.439
##    .Independenc_g5   10.080    0.233   43.200    0.000    9.623   10.537
##    .Emotion_rgl_g5   15.617    0.385   40.539    0.000   14.862   16.372
##    .OPEN             13.247    0.222   59.784    0.000   12.813   13.681
##    .CONSC            10.200    0.203   50.319    0.000    9.803   10.598
##    .EXTRAV           15.382    0.242   63.504    0.000   14.907   15.857
##    .AGREE             8.670    0.210   41.287    0.000    8.259    9.082
##    .NEUROT           18.896    0.289   65.377    0.000   18.330   19.463
##    Std.lv  Std.all
##     0.131    0.981
##     2.463    0.938
##     0.631    0.631
##     0.765    0.765
##     0.653    1.000
##     1.700    1.000
##     2.819    1.000
##    46.990    0.419
##    75.724    0.824
##    10.080    0.762
##    15.617    0.704
##    13.247    0.955
##    10.200    0.957
##    15.382    0.982
##     8.670    0.939
##    18.896    0.829
## 
## Defined Parameters:
##                    Estimate  Std.Err  z-value  P(>|z|) ci.lower ci.upper
##     fT_T_O            0.003    0.002    1.324    0.186   -0.001    0.006
##     gT_T_O           -0.001    0.001   -0.648    0.517   -0.002    0.001
##     cT_T_O           -0.001    0.001   -1.309    0.191   -0.004    0.001
##     fP_P_O           -0.010    0.011   -0.930    0.352   -0.032    0.011
##     gP_P_O           -0.001    0.001   -0.555    0.579   -0.003    0.002
##     cP_P_O           -0.008    0.009   -0.935    0.350   -0.025    0.009
##     fC_C_O            0.022    0.011    2.008    0.045    0.001    0.043
##     gC_C_O            0.027    0.009    3.038    0.002    0.009    0.044
##     cC_C_O            0.020    0.007    2.862    0.004    0.006    0.033
##     fS_S_O            0.033    0.022    1.530    0.126   -0.009    0.075
##     gS_S_O            0.009    0.007    1.243    0.214   -0.005    0.024
##     cS_S_O            0.025    0.016    1.617    0.106   -0.005    0.056
##     total_fO          0.043    0.061    0.702    0.483   -0.076    0.162
##     total_gO          0.129    0.036    3.560    0.000    0.058    0.200
##     total_cO          0.049    0.029    1.662    0.097   -0.009    0.106
##     fT_T_C           -0.004    0.003   -1.615    0.106   -0.009    0.001
##     gT_T_C            0.001    0.001    0.676    0.499   -0.002    0.003
##     cT_T_C            0.002    0.001    2.006    0.045    0.000    0.005
##     fP_P_C           -0.010    0.011   -0.931    0.352   -0.032    0.011
##     gP_P_C           -0.001    0.001   -0.572    0.568   -0.003    0.002
##     cP_P_C           -0.008    0.008   -0.963    0.336   -0.025    0.008
##     fC_C_C            0.001    0.004    0.241    0.810   -0.006    0.008
##     gC_C_C            0.001    0.004    0.241    0.810   -0.008    0.010
##     cC_C_C            0.001    0.003    0.241    0.809   -0.006    0.007
##     fS_S_C            0.076    0.024    3.157    0.002    0.029    0.123
##     gS_S_C            0.021    0.011    1.895    0.058   -0.001    0.043
##     cS_S_C            0.058    0.015    3.881    0.000    0.029    0.087
##     total_fC          0.067    0.052    1.277    0.202   -0.036    0.169
##     total_gC          0.054    0.034    1.579    0.114   -0.013    0.120
##     total_cC          0.097    0.027    3.526    0.000    0.043    0.150
##     fT_T_E           -0.004    0.003   -1.536    0.124   -0.009    0.001
##     gT_T_E            0.001    0.001    0.638    0.524   -0.002    0.003
##     cT_T_E            0.002    0.001    1.745    0.081   -0.000    0.005
##     fP_P_E            0.011    0.011    1.036    0.300   -0.010    0.033
##     gP_P_E            0.001    0.001    0.567    0.571   -0.002    0.003
##     cP_P_E            0.009    0.009    1.051    0.293   -0.008    0.026
##     fC_C_E           -0.006    0.006   -1.065    0.287   -0.017    0.005
##     gC_C_E           -0.007    0.006   -1.250    0.211   -0.019    0.004
##     cC_C_E           -0.005    0.004   -1.218    0.223   -0.014    0.003
##     fS_S_E            0.007    0.022    0.336    0.737   -0.035    0.050
##     gS_S_E            0.002    0.006    0.335    0.738   -0.010    0.014
##     cS_S_E            0.006    0.016    0.339    0.735   -0.027    0.038
##     total_fE          0.003    0.064    0.052    0.958   -0.122    0.128
##     total_gE          0.063    0.041    1.541    0.123   -0.017    0.143
##     total_cE          0.078    0.032    2.436    0.015    0.015    0.141
##     fT_T_A           -0.002    0.002   -1.096    0.273   -0.005    0.001
##     gT_T_A            0.000    0.001    0.625    0.532   -0.001    0.001
##     cT_T_A            0.001    0.001    1.341    0.180   -0.000    0.002
##     fP_P_A           -0.001    0.009   -0.111    0.911   -0.019    0.017
##     gP_P_A           -0.000    0.001   -0.111    0.911   -0.001    0.001
##     cP_P_A           -0.001    0.007   -0.111    0.912   -0.015    0.014
##     fC_C_A           -0.015    0.008   -1.865    0.062   -0.032    0.001
##     gC_C_A           -0.019    0.007   -2.794    0.005   -0.032   -0.006
##     cC_C_A           -0.014    0.005   -2.581    0.010   -0.024   -0.003
##     fS_S_A            0.061    0.021    2.841    0.004    0.019    0.103
##     gS_S_A            0.017    0.009    1.881    0.060   -0.001    0.034
##     cS_S_A            0.047    0.015    3.207    0.001    0.018    0.075
##     total_fA          0.054    0.049    1.093    0.274   -0.043    0.151
##     total_gA          0.008    0.032    0.240    0.811   -0.054    0.070
##     total_cA          0.034    0.026    1.302    0.193   -0.017    0.086
##     fT_T_N            0.001    0.002    0.349    0.727   -0.003    0.005
##     gT_T_N           -0.000    0.000   -0.342    0.732   -0.001    0.001
##     cT_T_N           -0.000    0.001   -0.352    0.725   -0.003    0.002
##     fP_P_N            0.052    0.017    3.050    0.002    0.019    0.086
##     gP_P_N            0.004    0.005    0.665    0.506   -0.007    0.014
##     cP_P_N            0.042    0.012    3.485    0.000    0.018    0.065
##     fC_C_N            0.012    0.007    1.663    0.096   -0.002    0.027
##     gC_C_N            0.015    0.008    1.908    0.056   -0.000    0.031
##     cC_C_N            0.011    0.005    2.042    0.041    0.000    0.022
##     fS_S_N           -0.130    0.038   -3.414    0.001   -0.204   -0.055
##     gS_S_N           -0.036    0.019   -1.927    0.054   -0.073    0.001
##     cS_S_N           -0.099    0.023   -4.264    0.000   -0.145   -0.054
##     total_fN         -0.011    0.070   -0.159    0.873   -0.147    0.125
##     total_gN         -0.110    0.045   -2.460    0.014   -0.197   -0.022
##     total_cN         -0.071    0.035   -2.011    0.044   -0.140   -0.002
##    Std.lv  Std.all
##     0.003    0.001
##    -0.001   -0.000
##    -0.001   -0.001
##    -0.010   -0.002
##    -0.001   -0.000
##    -0.008   -0.004
##     0.022    0.005
##     0.027    0.009
##     0.020    0.009
##     0.033    0.007
##     0.009    0.003
##     0.025    0.011
##     0.043    0.009
##     0.129    0.045
##     0.049    0.022
##    -0.004   -0.001
##     0.001    0.000
##     0.002    0.001
##    -0.010   -0.003
##    -0.001   -0.000
##    -0.008   -0.004
##     0.001    0.000
##     0.001    0.000
##     0.001    0.000
##     0.076    0.019
##     0.021    0.008
##     0.058    0.030
##     0.067    0.017
##     0.054    0.021
##     0.097    0.050
##    -0.004   -0.001
##     0.001    0.000
##     0.002    0.001
##     0.011    0.002
##     0.001    0.000
##     0.009    0.004
##    -0.006   -0.001
##    -0.007   -0.002
##    -0.005   -0.002
##     0.007    0.001
##     0.002    0.001
##     0.006    0.002
##     0.003    0.001
##     0.063    0.021
##     0.078    0.033
##    -0.002   -0.000
##     0.000    0.000
##     0.001    0.001
##    -0.001   -0.000
##    -0.000   -0.000
##    -0.001   -0.000
##    -0.015   -0.004
##    -0.019   -0.008
##    -0.014   -0.008
##     0.061    0.016
##     0.017    0.007
##     0.047    0.026
##     0.054    0.014
##     0.008    0.003
##     0.034    0.019
##     0.001    0.000
##    -0.000   -0.000
##    -0.000   -0.000
##     0.052    0.009
##     0.004    0.001
##     0.042    0.015
##     0.012    0.002
##     0.015    0.004
##     0.011    0.004
##    -0.130   -0.022
##    -0.036   -0.010
##    -0.099   -0.035
##    -0.011   -0.002
##    -0.110   -0.030
##    -0.071   -0.025
```

## Models 0: unimputed, for comparison

## Model 0a: Motor Function –> Big5

```
## lavaan 0.6-18 ended normally after 167 iterations
## 
##   Estimator                                         ML
##   Optimization method                           NLMINB
##   Number of model parameters                        44
## 
##   Number of observations                          9191
## 
## Model Test User Model:
##                                               Standard      Scaled
##   Test Statistic                                 0.000       0.000
##   Degrees of freedom                                 0           0
## 
## Model Test Baseline Model:
## 
##   Test statistic                              5199.948    3487.088
##   Degrees of freedom                                28          28
##   P-value                                        0.000       0.000
##   Scaling correction factor                                  1.491
## 
## User Model versus Baseline Model:
## 
##   Comparative Fit Index (CFI)                    1.000       1.000
##   Tucker-Lewis Index (TLI)                       1.000       1.000
##                                                                   
##   Robust Comparative Fit Index (CFI)                            NA
##   Robust Tucker-Lewis Index (TLI)                               NA
## 
## Loglikelihood and Information Criteria:
## 
##   Loglikelihood user model (H0)            -167542.170 -167542.170
##   Loglikelihood unrestricted model (H1)    -167542.170 -167542.170
##                                                                   
##   Akaike (AIC)                              335172.341  335172.341
##   Bayesian (BIC)                            335485.884  335485.884
##   Sample-size adjusted Bayesian (SABIC)     335346.059  335346.059
## 
## Root Mean Square Error of Approximation:
## 
##   RMSEA                                          0.000          NA
##   90 Percent confidence interval - lower         0.000          NA
##   90 Percent confidence interval - upper         0.000          NA
##   P-value H_0: RMSEA <= 0.050                       NA          NA
##   P-value H_0: RMSEA >= 0.080                       NA          NA
##                                                                   
##   Robust RMSEA                                               0.000
##   90 Percent confidence interval - lower                     0.000
##   90 Percent confidence interval - upper                     0.000
##   P-value H_0: Robust RMSEA <= 0.050                            NA
##   P-value H_0: Robust RMSEA >= 0.080                            NA
## 
## Standardized Root Mean Square Residual:
## 
##   SRMR                                           0.000       0.000
## 
## Parameter Estimates:
## 
##   Standard errors                           Robust.sem
##   Information                                 Expected
##   Information saturated (h1) model          Structured
## 
## Regressions:
##                    Estimate  Std.Err  z-value  P(>|z|) ci.lower ci.upper
##   OPEN ~                                                                
##     Motor_fine        0.092    0.058    1.593    0.111   -0.021    0.205
##     Motor_gross       0.120    0.036    3.371    0.001    0.050    0.190
##     Communication     0.038    0.028    1.380    0.168   -0.016    0.093
##   CONSC ~                                                               
##     Motor_fine        0.116    0.049    2.367    0.018    0.020    0.213
##     Motor_gross       0.043    0.032    1.329    0.184   -0.020    0.106
##     Communication     0.073    0.025    2.940    0.003    0.024    0.121
##   EXTRAV ~                                                              
##     Motor_fine        0.066    0.057    1.173    0.241   -0.044    0.177
##     Motor_gross       0.029    0.037    0.789    0.430   -0.043    0.101
##     Communication     0.085    0.029    2.890    0.004    0.027    0.143
##   AGREE ~                                                               
##     Motor_fine        0.120    0.046    2.614    0.009    0.030    0.210
##     Motor_gross      -0.049    0.029   -1.696    0.090   -0.107    0.008
##     Communication     0.082    0.025    3.260    0.001    0.033    0.131
##   NEUROT ~                                                              
##     Motor_fine        0.087    0.066    1.322    0.186   -0.042    0.216
##     Motor_gross      -0.203    0.042   -4.836    0.000   -0.286   -0.121
##     Communication     0.073    0.036    2.039    0.041    0.003    0.144
##    Std.lv  Std.all
##                   
##     0.092    0.020
##     0.120    0.042
##     0.038    0.017
##                   
##     0.116    0.029
##     0.043    0.017
##     0.073    0.038
##                   
##     0.066    0.014
##     0.029    0.010
##     0.085    0.037
##                   
##     0.120    0.033
##    -0.049   -0.021
##     0.082    0.045
##                   
##     0.087    0.015
##    -0.203   -0.057
##     0.073    0.026
## 
## Covariances:
##                    Estimate  Std.Err  z-value  P(>|z|) ci.lower ci.upper
##   Motor_fine ~~                                                         
##     Motor_gross       0.213    0.019   11.483    0.000    0.177    0.250
##     Communication     0.369    0.022   16.628    0.000    0.326    0.413
##   Motor_gross ~~                                                        
##     Communication     0.703    0.031   22.310    0.000    0.641    0.764
##  .OPEN ~~                                                               
##    .CONSC             2.153    0.164   13.148    0.000    1.832    2.474
##    .EXTRAV            2.059    0.188   10.940    0.000    1.690    2.428
##    .AGREE             2.521    0.152   16.607    0.000    2.224    2.819
##    .NEUROT           -0.026    0.208   -0.123    0.902   -0.434    0.383
##  .CONSC ~~                                                              
##    .EXTRAV            2.265    0.155   14.569    0.000    1.961    2.570
##    .AGREE             3.158    0.141   22.338    0.000    2.881    3.435
##    .NEUROT           -3.593    0.192  -18.735    0.000   -3.969   -3.217
##  .EXTRAV ~~                                                             
##    .AGREE             1.669    0.162   10.282    0.000    1.351    1.987
##    .NEUROT           -5.382    0.225  -23.932    0.000   -5.823   -4.941
##  .AGREE ~~                                                              
##    .NEUROT           -0.112    0.157   -0.713    0.476   -0.419    0.196
##    Std.lv  Std.all
##                   
##     0.213    0.196
##     0.369    0.264
##                   
##     0.703    0.315
##                   
##     2.153    0.176
##     2.059    0.140
##     2.521    0.221
##    -0.026   -0.001
##                   
##     2.265    0.177
##     3.158    0.318
##    -3.593   -0.233
##                   
##     1.669    0.139
##    -5.382   -0.289
##                   
##    -0.112   -0.008
## 
## Intercepts:
##                    Estimate  Std.Err  z-value  P(>|z|) ci.lower ci.upper
##    .OPEN             12.596    0.465   27.096    0.000   11.685   13.507
##    .CONSC            12.610    0.369   34.151    0.000   11.887   13.334
##    .EXTRAV           12.326    0.460   26.775    0.000   11.424   13.229
##    .AGREE            15.407    0.367   41.969    0.000   14.687   16.127
##    .NEUROT           11.955    0.508   23.551    0.000   10.960   12.950
##     Motor_fine        7.582    0.013  580.304    0.000    7.557    7.608
##     Motor_gross       5.501    0.017  320.923    0.000    5.468    5.535
##     Communication     6.510    0.026  255.139    0.000    6.460    6.560
##    Std.lv  Std.all
##    12.596    3.358
##    12.610    3.861
##    12.326    3.132
##    15.407    5.051
##    11.955    2.519
##     7.582    9.161
##     5.501    4.173
##     6.510    3.851
## 
## Variances:
##                    Estimate  Std.Err  z-value  P(>|z|) ci.lower ci.upper
##     Motor_fine        0.685    0.027   25.234    0.000    0.632    0.738
##     Motor_gross       1.738    0.039   44.199    0.000    1.661    1.815
##     Communication     2.858    0.049   58.329    0.000    2.762    2.954
##    .OPEN             14.024    0.207   67.716    0.000   13.618   14.430
##    .CONSC            10.626    0.186   57.155    0.000   10.262   10.990
##    .EXTRAV           15.453    0.234   66.074    0.000   14.994   15.911
##    .AGREE             9.272    0.199   46.578    0.000    8.881    9.662
##    .NEUROT           22.454    0.262   85.661    0.000   21.940   22.968
##    Std.lv  Std.all
##     0.685    1.000
##     1.738    1.000
##     2.858    1.000
##    14.024    0.997
##    10.626    0.996
##    15.453    0.998
##     9.272    0.997
##    22.454    0.997
```

## Model 0b: Cognition –> Big5

```
## lavaan 0.6-18 ended normally after 246 iterations
## 
##   Estimator                                         ML
##   Optimization method                           NLMINB
##   Number of model parameters                        62
## 
##   Number of observations                          8386
## 
## Model Test User Model:
##                                               Standard      Scaled
##   Test Statistic                                99.675      75.771
##   Degrees of freedom                                15          15
##   P-value (Chi-square)                           0.000       0.000
##   Scaling correction factor                                  1.315
##     Satorra-Bentler correction                                    
## 
## Model Test Baseline Model:
## 
##   Test statistic                              7139.065    5116.263
##   Degrees of freedom                                55          55
##   P-value                                        0.000       0.000
##   Scaling correction factor                                  1.395
## 
## User Model versus Baseline Model:
## 
##   Comparative Fit Index (CFI)                    0.988       0.988
##   Tucker-Lewis Index (TLI)                       0.956       0.956
##                                                                   
##   Robust Comparative Fit Index (CFI)                         0.989
##   Robust Tucker-Lewis Index (TLI)                            0.958
## 
## Loglikelihood and Information Criteria:
## 
##   Loglikelihood user model (H0)            -240591.347 -240591.347
##   Loglikelihood unrestricted model (H1)    -240541.510 -240541.510
##                                                                   
##   Akaike (AIC)                              481306.694  481306.694
##   Bayesian (BIC)                            481742.822  481742.822
##   Sample-size adjusted Bayesian (SABIC)     481545.797  481545.797
## 
## Root Mean Square Error of Approximation:
## 
##   RMSEA                                          0.026       0.022
##   90 Percent confidence interval - lower         0.021       0.018
##   90 Percent confidence interval - upper         0.031       0.026
##   P-value H_0: RMSEA <= 0.050                    1.000       1.000
##   P-value H_0: RMSEA >= 0.080                    0.000       0.000
##                                                                   
##   Robust RMSEA                                               0.025
##   90 Percent confidence interval - lower                     0.020
##   90 Percent confidence interval - upper                     0.031
##   P-value H_0: Robust RMSEA <= 0.050                         1.000
##   P-value H_0: Robust RMSEA >= 0.080                         0.000
## 
## Standardized Root Mean Square Residual:
## 
##   SRMR                                           0.009       0.009
## 
## Parameter Estimates:
## 
##   Standard errors                           Robust.sem
##   Information                                 Expected
##   Information saturated (h1) model          Structured
## 
## Latent Variables:
##                             Estimate  Std.Err  z-value  P(>|z|) ci.lower
##   Cognitive_ability_age5 =~                                             
##     Verbal_blty_g5             1.000                               1.000
##     Spatil_blty_g5             0.782    0.055   14.170    0.000    0.674
##   Self_regulation_age5 =~                                               
##     Independenc_g5             1.000                               1.000
##     Emotion_rgl_g5             1.187    0.049   24.094    0.000    1.091
##  ci.upper   Std.lv  Std.all
##                            
##     1.000    6.520    0.611
##     0.891    5.101    0.530
##                            
##     1.000    1.922    0.528
##     1.284    2.282    0.484
## 
## Regressions:
##                    Estimate  Std.Err  z-value  P(>|z|) ci.lower ci.upper
##   OPEN ~                                                                
##     ToM_age5         -0.216    0.134   -1.616    0.106   -0.478    0.046
##     Prosocialty_g5   -0.056    0.069   -0.817    0.414   -0.190    0.078
##     Cogntv_blty_g5    0.073    0.019    3.822    0.000    0.036    0.111
##     Self_regltn_g5    0.147    0.097    1.513    0.130   -0.043    0.337
##   CONSC ~                                                               
##     ToM_age5          0.311    0.110    2.817    0.005    0.095    0.527
##     Prosocialty_g5   -0.058    0.063   -0.923    0.356   -0.182    0.066
##     Cogntv_blty_g5    0.004    0.015    0.250    0.803   -0.025    0.032
##     Self_regltn_g5    0.321    0.077    4.145    0.000    0.169    0.473
##   EXTRAV ~                                                              
##     ToM_age5          0.382    0.141    2.709    0.007    0.106    0.658
##     Prosocialty_g5    0.026    0.072    0.356    0.722   -0.115    0.167
##     Cogntv_blty_g5   -0.016    0.018   -0.885    0.376   -0.052    0.020
##     Self_regltn_g5    0.143    0.101    1.423    0.155   -0.054    0.340
##   AGREE ~                                                               
##     ToM_age5          0.209    0.110    1.906    0.057   -0.006    0.424
##     Prosocialty_g5   -0.039    0.062   -0.622    0.534   -0.160    0.083
##     Cogntv_blty_g5   -0.049    0.016   -2.949    0.003   -0.081   -0.016
##     Self_regltn_g5    0.358    0.081    4.400    0.000    0.199    0.517
##   NEUROT ~                                                              
##     ToM_age5          0.160    0.178    0.896    0.370   -0.190    0.509
##     Prosocialty_g5    0.366    0.090    4.054    0.000    0.189    0.543
##     Cogntv_blty_g5    0.082    0.023    3.633    0.000    0.038    0.127
##     Self_regltn_g5   -0.452    0.121   -3.730    0.000   -0.689   -0.214
##    Std.lv  Std.all
##                   
##    -0.216   -0.021
##    -0.056   -0.024
##     0.478    0.128
##     0.282    0.076
##                   
##     0.311    0.035
##    -0.058   -0.029
##     0.024    0.007
##     0.617    0.189
##                   
##     0.382    0.035
##     0.026    0.011
##    -0.106   -0.027
##     0.275    0.070
##                   
##     0.209    0.025
##    -0.039   -0.021
##    -0.317   -0.104
##     0.688    0.226
##                   
##     0.160    0.012
##     0.366    0.125
##     0.537    0.113
##    -0.868   -0.182
## 
## Covariances:
##                             Estimate  Std.Err  z-value  P(>|z|) ci.lower
##   ToM_age5 ~~                                                           
##     Prosocialty_g5             0.025    0.007    3.723    0.000    0.012
##   Cognitive_ability_age5 ~~                                             
##     ToM_age5                   0.384    0.040    9.556    0.000    0.305
##   Self_regulation_age5 ~~                                               
##     ToM_age5                   0.063    0.014    4.500    0.000    0.036
##   Cognitive_ability_age5 ~~                                             
##     Prosocialty_g5             1.471    0.220    6.683    0.000    1.040
##   Self_regulation_age5 ~~                                               
##     Prosocialty_g5             2.001    0.085   23.611    0.000    1.835
##   Cognitive_ability_age5 ~~                                             
##     Self_regltn_g5             6.467    0.468   13.822    0.000    5.550
##  .OPEN ~~                                                               
##    .CONSC                      1.836    0.174   10.541    0.000    1.495
##    .EXTRAV                     1.970    0.196   10.053    0.000    1.586
##    .AGREE                      2.353    0.165   14.269    0.000    2.030
##    .NEUROT                    -0.055    0.231   -0.237    0.812   -0.507
##  .CONSC ~~                                                              
##    .EXTRAV                     2.134    0.169   12.632    0.000    1.803
##    .AGREE                      2.882    0.156   18.533    0.000    2.577
##    .NEUROT                    -3.409    0.200  -17.066    0.000   -3.800
##  .EXTRAV ~~                                                             
##    .AGREE                      1.505    0.170    8.831    0.000    1.171
##    .NEUROT                    -5.495    0.244  -22.552    0.000   -5.973
##  .AGREE ~~                                                              
##    .NEUROT                     0.170    0.181    0.939    0.348   -0.185
##  ci.upper   Std.lv  Std.all
##                            
##     0.038    0.025    0.042
##                            
##     0.463    0.059    0.162
##                            
##     0.091    0.033    0.090
##                            
##     1.903    0.226    0.138
##                            
##     2.167    1.041    0.639
##                            
##     7.383    0.516    0.516
##                            
##     2.178    1.836    0.156
##     2.354    1.970    0.136
##     2.676    2.353    0.214
##     0.398   -0.055   -0.003
##                            
##     2.465    2.134    0.169
##     3.187    2.882    0.300
##    -3.017   -3.409   -0.225
##                            
##     1.839    1.505    0.128
##    -5.018   -5.495   -0.295
##                            
##     0.524    0.170    0.012
## 
## Intercepts:
##                    Estimate  Std.Err  z-value  P(>|z|) ci.lower ci.upper
##    .Verbal_blty_g5   55.908    0.241  232.190    0.000   55.436   56.380
##    .Spatil_blty_g5   51.548    0.190  271.170    0.000   51.176   51.921
##    .Independenc_g5   16.457    0.050  331.142    0.000   16.360   16.554
##    .Emotion_rgl_g5   14.196    0.070  201.787    0.000   14.058   14.334
##    .OPEN             14.996    0.645   23.249    0.000   13.732   16.260
##    .CONSC            14.418    0.604   23.867    0.000   13.234   15.602
##    .EXTRAV           12.857    0.666   19.311    0.000   11.552   14.162
##    .AGREE            16.710    0.587   28.466    0.000   15.560   17.861
##    .NEUROT            8.375    0.853    9.823    0.000    6.704   10.046
##     ToM_age5          1.157    0.005  223.932    0.000    1.147    1.167
##     Prosocialty_g5    9.426    0.021  458.885    0.000    9.386    9.467
##    Std.lv  Std.all
##    55.908    5.236
##    51.548    5.360
##    16.457    4.517
##    14.196    3.010
##    14.996    4.021
##    14.418    4.417
##    12.857    3.252
##    16.710    5.486
##     8.375    1.758
##     1.157    3.181
##     9.426    5.785
## 
## Variances:
##                    Estimate  Std.Err  z-value  P(>|z|) ci.lower ci.upper
##     ToM_age5          0.132    0.004   37.279    0.000    0.125    0.139
##     Prosocialty_g5    2.655    0.061   43.602    0.000    2.535    2.774
##     Cogntv_blty_g5   42.510    3.499   12.148    0.000   35.651   49.369
##     Self_regltn_g5    3.695    0.228   16.189    0.000    3.248    4.143
##    .Verbal_blty_g5   71.519    3.400   21.033    0.000   64.855   78.184
##    .Spatil_blty_g5   66.478    2.811   23.648    0.000   60.969   71.988
##    .Independenc_g5    9.580    0.254   37.776    0.000    9.083   10.077
##    .Emotion_rgl_g5   17.033    0.381   44.738    0.000   16.286   17.779
##    .OPEN             13.507    0.221   61.222    0.000   13.074   13.939
##    .CONSC            10.301    0.201   51.360    0.000    9.908   10.694
##    .EXTRAV           15.536    0.258   60.112    0.000   15.029   16.042
##    .AGREE             8.967    0.216   41.596    0.000    8.544    9.389
##    .NEUROT           22.347    0.295   75.761    0.000   21.769   22.925
##    Std.lv  Std.all
##     0.132    1.000
##     2.655    1.000
##     1.000    1.000
##     1.000    1.000
##    71.519    0.627
##    66.478    0.719
##     9.580    0.722
##    17.033    0.766
##    13.507    0.971
##    10.301    0.967
##    15.536    0.994
##     8.967    0.967
##    22.347    0.985
```

## Model 0a: Motor Function –> Cognition

```
## lavaan 0.6-18 ended normally after 179 iterations
## 
##   Estimator                                         ML
##   Optimization method                           NLMINB
##   Number of model parameters                        43
## 
##   Number of observations                          8375
## 
## Model Test User Model:
##                                               Standard      Scaled
##   Test Statistic                               148.373     114.456
##   Degrees of freedom                                11          11
##   P-value (Chi-square)                           0.000       0.000
##   Scaling correction factor                                  1.296
##     Satorra-Bentler correction                                    
## 
## Model Test Baseline Model:
## 
##   Test statistic                              5787.790    3742.656
##   Degrees of freedom                                36          36
##   P-value                                        0.000       0.000
##   Scaling correction factor                                  1.546
## 
## User Model versus Baseline Model:
## 
##   Comparative Fit Index (CFI)                    0.976       0.972
##   Tucker-Lewis Index (TLI)                       0.922       0.909
##                                                                   
##   Robust Comparative Fit Index (CFI)                         0.977
##   Robust Tucker-Lewis Index (TLI)                            0.923
## 
## Loglikelihood and Information Criteria:
## 
##   Loglikelihood user model (H0)            -167235.548 -167235.548
##   Loglikelihood unrestricted model (H1)    -167161.362 -167161.362
##                                                                   
##   Akaike (AIC)                              334557.097  334557.097
##   Bayesian (BIC)                            334859.516  334859.516
##   Sample-size adjusted Bayesian (SABIC)     334722.870  334722.870
## 
## Root Mean Square Error of Approximation:
## 
##   RMSEA                                          0.039       0.034
##   90 Percent confidence interval - lower         0.033       0.029
##   90 Percent confidence interval - upper         0.044       0.038
##   P-value H_0: RMSEA <= 0.050                    1.000       1.000
##   P-value H_0: RMSEA >= 0.080                    0.000       0.000
##                                                                   
##   Robust RMSEA                                               0.038
##   90 Percent confidence interval - lower                     0.032
##   90 Percent confidence interval - upper                     0.045
##   P-value H_0: Robust RMSEA <= 0.050                         0.999
##   P-value H_0: Robust RMSEA >= 0.080                         0.000
## 
## Standardized Root Mean Square Residual:
## 
##   SRMR                                           0.016       0.016
## 
## Parameter Estimates:
## 
##   Standard errors                           Robust.sem
##   Information                                 Expected
##   Information saturated (h1) model          Structured
## 
## Latent Variables:
##                             Estimate  Std.Err  z-value  P(>|z|) ci.lower
##   Cognitive_ability_age5 =~                                             
##     Verbal_blty_g5             1.000                               1.000
##     Spatil_blty_g5             0.864    0.066   13.079    0.000    0.734
##   Self_regulation_age5 =~                                               
##     Independenc_g5             1.000                               1.000
##     Emotion_rgl_g5             1.126    0.048   23.674    0.000    1.033
##  ci.upper   Std.lv  Std.all
##                            
##     1.000    6.207    0.581
##     0.993    5.362    0.557
##                            
##     1.000    1.973    0.541
##     1.219    2.222    0.471
## 
## Regressions:
##                            Estimate  Std.Err  z-value  P(>|z|) ci.lower
##   ToM_age5 ~                                                           
##     Motor_fine               -0.007    0.006   -1.218    0.223   -0.019
##     Motor_gross              -0.002    0.003   -0.521    0.602   -0.009
##     Communication             0.007    0.003    2.356    0.018    0.001
##   Prosociality_age5 ~                                                  
##     Motor_fine                0.187    0.030    6.236    0.000    0.128
##     Motor_gross              -0.019    0.017   -1.144    0.253   -0.051
##     Communication             0.135    0.014    9.934    0.000    0.108
##   Cognitive_ability_age5 ~                                             
##     Motor_fine                0.727    0.161    4.507    0.000    0.411
##     Motor_gross               0.362    0.098    3.703    0.000    0.170
##     Communication             0.063    0.080    0.781    0.435   -0.095
##   Self_regulation_age5 ~                                               
##     Motor_fine                0.334    0.048    6.934    0.000    0.240
##     Motor_gross               0.037    0.030    1.244    0.214   -0.021
##     Communication             0.165    0.025    6.488    0.000    0.115
##  ci.upper   Std.lv  Std.all
##                            
##     0.004   -0.007   -0.016
##     0.005   -0.002   -0.006
##     0.012    0.007    0.030
##                            
##     0.246    0.187    0.094
##     0.014   -0.019   -0.015
##     0.162    0.135    0.139
##                            
##     1.043    0.117    0.095
##     0.553    0.058    0.076
##     0.220    0.010    0.017
##                            
##     0.429    0.169    0.138
##     0.096    0.019    0.025
##     0.215    0.084    0.140
## 
## Covariances:
##                             Estimate  Std.Err  z-value  P(>|z|) ci.lower
##   Motor_fine ~~                                                         
##     Motor_gross                0.201    0.019   10.636    0.000    0.164
##     Communication              0.361    0.023   15.692    0.000    0.316
##   Motor_gross ~~                                                        
##     Communication              0.704    0.032   21.813    0.000    0.641
##  .Cognitive_ability_age5 ~~                                             
##    .Self_regltn_g5             6.035    0.463   13.038    0.000    5.128
##    .ToM_age5                   0.360    0.040    9.084    0.000    0.282
##    .Prosocialty_g5             1.215    0.203    5.975    0.000    0.817
##  .Self_regulation_age5 ~~                                               
##    .ToM_age5                   0.063    0.014    4.408    0.000    0.035
##    .Prosocialty_g5             1.918    0.081   23.786    0.000    1.760
##  .ToM_age5 ~~                                                           
##    .Prosocialty_g5             0.024    0.007    3.540    0.000    0.011
##  ci.upper   Std.lv  Std.all
##                            
##     0.238    0.201    0.189
##     0.406    0.361    0.264
##                            
##     0.768    0.704    0.321
##                            
##     6.943    0.511    0.511
##     0.438    0.059    0.161
##     1.614    0.198    0.123
##                            
##     0.091    0.033    0.090
##     2.076    0.999    0.624
##                            
##     0.037    0.024    0.041
## 
## Intercepts:
##                    Estimate  Std.Err  z-value  P(>|z|) ci.lower ci.upper
##    .Verbal_blty_g5   48.003    1.310   36.640    0.000   45.435   50.570
##    .Spatil_blty_g5   44.710    1.163   38.435    0.000   42.430   46.990
##    .Independenc_g5   12.645    0.374   33.830    0.000   11.912   13.377
##    .Emotion_rgl_g5    9.898    0.436   22.688    0.000    9.043   10.753
##    .ToM_age5          1.180    0.045   26.109    0.000    1.091    1.268
##    .Prosocialty_g5    7.231    0.237   30.451    0.000    6.766    7.697
##     Motor_fine        7.588    0.013  569.208    0.000    7.562    7.614
##     Motor_gross       5.504    0.017  322.781    0.000    5.470    5.537
##     Communication     6.497    0.026  253.464    0.000    6.447    6.547
##    Std.lv  Std.all
##    48.003    4.496
##    44.710    4.647
##    12.645    3.470
##     9.898    2.099
##     1.180    3.242
##     7.231    4.439
##     7.588    9.310
##     5.504    4.213
##     6.497    3.867
## 
## Variances:
##                    Estimate  Std.Err  z-value  P(>|z|) ci.lower ci.upper
##     Motor_fine        0.664    0.026   25.554    0.000    0.613    0.715
##     Motor_gross       1.707    0.040   42.867    0.000    1.629    1.785
##     Communication     2.824    0.050   56.087    0.000    2.725    2.922
##    .Verbal_blty_g5   75.469    3.386   22.288    0.000   68.832   82.105
##    .Spatil_blty_g5   63.815    3.038   21.008    0.000   57.862   69.769
##    .Independenc_g5    9.385    0.261   35.947    0.000    8.873    9.896
##    .Emotion_rgl_g5   17.296    0.376   46.020    0.000   16.560   18.033
##    .ToM_age5          0.132    0.004   37.159    0.000    0.125    0.139
##    .Prosocialty_g5    2.566    0.058   43.956    0.000    2.451    2.680
##    .Cogntv_blty_g5   37.776    3.433   11.004    0.000   31.047   44.504
##    .Self_regltn_g5    3.686    0.232   15.914    0.000    3.232    4.140
##    Std.lv  Std.all
##     0.664    1.000
##     1.707    1.000
##     2.824    1.000
##    75.469    0.662
##    63.815    0.689
##     9.385    0.707
##    17.296    0.778
##     0.132    0.999
##     2.566    0.967
##     0.980    0.980
##     0.947    0.947
```

## Results table - initial models (unimputed)

|  |  |  |  |
| --- | --- | --- | --- |
|  | Motor - Big5 | Cog - Big5 | Motor - Cog |
|  | Estimate (Std. Err.) | Estimate (Std. Err.) | Estimate (Std. Err.) |
|  | Regression Slopes | | |
| OPEN |
| Motor.fine | 0.09(0.06) |  |  |
| Motor.gross | 0.12(0.04)\*\*\* |  |  |
| Communication | 0.04(0.03) |  |  |
| Theory of mind (age 5) |  | -0.22(0.13) |  |
| Prosociality (age 5) |  | -0.06(0.07) |  |
| Cognitive.ability.age5 |  | 0.07(0.02)\*\*\* |  |
| Self.regulation.age5 |  | 0.15(0.10) |  |
| CONSC |
| Motor.fine | 0.12(0.05)\* |  |  |
| Motor.gross | 0.04(0.03) |  |  |
| Communication | 0.07(0.02)\*\* |  |  |
| Theory of mind (age 5) |  | 0.31(0.11)\*\* |  |
| Prosociality (age 5) |  | -0.06(0.06) |  |
| Cognitive.ability.age5 |  | 0.00(0.01) |  |
| Self.regulation.age5 |  | 0.32(0.08)\*\*\* |  |
| EXTRAV |
| Motor.fine | 0.07(0.06) |  |  |
| Motor.gross | 0.03(0.04) |  |  |
| Communication | 0.09(0.03)\*\* |  |  |
| Theory of mind (age 5) |  | 0.38(0.14)\*\* |  |
| Prosociality (age 5) |  | 0.03(0.07) |  |
| Cognitive.ability.age5 |  | -0.02(0.02) |  |
| Self.regulation.age5 |  | 0.14(0.10) |  |
| AGREE |
| Motor.fine | 0.12(0.05)\*\* |  |  |
| Motor.gross | -0.05(0.03) |  |  |
| Communication | 0.08(0.03)\*\* |  |  |
| Theory of mind (age 5) |  | 0.21(0.11) |  |
| Prosociality (age 5) |  | -0.04(0.06) |  |
| Cognitive.ability.age5 |  | -0.05(0.02)\*\* |  |
| Self.regulation.age5 |  | 0.36(0.08)\*\*\* |  |
| NEUROT |
| Motor.fine | 0.09(0.07) |  |  |
| Motor.gross | -0.20(0.04)\*\*\* |  |  |
| Communication | 0.07(0.04)\* |  |  |
| Theory of mind (age 5) |  | 0.16(0.18) |  |
| Prosociality (age 5) |  | 0.37(0.09)\*\*\* |  |
| Cognitive.ability.age5 |  | 0.08(0.02)\*\*\* |  |
| Self.regulation.age5 |  | -0.45(0.12)\*\*\* |  |
| Theory of mind (age 5) |
| Motor.fine |  |  | -0.01(0.01) |
| Motor.gross |  |  | -0.00(0.00) |
| Communication |  |  | 0.01(0.00)\* |
| Prosociality (age 5) |
| Motor.fine |  |  | 0.19(0.03)\*\*\* |
| Motor.gross |  |  | -0.02(0.02) |
| Communication |  |  | 0.14(0.01)\*\*\* |
| Cognitive\_ability\_age5 |
| Motor.fine |  |  | 0.73(0.16)\*\*\* |
| Motor.gross |  |  | 0.36(0.10)\*\*\* |
| Communication |  |  | 0.06(0.08) |
| Self\_regulation\_age5 |
| Motor.fine |  |  | 0.33(0.05)\*\*\* |
| Motor.gross |  |  | 0.04(0.03) |
| Communication |  |  | 0.16(0.03)\*\*\* |
|  | Intercepts | | |
| OPEN | 12.60(0.46)\*\*\* | 15.00(0.65)\*\*\* |  |
| CONSC | 12.61(0.37)\*\*\* | 14.42(0.60)\*\*\* |  |
| EXTRAV | 12.33(0.46)\*\*\* | 12.86(0.67)\*\*\* |  |
| AGREE | 15.41(0.37)\*\*\* | 16.71(0.59)\*\*\* |  |
| NEUROT | 11.96(0.51)\*\*\* | 8.38(0.85)\*\*\* |  |
| Motor.fine | 7.58(0.01)\*\*\* |  | 7.59(0.01)\*\*\* |
| Motor.gross | 5.50(0.02)\*\*\* |  | 5.50(0.02)\*\*\* |
| Communication | 6.51(0.03)\*\*\* |  | 6.50(0.03)\*\*\* |
| Verbal ability (age 5) |  | 55.91(0.24)\*\*\* | 48.00(1.31)\*\*\* |
| Spatial ability (age 5) |  | 51.55(0.19)\*\*\* | 44.71(1.16)\*\*\* |
| Independence skills (age 5) |  | 16.46(0.05)\*\*\* | 12.64(0.37)\*\*\* |
| Emotion regulation (age 5) |  | 14.20(0.07)\*\*\* | 9.90(0.44)\*\*\* |
| Theory of mind (age 5) |  | 1.16(0.01)\*\*\* | 1.18(0.05)\*\*\* |
| Prosociality (age 5) |  | 9.43(0.02)\*\*\* | 7.23(0.24)\*\*\* |
|  | Fit Indices | | |
| χ2 | 0.00 | 99.67 | 148.37 |
| CFI | 1.00 | 0.99 | 0.98 |
| TLI | 1.00 | 0.96 | 0.92 |
| RMSEA | 0.00 | 0.03 | 0.04 |
| Scaled χ2 | 0.00(0) | 75.77(15)\*\*\* | 114.46(11)\*\*\* |
| +Fixed parameter | | | |
| \*p<0.05, \*\*p<0.01, \*\*\*p<0.001 | | | |

|  |  |  |  |
| --- | --- | --- | --- |
|  | Motor - Big5 | Cog - Big5 | Motor - Cog |
|  | Estimate (Std. Err.) | Estimate (Std. Err.) | Estimate (Std. Err.) |
|  | Regression Slopes | | |
| OPEN |
| Motor.fine | 0.09(0.06) |  |  |
| Motor.gross | 0.12(0.04)\*\*\* |  |  |
| Communication | 0.04(0.03) |  |  |
| Theory of mind (age 5) |  | -0.22(0.13) |  |
| Prosociality (age 5) |  | -0.06(0.07) |  |
| Cognitive.ability.age5 |  | 0.07(0.02)\*\*\* |  |
| Self.regulation.age5 |  | 0.15(0.10) |  |
| CONSC |
| Motor.fine | 0.12(0.05)\* |  |  |
| Motor.gross | 0.04(0.03) |  |  |
| Communication | 0.07(0.02)\*\* |  |  |
| Theory of mind (age 5) |  | 0.31(0.11)\*\* |  |
| Prosociality (age 5) |  | -0.06(0.06) |  |
| Cognitive.ability.age5 |  | 0.00(0.01) |  |
| Self.regulation.age5 |  | 0.32(0.08)\*\*\* |  |
| EXTRAV |
| Motor.fine | 0.07(0.06) |  |  |
| Motor.gross | 0.03(0.04) |  |  |
| Communication | 0.09(0.03)\*\* |  |  |
| Theory of mind (age 5) |  | 0.38(0.14)\*\* |  |
| Prosociality (age 5) |  | 0.03(0.07) |  |
| Cognitive.ability.age5 |  | -0.02(0.02) |  |
| Self.regulation.age5 |  | 0.14(0.10) |  |
| AGREE |
| Motor.fine | 0.12(0.05)\*\* |  |  |
| Motor.gross | -0.05(0.03) |  |  |
| Communication | 0.08(0.03)\*\* |  |  |
| Theory of mind (age 5) |  | 0.21(0.11) |  |
| Prosociality (age 5) |  | -0.04(0.06) |  |
| Cognitive.ability.age5 |  | -0.05(0.02)\*\* |  |
| Self.regulation.age5 |  | 0.36(0.08)\*\*\* |  |
| NEUROT |
| Motor.fine | 0.09(0.07) |  |  |
| Motor.gross | -0.20(0.04)\*\*\* |  |  |
| Communication | 0.07(0.04)\* |  |  |
| Theory of mind (age 5) |  | 0.16(0.18) |  |
| Prosociality (age 5) |  | 0.37(0.09)\*\*\* |  |
| Cognitive.ability.age5 |  | 0.08(0.02)\*\*\* |  |
| Self.regulation.age5 |  | -0.45(0.12)\*\*\* |  |
| Theory of mind (age 5) |
| Motor.fine |  |  | -0.01(0.01) |
| Motor.gross |  |  | -0.00(0.00) |
| Communication |  |  | 0.01(0.00)\* |
| Prosociality (age 5) |
| Motor.fine |  |  | 0.19(0.03)\*\*\* |
| Motor.gross |  |  | -0.02(0.02) |
| Communication |  |  | 0.14(0.01)\*\*\* |
[truncated: 585,909 more chars]
